# Stress-inducible phosphoprotein 1 (HOP/STI1/STIP1) regulates the accumulation and toxicity of α-synuclein in vivo

**DOI:** 10.1007/s00401-022-02491-8

**Published:** 2022-09-19

**Authors:** Rachel E. Lackie, Aline S. de Miranda, Mei Peng Lim, Vladislav Novikov, Nimrod Madrer, Nadun C. Karunatilleke, Benjamin S. Rutledge, Stephanie Tullo, Anne Brickenden, Matthew E. R. Maitland, David Greenberg, Daniel Gallino, Wen Luo, Anoosha Attaran, Irina Shlaifer, Esther Del Cid Pellitero, Caroline Schild-Poulter, Thomas M. Durcan, Edward A. Fon, Martin Duennwald, Flavio H. Beraldo, M. Mallar Chakravarty, Timothy J. Bussey, Lisa M. Saksida, Hermona Soreq, Wing-Yiu Choy, Vania F. Prado, Marco A. M. Prado

**Affiliations:** 1 Robarts Research Institute, The University of Western Ontario, 1151 Richmond St. N, London, ON N6A 5B7 Canada; 2 Program in Neuroscience, The University of Western Ontario, London, Canada; 3 Laboratory of Neurobiology, Department of Morphology, Institute of Biological Science, Universidade Federal de Minas Gerais (UFMG), Belo Horizonte, Brazil; 4 The Edmond and Lily Safra Center for Brain Sciences, Department of Biological Chemistry, The Alexander Silberman Institute of Life Sciences, The Hebrew University of Jerusalem, Jerusalem, Israel; 5 Department of Biochemistry, The University of Western Ontario, 1151 Richmond St. N, London, ON N6A 5B7 Canada; 6 Cerebral Imaging Centre, Douglas Research Institute, McGill University, Montreal, Canada; 7 Early Drug Discovery Unit, Montreal Neurological Institute, McGill University, McGill Parkinson Program, Neurodegenerative Diseases Group, Department of Neurology and Neurosurgery, Montreal Neurological Institute, McGill University, Montreal, Canada; 8 Department of Anatomy & Cell Biology, The University of Western Ontario, London, Canada; 9 Department of Physiology and Pharmacology, The University of Western Ontario, London, Canada

**Keywords:** Chaperone, Hsp90, Hsp70, STIP1, HOP, Parkinson’s, Lewy body, Touchscreens, α-Synuclein, Neuropathology, A53T, Pre-formed fibrils

## Abstract

The predominantly pre-synaptic intrinsically disordered protein α-synuclein is prone to misfolding and aggregation in synucleinopathies, such as Parkinson’s disease (PD) and Dementia with Lewy bodies (DLB). Molecular chaperones play important roles in protein misfolding diseases and members of the chaperone machinery are often deposited in Lewy bodies. Here, we show that the Hsp90 co-chaperone STI1 co-immunoprecipitated α-synuclein, and co-deposited with Hsp90 and Hsp70 in insoluble protein fractions in two mouse models of α-synuclein misfolding. STI1 and Hsp90 also co-localized extensively with filamentous S129 phosphorylated α-synuclein in ubiquitin-positive inclusions. In PD human brains, STI1 transcripts were increased, and in neurologically healthy brains, STI1 and α-synuclein transcripts correlated. Nuclear Magnetic Resonance (NMR) analyses revealed direct interaction of α-synuclein with STI1 and indicated that the STI1 TPR2A, but not TPR1 or TPR2B domains, interacted with the C-terminal domain of α-synuclein. In vitro, the STI1 TPR2A domain facilitated S129 phosphorylation by Polo-like kinase 3. Moreover, mice over-expressing STI1 and Hsp90ß presented elevated α-synuclein S129 phosphorylation accompanied by inclusions when injected with α-synuclein pre-formed fibrils. In contrast, reduced STI1 function decreased protein inclusion formation, S129 α-synuclein phosphorylation, while mitigating motor and cognitive deficits as well as mesoscopic brain atrophy in α-synuclein-over-expressing mice. Our findings reveal a vicious cycle in which STI1 facilitates the generation and accumulation of toxic α-synuclein conformers, while α-synuclein-induced proteostatic stress increased insoluble STI1 and Hsp90.

**Supplementary Information:**

The online version contains supplementary material available at 10.1007/s00401-022-02491-8.

## Introduction

The α-synuclein gene (*SNCA*) is causally linked to Parkinson’s disease (PD) and Dementia with Lewy bodies (DLB) [5, 96, 112, 113], protein misfolding diseases collectively known as synucleinopathies. In these diseases, α-synuclein protein misfolds, is cleaved and aggregates into proteinaceous and membranous inclusions known as Lewy bodies and Lewy neurites, where it is found to be phosphorylated at serine residue 129 (S129) [3, 53, 123]. Notably, higher expression and mutations in *SNCA* increase α-synuclein protein propensity to misfold [33, 34]. Somewhere in the continuum between misfolding and deposition in Lewy bodies, α-synuclein becomes toxic, leading to malfunction and degeneration of vulnerable neurons.

α-synuclein is an abundant pre-synaptic protein that associates with synaptic vesicle membranes [1, 22, 24, 88, 91, 124]. At membranes, α-synuclein can reorganize into ordered high-molecular-weight helical multimers [23, 124], including tetramers that seem to be necessary for its physiologic functions [7, 50, 59]. Recent work suggested that most cytoplasmic α-synuclein seems to be complexed with molecular chaperones in cells when it is not associated with membranes [21].

The concerted actions of chaperones and co-chaperones are essential for protein folding, stabilization, refolding, and targeting for degradation; their involvement in synucleinopathies is broadly supported [45, 63, 99]. Notably, the chaperone Hsp90 is abundantly deposited in Lewy bodies and has been associated with insoluble protein fractions in both humans with PD and mouse models of synucleinopathies [19, 79, 121]. Interestingly, inhibition of Hsp90 in cultured cells promoted α-synuclein interaction with mitochondrial membranes and its oligomerization [21].

Other chaperones and co-chaperones are also found in Lewy bodies [79, 90, 121]. Recent proteomics analysis of Lewy body formation in cultured primary neurons detected early accumulation of the Hsp90 regulatory co-chaperone Stress-inducible phosphoprotein 1 (*STIP1*, STI1 or HOP for Hsp-organizing protein) with misfolded α-synuclein [86]. STI1 is important for the chaperone cycle by bridging Hsp70 and Hsp90 and regulating Hsp90 ATPase activity [37, 68, 77, 125]. STI1 also works as a scaffold to recruit proteins to Hsp90 [116, 125] and can be secreted by cells to provide neuroprotective signals via the prion protein [9, 10, 12, 81, 129]. Notably, STI1 and Hsp90 can reduce α-synuclein oligomerization in vitro [38], and Hsp90 has also been suggested to either decrease or increase fibril formation depending on the presence of ATP [47]. STI1 is also one of the co-chaperones found to interact with α-synuclein in a proteomics analysis in cultured cells [21]. In response to proteostatic stress, the chaperone machinery (the chaperome) becomes abnormally connected, forming the epichaperome, which is deleterious for neuronal function [57, 64]. STI1 is a critical epichaperome regulator and reducing the levels of STI1 collapsed the abnormal connectivity of the epichaperome in cancer cells [30, 102].

The role played by chaperones and co-chaperones in α-synuclein misfolding and toxicity and how misfolded α-synuclein affects chaperome function is incompletely understood. Here, we report that STI1 mRNA is increased in Parkinson’s brains, and we demonstrate a direct interaction of the TPR2A domain of STI1 with the C-terminal region of α-synuclein, which seems to play a role in regulating α-synuclein phosphorylation. Mouse models of synucleinopathy present increased levels of aggregated chaperones and STI1; however, elevated levels of STI1 and Hsp90 increased aggregation of α-synuclein and its S129 phosphorylation (psyn129) in vivo. Conversely, reduced levels of STI1 decrease psyn129 inclusions, brain atrophy, and rescued high-level cognitive deficits in a mouse model of synucleinopathy. These findings illuminate the roles of STI1 in regulating the accumulation and toxicity of misfolded α-synuclein in vivo and suggest that targeting the interaction of STI1 with α-synuclein may modulate disease progression in synucleinopathies.

## Materials and methods

### Animals

Mice over-expressing the human A53T mutation in the *SNCA* gene under control of the mouse prion promoter [56, 82] (M83 line) were obtained from Jackson Laboratory on a C57BL/C3H background (B6;C3-Tg(Prnp-SNCA*A53T)83Vle/J, RRID:IMSR_JAX:004,479). STI1-ΔTPR1 and TgA mice, both on C57BL/6 backgrounds were generated as described previously [13, 75]. Homozygous M83 (+/+) mice were bred with STI1-ΔTPR1 heterozygous mice [75] or STI1-TgA+ mice [13]. Subsequent breeding generated M83 hemizygous (+/−) or M83 homozygous (+/+) containing different sets of STI1 alleles including STI1 wild type (STI1WT), STI1 heterozygous (WT/ΔTPR1; ΔHET), or homozygous (ΔTPR1/ΔTPR1; ΔTPR1). Homozygous M83 (+/+) mice were also bred with STI1-TgA+ mice [13] to generate M83 hemizygous (+/−) containing either normal levels of STI1 or over-expressing STI1(M83+/−:TgA). Mice were generated and maintained on the mixed C57BL/C3H and C57BL/6 backgrounds. Littermates were used for experiments.

Mice were housed in standard plexiglass cages and were given ad libitum access to food (Harlan), unless they were used for behavioral experiments (see below), and ad libitum access to water in temperature and humidity-controlled rooms (22–25 °C and with 40–60%, respectively) on a light/dark cycle from 7 am to 7 pm. M83+/+ and M83+/− mice were usually housed alone due to aggressive behavior, or with one-two other littermates if fighting was not present. For M83+/−:STI1 mouse studies, mice were aged to 3–4 months before receiving intracerebral injection of wild-type human α-synuclein pre-formed fibrils, and then, brain tissue was collected 3–4 months after surgery. M83+/+ :ΔTPR1 mice were aged to 11–12 months (at this age, 50% of M83+/+ cohort showed body weight loss and motor impairments [56]), and then, tissues were collected for experiments. Given the exploratory nature of these initial experiments and the correlations found in humans for *STIP1* and *HSP90AB1* in men and the predominance of PD in men [6], sex was not studied as a biological variable and we used male mice for all the experiments.

### Animal housing and food restriction for cognitive and motor behavior analysis

Non-transgenic (WT), M83+/+ :STI1WT, and M83+/+ :ΔHET mice that underwent behavioral tasks were singly housed (due to fighting and aggressive behavior). Minimum environmental enrichment was provided to the mice and cages were changed weekly. All tasks in the touchscreen battery are motivated by strawberry milkshake (Nielson—Saputo Dairy Products) reward. Thus, to ensure adequate motivation to work for food rewards, mice (12–14 weeks or older) were food-restricted at least 2 weeks prior to the start of behavioral testing and were maintained at 85% of free feeding body weight until the end of the touchscreen experiments [11, 13]. All mice were weighed, and food pellets ranging from 1.5–3 g (3.35 kcal/gram) were delivered to animals upon return to their respective home cage after daily testing. Food pellets are commercially available at Bio-Serv in Flemington, New Jersey (0.5 g, Cat# F0171 and 1 g Cat# F0173).

### Ethics statement

Animals were bred and housed at the University of Western Ontario animal facility and were managed and treated according to the Canadian Council of Animal Care (CCAC) guidelines and approved Animal Use Protocols (2020-162, 2020-163).

### Transcriptomic analysis

Differential expression (DE) of chaperone transcripts was assessed in the Substantia Nigra (SN), Medial Temporal Gyrus (MTG), and amygdala of PD (*n* = 13,9,10) vs. healthy (*n* = 8,8,7) donors using Tukey HSD after two-way ANOVA (Transcript_levels ~ Brain_region * PD.or.Ctrl) with False Discovery Rate (FDR) correction. Samples were obtained from the Netherlands Brain Bank (NBB). Tissue samples from PD patients and control donors taken post-mortem were received from the NBB after submission of a thoroughly justified application. The NBB obtained a written consent for brain autopsy (for research purposes) from donors from which tissues were collected. Correlation between chaperone and α-synuclein transcripts (Pearson followed by FDR correction) was assessed using the GTEx database (Genotype-Tissue Expression Project, extracted from the GTEx portal on 05.04.20) consisting of gene expression from previously healthy post-mortem donors. Analyzed data included four brain regions—putamen (*n* = 124), cortex (*n* = 158), substantia nigra (*n* = 88), and amygdala (*n* = 100). In each region, samples were divided into aged (60–80 years) and young (20–59 years) men and women. See Supplementary Tables 1 & 2 for information on donor samples from PD patients and healthy donors, respectively.

### Human wild-type (WT) α-synuclein pre-formed fibril (PFF) generation and characterization

Human WT α-synuclein PFFs were generated as described previously [115, 122]. Briefly, wild-type α-synuclein tagged with GST in the pGEX-6P-1 plasmid was expressed in BL-21(DE3) *E. coli*. Protein was purified with Glutathione Sepharose® 4B resin (GE Healthcare), followed by cleavage of the GST tag with the GST-HRV 3C protease, and purification through a GSTrap 4B column (GE Healthcare). Chromogenic endotoxin quantification kit (Thermo Scientific) was used to assess whether the levels of endotoxin were < 1 EU/mg of protein. PFFs were prepared by shaking monomeric α-synuclein (ThermoMixer, Eppendorf) at 1000 rpm, 37 °C for 5 days. Next, PFFs were sonicated using a Bioruptor® Plus sonication unit (Diagenode). Sonicated PFFs were aliquoted to smaller 20–25 µl volumes prior to storage at − 80 °C. Both electron microscopy and dynamic light scattering were used for the characterization of α-synuclein monomers and PFFs, confirming that PFFs were successfully sonicated from batch to batch, and that the mean size profile was < 100 nm in diameter by dynamic light scattering (DLS). PFFs were stored at − 80 °C and thawed to room temperature prior to experimental use.

### Stereotaxic intracerebral injections of WT human α-synuclein PFFs

Intracerebral injections were performed as described previously [82]. Briefly, 3–4-month-old male mice were anesthetized with Isoflurane (Cat# CP0406V2, Fresenius Kabi) at 4% and O_2_ flow maintained at 0.8 L/min in a closed chamber, and once unconscious, animals were moved to the stereotaxic apparatus inside a biological safety cabinet and kept at 2.0–3.0% isoflurane throughout surgery, connected to a nose mask adaptor. Mice also received 5 mg/kg Metacam (Meloxicam, NADA 141-219) intraperitoneally after being transferred to the stereotaxic apparatus. Animals received a single injection of either 2.5 µl of PFFs (12.5 µg from 5 mg/ml PFFs) or 2.5 µl of sterile PBS in the dorsal neostriatum of the right hemisphere + 0.2 mm relative to bregma (AP), + 2.0 mm from the midline (LV). The Hamilton syringe was lowered to a depth of 3.0 mm and brought back up 0.4 mm to a final depth of 2.6 mm (DV). The injection material was infused at a flow rate of 250 nl/min, taking a total of 10 min. One-to-two minutes after finishing the injection, the Hamilton syringe was slowly withdrawn, and the incision sutured. Animals were allowed to recover and were tested up to 14–16 weeks post-injection (wpi). Paralysis or significant health decline was observed in the majority of mice at this time point.

### Tissue collection and processing

Tissue was collected either for whole brain histology or dissected and stored at − 80 °C for biochemistry. For histology, animals were anesthetized with a lethal overdose of ketamine (100 mg/kg) and xylazine (20 mg/kg) in 0.9% sterile saline. Once unconscious and non-responsive, animals were transcardially perfused with ice-cold 1X PBS followed by cold 4% paraformaldehyde (PFA) [70, 74, 75]. M83+/− , M83+/− ::ΔTPR1, M83+/− :TgA mutant PFF-injected mice whole brains were collected and stored in 4% PFA for 24 h before transferred to 15% sucrose in 1X PBS for 24 h, then 30% sucrose for an additional 48 h, to allow for visualization of α-synuclein pathology in both hemispheres post-PFF injection. Brain tissue was frozen in Cryomatrix embedding media (Cat# 67–690-06, Thermo Scientific: Shandon) on dry ice, and then stored at − 80 °C. Tissue was cut at 15–25 µm on Leica CM1950 Cryostat and sections were mounted directly onto SuperFrost Plus Slides (Fisher Scientific) left to air dry and then stored at − 80 °C or stored free-floating in 1X PBS + 0.02% azide (PBS-N) at 4 °C, until use. For biochemistry experiments, tissue was dissected on ice and flash frozen on dry ice before being stored at − 80 °C. Likewise, for M83+/+ :STI1-WT and for M83+/+ :ΔTPR1 mice, tissues used for biochemistry experiments were collected from mice cervically dislocated and rapidly dissected on ice, flash frozen, and stored at − 80 °C.

### Immunohistochemistry

For phospho-S129 α-synuclein (psyn129), GFAP, ubiquitin, or Hsp90 immunofluorescent labelling, frozen sections were brought to room temperature (RT) and washed twice with 1X TBS for 5 min each, followed by boiling in 10 mM Sodium Citrate + 0.02% Tween (pH 6.1) antigen retrieval buffer at 95 °C for 20 min. Slides were then cooled to RT in a bucket of ice, in the same buffer, for 30–40 min. Sections were washed once with 1X TBS for 5 min, and then permeabilized in three washes of TBS + 0.2% Triton, 5 min each. Sections were then blocked for 1.5 h in 5% Donkey Serum, 2% Normal Goat serum in TBS, and 0.2% Triton at RT. Primary antibodies were diluted in the blocking buffer and sections were incubated for 16–18 h at 4 °C. Primary antibodies used were: anti-Human alpha Synuclein phospho (Ser129) (1:2000, Cat# ab51253, Abcam, RRID:AB_869973), Anti-Alpha-synuclein (phospho S129) antibody (1:2000 MJF-R13 ab168381, Abcam, RRID:AB_2728613), anti-GFAP (1:400, Cat# ab4674, Abcam, RRID:AB_304558), anti-ubiquitin (1:50, Cat# ab7254, Abcam, RRID:AB_305802), anti-Hsp90 (1:50, Cat# 4877, Cell Signaling Technology, RRID:AB_2233307), and anti-Hsp90 (1:50, ab59459, Abcam, RRID:AB_942030). After primary antibodies were removed, sections were washed 3 times with TBS + 0.2% Triton and then incubated with secondary antibodies: donkey anti-rabbit Alexa Fluor 647 (1:500, Cat#A-31573, ThermoFisher, RRID:AB_2536183), goat anti-mouse Alexa Fluor 488 (1:500, Cat# A-11001, ThermoFisher, RRID:AB_2534069), goat anti-chicken Alexa Fluor 488 (1:500, Cat# A-11039, ThermoFisher, RRID:AB_2534015), or donkey anti-mouse Alexa Fluor 546 (1:500, Catalog # A-10036, ThermoFisher RRID:AB_2534012) for 2 h at RT. After 3 washes, sections were stained with Hoechst 33342 (1:1000, Cat#62249, ThermoFisher) for 10 min, rinsed twice with TBS, and then, autofluorescence was quenched using TrueBlack Lipofuscin Autofluorescence Quencher (Cat# 23007), following the manufacturer’s instructions. Immunofluorescent labelling for STI1 (1:400, Bethyl Laboratories) or STI1 (1:300, H00010963-M35, RRID:AB_10718570) was performed as previously described [74]. As described above, cryosections were brought to room temperature from − 80 °C and subjected to sodium citrate antigen retrieval. After TBS washes, sections were blocked in 2% horse serum, 2% normal goat serum, 1% BSA, and 0.3% Triton-X-100 in TBS for 1.5 h at room temperature. STI1 (1:300, H00010963-M35, RRID:AB_10718570) was co-labelled with anti-psyn129 (1:1000, Cat# ab184674, Abcam, RRID:AB_2819037) or Anti-Alpha-synuclein (phospho S129) antibody (1:5000, MJF-R13 ab168381, Abcam, RRID:AB_2728613), and sections were put in blocking buffer with appropriate antibody concentrations as described above and incubated overnight at 4 °C. After 3 washes in TBS, sections were incubated with secondary antibodies. To reduce background autofluorescence, a goat anti-rabbit Alexa 633 (1:500, Cat# A-21063, RRID:AB_2535727) was used for STI1. For psyn129 rabbit antibodies, a goat anti-rabbit Alexa 488 antibody (1:500, Cat# A-11034, AB_2576217) was used. Autofluorescence was quenched as described above. Whole-section images were captured using EVOS Thermo Auto FL 2 (ThermoFisher) 20X objective (N.A. 0.4), or Leica DM6B Thunder Imager 20X (N.A. 0.8).

Colocalization images were captured using either Leica TCS SP8 confocal microscope using a 40X (N.A. 1.3) and 63X (N.A. 1.4) or Leica DM6B Thunder Imager with a 40X (N.A. 0.95) and 63X (N.A. 1.4) objectives. For qualitative imaging, at least 3 sections obtained from 2–3 mice per genotype were imaged. During initial fluorescence examination, experimenter compared relative signals to ensure that fluorescence signal saturation would not occur in the parameters used. Once appropriate parameters were established, the same settings were used for each genotype and the experimenter was blinded to genotype to acquire images used in the manuscript. Percent area quantification was performed using Fiji ImageJ (NIH) by separating channels, converting images to 8 bit, then setting an image threshold and using “Measure > Area Fraction function”. Brightness and contrast and threshold settings for each channel were identical between sections and genotypes and the experimenter was blind to conditions and genotype during analyses. For experiments in which we provide quantitative measures, 3–6 sections per mouse were obtained from 5–6 mice per genotype and the procedure used to blind the experimenter was the same.

## Co-staining of Amytracker and phosphorylated alpha-synuclein antibody

For co-staining of psyn129 and Amytracker, a fluorescent dye that binds specifically to the β-sheet structure of amyloid-like protein aggregates [86], the manufacturer protocol was followed (Ebba Biotech) with some modifications. At first, brain sections were mounted onto Superfrost Plus slides. Then, sections were washed with 1X TBS and permeabilized by washing them 3 times in TBS + 0.2% Triton, 5 min each. Sections were incubated with 5% donkey serum, 2% normal goat serum in TBS + 0.2% Triton at room temperature for 1 h, and then were processed for immunostaining by overnight incubation at 4 °C in the primary antibodies of anti-Human alpha-synuclein phospho (Ser129) (1:2000, Cat# ab51253, Abcam, RRID: AB_869973) diluted in the blocking buffer. After rinsing three times with TBS + 0.2% Triton, sections were incubated with the secondary goat anti-rabbit Alexa 488 antibody (1:500, Cat# A-11034, AB_2576217) for 2 h at room temperature. Brain sections were washed with TBS three times, and then stained with Hoechst 33342 (1:1000, Cat#62249, ThermoFisher) for 10 min, followed by rinsing with TBS 2 times. The sections were washed for 5 min with PBS and then incubated with the Amytracker tracer (1:1000, Cat# AmytrackerTM630, Ebba Biotech) for 30 min. Finally, the sections were washed 2 times with PBS, 5 min each. Whole section and colocalization images were captured using Leica DM6B Thunder Imager with 63X (N.A. 1.4) objective.

### Western blotting

Immunoblotting was performed as described previously [12, 74, 75, 92]. For biochemical analyses, tissue was collected as described above. For M83+/+ :ΔTPR1 animals, only one hemisphere was dissected after transcardial perfusion with ice-cold PBS. For M83+/− :TgA and M83 +/− ::ΔTPR1 mutant mice, hemispheres were separated to allow for distinguishing of injected (ipsilateral) vs non-injected (contralateral) hemisphere from PFF-injected and PBS-injected mice. To obtain whole-cell lysates, tissue was homogenized in ice-cold RIPA buffer (50 mM Tris pH 8.0, 150 mM NaCl, 5 mM EDTA, 0.1% SDS, 0.5% Sodium Deoxycholate, 1% Triton-X 100) with phosphatase inhibitors (1 mM NaF and 0.1 mM Na_3_VO_4_) and protease inhibitor cocktail (1:100, Catalog#539,134-1SET, Calbiochem), further lysed by suction through 1 cc syringe, and then sonicated at 4 °C with 3 pulses for 7 s each. Tissue was left to rock at 4 °C for 20 min, and then centrifuged for 20 min at 10,000 × g. Supernatant was collected as RIPA soluble fraction. The RIPA-insoluble pellet was then resuspended in 2% SDS and 4 M Urea, known to solubilize α-synuclein oligomers and aggregates [126], sonicated and then centrifuged for 20 min at 13,000 × g to isolate SDS/Urea-soluble fraction (which constitutes insoluble protein, using modified protocols [107]). Protein concentration was determined by ThermoFisher BCA protein assay (Cat# 23227) and 10–30 µg of protein was loaded onto 4–12% Bis–Tris Gradient gels (ThermoFisher) prior to protein transfer onto 0.2 µm PVDF membranes, (ThermoFisher). Membranes were fixed in 0.4% PFA for 30 min at RT and then were blocked with 5% milk in TBS-T (0.1% Tween) for 1 h at RT. For SDS/Urea blots, membranes were stained with 0.1% Amido Black, rinsed with water, and imaged prior to blocking, to serve as total protein loading control. Membranes were incubated with primary antibodies against anti-Human alpha Synuclein phospho (Ser129) (1:1000, Cat# ab51253, Abcam, RRID:AB_869973), Anti-Alpha-synuclein (phospho S129) antibody (1:1000, MJF-R13 ab168381, Abcam, RRID:AB_2728613), Anti-Human alpha Synuclein Monoclonal Antibody, Clone LB 509 (1:1000, Cat# ab27766, Abcam, RRID:AB_727020), anti-STI1 (1:5000, in-house antibody generated by Bethyl Laboratories), anti-Hsp70 (1:750, Cat# ab2787, Abcam, RRID:AB_303300), and anti-Hsp90 (1:1000, Cat# 4874, Cell Signaling Technology, RRID:AB_2121214). Anti-actin HRP was used as protein loading control (1:25,000, Cat#A3854, Sigma-Aldrich, RRID:AB_262011) and secondary antibodies were sheep anti-mouse HRP (1:5000, Cat#SAB3701095, Sigma-Aldrich, RRID: N/A), and goat anti-rabbit HRP (1:10,000, Cat#170–6515, BioRad, RRID:AB_11125142). Protein was visualized using chemiluminescence detection technique and imaged using ChemiDoc MP Imaging System (BioRad), and densitometry was analyzed using BioRad ImageLab software. Western blots were repeated at least 2–3 times with 4–5 animals per genotype usually run in a gel.

### Co-immunoprecipitation

Cortical brain tissue was collected from cervically dislocated adult mice and dissected on ice before stored at − 80 °C until use for immunoprecipitation of endogenous STI1. 6–10-month-old WT mice on the C57BL/6 background [75] and 10–11-month-old non-transgenic WT and M83+/+ on a C57BL/C3H background were used for experiments. Both cortices per mouse were homogenized on ice in 50 mM Tris pH 7.4, 150 mM NaCl, 1 mM EDTA, and 1% Triton lysis buffer with phosphatase inhibitors (1 mM NaF and 0.1 mM Na_3_VO_4_) and protease inhibitor cocktail (1:100, Catalog#539,134-1SET, Calbiochem), rocked for 25–30 min at 4 °C, and then centrifuged for 20 min at 12,000 rpm. 5 µg of protein (~ 0.1%) from each sample was taken and used to make input samples, which were resuspended in 2X SDS loading dye buffer and boiled at 95 °C for 10 min, then frozen at − 20 °C. The supernatant above was collected and protein concentration was determined using ThermoFisher 660 nm protein assay kit (Cat# 22662), with 4 mg of protein being used for immunoprecipitation. Protein lysates for immunoprecipitation were pre-cleared using Protein G Dynabeads (Cat # 10004D, Invitrogen) by rotation at 4 °C for 30 min, and then incubated overnight with either 5 µg of Normal Rabbit IgG antibody (Cat# 12–370, Millipore, RRID:AB_145841) or 5 µg of STI1 antibody (in-house antibody generated by Bethyl Laboratories), rotating at 4 °C. The following day, lysates were incubated with Protein G Dynabeads (Cat# 10004D, Invitrogen) for 1 h at 4 °C with rotation. Samples were then washed three times with lysis buffer (50 mM Tris pH 7.4, 150 mM NaCl, 1 mM EDTA, and 1% Triton lysis buffer with phosphatase inhibitors) and resuspended in 2X SDS loading buffer and boiled at 95 °C for 10 min. Input samples that were frozen overnight were also boiled again for 10 min at 95 °C and samples were loaded on 4–12% Bis–Tris Gradient gels (ThermoFisher) and blotting procedure was performed as described above. Experiments were repeated three times using the conditions described above, with different mice being used for each experiment (biological replicates). To obtain 4 mg of tissue, at least 2 mice per genotype were used per IP reaction, and lysed tissue of the same genotype was combined for each experiment (equating to 6 mice/condition). Antibodies used were: anti-Mouse α-synuclein (1:1000, Cat# 610787, BD Biosciences, RRID:AB_398108), anti-Human alpha Synuclein phospho (Ser129) (1:1000, Cat# ab51253, Abcam, RRID:AB_869973), Anti-Human alpha Synuclein Monoclonal Antibody, Clone LB509 (1:1000, Cat# ab27766, Abcam, RRID:AB_727020), anti-STI1 (1:5000, in-house antibody generated by Bethyl Laboratories), and anti-Hsp90 (Cat# 4874, Cell Signaling Technology, RRID:AB_2121214).

### Recombinant protein expression and purification

Uniformly ^15^ N-labelled human α-synuclein was expressed in *E. coli* BL21 (DE3) cells grown in minimal M9 medium containing ^15^NH_4_Cl (1 g/L) as the sole nitrogen source. When the OD600 reached 0.8, 0.5 mM of IPTG was added for induction. The cultures were incubated for 4 h at 37 °C before they were harvested by centrifugation. Pellets were washed with PBS and then stored at − 20 °C.

Bacterial pellets from 1 L of culture were resuspended in 25 mL of cold osmotic shock buffer (30 mM Tris–HCl pH 7.2 + 40% Sucrose (w/v) + 2 mM EDTA) and incubated at room temperature for 10 min. Suspensions were briefly centrifuged for 5 min at 13,000 × *g* at 4 °C and the supernatant was discarded. The cell pellets were resuspended in 25 mL cold water (4 °C) containing 20 µL of saturated MgCl_2_ solution and briefly incubated on ice for 3 min. To remove bacterial spheroplasts, samples were centrifuged at 16,000 × *g* for 10 min at 4 °C. Supernatant was then subjected to a boiling water bath (100 °C) for 20 min followed by chilling on NaCl-ice at − 20 °C for 20 min. The heat shocked protein samples were clarified by centrifugation at 16,000 × *g* for 15 min at 4 °C. Supernatant was filtered through a 0.8 µm filter and ammonium sulfate was slowly added to a final concentration of 60%. The ammonium sulfate precipitation was stirred for 1.5 h at 4 °C. Precipitated protein was recovered by centrifugation at 16,000 × *g* for 30 min at 4 °C, dissolved in a 20 mL volume of dialysis buffer I (25 mM Tris–HCl at pH 8.0 + 1 mM EDTA), loaded into 3.5 K MWCO dialysis tubing, and dialyzed overnight at 4 °C. Following several exchanges of dialysis buffer, the sample was filtered through 0.8 µm filter units and protein concentration was estimated by Lowry assay. For NMR experiments, the protein was exchanged several times by dialysis into 20 mM HEPES + 50 mM NaCl at pH 7.2 overnight at 4 °C. The final α-synuclein protein was 0.20 µm filtered, and concentrated with a 5 K MWCO Turbo VivaSpin15 concentrator to 500 µM. Samples of the purification were analyzed by 10% Tris-Tricine PAGE. A typical yield from 1 L of growth is 22–24 mg of α-synuclein.

pDEST17 (Invitrogen) expression plasmids encoding full-length mouse STI1, TPR1 domain (residues 1–118), TPR2A domain (residues 217–352), and TPR2B domain (residues 353–480), all fused with N-terminal tobacco etch virus (TEV) cleavable 6xHis tag, were transformed into *E. coli* BL21 (DE3) pLysS for the recombinant protein expression. Expression and purification of recombinant full-length mouse STI1 and the three TPR domains were performed as previously described [85].

### Phosphorylation of recombinant α-synuclein at S129 using PLK3

Approximately 5.2 mg of purified α-synuclein was added to a 500 µL volume reaction containing 4.2 µg of Polo-like kinase 3 (PLK3) (ThermoFisher PV3812), 50 mM HEPES, 10 mM MgCl_2_, 1 mM EGTA, 1 mM DTT, and 2 mM ATP, and incubated overnight at 30 °C. Following incubation, the sample was exchanged by dialysis overnight using a 5 mL 500-1000Da MWCO dialysis cassette with 20 mM HEPES and 50 mM NaCl at pH 7.2. The protein was concentrated with a 5 K MWCO Vivaspin Turbo4 (Sartorius) to a volume of 1 mL and Lowry protein assay was used to estimate the concentration. ~ 95% of the phosphorylated α-synuclein was recovered.

For the S129 phosphorylation time-course experiments, 300 µg of purified α-synuclein was added to a 200 µL volume (105 µM) reaction containing 0.13 µg of PLK3 kinase, 50 mM HEPES, 10 mM MgCl_2_, 1 mM EGTA, 1 mM DTT, and 2 mM ATP, and incubated overnight at 30 °C. For the reaction with STI1 TPR1 or TPR2A domain included, an equal molar quantity (105 µM) of purified TPR1 or TPR2A was added to the reaction. Additionally, a reaction was done with TPR1 at a mg/mL concentration (1.7 mg/mL) that matched that of 105 µM TPR2A. All reactions were made in duplicate, and each replicate was incubated at 30 °C. At various time points (0, 10, 20), a 5 µL volume (~ 8 µg of α-synuclein) was removed from each reaction and prepared for SDS-PAGE by addition of 4X Laemmli buffer, heated for 4 min at 100 °C, and stored at − 20 °C until completion of the time-course. Samples were thawed, loaded onto SDS-PAGE, and transferred to a blotting membrane and probed with anti-S129 α-synuclein antibody (Cat# ab51253, Abcam, RRID:AB_869973) as described above. These experiments were repeated 2–3 times with independent generated protein samples.

### Solution NMR spectroscopy

NMR experiments were conducted at 25 °C on a 600 MHz Varian Inova spectrometer equipped with a cryogenic triple resonance HCN probe. The ^1^H-^15^ N HSQC spectra were collected using 128 × 537 complex points in the ^15^ N and ^1^H dimensions, respectively. The NMR data were processed using NMRPipe and analyzed using NMRViewJ software. Chemical shift perturbation analyses of the NMR titration series were performed using the Titration Analysis function in NMRViewJ and in-house Matlab scripts. The composite chemical shift changes of ^1^H-^15^ N peaks were calculated according to ∆δ_comp_ = [(∆δ_1H_)^2^ + (0.14*∆δ_15N_)^2^]^1/2^, where ∆δ_1H_ and ∆δ_15N_ are the changes in ^1^H and ^15^ N chemical shifts (in ppm) upon the addition of binding target. The binding affinities were determined based on the chemical shift perturbations and protein concentrations determined by amino acid analysis (SPARC BioCentre, The Hospital for Sick Children, Toronto), assuming fast exchange between the free and ligand-bound forms [127].

### Motor assessments for M83+/−, M83+/−:TgA and M83+/− ::ΔTPR1 mice

For M83+/−:TgA and M83+/− ::ΔTPR1 PFF and PBS-injected mice, motor assessments were performed at 14–16 wpi (the time-point when the majority of cohort began to significantly deteriorate and show slow movement and gait disturbance). The experimenter was blind to genotype during motor assessments, but not during data analyses.

#### Grip force

Grip force in the forelimbs was measured using Columbus Instruments Grip Strength Meter. Animals were lowered, allowing them to grip onto grip strength bar and were pulled horizontally by the tail until they let go, and placed in a large plexiglass bucket filled with bedding. The force exerted as the mouse let go of the bar was recorded in Newtons, with a total of 10 pulls per mouse being recorded. The largest value out of ten was used for analyses as described previously [65, 72, 98]. In analyses comparing the percentage of mice that exerted a grip force less than 1.0 N, each trial was considered and an average across each genotype and treatment condition was analyzed.

#### Wire hang

Wire hang was performed as described elsewhere [65, 72]. Mice were placed on a metal grid 45 cm above a large tub filled to the top with wood-chip bedding. Animals were suspended upside down on the wire grid for a total of 60 s for 3 trials, trials spaced at least 5 min apart. The average time to fall was recorded and used for analyses. In analyses comparing the percentage of mice that held on for less than 10 s, each trial was considered and an average across each genotype and treatment condition was assessed.

### Touchscreen behavioral testing

All behavioral tests were conducted during the light phase. Paired visual discrimination task with reversal (PVD-R) and the 5-Choice serial reaction time task (5-CSRTT) were used to evaluate reversal learning and attention in mice, respectively, as previously described [11, 72, 104]. Both tasks were conducted using the automated Bussey–Saksida touchscreen system for mice (model 80614; Lafayette Instrument, Lafayette, Indiana) and the data collected using ABET II Touch software Version 2.20 (Lafayette Instrument, Lafayette, Indiana). The software is used to run the task and record the behavioral activity of the mouse. Each mouse was scheduled for only one run at about the same time daily. All touchscreen data in this study were deposited into the Mousebytes database (www.mousebytes.ca [11]). The general procedure, including habituation and the pre-training program, for both PVD-R and 5-CSRTT in a touch-screen-based automated operant system for mice was described in detail elsewhere [11, 72].

#### 5-CSRTT

The 5-CSRTT was performed to measure attention in mice as previously described [13, 71]. A mask with five rectangular windows was placed in front of the touchscreen and mice were required to respond to a brief light stimulus pseudo-randomly presented in one of the five windows on the touchscreen. In each block of 20 trials, the stimulus was presented 4 times in each window. Illumination of the reward magazine signaled a head poke to initiate each trial. A 5–10 s delay interval followed, and a light stimulus was displayed in one of the windows. The mouse was required to respond to the stimulus within a period of up to 5 s (limited hold). The duration of stimulus presentation (or illumination of the window) was initially set to 4 s. The first response to a window, upon stimulus display, or within the limited hold period was recorded. Reward was delivered in the reward tray magazine when a correct response was made. The criteria for the 4 s stimulus duration training included at least 80% accuracy and 20% omission or less, and 30–50 trials must be completed on 2 out of 3 consecutive days. The 4 s stimulus duration training was followed by a 2 s stimulus duration training. Mice were trained on this task until they reached a criterion of stable performance at 80% accuracy with stimuli duration of 2 s. After reaching criteria, mice were subjected to probe trials to test for attentional deficits. Each mouse performed two sessions with 1.5, 1.0, 0.8, and 0.6 s stimulus duration (the order of the probe trials sessions were randomized for each counterbalanced group). The probe trial schedules were identical to the 4 s and 2 s schedules. Each intra-probe session consisted of two consecutive days of 2 s stimulus duration sessions. Attention was measured in M83+/+ mice (*n* = 10) and non-transgenic WT controls (*n* = 12) at 8 months of age.

#### PVD-R

In the acquisition phase, mice were required to choose between a rewarded (S+ , fan image) and unrewarded (S-, marble image) stimulus displayed in a mask with two windows placed in front of the touchscreen. The location of the S+ and S- stimuli was pseudo-randomly either at the left or right window and the same stimulus arrangement was not presented more than 3 times. When the mouse touched the S+ (correct), the stimuli were removed, and the strawberry milkshake reward was delivered along with illumination of the magazine light and a tone. An incorrect response (touching the S− image) was followed by a 5 s timeout with the house light on. After an incorrect response, the mouse initiated the correction trial by entering the magazine. Correction trials preserved the left/right arrangement of the S+/S− images from the incorrect trial until a correct choice was made. The results of correction trials do not contribute to the overall trial count or correct/incorrect responses. Each session ended once the mouse completed 30 trials or reached a 60-min timeout. The mouse was required to achieve at least 80% correct responses (24/30 trials correct) for 2 out of 3 consecutive days to reach acquisition criterion. Once all animals in a cohort had reached the acquisition criterion, they were put back on the task and received 2 further task sessions that served as baseline performance (B1 and B2). Baseline sessions were identical to the PVD acquisition and there were no criteria required. Following the baseline sessions, mice were tested on the reversal learning task for 30 trials per session, for 10 sessions. In the reversal phase, the S+ and S− contingencies were reversed, i.e., S+ was the marble image and the S− the fan image. Trial initiation and correction trials happened in the same fashion as during acquisition and there were no criteria required for the reversal phase. The session also ended either after completion of 30 trials or in 60 min. The measurements were compared between M83+/+ : STI1WT mice (*n* = 20) and littermate M83+/+ :ΔHET mice (*n* = 18) and WT controls (*n* = 20).

### MRI imaging

A subset of mice used for touchscreen testing was prepared for ex vivo MRI; WT control mice (*n* = 8), M83+/+ :STI1WT mice (*n* = 5) and M83+/+ :ΔHET mice (*n* = 8). These mice were perfused with 0.9% (wt/vol) phosphate-buffered saline with 0.4% ProHance (gadoteridol, a gadolinium-based MRI contrast agent and 0.1% heparin, an anticoagulant), followed by 4% paraformaldehyde with 0.1% heparin. To best acquire ex vivo images, the brains were kept in-skull and the surrounding tissue (facial muscles and eyeballs) was also kept, as this reduces the likelihood of scanner artifacts, specifically caused by air bubbles [25]. Prior to scanning, the brains were stored in sodium azide 0.1 M for about 3–4 weeks, until the brains were scanned.

The MRI acquisition was performed on 7.0-T Bruker Biospec (70/30 USR) 30-cm inner bore diameter; AVANCE electronics) at the Douglas Research Centre (Montreal, QC, Canada). High-resolution ex vivo T1-weighted images (FLASH; Fast Low Angle SHot) were acquired for each subject (TR = 21.5 ms, TE = 5.1 ms, 70 μm isotropic voxels, 2 averages, scan time = 32 min, matrix size = 258 × 228x130, flip angle = 20°).

All brain images were converted to NIfTI format (Neuroimaging Informatics Technology Initiative) from the native Bruker format, and then converted to the MINC (Medical Imaging NetCDF) file format for processing. Image processing was performed using the MINC suite of software tools (http://bic-mni.github.io). Next, the images were stripped of their native coordinate system, left–right flipped to compensate for Bruker’s native radiological coordinate system, denoised using patch-based adaptive non-local means algorithm [35], and affinely registered to an average mouse template (the Dorr–Steadman–Ullman atlas; [43, 114, 119, 120]) to produce a rough brain mask. Next, a bias field correction was performed, and intensity inhomogeneity was corrected using N4ITK [117] at a minimum distance of 5 mm.

The images were manually inspected for artifacts (hardware and software artifacts or tissue heterogeneity and foreign bodies) both prior to and after preprocessing, and images with said artifacts were excluded (*n* = 1) (https://github.com/CoBrALab/documentation/wiki/Mouse-QC-Manual-(Structural)).

The preprocessed MRI images were segmented into 355 structures per hemisphere (with 53 interhemispheric regions) using the Multiple Automatically Generated Templates (MAGeT) Brain segmentation algorithm [27], and a down-sampled version of the Allen Mouse Brain Atlas [78]. Using the Automatic Normalization Tools (ANTS) (https://github.com/vfonov/mincANTS), nonlinear registration techniques are implemented to first propagate the atlas segmentations onto a subset of the subject data, known as the template library. From there, the segmentations from the template library are then propagated to each subject image yielding multiple segmentations per subject, which are then fused using majority-vote label fusion to generate a final segmentation. From these final segmentations, volumes of brain structures were computed and analyzed for group differences. A full list of the anatomical regions segmented in the modified Allen brain atlas is described in Supplementary Table 3.

Prior to performing MRI analysis, quality control of the segmentations was performed by visual inspection of overlaying the final segmentations atop each subject image to ensure the labels matched the underlying anatomy [54, 60, 103].

Statistical analyses were carried out using R software (3.5.0) and the RMINC package (https://wiki.phenogenomics.ca/display/MICePub/RMINC). The analysis was performed using linear models to examine group differences at each region using the MAGeT-Brain volume outputs, specifically to examine group differences between WT control mice, M83+/+ : STI1WT, and M83+/+ :ΔHET mice. All results were corrected for multiple comparisons using the FDR [8]; specifically with a 5% FDR threshold. Data are available in the following repository https://doi.org/10.5281/zenodo.6620797.

### Statistical analyses

Data were analyzed using GraphPad Prism v8 software. Two group comparisons were analyzed using unpaired two-tailed t test. Whereas analyses comparing three groups were analyzed using one-way ANOVA with appropriate post hoc comparisons, and multivariate two group comparisons were analyzed using two-way ANOVA with appropriate post hoc comparisons when required. Normality for behavioral data was evaluated using Shapiro–Wilk test and non-normal data were analyzed using Kruskal–Wallis test (non-parametric One-Way ANOVA) or non-parametric *t* test Mann–Whitney test. For behavioral analyses, effect size was calculated using open-access One-way ANOVA effect size calculator: https://webpower.psychstat.org/models/means03/effectsize.php. Full statistical analyses details for each analysis and relevant post hoc comparisons are reported in the Figure Legends (*t*, *F*, *df*, *N*, *p*). For the 5-CSRTT, accuracy, omission, reward collection latency, correct touch latency, premature responses, number of trials completed, and perseverative response were recorded and analyzed using repeated-measures two-way ANOVA. For the PVD-R task, the parameters number of sessions to reach acquisition criteria (analyzed by one-way ANOVA adjusted for multiple comparisons with Tukey's multiple comparisons test), the percentage of accuracy, number of correction trials, correct touch latency (s), reward collection latency and trials completed (analyzed by repeated-measures two-way ANOVA adjusted for multiple comparisons with Tukey's multiple comparisons test) were recorded. Data are presented as mean ± SEM and (*) denotes *p* < 0.05, (**) denotes *p* < 0.01, (***) denotes *p* < 0.001. For co-immunoprecipitation experiments, individual experiments were repeated three times, with the cortices of two different animals constituting each IP condition (*n* = 6) different animal tissue lysates being used in each technical replicate.

## Results

### STI1 interacts with α-synuclein

**Fig. 1:**
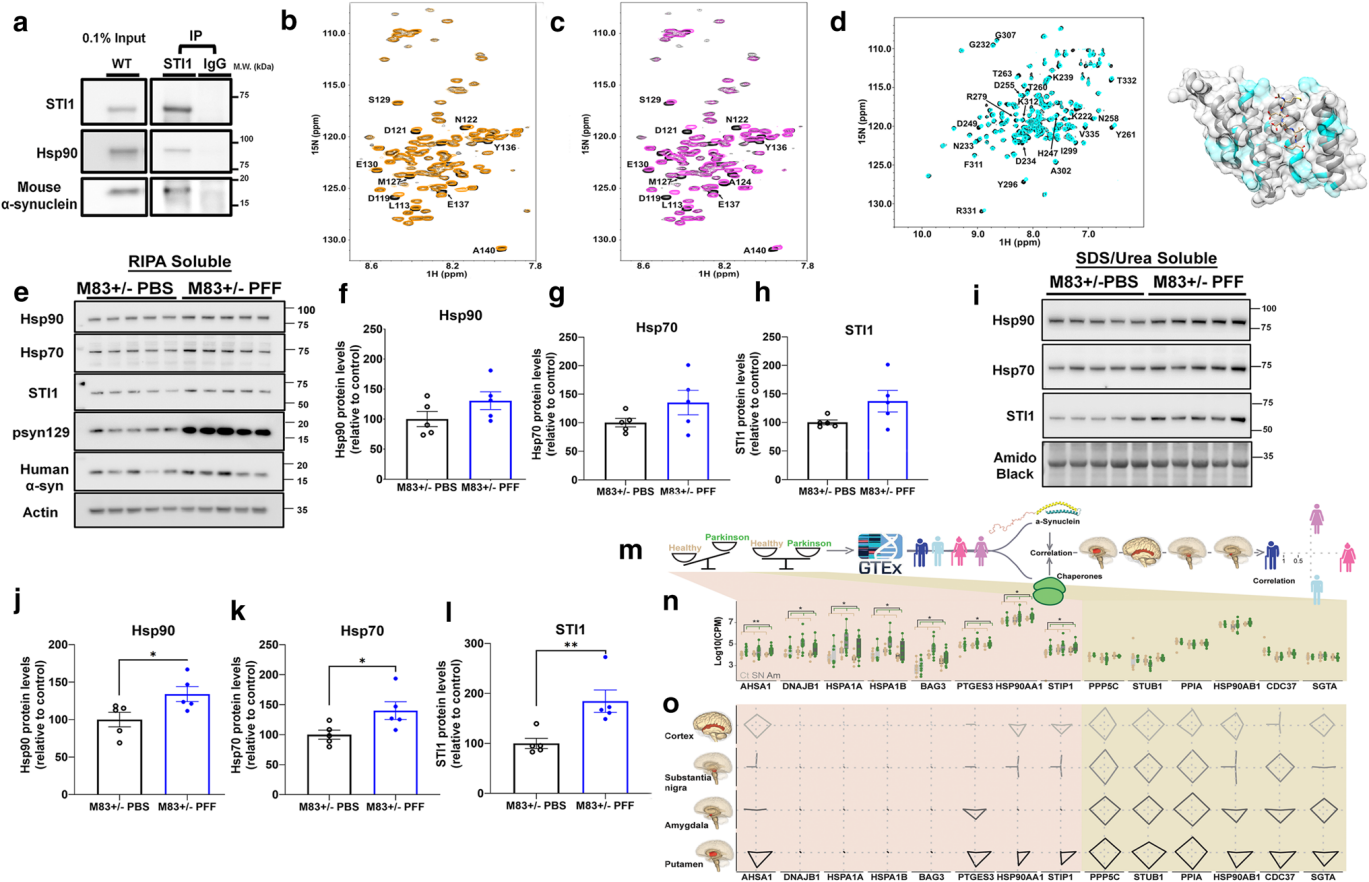
Interaction of α-synuclein and STI1, abnormal chaperone aggregation, and relationship between α-synuclein and chaperones **a** Co-immunoprecipitation experiments in 6–10-month-old wild-type mice. STI1 was immunoprecipitated using anti-STI1 antibody (5 µg, Bethyl Laboratories) and control precipitation was done using anti-Rabbit IgG antibody (5 µg). Representative co-immunoprecipitation of STI1 with pan-Hsp90 and mouse α-synuclein is shown. **b** 600 MHz ^1^H-^15^ N HSQC spectrum of 100 µM α-synuclein (in 20 mM Hepes, 50 mM NaCl, pH 7.2) in the absence (black) and the presence (orange) of 200 µM of STI1. **c** 600 MHz ^1^H-^15^ N HSQC spectrum of 100 µM α-synuclein (in 20 mM Hepes, 50 mM NaCl, pH 7.2) in the absence (black) and the presence (magenta) of 200 µM of STI1 TPR2A domain. **d** Left*:* 600 MHz ^1^H-^15^ N HSQC spectrum of 150 µM STI1 TPR2A (in 20 mM Hepes, 50 mM NaCl, pH 7.2) in the absence (black) and presence (cyan) of 150 µM of unlabelled α-synuclein. Labelled are the residues that show chemical shift changes upon binding. The peaks were assigned based on the literature [42, 46, 97]. Right*:* The structure of human STI1 TPR2A in complex with the Hsp90 pentapeptide (PDB: 1ELR; TPR2A is shown in ribbon and Hsp90 peptide is shown in sticks). Colored in cyan are residues labelled in the spectrum on the left. **e** Cortical lysates from M83+/− PBS and M83+/− PFF-injected mice were isolated in RIPA buffer. Representative immunoblot of Hsp90, Hsp70, STI1, psyn129, and human α-synuclein protein levels. **f–h** Densitometric quantification of **e**. **f** Hsp90 (*t*(8) = 1.57, *p* = 0.15), **g** Hsp70 (*t*(8) = 1.60, *p* = 0.16), and **h** STI1 (*t*(8) = 1.92, *p* = 0.09) relative to actin loading control. **i** immunoblotting for Hsp90, Hsp70, and STI1 in 2% SDS + 4 M urea cortical tissue lysates (same animals as used in **e–h**). **j–l** Densitometric quantification of **i**. **j** Hsp90 (*t*(8) = 2.43, *p* = 0.041), **k** Hsp70 (*t*(8) = 2.42, *p* = 0.041), and **l** STI1 (*t*(8) = 3.44, *p* = 0.009) relative to Amido Black stain loading control. Data represented as mean ± SEM and analyzed using unpaired *t* test—where *t* (8) refers to the degrees of freedom. Co-immunoprecipitation experiments (**a**) were reproduced three times with different animals (biological replicates). Within the α-synuclein rows, images that were cropped separately are from the same immunoblot, at different exposure times (8 s for input and 60 s for IP α-synuclein). **m** Chaperone transcript levels were upregulated (first half, *AHSA1-STIP1*) or unchanged (*PPP5C-SGTA*) in PD [Netherlands Brain Bank (NBB) dataset] in the medial temporal gyrus (Ct; *n* = 17), substantia nigra (SN; *n* = 21) and amygdala (Am; *n* = 17), and correlation of their levels with α-synuclein was sought separately in young and aged men and women of the GTEx cohort, in the cortex (*n* = 158), substantia nigra (*n* = 88), amygdala (*n* = 100), and putamen (*n* = 124). **n** Log10 of counts per million of the different chaperones in the NBB dataset. Tan boxplots show control patients and green boxplots show PD patients. For each chaperone transcript, boxplot pairs are shown for the medial temporal gyrus (Ct), substantia nigra (SN), and amygdala (Am). All the chaperone transcripts between *AHSA1 and STIP1* were significantly elevated in PD patients throughout the three brain regions checked (AHSA1 *p* < 0.01, DNAJB1 *p* < 0.03, HSPA1A *p* < 0.03, HSPA1B *p* < 0.03, BAG3 *p* < 0.03, PTGES3 *p* < 0.03, HSP90AA1 *p* < 0.03, STIP1 *p* < 0.03; ANOVA, FDR) **o.** Each grid represents the correlation for the corresponding chaperone (column) in the noted brain region (row). The upper and lower parts of the Y-axis indicate correlation in young women and men, respectively, and the left and right sides of the *X* axes, aged men and women. Only significant correlations are shown (*p* < 0.05, Pearson, FDR). See Supplementary Table 4 for full statistics

In vitro evidence suggests that most α-synuclein in cells form complexes with chaperones, including Hsp70 and Hsp90, and interactome analyses immunoprecipitating α-synuclein indicate that STI1 may also be an interactor of α-synuclein [21]. We tested whether STI1 and α-synuclein form a protein complex in mouse brain cortical tissue, and we found that precipitation of STI1 also specifically co-immunoprecipitated mouse α-synuclein, as well as Hsp90 (Fig. 1a). To test the possibility of a direct interaction between STI1 and α-synuclein, we performed NMR ^1^H-^15^ N HSQC experiments on ^15^ N-labelled recombinant human α-synuclein in the absence and presence of STI1. Upon adding recombinant mouse STI1 protein to a solution of ^15^ N-labelled human α-synuclein, we observed noticeable chemical shift perturbations in the C-terminal region of α-synuclein (aa 110-140, Fig. 1b), suggesting that STI1 can directly interact with the α-synuclein carboxy terminal tail. Significant chemical shift perturbations were observed for residues D119, D121, N122, S129, E137, and A140. Quantitative analysis of the NMR titration by addition of increasing STI1 concentrations to ^15^ N-labelled α-synuclein reveals that the chemical shifts of these residues change as a function of STI1 concentrations, indicating that α-synuclein undergoes fast exchange between the free and STI1-bound forms (Supp. Fig. S1c, online resource). The apparent K_D_ derived from the chemical shift perturbations is 176 ± 15 µM.

STI1 contains three tetratricopeptide repeat (TPR) domains that are modular protein–protein interaction sites. Therefore, we repeated the NMR experiment with each of these individual domains separately to investigate their potential interaction with α-synuclein (Supp Figs. S1a&b, online resource, Fig. 1c). TPR1 and TPR2B are known to interact with Hsp70, whereas TPR2A interacts with Hsp90, facilitating the inhibition of its ATPase activity and recruiting/holding clients to Hsp90 [37, 49, 110, 125]. We found that adding STI1 TPR2A domain reproduced the exact same chemical shift perturbation patterns in α-synuclein as full-length STI1 (Fig. 1c). NMR titration analyses indicated that TPR2A has a moderately increased affinity for α-synuclein compared to full-length STI1 (K_D_ of 79 ± 5 µM, Supp Fig. S1d, online resource). In contrast, neither TPR1 nor TPR2B induced any significant chemical shift changes on α-synuclein (Supp Figs. S1a&b). We also investigated whether α-synuclein can trigger chemical shift changes in the STI1 TPR2A domain. Upon addition of an equimolar concentration of α-synuclein to ^15^ N-labelled STI1 TPR2A, noticeable chemical shift changes were observed for a significant number of residues, including K239, D249, T260, Y261, T263, Y296, and R331 to name a few. Remarkably, many of these amino acid residues in TPR2A that show chemical shift perturbations are adjacent to the regions interacting with Hsp90 (Fig. 1d) [109]. Given the proximity of binding sites, we tested whether α-synuclein and Hsp90 compete for STI1. A pentapeptide motif (MEEVD) encoding the C-terminus of Hsp90, which has been shown to bind STI1 TPR2A with a K_D_ of ~ 11 µM [109], was used to gauge the effect of Hsp90 on the binding of TPR2A to α-synuclein. In the presence of an equimolar concentration of unlabelled TPR2A, chemical shift changes were observed for specific residues in the C-terminal region of α-synuclein (Supp Fig. S1e, online resource), as we observed in Fig. 1c. Upon the addition of an equimolar concentration of the Hsp90 peptide to the α-synuclein–TPR2A mixture, the chemical shift perturbations of α-synuclein were reduced. These experiments demonstrate that Hsp90 can effectively compete with α-synuclein for the STI1 TPR2A domain (Supp Fig. S1e, online resource). Together, these results indicate that STI1 and α-synuclein interact directly and form a complex in the mammalian brain. The complex interactions between STI1, Hsp90, and α-synuclein suggest that STI1 may act as an intermediate to facilitate α-synuclein delivery to Hsp90, or that STI1 may have direct effects on α-synuclein, or both. STI1 has been previously shown to interact with Hsp90 clients to structurally reorientate them prior to client transfer to Hsp90 [125]; therefore, these results suggest STI1 might be acting in a similar manner on α-synuclein.

### Elevated levels of insoluble STI1, Hsp90, and Hsp70 after α-synuclein aggregation

Numerous chaperones and co-chaperones, including Hsp90 and Hsp70, co-localize with Lewy bodies and in the brains of PD and DLB patients, Hsc70 and Hsp90 protein levels are increased in insoluble tissue fractions and correlate with insoluble α-synuclein deposition [121]. To test the possibility that STI1 associates with α-synuclein inclusions, we used M83 hemizygous mice (M83+/−) unilaterally injected with pre-formed human wild-type α-synuclein fibrils (PFFs) into the right dorsal striatum [82]. M83 mice express human A53T α-synuclein under control of the mouse prion protein promoter and are widely used to follow prion-like propagation of misfolded α-synuclein upon PFF injections. After PFF injection, the host human and mouse α-synuclein misfold, spread [83], and deposit in inclusions several weeks after PFF injection [31, 62, 82, 100]. During this process, α-synuclein becomes phosphorylated at S129 (psyn129), which can be used to identify its pathological spreading.

We characterized the PFFs used for injection by dynamic light scattering (DLS) and electron microscopy, confirming that the sonicated fibrils were between 5 and 100 nm in diameter (Supp. Figs. S2a&b). M83+/− mice injected with PFFs showed robust psyn129 labelling throughout the brain in both hemispheres, whereas PBS-injected M83+/− mice had no labelling for psyn129 (Supp. Fig. S2c). PFF-injected mice became symptomatic between 12 and 16 weeks post-injection, presenting decreased mobility and grooming, as well as weight loss, at which point they were euthanized. Immunocytochemistry experiments show that ubiquitin and Hsp90 partially co-localize with psyn129 (Supp. Fig. S2d&e) in PFF-injected mice, confirming previous results that these proteins are deposited in filamentous inclusions [82, 121]. Notably, STI1 also partially co-localizes with psyn129 (Supp. Fig. S2f). Moreover, we labelled brain sections in M83+/− PBS-injected mice (Supp. Fig. S2g) or PFF-injected (Supp. Fig. S2h) with Amytracker, a fluorescent dye used to label misfolded proteins including α-synuclein [52, 86], and found partial overlap between psy129 immunolabelling and Amytracker staining, indicating the pS129 antibody does indeed label misfolded inclusions. These results further corroborate the findings of others that chaperones and their co-chaperones are sequestered into α-synuclein inclusions [19, 79, 90, 121].

To evaluate how spreading of misfolded α-synuclein affects the chaperome, we compared STI1, Hsp90, and Hsp70 presence in detergent-soluble or insoluble fractions in PFF-injected mice and PBS-injected mice, using immunoblots. In RIPA-soluble fractions, psyn129 is increased in PFF-injected mice, and the levels of STI1, Hsp90, and Hsp70 did not change significantly when compared to PBS-injected M83 mice (Figs. 1e–h). In contrast, in the insoluble fraction (SDS/Urea solubilization of RIPA-insoluble pellets), the levels of STI1, Hsp90, and Hsp70 were augmented (Figs. 1i–l), suggesting that the molecular chaperone machinery shows increased aggregation or is more sequestered in insoluble fractions in PFF-injected mice, potentially diminishing overall chaperome activity. Indeed, when we compared WT control mice and M83+/− mice not injected with PFFs we did not find accumulation of Hsp90, Hsp70, or STI1 in soluble or insoluble fractions, further suggesting that this accumulation is triggered by the injection of PFFs (Supp. Fig. S3a-h).

The above experiments and previous work [21, 86, 121] indicate a relationship between the Hsp90/Hsp70/STI1 machinery and α-synuclein inclusions and toxicity in mice. To test for potential relevance in humans, we measured the levels of different chaperone-encoding transcripts in the Substantia nigra (SN), medial temporal gyrus (Ct), and amygdala (Am) of PD patients and matched controls from RNA-Seq datasets obtained using brain samples from the Netherlands Brain Bank [61] (Fig. 1m, n). Intriguingly, *STIP1* levels were elevated in all of these brain regions from PD patients, along with seven other chaperones and co-chaperones (*AHSA1, DNAJB1, HSPA1A, HSPA1B, BAG3, PTGES3, HSP90AA1,* Fig. 1 m, n), most of which interact with and regulate the Hsp70/90 machinery. However, other chaperone transcripts remained unchanged. To further examine this relationship, we tested the correlation of the different chaperone transcript subgroups with that of α-synuclein mRNA (*SNCA*) in four brain regions of young and aged (20 < 59, 60 > 80) men and women donors of the GTEx dataset (Fig. 1o, see Supplementary Table 1 for samples and conditions for PD patients, and Supplementary Table 2 for healthy donors from GTEx dataset, online resource). Most of the chaperones that were differentially expressed in PD brains showed null correlation with α-synuclein in all the analyzed brain regions. In contrast, most of the chaperones that were unchanged in PD showed significant correlations with α-synuclein in at least one of the tested brain regions (Fig. 1o). Exceptions included *STIP1, HSP90AA1, PTGES3,* and *AHSA1,* all of which showed both elevation in PD patient brains and correlation with α-synuclein in healthy control brains (Fig. 1o). Furthermore, the correlation patterns of these transcripts were highly similar, especially for *STIP1* and *HSP90AA1* which presented exactly the same pattern, suggesting co-regulation of these transcripts. Finally, the correlation of *STIP1* with α-synuclein transcripts was the most significant in the putamen and cortices of young men (*p* < 4e−5, *p* < 3e−4, Pearson False Discovery rate—FDR 5%, See Supplementary Table 4 for full statistics). These results propose that that two of the regions significantly affected in synucleinopathies could be impacted by levels of STI1 and its regulation of the chaperome [41, 108].

### Elevated Hsp90-STI1 levels facilitate prion-like propagation and deposition of insoluble α-synuclein in mice injected with PFFs

**Fig. 2:**
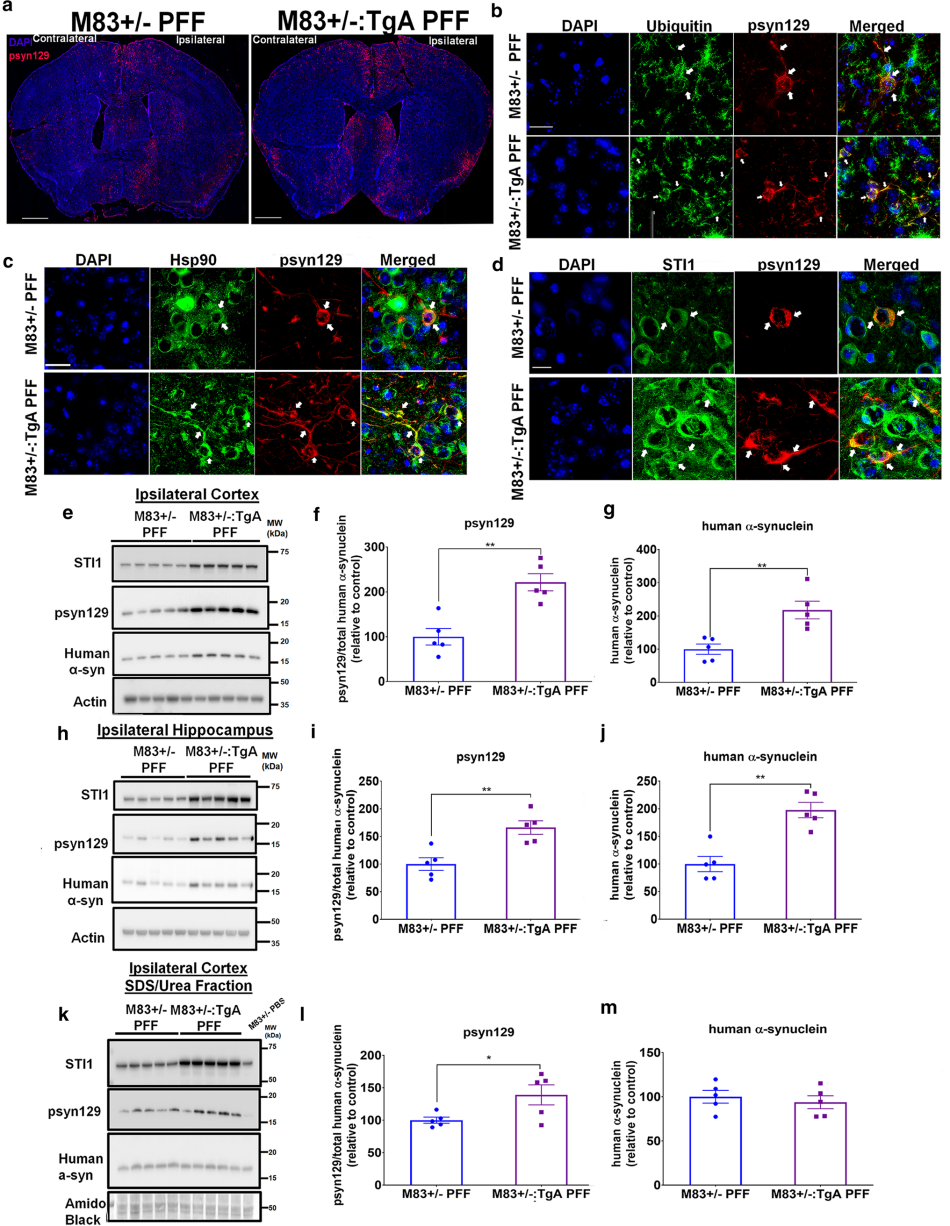
Overexpression of STI1 and Hsp90ß exacerbates pS129 labelled pathology. **a** Representative images of psyn129 (red) immunolabelling with nuclear marker (blue) in coronal sections from male M83+/− and M83+/−:TgA mice injected unilaterally in the right dorsal neostriatum with WT human α-synuclein PFFs. Mice were injected at 3 months of age and brains were collected 14–16 weeks post-inoculation. Images shown are near the coordinates of injection, taken at 20× magnification Scale bar is 1 mm. **b** Higher magnification imaging of ubiquitin and psyn129 co-labelling in the ipsilateral ACC of M83+/− and M83+/−:TgA PFF-injected mice. Images were captured at 63X and post-processed at 2X zoom. **c** Higher magnification imaging of Hsp90 (green) and psyn129 (red) **d** STI1 (green) co-localization with psyn129 (red) inclusions in the ipsilateral ACC of M83+/− and M83+/−:TgA PFF-injected mice, captured at 63×, post-processed to 2× zoom. Arrows illustrate areas where considerable co-localization was observed. Images are representative from at least 4 individual mice. **b**–**d** Scale bars are 50 µm. **e** Immunoblot for STI1, psyn129, human α-synuclein in RIPA whole-cell lysates from the ipsilateral cortex of M83+/− and M83+/−:TgA PFF-injected mice. **f** Densitometric quantification of psyn129 (relative to human α-synuclein; *t*(8) = 5.73, *p* = 0.0018). **g** Human α-synuclein quantification (relative to actin loading control; (*t*(8) = 3.839, *p* = 0.005). **h** Immunoblot for STI1, psyn129, human α-synuclein in RIPA lysates from the ipsilateral hippocampus of M83+/− and M83+/−:TgA PFF-injected mice. **i**. densitometric quantifications of psyn129 (relative to human α-synuclein; *t*(8) = 3.961, *p* = 0.0042) and **j.** human α-synuclein (relative to actin loading control; *t*(8) = 791, *p* = 0.0011). **k**. SDS/Urea-soluble STI1, psyn129, and human α-synuclein in ipsilateral cortical lysates. **l**. Densitometric quantification of psyn129 (relative to human α-synuclein; *t*(8) = 2.416, *p* = 0.0421), and **m** human α-synuclein (relative to amido black; *t*(8) = 0.6061, *p* = 0.5613). Data represented as mean ± SEM and analyzed using unpaired *t* test. *p < 0.05, **p < 0.01. *N* = 5 M83+/− PFF (filled blue circles) and *N* = 5 M83+/−:TgA PFF (purple squares)

Given the increased sequestration of Hsp70, Hsp90, and STI1 to insoluble fractions in the brains of PFF-injected M83+/− mice, and the increased levels of STI1 and other chaperone transcripts in PD (Fig. 1n), we tested whether increased STI1 levels are compensatory and could mitigate for potentially decreased chaperone function in synucleinopathy brains. For this purpose, we generated M83+/− mice over-expressing STI1 by crossing them with the STI1TgA line, a Bacterial Artificial Chromosome (BAC) transgenic STI1 over-expressor [12, 13]. In this line, STI1 protein levels are two-to-four fold elevated, which also increases the protein levels of Hsp90ß by about twofold [13], supporting the entwined relationship between these two genes [28]. We injected M83+/− and M83+/−:TgA male mice with PFFs and compared psyn129 labelling and protein levels. Surprisingly, M83+/−:TgA PFF-injected animals presented more psyn129 immunoreactivity compared to M83+/− PFF-injected mice (Fig. 2a) in these semi-quantitative immunolabelling experiments. Despite this increase in labelling, the overall pattern was similar to that of control M83+/− mice, and the psyn129 labelling was widespread in different regions of the cortex, striatum, hippocampus, thalamus, and brainstem, both ipsilateral and contralateral to the injection site (Fig. 2a). Additionally, for M83+/−:TgA PFF-injected mice, labelling in the contralateral side was much more evident than in mice with normal STI1 levels (Fig. 2a). Higher magnification images of cells labelled with α-synuclein phospho-S129 antibody revealed partial overlap between ubiquitin and psyn129 in cytoplasmic inclusions, and increased neuritic inclusions in the Anterior Cingulate Cortex (ACC) (Fig. 2b). Likewise, Hsp90 and psyn129 labelling partially co-localized in cytoplasmic inclusions and in neuritic pathology of both M83+/− and M83+/−:TgA PFF-injected mice (Fig. 2c). Noteworthy, psyn129-positive filamentous labelling in the cell body and neurites found in M83+/−:TgA-PFF-injected mice were positive for STI1 (Fig. 2d). Of note, our qualitative assessment indicates that not all inclusions displayed partial co-localization; however, those with cytoplasmic labelling were more likely to display colocalization with STI1 or Hsp90. Images from the ACC and hippocampus further confirmed increased inclusions of psyn129-positive staining in M83+/−:TgA PFF-injected mice compared to M83+/− mice (Supp. Figs. S3i&j, online resource). Notably, the pattern of staining was qualitatively similar between genotypes (Fig. 2b–d, Supp. Figs.S3 i&j).

We further evaluated the effect of over-expressing STI1 on psyn129 quantitatively using immunoblotting in the ipsilateral cortex and hippocampus with RIPA-soluble lysates, to extract cytoplasmic and membrane-bound proteins. We found twofold more psyn129 in M83+/−:TgA PFF-injected mice normalized by the levels of human α-synuclein in the ipsilateral cortex when compared with mice with normal levels of STI1 (Fig. 2e, f). Notably, we also observed a twofold increase in total human α-synuclein protein levels (Fig. 2e, g). Similarly, psyn129 and total human α-synuclein levels were elevated in the ipsilateral hippocampus of M83+/−:TgA PFF-injected mice compared to M83+/− PFF controls (Fig. 2h–j). In addition, M83+/−:TgA PFF-injected mice presented more SDS/Urea-extractable psyn129 from RIPA-insoluble fractions when compared with mice with normal STI1 levels (Fig. 2k, l). For experiments testing the levels of insoluble proteins, we used only cortex tissue due to higher wield of protein extracts. Phosphorylated synuclein was more abundant in the RIPA soluble fraction than in the insoluble fraction, suggesting that most phosphorylated α-synuclein was present in soluble inclusions. Total human α-synuclein levels were similar between genotypes in the RIPA-insoluble/SDS/urea extractable fraction (Fig. 2k, m), indicating that the insoluble toxic phosphorylated species is impacted by levels of STI1.

We also tested whether increased STI1 and Hsp90 levels per se can lead to accumulation of RIPA-soluble mouse α-synuclein. To do that, we performed immunoblot analyses in control (C57BL/6j) WT and STI1 over-expressing mice (TgA), with no mutations in the *SNCA* gene. As observed in Supp. Figs. S3k&l, levels of endogenous mouse α-synuclein protein were identical in both genotypes. This result further supports the notion that the larger protein burden in PFF-injected animals over-expressing STI1 is related to accumulation of misfolded α-synuclein in response to the PFF injections. Thus, unexpectedly, increased STI1 and Hsp90 levels favor elevated psyn129 accumulation in the brains of PFF-injected M83+/− mice.

### Mice with hypomorphic STI1 alleles and decreased expression of mutated STI1 show reduced α-synuclein pS129 labelling and aggregation

**Fig. 3:**
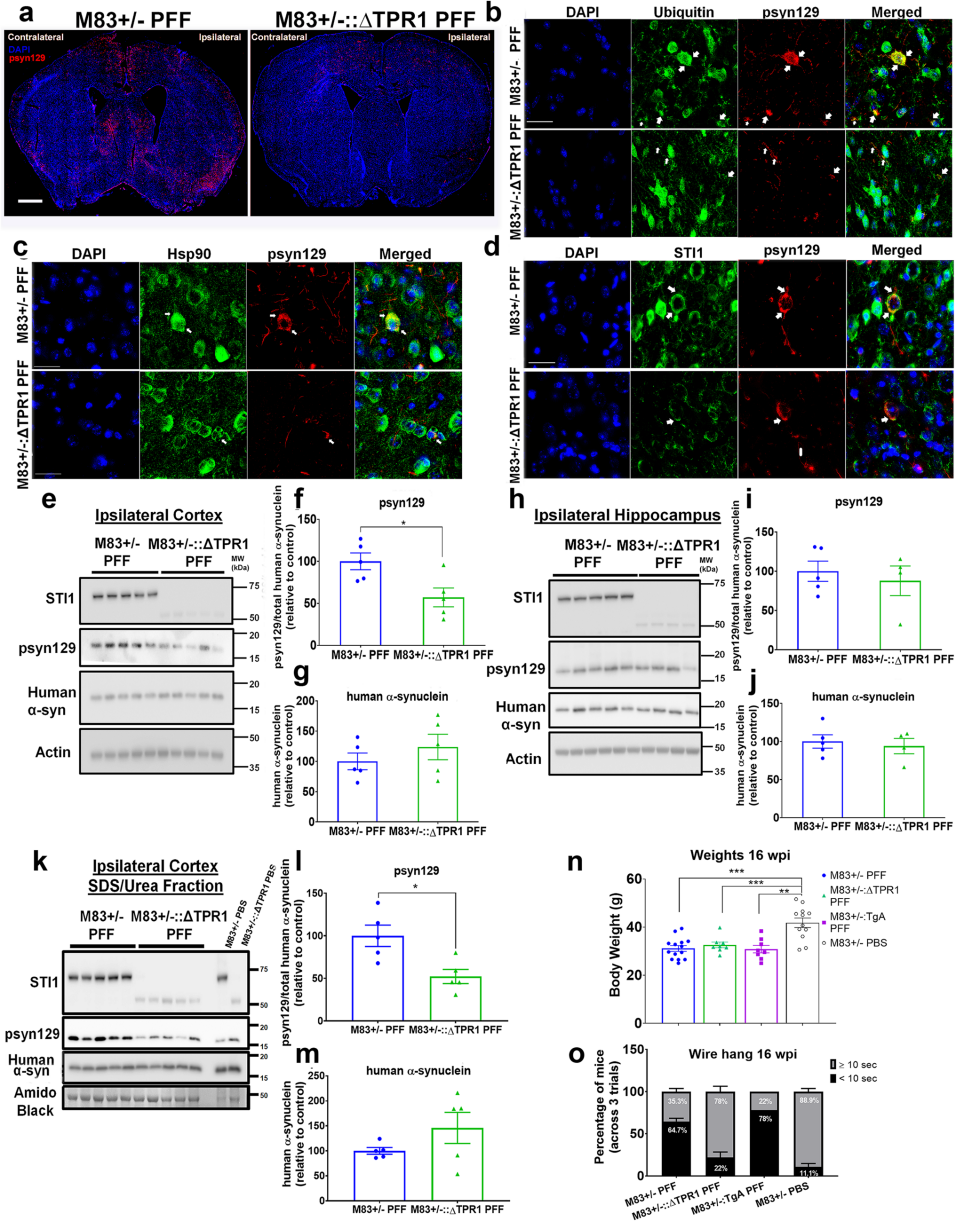
Reduced synucleinopathy in mice carrying STI1 hypomorphic alleles (M83+/−::ΔTPR1 mice). **a** Representative images of psyn129 (red) immunolabelling with nuclear marker (blue) in coronal sections from male M83+/− and M83+/−::ΔTPR1 mice injected with PFFs. Images shown are near the coordinates of injection and were captured at 20× magnification. Ipsilateral and contralateral hemispheres are labelled as respective to right hemisphere intrastriatal injection. Scale bar = 1 mm. Quantification of immunolabelling is shown in Supplementary Fig. S4a&b. **b**–**d** Higher resolution imaging of co-localization analyzes in the Anterior Cingulate Cortex from M83+/− and M83+/−::ΔTPR1 PFF injected mice. Images taken at 63× with Leica Thunder microscope, with images post-processed to 2× zoom. Arrows illustrate areas where considerable co-localization was observed. Scale bars are 25 µm for all images. **b** Colocalization of ubiquitin with psyn129 (pSer129 antibody) **c** Colocalization of Hsp90 with psyn129 (pSer129 antibody) **d** Colocalization of STI1 with psyn129 (pSer129 antibody). **e** Representative immunoblot of STI1, psyn129, and human α-synuclein in whole lysates from the ipsilateral cortex. **f** Densitometric quantification of psyn129 (relative to human α-synuclein; (*t*(8) = 2.852, *p* = 0.0214). **g** Densitometric quantification of human α-synuclein (relative to actin; (*t*(8) = 0.9439, *p* = 0.3729)). *N* = 5 mice/genotype. **h** Representative immunoblot from the ipsilateral hippocampus of M83+/− and M83+/−::ΔTPR1 PFF injected mice. **i** Densitometric quantifications of psyn129 (relative to human α-synuclein; *t*(7) = 0.5526, *p* = 0.598) and **j** human α-synuclein (normalized to actin; *t*(7) = 0.4552, *p* = 0.6627). *N* = 5 M83+/− PFF, *N* = 4 M83+/−::ΔTPR1 PFF mice. **k** Immunoblot of STI1, psyn129, and human α-synuclein in ipsilateral cortical SDS/Urea lysates. **l** Densitometric quantification of psyn129 (relative to human α-synuclein; *t*(8) = 5.236, *p* = 0.0034) and **m** human α-synuclein (relative to amido black; *t*(8) = 1.139, *p* = 0.3063). *N* = 5 M83+/− PFF and *N* = 5 M83+/−::ΔTPR1. M83+/− PFF individual mice represented as blue circles, and M83+/−::ΔTPR1 individual mice identified as green triangles. Data represented as mean ± SEM and analyzed using unpaired *t* test. **n** Mouse body weight at 14–16 wpi, just prior to tissue collection (one-way ANOVA, genotype/group: *F*(3,38), 12.02, *p* < 0.0001; adjusted for multiple comparison’s and analyzed using Tukey’s post hoc test: M83+/− PFF vs. M83+/−::ΔTPR1 PFF *p* = 0.9146, M83+/− PFF vs. M83+/−:TgA PFF *p* = 0.999, M83+/− PFF vs. M83+/− PBS *p* < 0.0001, M83+/−::ΔTPR1 PFF vs. M83+/− PBS *p* = 0.0017, M83+/−:TgA PFF vs. M83+/− PBS *p* = 0.0002). *N* = 16 M83+/− PFF, N = 9 M83+/−::ΔTPR1 PFF, *N* = 9–10 M83+/−:TgA PFF and *N* = 15 M83+/− PBS for weight analyses. M83+/− PBS group includes STI1-WT, ΔTPR1 and TgA M83+/− PBS-injected mice as no significant differences were found between genotypes (one-way ANOVA, genotype/group: *F*(2,12) = 1.114, *p* = 0.3598). **o** Wire hang motor assessment. Mice were suspended upside down and the latency to fall was recorded across 3 trials spaced at least 10 min apart. Percentage of mice (across the three trials) that fell before 10 s ( <) or at and above ( ≥) 10 s. Percentages displayed on graph bars represent the genotype average. Data analyzed using one-way ANOVA with appropriate post hoc comparisons. There was a significant interaction of genotype x time (*F*(3,8) = 62.70, *p* < 0.0001, and data were adjusted for post hoc comparison’s using Tukey’s multiple comparisons test: M83+/− PFF vs. M83+/−::ΔTPR1 PFF *p* = 0.0004 (Cohen’s *d*, effect size = 6.20), M83+/− PFF vs. M83+/−:TgA PFF *p* = 0.176 (Cohen’s *d*, effect size = 1.94), M83+/− PFF vs. M83+/— PBS *p* < 0.0001 (Cohen’s *d*, effect size = 7.78), M83+/−::ΔTPR1 PFF vs. M83+/− PBS *p* = 0.3059 (Cohen’s d, effect size = 1.58), M83+/−:TgA PFF vs. M83+/− PBS *p* < 0.0001(Cohen’s *d*, effect size = 9.72). *N* = 17 M83+/− PFF (filled blue circles), *N* = 9 M83+/−::ΔTPR1 PFF (green triangles), *N* = 10 M83+/−:TgA PFF (purple squares) and *N* = 15 M83+/− PBS (gray open circles) for wire hang analyses. Data represented as mean ± SEM. *p < 0.05, **p < 0.01, ***p < 0.0001

Given that augmented levels of STI1 and Hsp90 favor the accumulation of the psyn129 form of α-synuclein, which normally labels misfolded protein [29, 69, 105], we tested whether STI1 is required for psyn129 propagation and deposition. We crossed M83+/− mice with the homozygous hypomorphic STI1 ΔTPR1 mice, carrying a mutant STI1 protein lacking the TPR1 domain, that is only minimally expressed (STI1 expression level reduced by 80%) [75]. Homozygous ΔTPR1 mice express the mutated protein with a 53 kDa molecular mass instead of the 66 kDa of full-length STI1. The mutant ΔTPR1 protein decreases activity of the chaperone machinery in vivo, with similar effects to those of a profound loss of function of STI1 [75]. Qualitative and quantitative analyses of psyn129 in ipsilateral and contralateral hemispheres revealed significantly reduced psyn129 pathology in M83+/−::ΔTPR1 PFF-injected mice (Fig. 3a, Supp. Fig.S4a) when compared to controls (M83+/− PFF-injected). In the hemisphere ipsilateral to the PFF injection, psyn129 immunoreactivity area was significantly reduced (*p* < 0.05, Supp. Fig. S4a); however, in the contralateral hemisphere, psyn129 pathology showed only a trend to be reduced, which did not reach statistical significance (Supp. Fig. S4a), likely due to the lower amount of pathology found in this hemisphere.

To determine whether knocking down STI1 affected accumulation of psyn129 inclusions to cortical and subcortical regions, we quantified psyn129 immunoreactivity in the striatum, cortex, and hippocampus by immunofluorescence. All of the regions examined in M83+/−::ΔTPR1 PFF-injected mice showed less psyn129 immunoreactivity (Supp. Figs. S4c–j), albeit in the hippocampus the decrease just failed statistical significance (*p* = 0.052) compared to M83+/− PFF-injected mice. Additionally, we observed partial co-localization of psyn129 with ubiquitin (Fig. 3b) and Hsp90 (Fig. 3c), and reduced levels of mutated STI1 co-localized with psyn129 in M83+/−::ΔTPR1 PFF-injected mice (Fig. 3d). Similar results were obtained when using a human-specific psyn129 antibody (MJFR-13), as well as when staining with the antibody EP1536Y that reacts with both mouse and human phosphorylated α-synuclein in mice with more or less STI1 (Supp. Figs. S5a&b, online resource). Overall, in individual cells, psyn129 was less intense in M83+/−::ΔTPR1 PFF-injected mice compared with M83+/− mice injected with PFFs (Fig. 3b–d). Patterns of colocalization were similar among Hsp90, ubiquitin, and STI1 with psyn129 in M83+/− and M83+/−::ΔTPR1 PFF-injected mice, despite the low levels of psyn129 and STI1 (Supp. Figs. S6a-f, online resource). This indicates that although immunolabelling levels of psyn129 are affected by the STI1-ΔTPR1 protein, there were no major changes in protein localization with α-synuclein inclusions.

Quantitative immunoblotting analysis with protein lysates obtained from ipsilateral cortical and hippocampal tissues using RIPA to extract membrane-bound and cytoplasmic proteins from PFF-injected mice further supports immunofluorescence data. We detected significantly reduced psyn129 levels in the ipsilateral cortex of M83+/−::ΔTPR1 PFF-injected mice compared to M83+/− PFF-injected controls (Fig. 3e, f). Notably, total human α-synuclein protein levels were similar between genotypes (Fig. 3e, g). Interestingly, in the hippocampus ipsilateral to PFF injection, psyn129 and total human α-synuclein levels were comparable between genotypes (Fig. 3h–j, suggesting a region-specific effect. We did not observe significant changes in endogenous mouse α-synuclein protein levels in ΔTPR1 mice (Supp. Figs. S6g&h, online resource), again indicating that modifying the levels of STI1 impacts mostly psyn129 accumulation.

Due to the presence of psyn129 α-synuclein inclusions in PFF-injected animals, we next investigated whether reducing the levels of STI1 modified the amount of RIPA-insoluble (SDS/Urea-soluble) α-synuclein. We found reduced psyn129 protein levels in the cortical fraction (Fig. 3k, l), with no significant alterations in total human α-synuclein (Fig. 3k, m). These results indicate that reducing the levels of STI1 using the ΔTPR1 allele in the M83+/− PFF model can decrease both soluble and insoluble aggregated psyn129, at least in certain brain regions.

Mice injected with PFFs or PBS were followed for 13–16 weeks after injection, until weight loss and health decline were observed in the cohort. In general, all PFF-injected mice lost weight when compared to PBS-injected mice (Fig. 3n), suggesting progression of disease in all genotypes. We analyzed gross motor behavior in these mice and found that whereas most M83+/− and M83+/−:TgA PFF-injected mice were unable to maintain their grip when upside down, the majority of M83+/−::ΔTPR1 PFF-injected mice performed similarly to PBS-injected mice (Fig. 3o), suggesting some protection by hypomorphic STI1.

### STI1 facilitates S129 phosphorylation and toxicity of α-synuclein

M83+/− PFF-injected mice present fast prion-like spreading of α-synuclein, as reported elsewhere, with pathology starting to accumulate within 30 days after PFF inoculation, with more robust pathology and motor deficits 90 days post-inoculation [82, 115]. Additionally, neuropathology in PFF-injected mice is widespread across the brain and present in high concentrations in anatomically connected regions [62, 82]. To evaluate α-synuclein misfolding and toxicity in a less-aggressive mouse model of synucleinopathy, without PFF seeding, we used M83 homozygous (M83+/+) mice with two copies of the A53T *SNCA* transgene, which develop a more gradual but variable phenotype. These mice present with symptomatic disease between 8 and 16 months of age (median age of onset 11–12 months [56]), accompanied by elevated levels of psyn129 [15, 56, 126].

**Fig. 4:**
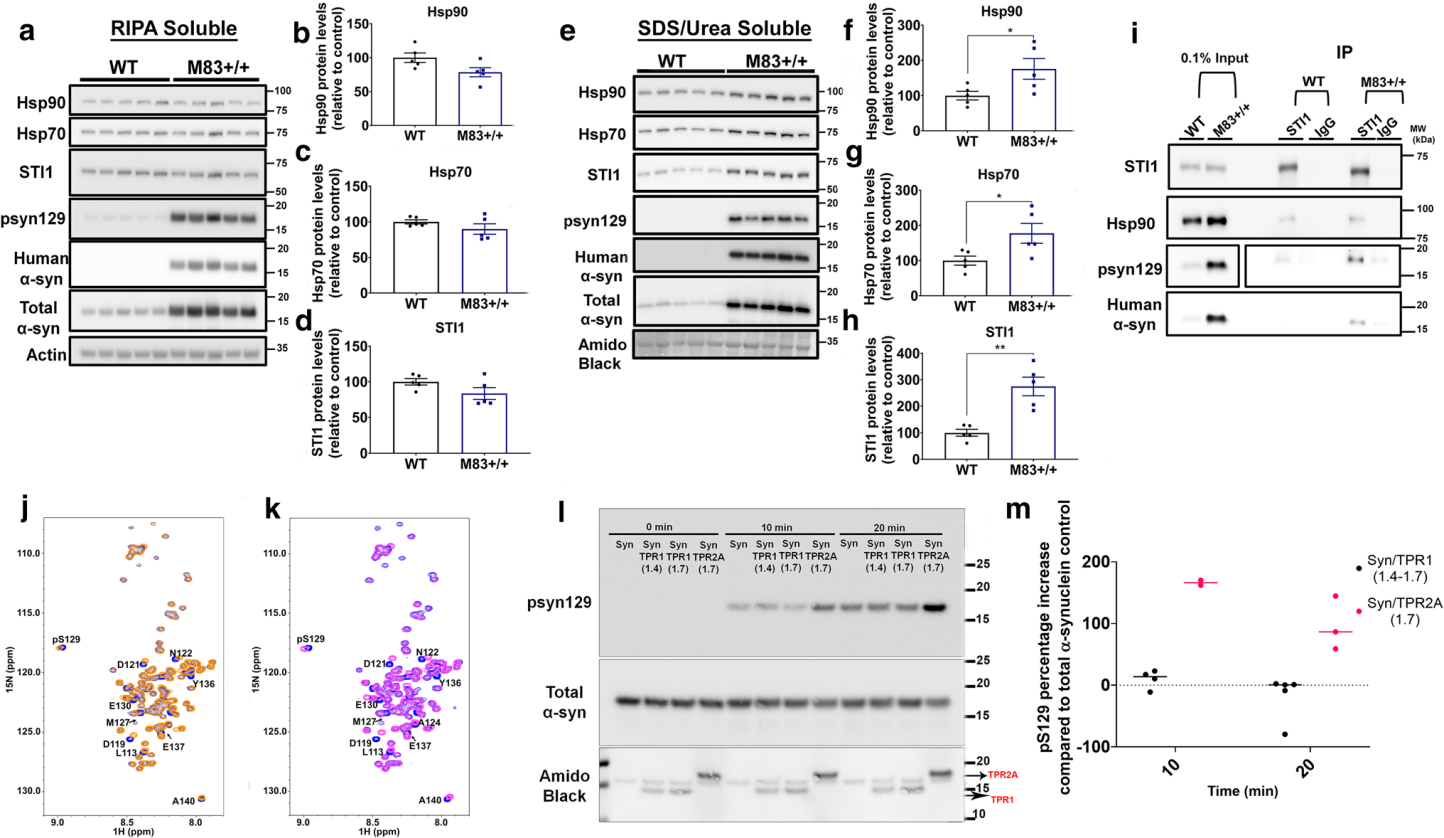
pS129 phosphorylation of α-synuclein is regulated by STI1. **a** Representative immunoblot of panHsp90, Hsp70, STI1, pSer129 α-synuclein (psyn129), human α-synuclein, and total (mouse and human) α-synuclein in RIPA lysates from cortex of M83+/+ . **b** Densitometric quantification of Hsp90 (*t*(8) = 2.12, *p* = 0.06). **c** Densitometric quantification of constitutive Hsp70 (*t*(8) = 1.28, *p* = 0.23). **d** Densitometric quantification of STI1 (*t*(8) = 1.73, *p* = 0.12). **e** Representative immunoblots from 2% SDS and 4 M Urea extraction in cortical lysates for panHsp90, Hsp70, STI1, psyn129, human α-synuclein, and total (mouse and human) α-synuclein. **f** Densitometric quantification of Hsp90 (*t*(8) = 2.39, *p* = 0.044), **g** Hsp70 (*t*(8) = 2.50, *p* = 0.037), and **h.** STI1 (*t*(8) = 4.652, *p* = 0.002). **i** Immunoprecipitation by STI1 (5 µg) and IgG (5 µg) antibodies was performed in both control non-transgenic WT and M83+/+ 10–11-month-old male mouse cortical tissues. STI1 co-immunoprecipitated Hsp90 in animals of both genotypes. Likewise, STI1 co-immunoprecipitated psyn129 at low levels in controls, as aged non-transgenic mice also express low levels of psyn129, observed in input lanes and STI1 IP lanes. STI1 also co-immunoprecipitated human α-synuclein, exclusively in M83+/+ animals, as would be expected given control mice do not express the human protein [56]. Experiments were repeated three times, with different mice. Within the psyn129 row, images that were cropped separately are from the same immunoblot, at different exposure times. Graphs in **b**–**d** and **f**–**h** with black circles (WT) and dark blue squares (M83+/+), each individual circle or square represents a single animal. Data represented as mean ± SEM and analyzed using unpaired two-tailed *t* test. *p < 0.05, **p < 0.001. **j** 600 MHz ^1^H-^15^ N HSQC spectrum of 100 µM psyn129 (in 20 mM Hepes, 50 mM NaCl, pH 7.2) in the absence (black) and the presence (orange) of 200 µM of STI1. **k** 600 MHz ^1^H-^15^ N HSQC spectrum of 100 µM psyn129 (in 20 mM Hepes, 50 mM NaCl, pH 7.2) in the absence (black) and the presence (magenta) of 200 µM of TPR2A domain. **l** Representative immunoblot for in vitro phosphorylation of α-synuclein at 0, 10, and 20 min of incubation with PLK3 in the presence and absence of TPR1 or TPR2A. The left corner of the gels shows molecular weight markers, **m** Quantification of distinct blots prepared using independent prepared samples. The results using two concentrations of TPR1 were pooled together, as there was no difference between them. At 0 min, PLK3 was omitted. Psyn129 was detected with the same antibody as in other experiments, demonstrating the specificity for phosphorylated α-synuclein

We first tested whether STI1 and chaperones are present in insoluble fractions in M83+/+ mice cortical lysates, as observed in M83+/− PFF-injected mice. Analysis of cortical brain protein extracts from 11–12-month-old non-transgenic (WT) and M83+/+ male mice (when 50% of the mice were symptomatic [56]) revealed comparable Hsp70, Hsp90 and STI1 protein levels in the RIPA-soluble fraction on both genotypes (Fig. 4a–d). However, in SDS/urea soluble lysates (RIPA-insoluble), Hsp90 and Hsp70 protein levels were 1.5-fold higher in M83+/+ tissues compared to non-transgenic mice (Fig. 4e–g). Notably, STI1 protein levels were approximately three-fold higher in M83+/+ SDS/Urea soluble lysates compared to non-transgenic mice (Fig. 4e, h). We conclude that the Hsp90/Hsp70/STI1 chaperone machinery is abnormally aggregated with α-synuclein in M83+/+ mice presenting increased α-synuclein misfolding and S129 phosphorylation.

Given that STI1 interacts with endogenous mouse α-synuclein, we further tested whether STI1 could form a complex with phosphorylated human α-synuclein in M83+/+ mouse brains. For these experiments, we used cortical tissue from control and M83+/+ male mice aged 10–11-months, which was the time-point where we observed at least 50% of the cohort to be physically declining, as reported previously [56]. STI1 co-immunoprecipitated Hsp90, in both non-transgenic (WT) and M83+/+ mice (Fig. 4i). Furthermore, we observed co-immunoprecipitation of psyn129 in M83+/+ mice, and STI1 pulldown co-immunoprecipitated human α-synuclein only from M83+/+ mice (Fig. 4i). These results indicate that STI1 can interact with non-phosphorylated and phosphorylated α-synuclein in the mouse brain, further supporting the hypothesis that STI1 is directly involved in the toxicity and deposition of malignant α-synuclein.

To further test for direct interaction between STI1 and phosphorylated α-synuclein, we used NMR. Recombinant human α-synuclein was phosphorylated at S129 using Polo-like kinase 3 (PLK3 kinase), which is part of a family of kinases that is thought to phosphorylate α-synuclein in vivo [14, 89]. Comparison of phosphorylated and non-phosphorylated α-synuclein using NMR indicated that under these conditions almost all recombinant protein was phosphorylated (Supp. Fig. S7, online resource). Noticeable chemical shift changes were observed for the C-terminal residues of phosphorylated α-synuclein in the presence of full-length STI1 (Fig. 4j) or TPR2A domain (Fig. 4k), indicating that the C-terminal domain of psyn129 still interacts with the STI1 TPR2A domain. NMR titration analysis shows that psyn129 has a binding affinity (K_D_ of 56 ± 27 µM) for STI1 TPR2A similar to that of α-synuclein (Supp. Fig. S7, online resource). To test the possibility that STI1 may regulate phosphorylation of α-synuclein at S129, we incubated α-synuclein with PLK3 and the TPR2A domain (which reproduced the binding of STI1 to α-synuclein) or the TPR1 domain (which does not bind to α-synuclein) for 10 or 20 min, then resolved proteins by SDS-PAGE, and blotted for α-synuclein and psyn129 (Fig. 4l). This analysis revealed that α-synuclein was more phosphorylated at S129 in the presence of TPR2A in all time points than in samples containing TPR1 (Fig. 4l, m). Predictably, in samples without PLK3 (0 min), the phospho-specific antibody for α-synuclein did not recognize the protein, confirming the specificity of the Western blots. These results suggest that STI1 may facilitate S129 synuclein phosphorylation.

### M83 homozygous mice expressing ΔTPR1 alleles have less psyn129 pathology markers, improved cognition, and imaging biomarkers

**Fig. 5:**
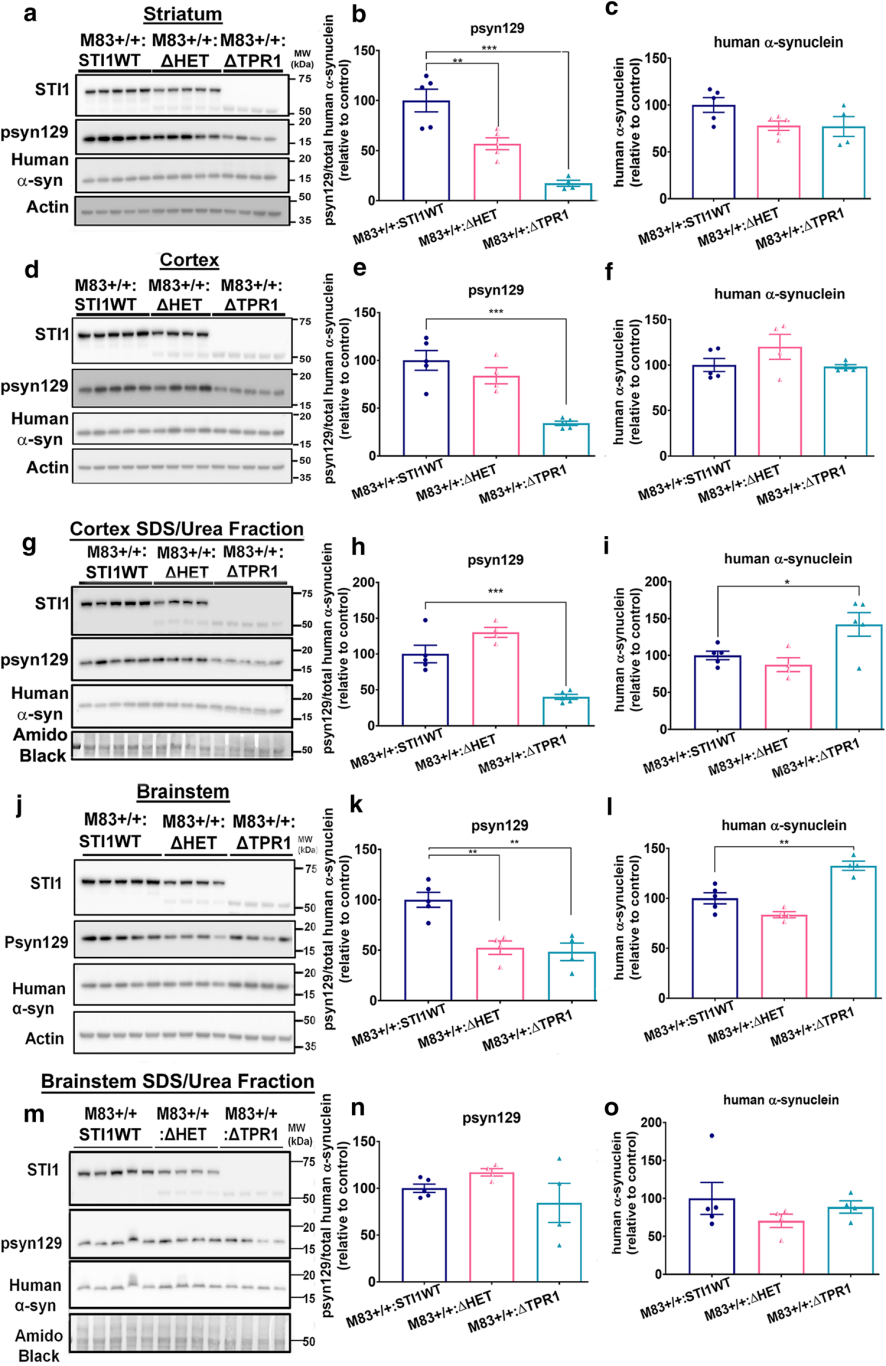
Reduced psyn129 in M83+/+ mice carrying STI1 hypomorphic alleles. **a** Immunoblot from RIPA extracted striatal lysates from M83+/+:ΔTPR1 mice. **b** Densitometric quantification of psyn129 (ab51253) antibody, relative to total human α-synuclein (genotype: *F*(2,11) = 2484, *p* < 0.0001, adjusted for multiple comparisons with Dunnett’s multiple comparisons: M83+/+:STI1WT vs. M83+/+:ΔHET *p* = 0.005, M83+/+:STI1WT vs. M83+/+:ΔTPR1 *p* < 0.0001) **c** Human α-synuclein relative to actin loading control (genotype: *F*(2,11) = 2.887, *p* = 0.098). **d** Immunoblot from RIPA cortical lysates and **e.** Densitometric quantifications of psyn129 (ab51253) relative to total human α-synuclein (genotype: *F*(2,11) = 20.57, *p* = 0.0002, adjusted for multiple comparisons with Dunnett’s multiple comparisons test: M83+/+:STI1WT vs. M83+/+:ΔHET *p* = 0.305, M83+/+:STI1WT vs. M83+/+:ΔTPR1 *p* = 0.0001) **f** Densitometric quantification of human α-synuclein relative to actin loading control (genotype: *F*(2,11) = 2.048, *p* = 0.175). **g** Representative immunoblot for STI1, psyn129, and human α-synuclein in M83+/+ :ΔTPR1 mice in SDS/Urea-soluble cortical fraction (RIPA-insoluble fraction). **h** Densitometric quantification of psyn129 (relative to human α-synuclein; genotype, *F*(2,11) = 106, *p* = 0.0009, adjusted for multiple comparisons with Dunnett’s multiple comparisons: M83+/+:STI1WT vs. M83+/+:ΔHET *p* = 0.619, M83+/+:STI1WT vs. M83+/+:ΔTPR1 *p* = 0.0008). **i** Densitometric quantification of human α-synuclein (relative to amido black) (genotype, *F*(2,11) = 19.49, *p* = 0.0002, Dunnett’s multiple comparisons: M83+/+:STI1WT vs. M83+/+:ΔHET *p* = 0.868, M83+/+:STI1WT vs. M83+/+:ΔTPR1 *p* = 0.0003). **j** Immunoblot for RIPA brainstem lysates and **k** Densitometric quantifications of psyn129 (ab51253) relative to total human α-synuclein (genotype *F*(2,10) = 14.93, *p* = 0.001, Dunnett’s multiple comparisons test: M83+/+:STI1WT vs. M83+/+:ΔHET *p* = 0.0024, M83+/+:STI1WT vs. M83+/+:ΔTPR1 *p* = 0.0013). **l** Densitometric quantifications of human α-synuclein relative to actin loading control (genotype *F*(2,10) = 25.60, *p* = 0.0001, Adjusted for multiple comparisons with Dunnett’s multiple comparisons test: M83+/+:STI1WT vs. M83+/+:ΔHET *p* = 0.0594, M83+/+:STI1WT vs. M83+/+:ΔTPR1 *p* = 0.0012). **m** Immunoblots from SDS/Urea-soluble brainstem tissue lysates. **n** densitometric quantification psyn129 (relative to human α-synuclein) (genotype, *F*(2,10) = 1.827, *p* = 0.217). **o**. Densitometric quantification of human α-synuclein (relative to amido black) (genotype (*F*(2,10) = 0.9151, *p* = 0.4316). *N* = 4–5 mice per genotype. Individual dark blue circles (M83+/+:STI1WT), half-filled pink triangles (M83+/+:ΔHET), or filled turquoise triangles (M83+/+:ΔTPR1) represent individual animals. White open circle represents M83 non-transgenic mice (WT mice). Immunoblotting data presented as mean ± SEM and analyzed using one-way ANOVA with appropriate post hoc comparisons, when required. *p < 0.05, **p < 0.01, ***p < 0.0001

To further investigate the role of STI1 for α-synuclein toxicity in vivo, we generated mice with one or two copies of the STI1 hypomorphic allele ΔTPR1 (ΔHET, 40% less STI1 or ΔTPR1, 80% less STI1) expressing two copies of the human A53T transgene (M83+/+). Immunoblotting analysis demonstrated reduced levels of psyn129 in M83+/+ animals crossed with ΔTPR1 mice compared to littermate M83+/+ mice (M83+/+:STI1WT) (Fig. 5a, l), in line with our observations in PFF-injected mice. In the striatum, we detected significantly reduced psyn129 levels in both M83+/+:ΔHET mice (Fig. 5a, b, ~ 50%) and M83+/+:ΔTPR1 mice (Fig. 5a, b, ~ 80%), compared to M83+/+:STI1WT mice. Additionally, we found 65% lower psyn129 (normalized to total human α-synuclein levels) in the cortex of M83+/+:ΔTPR1 mice (Fig. 5d, e); however, no significant difference was found between M83+/+ :STI1WT and M83+/+ :ΔHET mice in the cortex (Fig. 5e), indicating a region-specific and STI1 dose-specific effect. Total human α-synuclein levels were unchanged across genotypes in striatal lysates and cortical tissue lysates (Fig. 5c, f). ΔTPR1 expression also decreased psyn129 in RIPA-insoluble, SDS/urea soluble cortical fractions of M83+/+ mice (Fig. 5g, i). In the brainstem, we found decreased psyn129 levels in both M83+/+:ΔHET mice (Fig. 5j, k, ~ 50%) and M83+/+:ΔTPR1 mice (Fig. 5j, k, ~ 60%) compared to M83+/+:STI1WT mice. However, total human α-synuclein levels were moderately elevated in M83+/+:ΔTPR1 mice brainstem lysates (Fig. 5j, l). There was no difference between genotypes in psyn129 and human α-synuclein protein levels in SDS/Urea-soluble brainstem lysates (Fig. 5m–o), albeit the values were more variable. Given that disease onset is quite variable in M83+/+ mice, it is possible that the differences occur because of this variability.

**Fig. 6:**
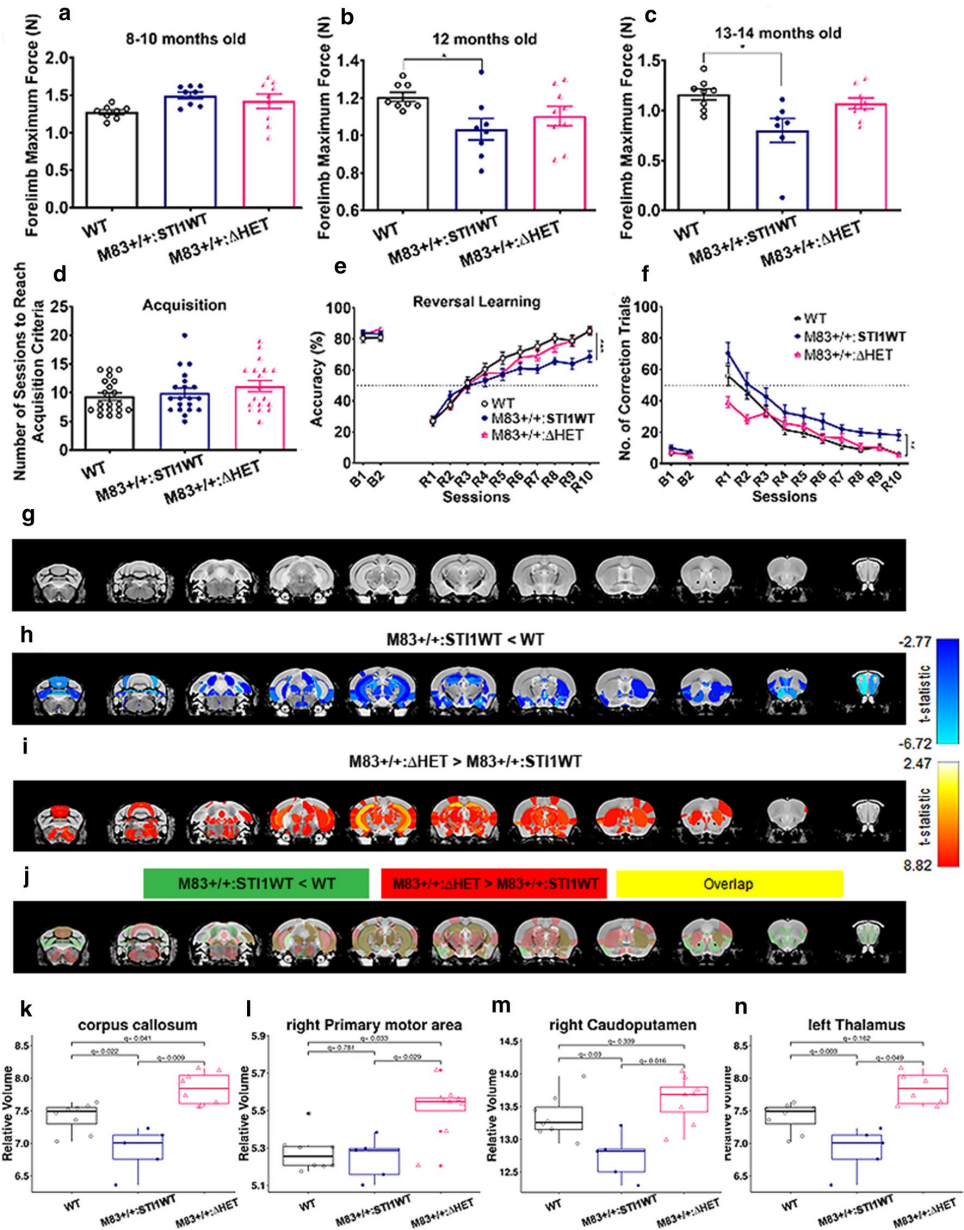
Improved behavior and markers of brain atrophy in M83+/+ mice carrying STI1 hypomorphic allele. **a** Grip force (N) analysis at 8–10 months of age for WT control mice (*n* = 8), M83+/+:STI1WT mice (*n* = 8) and M83+/+:ΔHET mice (*n* = 9) (genotype: *F*(2,22) = 2.761, *p* = 0.0851, adjusted for multiple comparisons with Tukey's multiple comparisons test: WT vs. M83+/+:STI1WT *p* = 0.0754, WT vs. M83+/+:ΔHET *p* =0.2828, M83+/+:STI1WT vs. M83+/+:ΔHET *p* = 0.6983). **b.** Grip force analysis at 12 months of age for WT control mice (*n* = 8), M83+/+:STI1WT mice (*n* = 8) and M83+/+:ΔHET mice (*n* = 9) (genotype: *F*(2,22) = 3.31, *p* = 0.0554, adjusted for multiple comparisons with Tukey's multiple comparisons test: WT vs. M83+/+:STI1WT, *p* = 0.0455, WT vs. M83+/+:ΔHET *p* = 0.2834, M83+/+:STI1WT vs. M83+/+:ΔHET *p* = 0.5418). **c** Grip force analysis performed at 13–14 months old for one-way ANOVA (genotype: *F*(2,21) = 5.583, *p* = 0.0114, adjusted for multiple comparisons with Tukey's multiple comparisons test: WT vs. M83+/+:STI1WT *p* = 0.0106, WT vs. M83+/+:ΔHET *p* = 0.6579, M83+/+:STI1WT vs. M83+/+:ΔHET, *p* = 0.0551). **d** Number of sessions to reach acquisition criteria in the PVD-R test using Marble (S−) and Fan (S+) stimuli during the acquisition phase. One-way ANOVA (genotype: *F*(2,55) = 1.238, *p* = 0.2978, adjusted for multiple comparisons with Tukey's multiple comparisons test: WT vs. M83+/+:STI1WT *p* = 0.8587, WT vs. M83+/+:ΔHET, *p* = 0.2751, M83+/+:STI1WT vs. M83+/+:ΔHET, *p* = 0.5555). **e** Reversal learning phase for the PVD-R test. Mice were baselined for 2 days (B1 and B2) and tested on reversal learning. Repeated-measures two-way ANOVA (Session: *F*(6.820, 373.9) = 154.2, *p* < 0.0001; Genotype: *F*(2, 55) = 2.821, *p* = 0.0682; Interaction: *F*(22, 603) = 3.744, *p* < 0.0001, adjusted for multiple comparisons with Tukey's multiple comparisons test: WT vs. M83+/+:STI1WT *p* = 0.0025, WT vs. M83+/+:ΔHET, *p* = 0.7172, M83+/+:STI1WT vs. M83+/+:ΔHET *p* = 0.0311)). **f** Number of correction trials during reversal learning. Repeated-measures two-way ANOVA (Session: *F*(4.312, 237.1) = 84.47, *p* < 0.0001; Genotype: *F*(2, 55) = 7.170, *p* = 0.0017; Interaction: *F*(22, 605) = 2.866, *p* < 0.0001, Adjusted for multiple comparisons with Tukey's multiple comparisons test: WT vs. M83+/+:STI1WT, *p* < 0.0001, WT vs. M83+/+:ΔHET *p* = 0.5810, M83+/+:STI1WT vs. M83+/+:ΔHET, *p* < 0.0001). WT control mice (*n* = 20), M83+/+:STI1WT mice (*n* = 20), and M83+/+:ΔHET mice (*n* = 18). Parameters were measured across baseline days 1 and 2 (B1, B2) and reversal days 1 to 10 (R1–R10). **g** Coronal slices of the group average MRI brain from posterior (left) to anterior (right) slices. **h** Volumetric differences between M83+/+:STI1WT and WT. **i** Volumetric differences between M83+/+:ΔHET and M83+/+:STI1WT. In terms of t-statistics thresholded at FDR, 5% are displayed on top of the group average brain, such that the color maps denote the significance of the difference observed; cooler colors describe volumetric decreases whereas warmer colors described volumetric increases for the group of interest. We observed global volumetric decreases throughout the brain in M83+/+ mice harboring two copies of the human A53T *SNCA* transgene compared to WT mice (images in h) and volume increases in the M83 mice with one copy of the TPR1 hypomorphic allele (M83+/+:ΔHET) (compared to M83+/+:STI1WT mice with no ΔTPR1 copy, images in **i**. **j** Examination of both patterns of volumetric difference by overlaying the two. Green regions denoting smaller volumes for M83+/+:STI1WT compared to WT, red regions denoting larger volumes for the M83+/+:ΔHET compared to M83+/+:STI1WT, and yellow denoting the regions observed in both comparisons). We observed, for several of the regions, that the volumetric decreases for the M83+/+:STI1WT mice (compared to WT) get reverted with the introduction of the ΔTPR1 hypomorphic allele, such that we observe volumetric increases in M83+/+:ΔHET mice compared to M83+/+:STI1WT controls. **k**–**m** Quantitative analysis of some of the volumetric changes observed (albeit with differing *t* values). **k** White matter tracts such as the corpus callosum. **l** Right primary motor areas. **m** Subcortical structures (right caudoputamen). **n** Thalamus. We observed reversal of volumetric decreases in the corpus callosum, motor cortex, right caudateputamen, and thalamus. Notably, we also observed regions where atrophy is not recovered with the presence of ΔTPR1 allele. Namely, the (left caudoputamen), we did not observe a reversal of the volumetric decrease in M83+/+ . In fact, in the left caudoputamen, we observed further volumetric increase in the M83+/+:ΔHET mice (compared to M83+/+ mice). Similarly, we observed regions that show volume increases only in the M83+/+:ΔHET mice. Namely, the right primary motor area. Individual unfilled black circles (WT), dark blue circles (M83+/+:STI1WT), and half-filled pink triangles (M83+/+:ΔHET) represent individual animals

To evaluate whether reducing levels of STI1 using the ΔTPR1 allele could protect against the consequences of α-synuclein toxicity, we first evaluated motor behavioral performance. We failed to detect major changes in motor behavior in M83+/+ mice (locomotor activity, rotarod, and wire hang at 6–8 months of age, data not shown, but results were similar to [95]). However, we observed a progressive decrease in forelimb grip force in M83+/+ : STI1WT animals compared to controls (WT) after 12 months of age (Fig. 6a, b). For behavioral experiments, we used mice with one STI1 hypomorphic allele to avoid potential confounding brain volume and behavioral effects of homozygous ΔTPR1 mice [75]. Reducing STI1 with just one of the ΔTPR1 alleles mitigates grip force deficits of M83+/+ mice after 12 months of age (Fig. 6c), indicating that even partial reduction in psyn129 protein levels seems sufficient to restore some motor deficits.

To understand whether increased psyn129 can cause high-level cognitive deficits and affect brain function, we used touchscreen tests that probe cognitive domains that have been previously shown to be affected in synucleinopathies [40, 51, 76, 80, 95, 101, 118], with focus on attention [11, 13, 71, 87] and reversal learning [11, 71, 87]. M83+/+:STI1WT mice presented no attentional deficits when tested using the 5-choice serial reaction time task (5-CSRTT) at 8 months of age (Supp. Fig. S8a-g, online resource). In contrast, using pairwise visual discrimination and reversal (PVD-R), which tests the ability to relearn a new contingency [11, 71], (see Supp. Fig. S9a, online resource for experimental timelines and recorded parameters for PVD task), we found that M83+/+:STI1WT are able to discriminate between two images similar to controls (Fig. 6d acquisition), but presented deficits in the late part of the reversal phase (Fig. 6e, f), as early as 6–8 months of age. In contrast, mice expressing one ΔTPR1 allele (M83+/+:ΔHET), which reduces STI1 by about 50%, present mitigation of these deficits (Fig. 6e, f), suggesting that decreasing STI1 function rescued cognitive dysfunction due to α-synuclein toxicity.

To further evaluate brain toxicity in conditions in which mice had cognitive deficits or were rescued, we performed *ex vivo* T1-weighted magnetic resonance imaging (MRI) (70-micron isotropic voxel resolution acquired using a 7 T Bruker Biospec) on the brains of a subset of mice that performed the touchscreen testing. We sought to investigate brain abnormalities 1) due to over-expression of human A53T *SNCA* (in M83+/+ mice compared to non-transgenic mice) and 2) to examine whether the brain differences are reverted with the presence of one copy of the ΔTPR1 allele. Moreover, since volumetric differences were previously found in ΔTPR1 but not ΔHET mice [75], we focused our analyses on this genotype crossed with M83+/+ animals that performed the touchscreen tests. We observed global volumetric decreases throughout the brain that survive a 5% False Discovery Rate [8] in M83+/+:STI1WT mice when compared to non-transgenic WT mice (Fig. 6h). Notably, for these regions, we similarly observe volumetric increases in the M83+/+:STI1WT mice with one copy of the ΔTPR1 allele (M83+/+:ΔHET, compared M83+/+:STI1WT mice), suggesting that patterns of volumetric decrease were mitigated by the introduction of the ΔTPR1 hypomorphic allele (Fig. 6i). This effect was observed specifically in subcortical regions and the cortical areas to which those regions project, along with the corresponding white matter tracts. Notably, we also observed a few regions in which atrophy was not recovered with the ΔTPR1 allele, including regions close to the caudoputamen. Full volumetric results for each of the comparisons performed (M83+/+:STI1WT versus WT mice, and M83+/+:ΔHET versus M83+/+:STI1WT) are provided in Supplementary Table 5 (online resource) for each structure in the Allen Brain Atlas and includes standardized beta-, *t*-, *p*-, and *q*-values. Overall, these experiments suggest that pathology and toxicity of α-synuclein can be mitigated by decreasing the gene dosage of STI1.

## Discussion

**Fig. 7:**
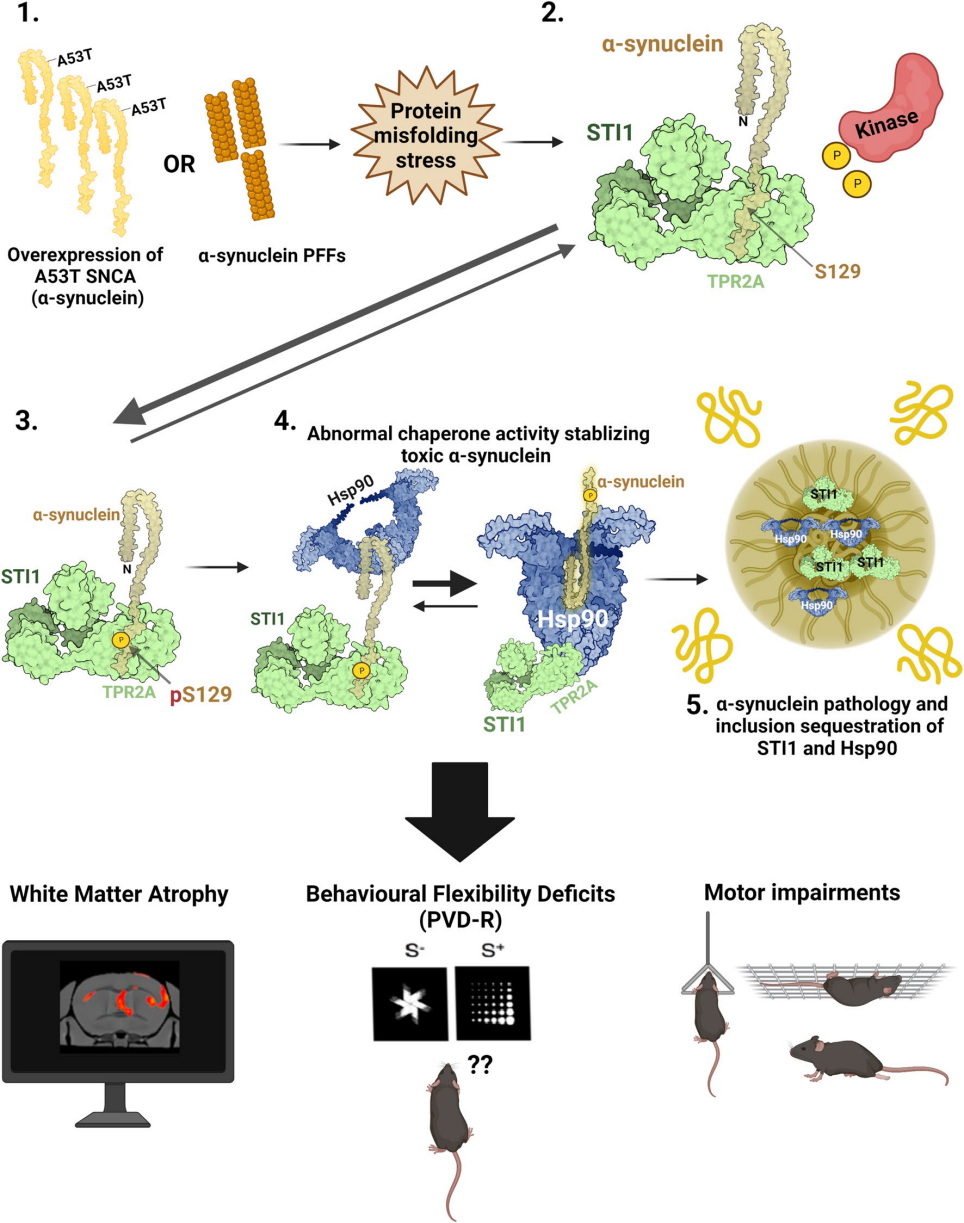
Hypothetical model of STI1 regulation of toxic α-synuclein and promotion of phosphorylation at S129. **1** Toxic α-synuclein species disturb chaperome and contribute to protein misfolding stress. **2** The TPR2A domain of STI1 interacts with the C-terminal domain of α-synuclein forming a complex in the brain. **3** The STI1-α-synuclein interaction may facilitate α-synuclein phosphorylation at S129. **4** STI1 may scaffold α-synuclein to deliver it to Hsp90. **5** STI1 and Hsp90 are sequestered into inclusions in multiple brain regions. Thus, α-synuclein function and potential to aggregate may depend on different chaperones and their co-chaperones in specific cell types. These toxic effects lead to white matter atrophy, cognitive, and motor deficits. Figure designed with BioRender.com software

Using mouse models of spontaneous and seeded α-synuclein misfolding, we identified a vicious cycle in which accelerated proteostatic stress induced by α-synuclein misfolding facilitates accumulation of the chaperone machinery in insoluble species. Conversely, we found that STI1 interaction with α-synuclein contributes to its toxicity, tissue inclusions, and cognitive dysfunction (Fig. 7). Mechanistically, the TPR2A domain of STI1 interacts with the C-terminal domain of α-synuclein and STI1-α-synuclein form a complex in the brain. Unlike previous mass spectrometry analyses in HEK293 cells, which indicated STI1 interaction with α-synuclein via the N-terminus region [21], our findings reflect STI1 interaction with multiple regions of α-synuclein. This more extensive interaction of STI1 with clients is compatible with recent results using Cryo-EM of Hsp90 chaperone complex with the glucocorticoid receptor [125], which revealed unexpected interactions of STI1 directly with client proteins. While the sequence of these interactions between α-synuclein, STI1, Hsp70, and Hsp90 remains to be resolved, the α-synuclein interface with TPR2A suggests some overlap with Hsp90 binding. These results indicate that STI1 may scaffold α-synuclein to deliver it to Hsp90, as previously described with other protein complexes [116, 125], and more recently in a model of chaperone cycle for the classical client protein, glucocorticoid receptor [37, 125]. The correlation between expressed chaperone and co-chaperone transcripts and α-synuclein in humans are also supportive of a role for the chaperome in α-synuclein function in multiple brain regions and ages, especially considering that other members of the machinery also present increased expression in Parkinson’s disease brains [19, 26, 106, 121]. Together, these results support the notion that α-synuclein function and potential to aggregate may depend on different chaperones and their co-chaperones in specific cell types. Indeed, the increased levels of STI1 and other chaperone mRNAs (and presumably proteins) found in the brain of PD patients concurs with the findings of increased levels of aggregated STI1 in insoluble protein fractions in mice. It should be noted that all the chaperome genes we examined in humans have been shown to be also differentially regulated during Lewy-body formation in cultured neurons in a recent study [86].

Phosphorylation of α-synuclein at S129 is a well-established and abundant pathological marker in PD and DLB [3, 53]. α-Synuclein association into inclusions promotes increased phosphorylation at S129 and enhances the propensity to self-aggregate and recruit more α-synuclein to form inclusions [14, 29, 69, 86, 105]. However, whether phosphorylation at S129 has causal roles in aggregation is not entirely clear [4, 93]. Indeed, recent work suggests that phosphorylation of S129 occurs after more mature aggregation of the proteins [86] and that S129 phosphorylation may decrease aggregation of α-synuclein [55]. However, our results suggest that STI1 plays a role in vivo in S129 phosphorylation, but also facilitates α-synuclein inclusion deposition.

In both mouse models of α-synuclein misfolding (M83+/+ and M83+/− PFF injection), reduced levels of STI1 led to less psyn129, either in RIPA-soluble or insoluble forms. In contrast, increased expression of STI1 and Hsp90 augmented psyn129 and insoluble α-synuclein deposition. It is unlikely that the regulation of psyn129 by STI1 is a result from increased proteasome activity due to reduced STI1, given that normal levels of STI1 seem to be necessary for optimal proteasome function [16]. STI1 is also important for degradation via chaperone-mediated autophagy [2], suggesting that decreased STI1 levels should not activate this degradation pathway to decrease the levels of phosphorylated protein. In vitro NMR and phosphorylation analyses are supportive of a direct role of STI1 interaction with α-synuclein, perhaps helping S129 to become more accessible to kinases or accelerating the rate by which α-synuclein is phosphorylated when aggregated or increasing kinase activity (Fig. 7). However, these conformational changes induced by STI1 may also favor misfolding of α-synuclein, leading to increased propensity to accumulate and then be phosphorylated. In agreement with these results, a recent proteomic study found STI1 to be present early during the formation of initial aggregates of α-synuclein [86], placing these two proteins in the proper location to facilitate S129 phosphorylation. The exact mechanism by which STI1 regulates this process and how Hsp70, Hsp90, and other co-chaperones participate in it will need to be further investigated. Of note, our phosphorylation experiments conducted in vitro with soluble proteins may provide a very different biochemical environment than during phosphorylation of filamentous α-synuclein in vivo*.* Nonetheless, these results suggest that α-synuclein misfolding and its propensity to be phosphorylated at S129 and form inclusions are regulated by STI1 and the Hsp90 chaperone machinery in vivo.

The role of STI1 in protein misfolding and aggregation in vivo is still far from being understood. In yeast, STI1 buffered proteotoxicity by rearranging amyloid-like proteins into SDS-resistant inclusions [128], whereas extracellular STI1 protected cultured neurons against amyloid-β toxicity [92]. Conversely, but similar to results shown here, STI1 over-expression in vivo led to elevated levels of insoluble amyloid-β, accumulation of STI1 and Hsp90 in amyloid plaques, and worsened spatial memory of 5xFAD mice [74]. Aging, the most important risk factor for protein misfolding diseases, causes imbalances in the chaperone machinery [20, 75], and STI1 is reduced with normal aging in mammals [75]. In Alzheimer’s disease human brains, STI1 levels are elevated [92], and STI1 accumulates in mature human extracellular plaques [74]. Hsp90 and STI1 were found also by others to be upregulated in PD and DLB patient brains [19, 106, 121]. These findings, together, suggest that the increased STI1 levels found in AD and PD does not improve or compensate for abnormal chaperone function, or improve extracellular signaling; it may further imbalance the chaperone machinery and is deleterious. Indeed, in a transcriptome analysis in AD brains, the *STIP1* gene was found to be one of the key nodes regulating abnormal proteostatic stress [130].

Although chaperones are thought in general to protect against protein misfolding [20, 32, 73], there are several examples in which their activity or imbalance help to preserve misfolded and toxic proteins, such as hyperphosphorylated tau [44, 67, 84], and thus potentially increase toxicity. Inhibitors of Hsp90 increase the degradation of misfolded tau, suggesting that this chaperone stabilizes toxic tau [39]. Notably, Hsp90 co-chaperones FKBP51 and Aha1 stabilize toxic tau conformers and augment tau aggregation in vitro and in vivo, respectively [18, 36, 66].

Augmented proteostatic stress in proteinopathies causes a reorganization of chaperome connectivity leading to the formation of abnormal complexes known as epichaperomes [64]. This abnormal connectivity and formation of higher molecular weight assemblies is toxic to neurons [64] and affects numerous client proteins [58, 102]. It is not clear whether the increased accumulation of chaperones and co-chaperones in insoluble fractions we observed due to α-synuclein misfolding represents the epichaperome, but interference with STI1 collapses the epichaperome [30, 102], consistent with our observations that a hypomorphic STI1 allele in mice protected against α-synuclein accumulation and toxicity. The localization of STI1 and Hsp90 with α-synuclein inclusions is also consistent with previous findings of STI1 being present in early Lewy body-like structures [86] and Hsp90 being the most abundant chaperone of Lewy bodies [121]. These observations suggest that Hsp90 and STI1 may get selectively entrapped in protein deposits because of their roles in regulating α-synuclein activity and physiological functions. Hence, misfolding of α-synuclein disturbs chaperone function, which is not compensated by increased levels of STI1 and Hsp90, but rather the abnormal chaperome is corrected by decreasing the levels/activity of STI1.

Our data reveal that STI1 binding to the C-terminal region of mutated misfolded α-synuclein facilitates its phosphorylation at S129. This process is involved in the toxicity of α-synuclein, which we functionally evaluated via motor behavior deficits, high-level cognitive function, and atrophy of specific brain regions. Of note, our analysis of reversal learning using touchscreens was much more sensitive than motor function assessments in M83+/+ mice. Executive dysfunction and deficits in reversal learning and behavioral flexibility are cognitive alterations found in human synucleinopathies [41, 111, 118]; therefore, the touchscreen experiments and MR imaging shown here provide a much more translatable readout from mice to humans [94]. α-Synuclein toxicity seems to selectively interfere with reversal learning, as we were unable to detect attentional deficits in these mice in early ages, despite pattern of atrophy in the brain that may suggest other cognitive deficits. Further experiments using batteries of translatable touchscreen tests may provide other relevant endpoints to investigate cognition in models of synucleinopathies.

STI1 regulates multiple other co-chaperones and expression of one ΔTPR1 hypomorphic allele can also decrease the levels of co-chaperones such as cyclophilin A (CypA), which has recently been shown to favor α-synuclein misfolding [48], providing a potential complementary mechanism by which α-synuclein can be regulated. Because STI1 in yeast is a co-chaperone that preferentially regulates kinases [17], we cannot exclude the possibility that it may also directly regulate kinase activity, including PLK3. As a limitation, STI1 mouse models cannot distinguish between direct effects of STI1 or its role in the chaperone machinery. Mice with increased STI1 levels also present increased levels of Hsp90ß [13], whereas the phenotypes of hypomorphic mice are consistent with loss of STI1 function, including disruption of Hsp90 chaperone activity [75]. Future experiments specifically modulating STI1 levels (i.e., by adeno-associated virus) or using conditional knockdown mice could be employed to differentiate the STI1 vs. Hsp90 specific effects, as well as developmental confounds. Other limitations include the variability of disease onset in M83+/+ mice (from 8 to 16 months of age) [56], and the severe development of inclusions in PFF-injected mice. Moreover, as an important limitation, all our biochemical and behavioral studies, given their exploratory nature and our initial transcriptome analysis, were performed in male mice. Future experiments will need to determine the roles of STI1 and Hsp90, if any, in female mice regarding α-synuclein misfolding and aggregation. Our results are consistent with a role of the chaperone machinery, particularly Hsp90 and STI1, in forming complexes with α-synuclein that are involved in potential toxic functions, or stabilization of conformers on the pathway to misfolding and protein aggregation. Targeting the interaction of STI1 with α-synuclein may therefore provide ways to mitigate α-synuclein misfolding and toxicity.

## Supplementary Information

Below is the link to the electronic supplementary material.

Supplementary Figure 1: Characterization of the interactions between STI1 and α-synuclein by NMR titration. a. 600 MHz 1H-15N HSQC spectrum of 100 µM α-synuclein (in 20 mM Hepes, 50 mM NaCl, pH 7.2) in the absence (black) and the presence (green) of 200 µM of STI1 TPR1 domain. b. 600 MHz 1H-15N HSQC spectrum of 100 µM α-synuclein (in 20 mM Hepes, 50 mM NaCl, pH 7.2) in the absence (black) and the presence (cyan) of 200 µM of STI1 TPR2B domain. c. NMR titration of unlabeled STI1 to 15N-labeled α-synuclein. The concentration of α-synuclein was kept constant at 100 µM (estimated by Lowry assay) while the concentrations of STI1 varied from 0 µM to 200 µM in increments of 25 µM (estimated by Lowry assay). The KD value was determined based on the titration data of D119, D121, and E137, the three residues that displayed the largest chemical shift changes upon the addition of STI1. Protein concentrations determined by amino acid analysis were used in fitting the KD value. d. NMR titration of unlabeled TPR2A to 15N-labelled α-synuclein. The concentration of α-synuclein was kept constant at 100 µM (estimated by Lowry assay) while the concentrations of TPR2A varied from 0 µM to 200 µM (estimated by Lowry assay) in increments of 25 µM. The KD value was determined based on the titration data of D119, D121, and E137, the three residues that displayed the largest chemical shift changes upon the addition of TPR2A. Protein concentrations determined by amino acid analysis were used in fitting the KD value. e. Overlay of 1H-15N HSQC spectra of 100 µM 15N-labelled α-synuclein (black), 100 µM 15N-labelled α-synuclein + 100 µM TPR2A (red), and 100 µM 15N-labeled α-synuclein + 100 µM TPR2A + 100 µM Hsp90 pentapeptide (green). Insets in panels c, d, and e highlight the chemical shift perturbations of several selected residues upon addition of binding ligands.
Supplementary Figure 2. Characterization of WT human α-synuclein PFFs and pathology induced in M83+/- mice. a. Electron microscopy images of unsonicated and sonicated PFFs. Sonication of α-syn fibrils results in fibrils of smaller length following aggregation, leading to α-syn PFFs with a length <100 nm. b. Quantification of sonicated PFF diameter by Dynamic Light Scattering (DLS). The mean diameters for batches of PFF used in mouse injections, as quantified by DLS in three separate measurements per batch. c. Whole section tiled imaging of nuclear marker and psyn129 labelling in M83+/- PBS and PFF mice, images acquired at 20x magnification near coordinates for injection. Scale bar = 1 mm. d-f. Higher resolution imaging of colocalization analyzes in the ipsilateral cortex of M83+/- PFF injected mice. d. Colocalization of Ubiquitin with psyn129. e. Colocalization of Hsp90 with psyn129. f. Colocalization of STI1 with psyn129. g & h. Labeling for psyn129 and Amytracker in the piriform pyramidal layer of M83+/- PBS and M83+/- PFF injected mice. psyn129 partially colocalizes with misfolded and potentially aggregated proteins. Arrows indicate regions with high co-labelling between psyn129 and Amytracker, whereas arrowheads indicate cells with predominant Amytracker labeling, but little psyn129. Images acquired at 63X magnification and post-processed 2X zoom factor (zoom factor only applied to d-f). Scale bar = 25 µm. g. As expected, little to no staining is observed in M83+/- PBS injected mice. h. pathology labelling is abundant in M83+/- PFF injected mice.
Supplementary Figure 3. a. Representative immunoblots of panHsp90, Hsp70, STI1, pSer129 α-synuclein (psyn129), human α-synuclein, and total (mouse and human) α-synuclein in RIPA lysates from cortex of WT and M83+/- mice (no inoculations with PFFs) b. Densitometric quantification of Hsp90 (t(7)= 2.605, p=0.0352). c. Densitometric quantification of constitutive Hsp70 (t(7) = 2.869, p=0.0240). d. Densitometric quantification of STI1 (t(7) = 3.282, p=0.0134). e. Representative immunoblots from 2% SDS and 4 M Urea extraction in cortical lysates for panHsp90, Hsp70, STI1, psyn129, human α-synuclein, and total (mouse and human) α-synuclein. f. Densitometric quantification of Hsp90 (t(7) = 0.563, p=0.591), g. Densitometric quantification of Hsp70 (t(7) = 0.361, p = 0.729), h. Densitometric quantification of STI1 (t(7) = 1.072, p=0.320). i. Representative immunolabelling for psyn129 (red), and nuclear marker (blue) in the ipsilateral ACC j. Representative immunolabelling for psyn129 (red), and nuclear marker (blue) in the dentate gyrus of M83+/- and M83+/-:TgA PFF injected mice. 40X images, 2X zoom, with scale bars = 25 µm. k. Immunoblots from C57BL/6 non-M83 transgenic WT and TgA mouse cortical lysates. l. Densitometric quantification of total mouse α-synuclein normalized to actin loading control (t(8) = 0.6609, p=0.5272). Data represented as mean ± SEM and analyzed using unpaired t-test.
Supplementary Figure 4. Quantification of the percent area immunolabeled with psyn129 in the a. ipsilateral hemisphere (t(9) = 3.006, p=0.015) and b. contralateral hemisphere (t(9) = 1.769, p=0.1107). Imaged at 20X magnification. (N=6 for M83+/- PFF mice, N=5 for M83+/-::ΔTPR1 PFF injected mice, images captured near plane of injection site. Representative image in Fig. 3a. Data represented as mean ± SEM and analyzed via unpaired t-test. c. Representative microscopy immunofluorescence images (Leica Thunder) in the ipsilateral caudate putamen (region of injection) with nuclear marker (blue), GFAP (astrocytes, green), psyn129 (red) and d. quantification of percent area with psyn129 immunoreactivity in the ipsilateral caudate putamen (Mann Whitney non-parametric test, p=0.0079). N=5/genotype. e. Representative microscopy images of the ipsilateral ACC of M83+/- (N=6) and M83+/-::ΔTPR1 PFF-injected mice (N=5). f. quantification of percent area with psyn129 immunoreactivity in the ipsilateral ACC (t(8) = 2.616, p=0.0308). g. Representative images in the ventrolateral cortical area, the piriform pyramidal layers (ipsilateral, PPLs) within M83+/- (N=6) and M83+/-::ΔTPR1 PFF-injected mice (N=5). h. Quantification of percent area with psyn129 immunoreactivity in the ipsilateral PPLs (t(8) = 2.911, p=0.0196). i. Representative images of the ipsilateral hippocampus (image set within the dentate gyrus) and j. respective quantification of percent area with psyn129 immunoreactivity in the whole ipsilateral hippocampus (t(8)= 2.288, p=0.0514). N=5/genotype. Scale bars are 50 µm and images taken at 40x with post processing at 2X zoom. Images used for quantification were tiling images of the whole section, taken at 20X. Individual circles (M83+/- PFF) and filled triangles (M83+/-:ΔTPR1 PFF) represent individual animals. Data represented as mean ± SEM. Data were analyzed with unpaired t-test and assessed for normality using Shapiro-Wilk test. If data were non-normal, Mann Whitney non-parametric test was used. *p<0.05, **p<0.01.              
Supplementary Figure 5. Qualitative representative pattern of staining of human pSer129 (MJF-R13) antibody in M83+/- PFF injected mutant STI1 mice. M83+/-, M83+/-:TgA and M83+/-::ΔTPR1 PFF mice immunolabelling for human pSer129 (red) in the a. Anterior Cingulate Cortex (ACC) and b. piriform pyramidal layers (PPLs). Both are regions with abundant pathology. Images were acquired at 40X magnification. Scale bars are 50 µm.
Supplementary Figure 6. Ubiquitin, Hsp90, and STI1 cytoplasmic staining co-labels with psyn129 aggregates in M83+/- PFF injected mice. Representative images of inclusions in M83+/- and M83+/-:ΔTPR1-PFF injected mice. a. partial colocalization between psyn129 and ubiquitin in ipsilateral piriform pyramidal layer (PPL) b. partial colocalization between psyn129 and ubiquitin in the ipsilateral Dentate gyrus. Likewise, Hsp90 and psyn129 partial colocalization in c. PPL. d. partial colocalization between psyn129 and Hsp90 in the Dentate gyrus. e. partial colocalization between psyn129 and STI1 in the PPL. f. partial colocalization between psyn129 and STI1 in the Dentate gyrus. Arrows illustrate areas where co-localization was observed. Images were taken at 63X magnification and post processed to 2X zoom. Scale bars are 25 µm for all images. g. Representative immunoblot and h. Densitometric quantification of mouse α-synuclein protein levels in WT and ΔTPR1 mouse tissues with 50% less STI1 (ΔHET) or 80% less STI1 (ΔTPR1) (N=4 mice/genotype). Data are mean ± SEM and analyzed using One-way ANOVA with no significant different between genotypes (F(2,9) = 0.494, p=0.63).
Supplementary Figure 7. NMR titration of unlabeled TPR2A to 15N-labelled psyn129. a. The concentration of psyn129 was kept constant at 100 µM (estimated by Lowry assay), while the concentrations of TPR2A varied from 0 µM to 200 µM (estimated by Lowry assay) in increments of 25 µM. The KD value was determined based on the titration data of D119, D121, and E137, the three residues that displayed the largest chemical shift changes upon the addition of TPR2A. Protein concentrations determined by amino acid analysis were used to in fitting the KD value. Insets in the figure highlight the chemical shift perturbations of several selected residues upon addition of TPR2A. The dashed square indicates the location of the S129 peak of non-phosphorylated α-synuclein obtained in the other titrations. The lack of signal in that position indicates that α-synuclein was almost completely phosphorylated.
Supplementary Figure 8. Five-choice serial reaction time task (5-CSRTT) used to measure attention in M83 mice and their non-transgenic controls at 8 months old. a. Accuracy during probe trial sessions. Repeated measures two-way ANOVA (Stimulus duration: F(2.090, 39.71) = 24.62, p < 0.0001; Genotype: F(1, 20) = 3.263, p = 0.0859; Interaction: F(3, 57) = 1.628, p = 0.1930). b. Rate of omission. Repeated measures two-way ANOVA (Stimulus duration: F(2.803, 53.25) = 24.23, p < 0.0001; Genotype: F(1, 20) =0.2109, p = 0.6510; Interaction: F(3, 57) = 0.8955, p = 0.4491). c. Number of Premature responses. Repeated measures two-way ANOVA (Stimulus duration: F(2.679, 50.89) = 3.310, p = 0.0318; Genotype: F(1, 20) = 0.6922, p = 0.4152; Interaction: F(3, 57) = 0.4390, p = 0.7260). d. Correct touch latency (s). Repeated measures two-way ANOVA (Stimulus duration: F(2.506, 58.48) = 9.142, p < 0.0001; Genotype: F(1, 25) = 5.540e-009, p = 0.9999; Interaction: F(3, 70) = 0.6604, p = 0.5791). e. Reward collection latency (s). Repeated measures two-way ANOVA (Stimulus duration: F(2.558, 48.60) = 1.609, p = 0.2046; Genotype: F(1, 20) = 0.1751, p = 0.6801; Interaction: F(3, 57) = 2.969, p = 0.0393). f. Number of Perseverative responses. Repeated measures two-way ANOVA (Stimulus duration: F(1.219, 23.16) = 1.725, p = 0.2037; Genotype: F(1, 20) = 0.01583, p = 0.9011; Interaction: F(3, 57) = 0.5263, p = 0.6660). g. Number of trials completed. Repeated measures two-way ANOVA (Stimulus duration: F(1.732, 46.19) = 0.5924, p = 0.2037; Genotype: F(1, 80) = 0.001581, p = 0.9684; Interaction: F(3, 80) = 1.235, p = 0.3025). Attention was measured, in M83+/+:STI1WT mice (n=10) and their non-transgenic WT controls (n=12) at 8 months old, using the 5-choice Serial Reaction Time Testing (5-CSRTT) performance probe trials with 1.5-, 1.0-, 0.8- and 0.6-second stimulus durations. Results are expressed as mean ± SEM. Asterisks indicate statistical differences between groups: *p<0.05. Individual unfilled black circles (WT), dark blue circles (M83+/+:STI1WT), half-filled pink triangles (M83+/+:ΔHET) represent individual animals.
Supplementary Figure 9. Additional parameters for cognitive behavior measured in the PVD-R task. a. Timeline for the testing mice on the PVD-R test. b. Correct touch latency (s). Repeated measures two-way ANOVA (Session: F(3.551, 194.7) = 30.14, p < 0.0001; Genotype: F(2, 55) = 10.46, p = 0.0001; Interaction: F(22, 603) = 5.527, p < 0.0001, Adjusted for multiple comparisons with Tukey's multiple comparisons test: WT vs. M83+/+: STI1WT p= 0.5341, WT vs. M83+/+:ΔHET p < 0.0001, M83+/+:STI1WT vs. M83+/+:ΔHET p < 0.0001). c. Reward collection latency (s). Repeated measures two-way ANOVA (Session: F(7.529, 407.3) = 2.737, p= 0.0071; Genotype: F(2, 55) = 12.62, p 0.0001; Interaction: F(22, 595)=1.982, p=0.0050, adjusted for multiple comparisons with Tukey's multiple comparisons test: WT vs. M83+/+:STI1WT p < 0.0001, WT vs. M83+/+:ΔHET p < 0.0001, M83+/+:STI1WT vs. M83+/+:ΔHET p < 0.0001). d. Number of trials completed in the PVD reversal learning (PVD-R) task. Repeated measures two-way ANOVA (Session: F(3.479, 185.7) = 7.461, p < 0.0001; Genotype: F(2, 55) = 3.001, p=0.0579; Interaction: F(22, 587) = 1.749, p = 0.0188, adjusted for multiple comparisons with Tukey's multiple comparisons test: WT vs. M83+/+:STI1WT p = 0.5299, WT vs. M83+/+:ΔHET p = 0.3415, M83+/+:STI1WT vs. M83+/+:ΔHET p = 0.0407).). WT control mice (n=20), M83+/+: STI1WT mice (n=20) and M83+/+:ΔHET mice (n=18). Marble (S-) and fan (S+) stimuli were used during the PVD-R acquisition phase. Parameters were measured across baseline days 1 and 2 (B1, B2) and reversal days 1 to 10 (R1-R10). Results are expressed as mean ± SEM. Asterisks indicate statistical differences between groups: *p<0.05, **p<0.001, ***p<0.0001. Individual unfilled black circles (WT), dark blue circles (M83+/+:STI1WT), half-filled pink triangles (M83+/+:ΔHET) represent individual animals.
Supplementary Table 1: List of anonymized control and PD samples analyzed using RNAseq per age group and brain region, Lewy body pathology severity and length of disease.
Supplementary Table 2: Healthy control brain tissue information from the GTEX database used for RNA seq analyses.
Supplementary Table 3: A full list of the anatomical regions segmented in the modified Allen brain atlas.
Supplementary Table 4: Correlations, p value and FDR information for Transcriptomic Analysis.
Supplementary Table 5: Volumetric statistical results for each structure in the modified Allen Brain Atlas in terms of standardised betas, t-, p- and q- values for each of the comparisons performed (M83+/+:STI1WT versus WT mice, and M83+/+:ΔHET versus M83+/+:STI1WT). Supplementary file 1 (PDF 35970 KB)

## Data Availability

Data are available in the following repositories: **MRI data** https://doi.org/10.5281/zenodo.6620797; **Motor behavior and MRI data – MouseBytes + repository** https://mousebytes.ca/comp-edit?repolinkguid=f2d0a4f6-f61d-40ea-bf64-fc3ee06ede5d**. PVD Cohort 2-** https://mousebytes.ca/data-link?linkguid=298fbf10-e7c6-484e-85e9-2c2199e1ea52**. PVD Cohort 1-** https://mousebytes.ca/data-link?linkguid=bddd03a1-43a3-4834-a3a2-303738551046**. 5-choice test-** https://mousebytes.ca/data-link?linkguid=61d7dfa4-a749-43e5-9822-93bebab1a673. **Biochemical data and quantifications-** https://figshare.com/projects/Stress-inducible_phosphoprotein_1_HOP_STI1_STIP1_regulates_the_accumulation_and_toxicity_of_-synuclein_in_vivo/140915.
