# Supplementary material for: Stress-inducible phosphoprotein 1 (HOP/STI1/STIP1) regulates the accumulation and toxicity of α-synuclein in vivo
Source: Acta Neuropathol. 2022 Sep 19;144(5):881–910. doi: 10.1007/s00401-022-02491-8 (PMC9547791; doi:10.1007/s00401-022-02491-8)
Supplement: Supplementary file 1 — Supplementary Figure 1: Characterization of the interactions between STI1 and α-synuclein by NMR titration. a. 600 MHz 1H-15N HSQC spectrum of 100 µM α-synuclein (in 20 mM Hepes, 50 mM NaCl, pH 7.2) in the absence (black) and the presence (green) of 200 µM of STI1 TPR1 domain. b. 600 MHz 1H-15N HSQC spectrum of 100 µM α-synuclein (in 20 mM Hepes, 50 mM NaCl, pH 7.2) in the absence (black) and the presence (cyan) of 200 µM of STI1 TPR2B domain. c. NMR titration of unlabeled STI1 to 15N-labeled α-synuclein. The concentration of α-synuclein was kept constant at 100 µM (estimated by Lowry assay) while the concentrations of STI1 varied from 0 µM to 200 µM in increments of 25 µM (estimated by Lowry assay). The KD value was determined based on the titration data of D119, D121, and E137, the three residues that displayed the largest chemical shift changes upon the addition of STI1. Protein concentrations determined by amino acid analysis were used in fitting the KD value. d. NMR titration of unlabeled TPR2A to 15N-labelled α-synuclein. The concentration of α-synuclein was kept constant at 100 µM (estimated by Lowry assay) while the concentrations of TPR2A varied from 0 µM to 200 µM (estimated by Lowry assay) in increments of 25 µM. The KD value was determined based on the titration data of D119, D121, and E137, the three residues that displayed the largest chemical shift changes upon the addition of TPR2A. Protein concentrations determined by amino acid analysis were used in fitting the KD value. e. Overlay of 1H-15N HSQC spectra of 100 µM 15N-labelled α-synuclein (black), 100 µM 15N-labelled α-synuclein + 100 µM TPR2A (red), and 100 µM 15N-labeled α-synuclein + 100 µM TPR2A + 100 µM Hsp90 pentapeptide (green). Insets in panels c, d, and e highlight the chemical shift perturbations of several selected residues upon addition of binding ligands. Supplementary Figure 2. Characterization of WT human α-synuclein PFFs and pathology induced in M83+/- mice. a. Electron microscopy i [file 401_2022_2491_MOESM1_ESM.pdf]

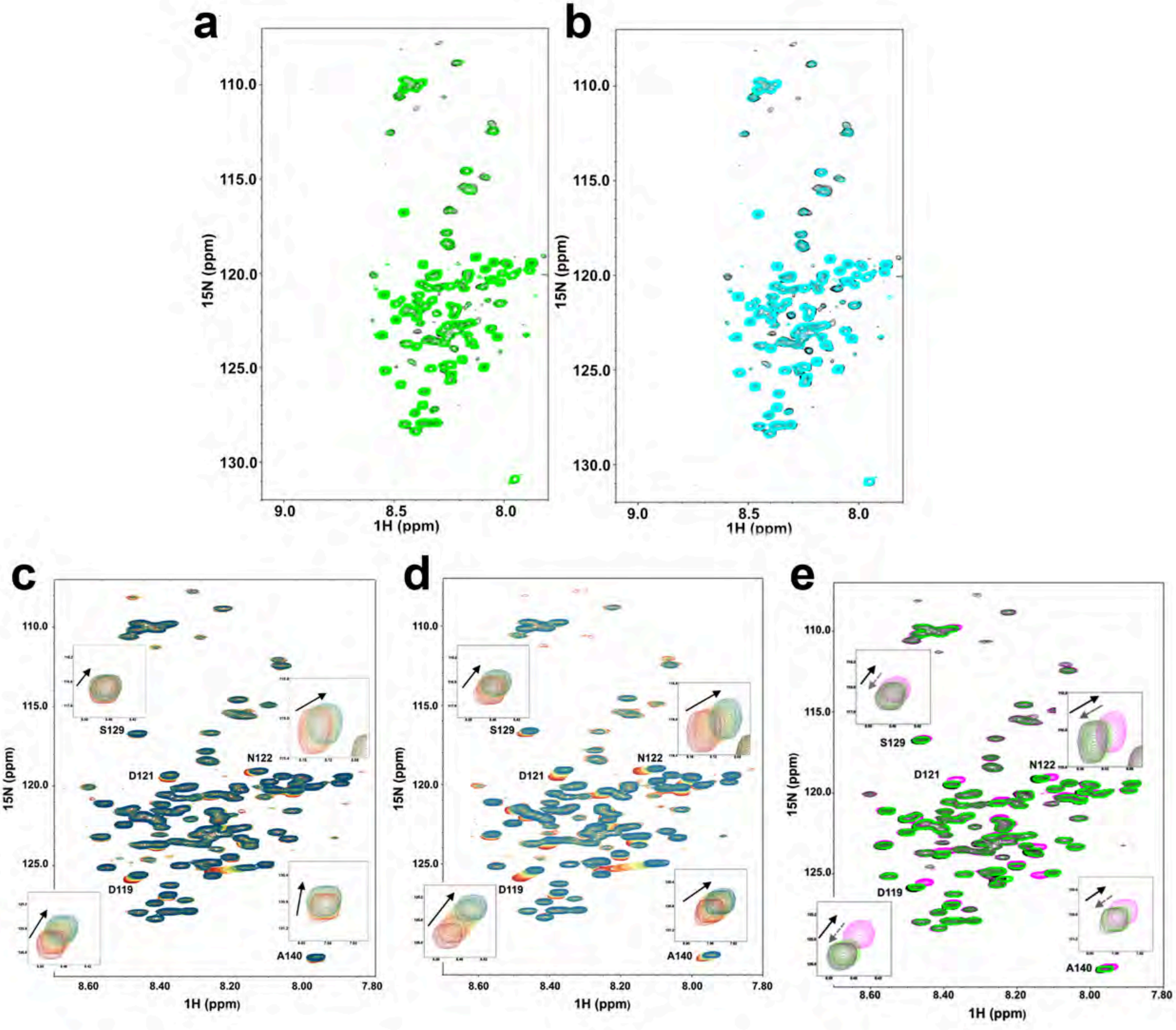
a
b
c
d
e
15N (ppm)
1H (ppm)
110.0
115.0
120.0
125.0
130.0
9.0
8.5
8.0
8.60
8.40
8.20
8.00
7.80
S129
D121
N122
D119
A140

Fig. S1

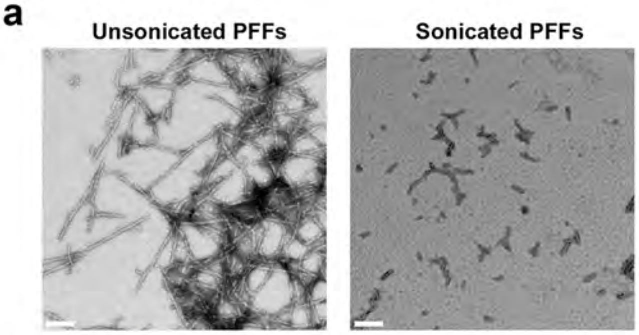

b

PFF Batch #	Species 1 diameter (nm)	% of species in sample
1	82.97 ± 39.08	100
2	69.83 ± 44.42	100
3	92.84 ± 45.59	100
4	96.21 ± 47.02	100

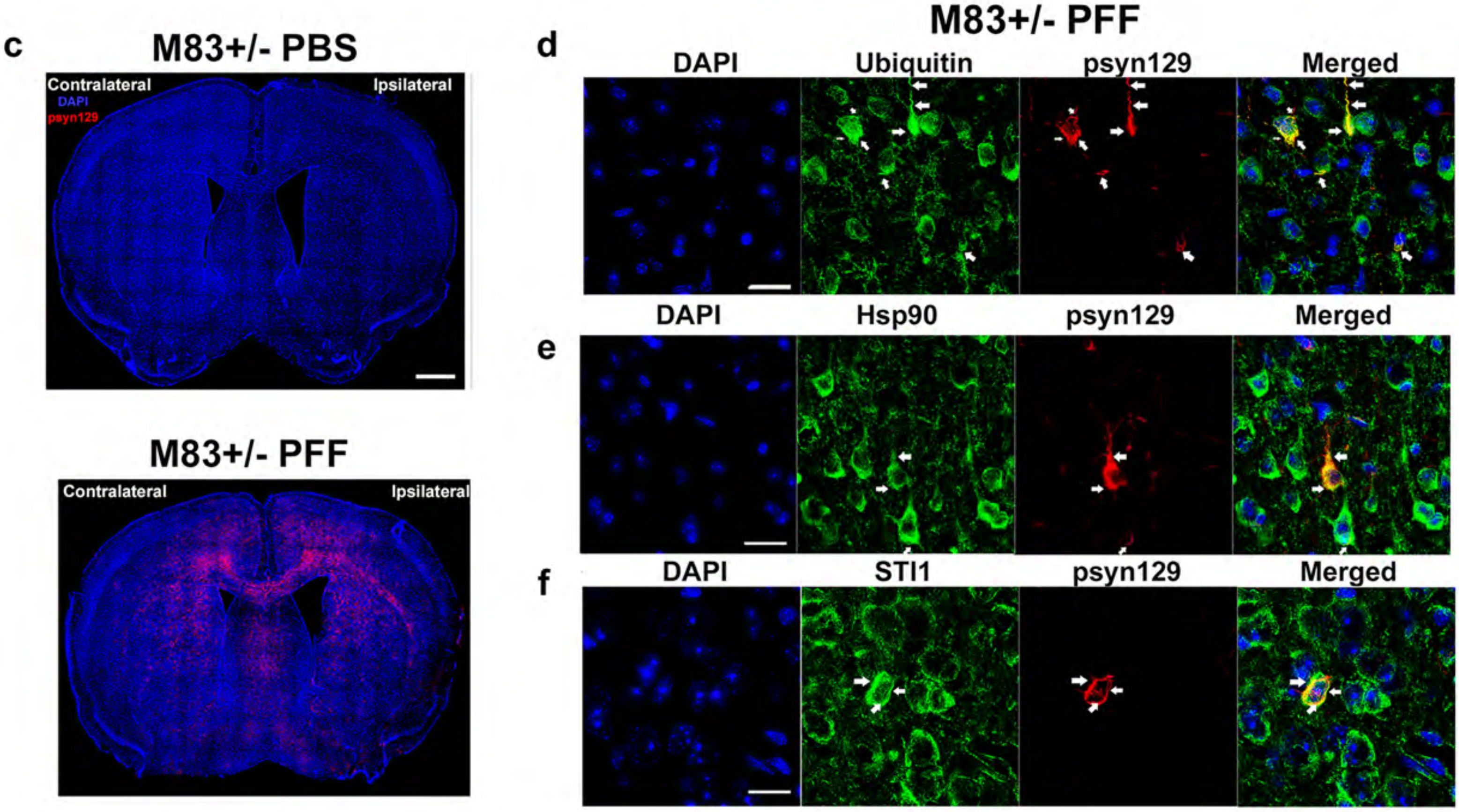

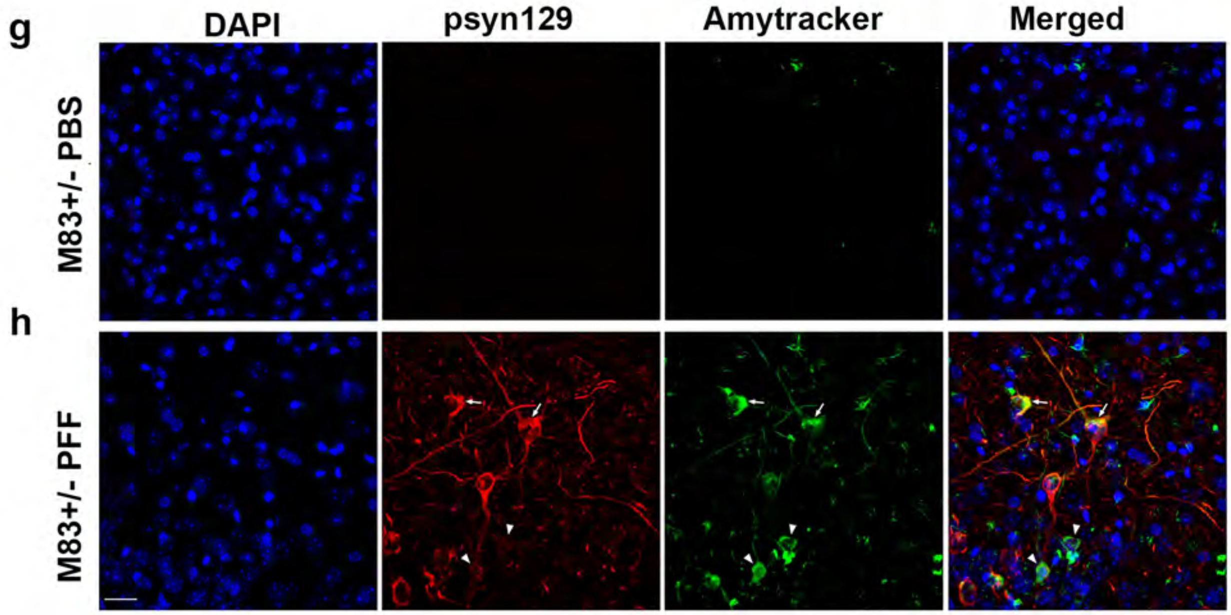

Fig. S2

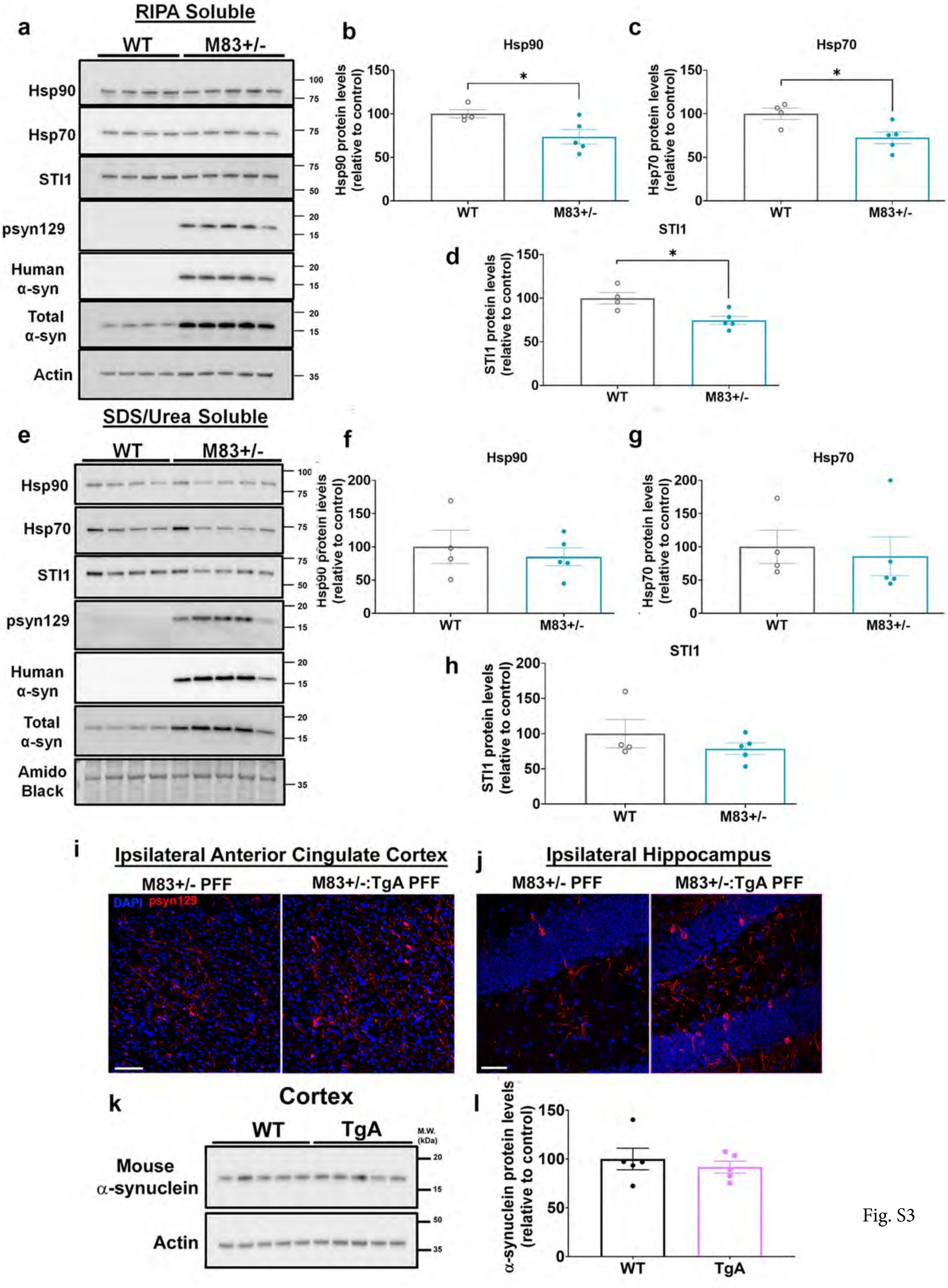

a
RIPA Soluble
WT
M83+/-
Hsp90
Hsp70
STI1
psyn129
Human α-syn
Total α-syn
Actin
b
Hsp90
Hsp90 protein levels (relative to control)
c
Hsp70
Hsp70 protein levels (relative to control)
d
STI1
STI1 protein levels (relative to control)
e
SDS/Urea Soluble
Amido Black
f
g
h
i
Ipsilateral Anterior Cingulate Cortex
M83+/- PFF
M83+/-:TgA PFF
DAPI psyn129
j
Ipsilateral Hippocampus
k
Cortex
WT
TgA
Mouse α-synuclein
Actin
M.W. (kDa)
l
α-synuclein protein levels (relative to control)

Fig. S3

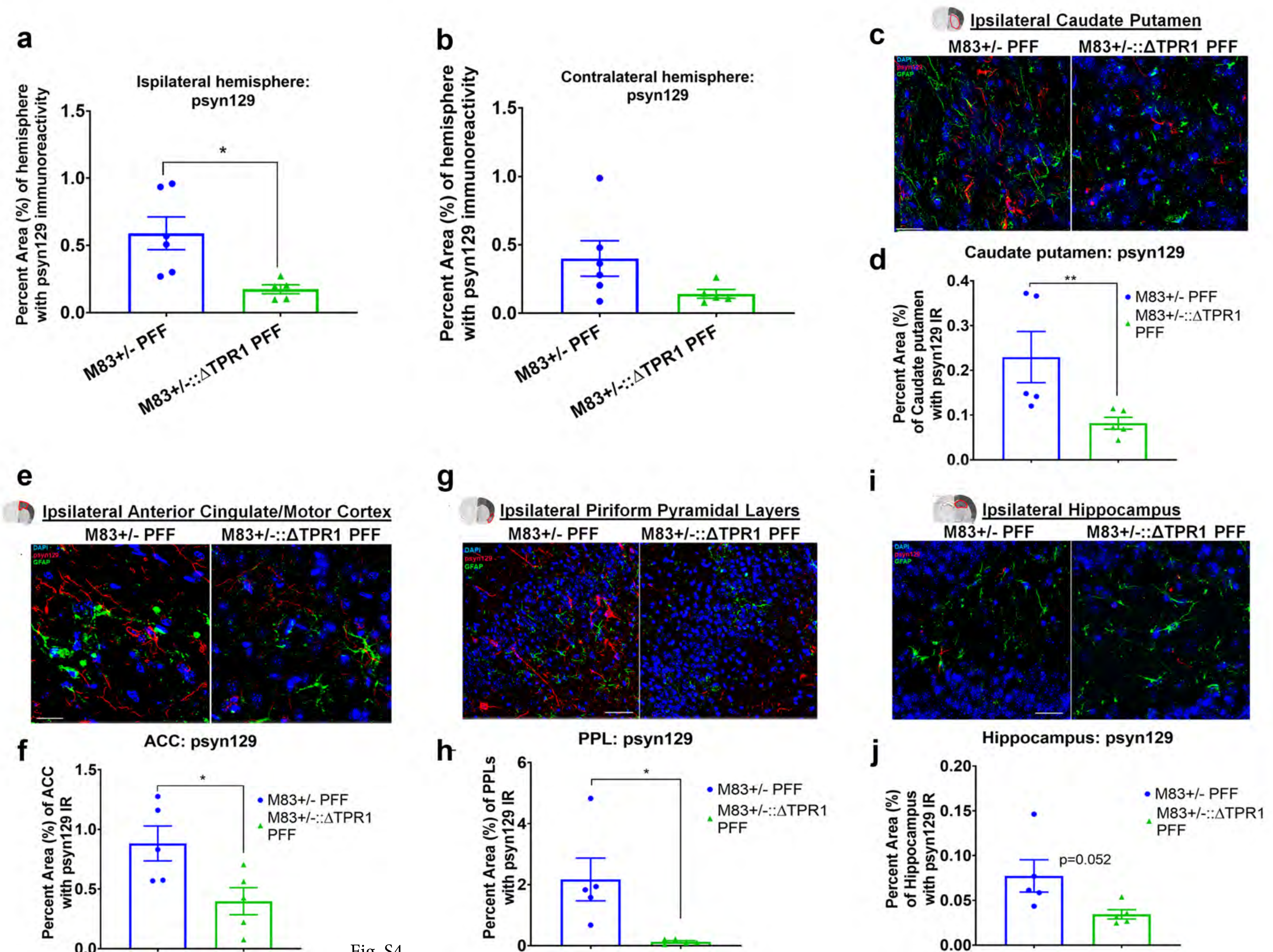
a
Ispilateral hemisphere: psyn129
Percent Area (%) of hemisphere with psyn129 immunoreactivity
M83+/- PFF
M83+/-::ΔTPR1 PFF
b
Contralateral hemisphere: psyn129
Percent Area (%) of hemisphere with psyn129 immunoreactivity
c
Ipsilateral Caudate Putamen
M83+/- PFF
M83+/-::ΔTPR1 PFF
d
Caudate putamen: psyn129
Percent Area (%) of Caudate putamen with psyn129 IR
e
Ipsilateral Anterior Cingulate/Motor Cortex
f
ACC: psyn129
Percent Area (%) of ACC with psyn129 IR
g
Ipsilateral Piriform Pyramidal Layers
h
PPL: psyn129
Percent Area (%) of PPLs with psyn129 IR
i
Ipsilateral Hippocampus
j
Hippocampus: psyn129
Percent Area (%) of Hippocampus with psyn129 IR
p=0.052

Fig. S4

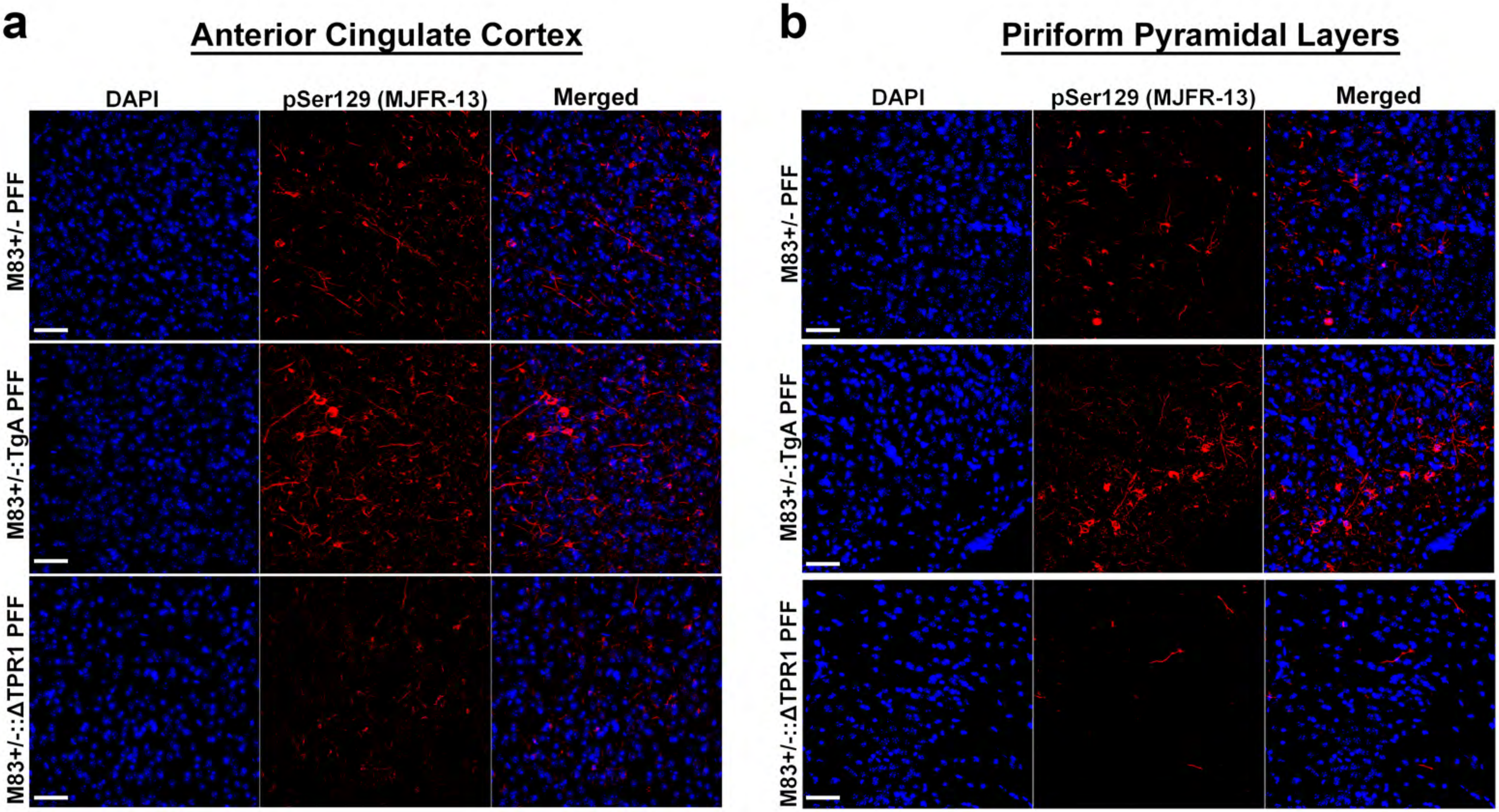
a
Anterior Cingulate Cortex
DAPI
pSer129 (MJFR-13)
Merged
M83+/- PFF
M83+/-:TgA PFF
M83+/-::ΔTPR1 PFF
b
Piriform Pyramidal Layers
DAPI
pSer129 (MJFR-13)
Merged
M83+/- PFF
M83+/-:TgA PFF
M83+/-::ΔTPR1 PFF

Fig. S5

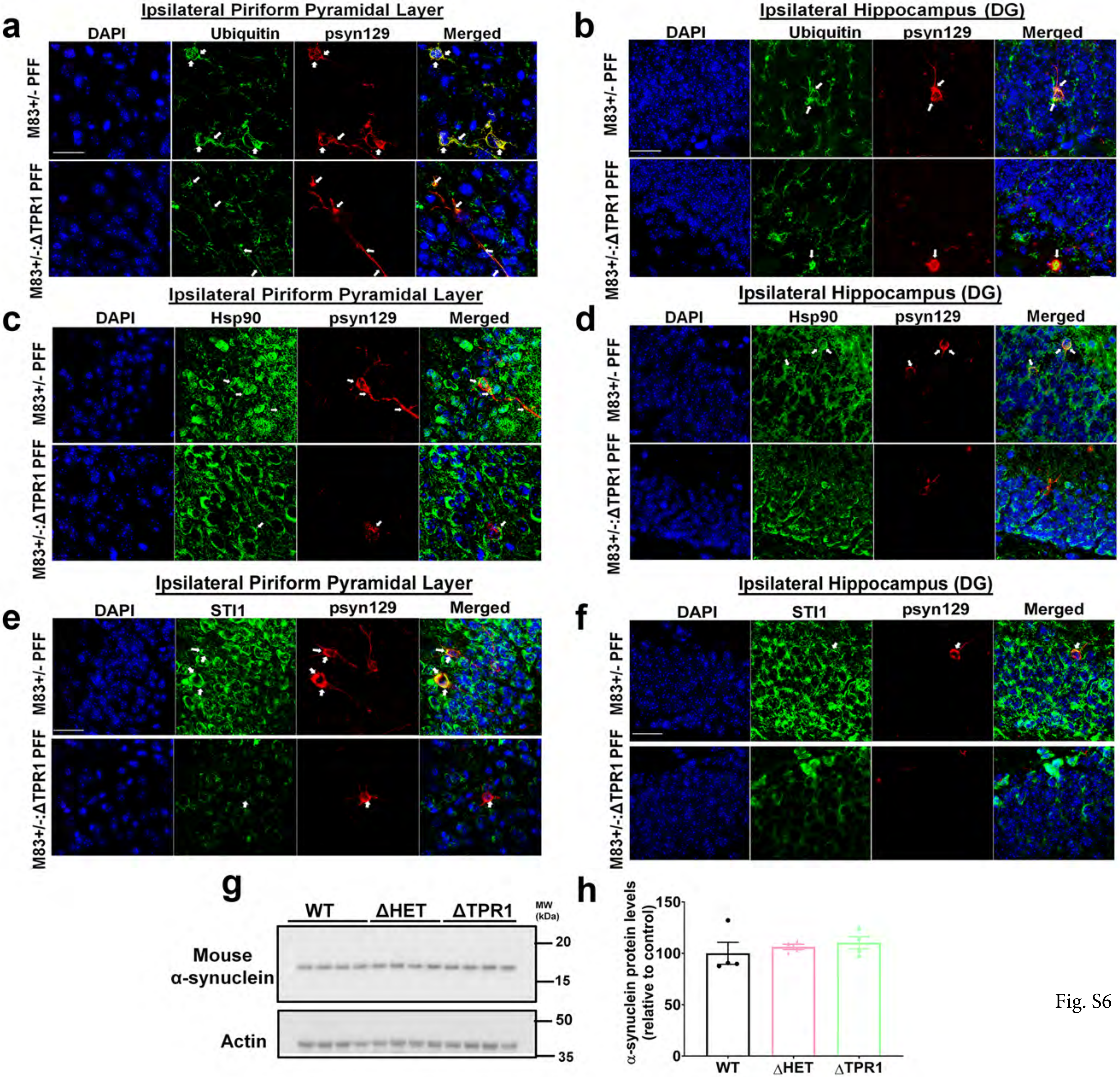
a
Ipsilateral Piriform Pyramidal Layer
DAPI
Ubiquitin
psyn129
Merged
M83+/- PFF
M83+/-:ΔTPR1 PFF
b
Ipsilateral Hippocampus (DG)
DAPI
Ubiquitin
psyn129
Merged
M83+/- PFF
M83+/-:ΔTPR1 PFF
c
Ipsilateral Piriform Pyramidal Layer
DAPI
Hsp90
psyn129
Merged
M83+/- PFF
M83+/-:ΔTPR1 PFF
d
Ipsilateral Hippocampus (DG)
DAPI
Hsp90
psyn129
Merged
M83+/- PFF
M83+/-:ΔTPR1 PFF
e
Ipsilateral Piriform Pyramidal Layer
DAPI
STI1
psyn129
Merged
M83+/- PFF
M83+/-:ΔTPR1 PFF
f
Ipsilateral Hippocampus (DG)
DAPI
STI1
psyn129
Merged
M83+/- PFF
M83+/-:ΔTPR1 PFF
g
WT
ΔHET
ΔTPR1
MW (kDa)
Mouse α-synuclein
Actin
20
15
50
35
h
α-synuclein protein levels (relative to control)
150
100
50
0
WT
ΔHET
ΔTPR1

Fig. S6

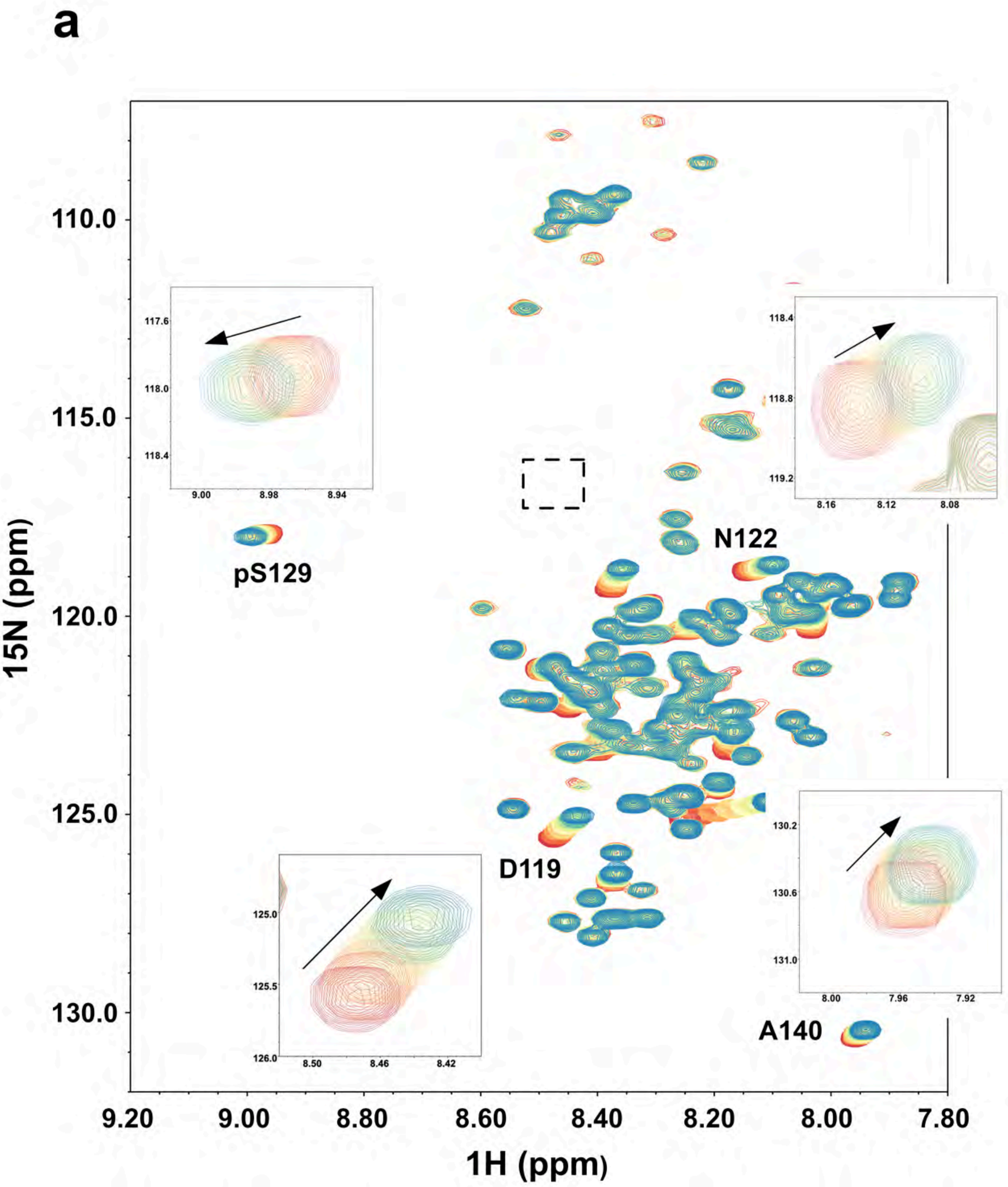

a
110.0
115.0
120.0
125.0
130.0
15N (ppm)
9.20
9.00
8.80
8.60
8.40
8.20
8.00
7.80
1H (ppm)
pS129
N122
D119
A140
117.6
118.0
118.4
9.00
8.98
8.94
118.4
118.8
119.2
8.16
8.12
8.08
125.0
125.5
126.0
8.50
8.46
8.42
130.2
130.6
131.0
8.00
7.96
7.92

Fig. S7

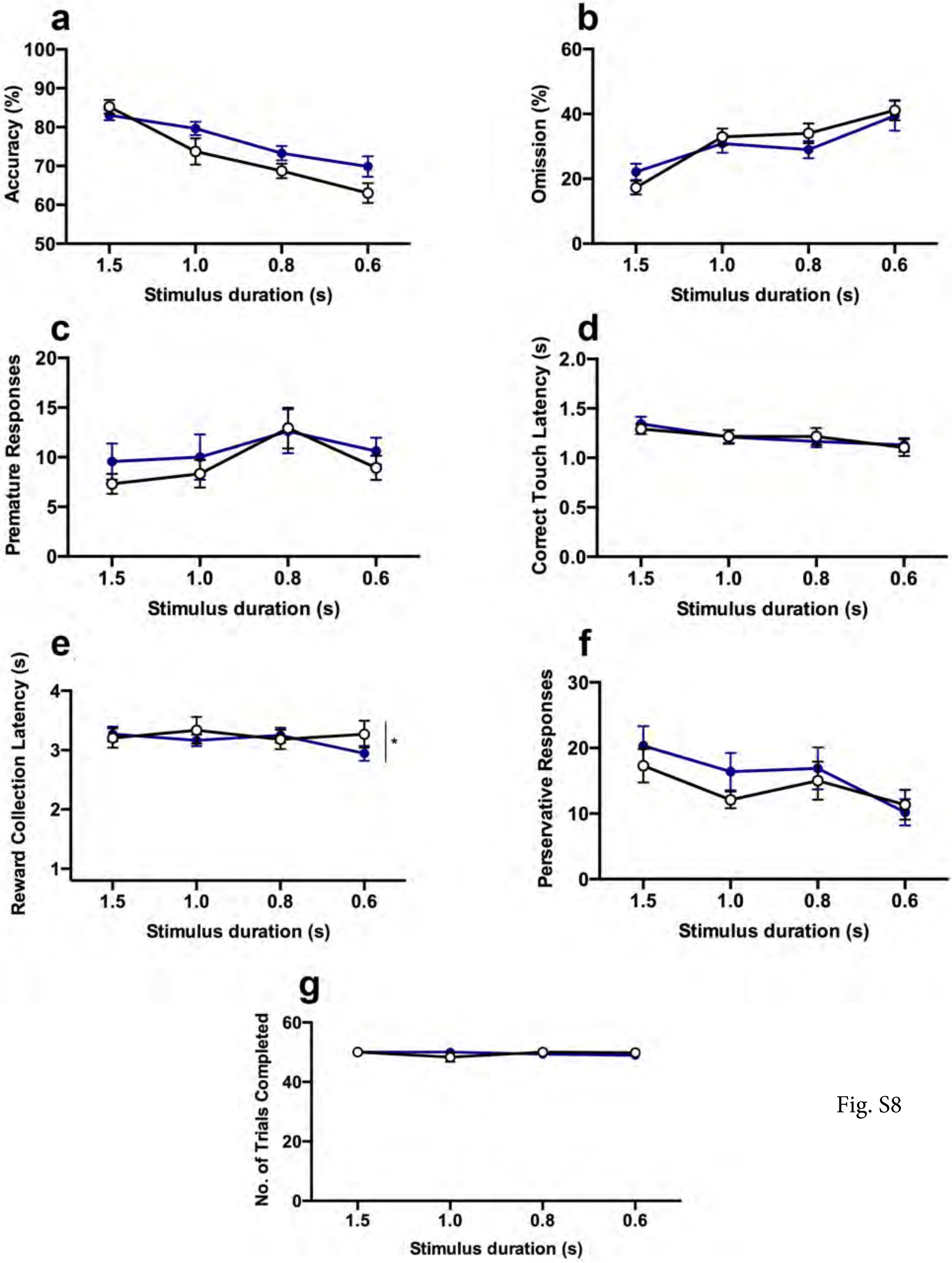

a
Accuracy (%)
100
90
80
70
60
50
1.5
1.0
0.8
0.6
Stimulus duration (s)
b
Omission (%)
60
40
20
0
Stimulus duration (s)
c
Premature Responses
20
15
10
5
0
Stimulus duration (s)
d
Correct Touch Latency (s)
2.0
1.5
1.0
0.5
0.0
Stimulus duration (s)
e
Reward Collection Latency (s)
4
3
2
1
*
Stimulus duration (s)
f
Perservative Responses
30
20
10
0
Stimulus duration (s)
g
No. of Trials Completed
60
40
20
0
Stimulus duration (s)

Fig. S8

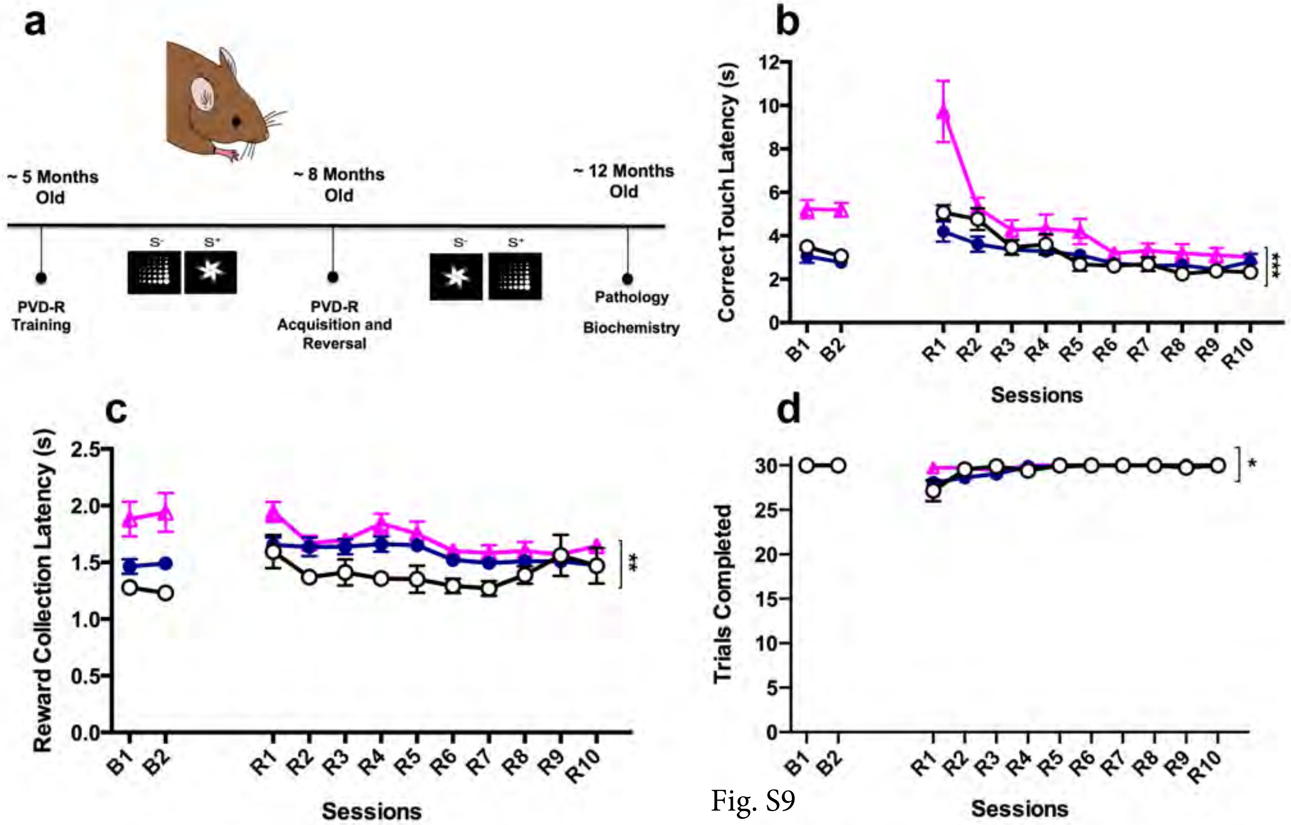

a
~ 5 Months Old
~ 8 Months Old
~ 12 Months Old
S-
S+
S-
S+
PVD-R Training
PVD-R Acquisition and Reversal
Pathology
Biochemistry
b
Correct Touch Latency (s)
Sessions
***
c
Reward Collection Latency (s)
Sessions
**
d
Trials Completed
Sessions
*

Fig. S9

Supplementary Table 1

Sample	BrainID	Region	Condition	LewyBodiesScore	Sex
MTG6	S03/254	MTG	Control	0	M
MTG7	S05/269	MTG	Control	0	M
SN4	S09/039	SN	Control	0	M
Amy13	S09/195	Amygdala	Parkinson	5	M
SN12	S09/195	SN	Parkinson	5	M
MTG1	S10/109	MTG	Control	0	F
MTG16	S11/026	MTG	Parkinson	6	M
SN20	S11/026	SN	Parkinson	6	M
Amy19	S11/062	Amygdala	Parkinson	6	M
MTG17	S11/062	MTG	Parkinson	6	M
SN16	S11/062	SN	Parkinson	6	M
Amy6	S11/090	Amygdala	Control	0	F
MTG2	S11/090	MTG	Control	0	F
SN13	S11/090	SN	Control	0	F
Amy20	S12/011	Amygdala	Parkinson	4	M
MTG18	S12/011	MTG	Parkinson	4	M
SN26	S12/011	SN	Parkinson	4	M
Amy10	S12/013	Amygdala	Parkinson	6	M
MTG19	S12/013	MTG	Parkinson	6	M
Amy3	S12/042	Amygdala	Control	0	F
SN17	S12/042	SN	Control	0	F
Amy1	S12/070	Amygdala	Control	0	M
Amy23	S12/076	Amygdala	Parkinson	6	F
MTG11	S12/076	MTG	Parkinson	6	F
SN3	S12/076	SN	Parkinson	6	F
Amy7	S12/104	Amygdala	Control	0	M
Amy8	S13/011	Amygdala	Control	0	F
MTG4	S13/011	MTG	Control	0	F
SN2	S13/011	SN	Control	0	F
SN6	S13/016	SN	Control	1	M
Amy15	S13/027	Amygdala	Parkinson	6	M
SN28	S13/027	SN	Parkinson	6	M
SN7	S13/041	SN	Parkinson	6	M
Amy5	S13/056	Amygdala	Control	1	M
MTG8	S13/056	MTG	Control	1	M
SN24	S13/056	SN	Control	1	M
Amy18	S13/086	Amygdala	Parkinson	6	M
SN9	S13/086	SN	Parkinson	6	M
MTG12	S13/088	MTG	Parkinson	5	F
SN8	S13/088	SN	Parkinson	5	F
MTG13	S14/009	MTG	Parkinson	6	F
SN21	S14/009	SN	Parkinson	6	F
Amy21	S14/022	Amygdala	Parkinson	6	M
MTG22	S14/022	MTG	Parkinson	6	M

SN11	S14/022	SN	Parkinson	6	M
Amy16	S14/032	Amygdala	Parkinson	6	M
MTG23	S14/032	MTG	Parkinson	6	M
SN23	S14/032	SN	Parkinson	6	M
MTG5	S14/043	MTG	Control	0	F
Amy2	S14/051	Amygdala	Control	0	M
MTG9	S14/051	MTG	Control	0	M
SN27	S14/051	SN	Control	0	M
Amy9	S14/076	Amygdala	Parkinson	6	M
SN15	S14/076	SN	Parkinson	6	M
SN14	S92/001	SN	Control	0	M

Age	AgeGroup	PDLengthYea	RIN
82	old	0	6.6
87	old	0	6.4
78	young	0	8.2
69	young	6	6.8
69	young	6	7.7
60	young	0	6.8
77	young	17	7.4
77	young	17	7.1
65	young	19	7.5
65	young	19	8.2
65	young	19	7.5
85	old	0	7.8
85	old	0	7.1
85	old	0	7.6
73	young	13	7.5
73	young	13	7.8
73	young	13	6.6
76	young	18	6.5
76	young	18	6.2
83	old	0	6.7
83	old	0	7.3
79	young	0	6.1
81	old	33	8.5
81	old	33	7.2
81	old	33	8.3
79	young	0	8
92	old	0	8.7
92	old	0	8
92	old	0	8.3
83	old	0	8.1
76	young	10	7.1
76	young	10	6.5
70	young	11	8.1
95	old	0	7.6
95	old	0	7.1
95	old	0	6.8
81	old	8	7.4
81	old	8	7.9
71	young	14	6.9
71	young	14	8
83	old	22	6.4
83	old	22	7
77	young	18	7.8
77	young	18	7.5

77	young	18	7.9
83	old	15	7.1
83	old	15	6.8
83	old	15	6.9
60	young	0	7.5
92	old	0	6.5
92	old	0	7.1
92	old	0	6.5
78	young	17	6.1
78	young	17	7.6
83	old	0	7.6

Supplementary Table 2

Sample	SubjectID	Region	Sex
GTEX.1117F.3226.SM.5N9CT	GTEX-1117F	Cortex	Female
GTEX.111FC.3126.SM.5GZZ2	GTEX-111FC	Cortex	Male
GTEX.1128S.2726.SM.5H12C	GTEX-1128S	Cortex	Female
GTEX.117XS.3026.SM.5N9CA	GTEX-117XS	Cortex	Male
GTEX.1192X.3126.SM.5N9BY	GTEX-1192X	Cortex	Male
GTEX.11DXY.3226.SM.5GIDE	GTEX-11DXY	Cortex	Male
GTEX.11EI6.3026.SM.5GZZO	GTEX-11EI6	Cortex	Male
GTEX.11EMC.3226.SM.5EGKW	GTEX-11EMC	Cortex	Female
GTEX.11GS4.2326.SM.5A5KS	GTEX-11GS4	Cortex	Male
GTEX.11GS4.3126.SM.5A5LH	GTEX-11GS4	Cortex	Male
GTEX.11GSO.2926.SM.5HL73	GTEX-11GSO	Cortex	Male
GTEX.11GSO.0011.R2a.SM.57WDF	GTEX-11GSO	Substantia ni	Male
GTEX.11GSP.0011.R7b.SM.57WC3	GTEX-11GSP	Putamen	Female
GTEX.11GSP.3226.SM.5986O	GTEX-11GSP	Cortex	Female
GTEX.11NUK.2926.SM.5A5MD	GTEX-11NUK	Cortex	Male
GTEX.11NV4.0011.R7a.SM.57WDH	GTEX-11NV4	Putamen	Male
GTEX.11NV4.2126.SM.5N9DS	GTEX-11NV4	Cortex	Male
GTEX.11PRG.2926.SM.5987A	GTEX-11PRG	Cortex	Male
GTEX.11PRG.0011.R2a.SM.69LQ5	GTEX-11PRG	Substantia ni	Male
GTEX.11TTK.1926.SM.5PNW8	GTEX-11TTK	Cortex	Female
GTEX.11TTK.2926.SM.5PNYP	GTEX-11TTK	Cortex	Female
GTEX.11UD1.0011.R2b.SM.5BC6O	GTEX-11UD1	Substantia ni	Female
GTEX.11WQC.0011.R7b.SM.5BC7A	GTEX-11WQC	Putamen	Male
GTEX.11WQK.3026.SM.5EQL6	GTEX-11WQK	Cortex	Male
GTEX.11ZTS.0011.R2b.SM.5BC75	GTEX-11ZTS	Substantia ni	Female
GTEX.11ZUS.2926.SM.5FQSL	GTEX-11ZUS	Cortex	Male
GTEX.11ZVC.0011.R4a.SM.5BC6Z	GTEX-11ZVC	Amygdala	Female
GTEX.12126.1026.SM.5P9JJ	GTEX-12126	Cortex	Male
GTEX.12WSA.0011.R2a.SM.57WDK	GTEX-12WSA	Substantia ni	Male
GTEX.12WSA.0011.R4a.SM.57WB7	GTEX-12WSA	Amygdala	Male
GTEX.12WSA.0011.R7b.SM.5GU5U	GTEX-12WSA	Putamen	Male
GTEX.12WSA.2926.SM.5EQ4D	GTEX-12WSA	Cortex	Male
GTEX.12WSD.0011.R4b.SM.5LZUA	GTEX-12WSD	Amygdala	Female
GTEX.12WSD.3126.SM.5HL7P	GTEX-12WSD	Cortex	Female
GTEX.12WSD.0011.R2a.SM.5LZWB	GTEX-12WSD	Substantia ni	Female
GTEX.12WSD.0011.R7b.SM.5PNWE	GTEX-12WSD	Putamen	Female
GTEX.12WSE.0011.R7a.SM.5PNWF	GTEX-12WSE	Putamen	Male
GTEX.12WSF.0011.R7b.SM.5HL99	GTEX-12WSF	Putamen	Male
GTEX.12WSF.3126.SM.6M478	GTEX-12WSF	Cortex	Male
GTEX.12WSF.0011.R2a.SM.5DUVU	GTEX-12WSF	Substantia ni	Male
GTEX.12WSF.0011.R4b.SM.5HL88	GTEX-12WSF	Amygdala	Male
GTEX.12WSG.0826.SM.5EQ5A	GTEX-12WSG	Cortex	Female

GTEX.12WSH.0011.R2a.SM.5GU68	GTEX-12WSH	Substantia ni	Male
GTEX.12WSH.0011.R4a.SM.5GU6K	GTEX-12WSH	Amygdala	Male
GTEX.12WSH.3026.SM.5CVNI	GTEX-12WSH	Cortex	Male
GTEX.12WSI.0011.R7b.SM.5GU78	GTEX-12WSI	Putamen	Male
GTEX.12WSM.0011.R7b.SM.5EGLD	GTEX-12WSM	Putamen	Male
GTEX.12ZZW.0011.R4a.SM.5DUX9	GTEX-12ZZW	Amygdala	Male
GTEX.12ZZW.0011.R7a.SM.5DUWC	GTEX-12ZZW	Putamen	Male
GTEX.12ZZW.2926.SM.5LZUP	GTEX-12ZZW	Cortex	Male
GTEX.12ZZX.0011.R2a.SM.5EGLG	GTEX-12ZZX	Substantia ni	Female
GTEX.12ZZX.0011.R7b.SM.5DUVV	GTEX-12ZZX	Putamen	Female
GTEX.12ZZX.2926.SM.5GCOQ	GTEX-12ZZX	Cortex	Female
GTEX.12ZZY.0011.R7a.SM.5EGLB	GTEX-12ZZY	Putamen	Male
GTEX.12ZZY.3026.SM.5GCOU	GTEX-12ZZY	Cortex	Male
GTEX.12ZZZ.0011.R7b.SM.5EGLE	GTEX-12ZZZ	Putamen	Male
GTEX.12ZZZ.0011.R4b.SM.5DUV7	GTEX-12ZZZ	Amygdala	Male
GTEX.12ZZZ.3026.SM.5BC67	GTEX-12ZZZ	Cortex	Male
GTEX.13112.0011.R7b.SM.5DUVW	GTEX-13112	Putamen	Male
GTEX.13112.0011.R4b.SM.5DUXL	GTEX-13112	Amygdala	Male
GTEX.13112.2126.SM.5GCO4	GTEX-13112	Cortex	Male
GTEX.1313W.0011.R4b.SM.5KLZV	GTEX-1313W	Amygdala	Female
GTEX.1313W.0011.R7b.SM.5DUWL	GTEX-1313W	Putamen	Female
GTEX.1313W.3126.SM.5LZUI	GTEX-1313W	Cortex	Female
GTEX.1313W.0011.R2a.SM.5EGLF	GTEX-1313W	Substantia ni	Female
GTEX.131XH.0011.R4b.SM.5DUWB	GTEX-131XH	Amygdala	Male
GTEX.131XH.0011.R2b.SM.5DUVY	GTEX-131XH	Substantia ni	Male
GTEX.131XW.3126.SM.5LZUC	GTEX-131XW	Cortex	Female
GTEX.131YS.0011.R4a.SM.5DUVL	GTEX-131YS	Amygdala	Female
GTEX.131YS.3126.SM.5KLYT	GTEX-131YS	Cortex	Female
GTEX.131YS.0011.R7a.SM.5IJDA	GTEX-131YS	Putamen	Female
GTEX.132Q8.0011.R7b.SM.5N9F1	GTEX-132Q8	Putamen	Male
GTEX.132Q8.0011.R2b.SM.5EQ5Z	GTEX-132Q8	Substantia ni	Male
GTEX.132Q8.3026.SM.5PNVG	GTEX-132Q8	Cortex	Male
GTEX.139TT.0011.R7b.SM.5J2MN	GTEX-139TT	Putamen	Male
GTEX.13CF2.0011.R7b.SM.5K7UV	GTEX-13CF2	Putamen	Male
GTEX.13CF2.0011.R2a.SM.5L3DC	GTEX-13CF2	Substantia ni	Male
GTEX.13CZV.0011.R4b.SM.5J2NY	GTEX-13CZV	Amygdala	Male
GTEX.13CZV.0011.R7b.SM.5LZXH	GTEX-13CZV	Putamen	Male
GTEX.13FHO.0011.R7b.SM.5LZXD	GTEX-13FHO	Putamen	Male
GTEX.13FHO.3026.SM.5J1O9	GTEX-13FHO	Cortex	Male
GTEX.13FHP.0011.R7b.SM.5LZYI	GTEX-13FHP	Putamen	Male
GTEX.13FHP.3026.SM.5IJBS	GTEX-13FHP	Cortex	Male
GTEX.13FLV.0011.R7b.SM.5K7WW	GTEX-13FLV	Putamen	Male
GTEX.13FLV.0011.R4a.SM.5LZYU	GTEX-13FLV	Amygdala	Male

GTEX.13FLW.1426.SM.5K7YE	GTEX-13FLW	Cortex	Male
GTEX.13FLW.0011.R7b.SM.5L3EZ	GTEX-13FLW	Putamen	Male
GTEX.13FXS.0011.R2b.SM.5K7XX	GTEX-13FXS	Substantia ni	Male
GTEX.13FXS.0011.R7a.SM.5L3FC	GTEX-13FXS	Putamen	Male
GTEX.13FXS.3126.SM.5J2NN	GTEX-13FXS	Cortex	Male
GTEX.13G51.0011.R2b.SM.5LZXS	GTEX-13G51	Substantia ni	Male
GTEX.13G51.0011.R7b.SM.5LZXG	GTEX-13G51	Putamen	Male
GTEX.13IVO.2926.SM.5L3CZ	GTEX-13IVO	Cortex	Male
GTEX.13JUV.0011.R7b.SM.5LZZG	GTEX-13JUV	Putamen	Female
GTEX.13JUV.2926.SM.5LZX7	GTEX-13JUV	Cortex	Female
GTEX.13JVG.3126.SM.5L3FH	GTEX-13JVG	Cortex	Male
GTEX.13JVG.0011.R4a.SM.5MR4C	GTEX-13JVG	Amygdala	Male
GTEX.13JVG.0011.R7b.SM.5MR3Q	GTEX-13JVG	Putamen	Male
GTEX.13N2G.0011.R2a.SM.5MR4Q	GTEX-13N2G	Substantia ni	Male
GTEX.13N2G.0011.R4b.SM.5MR54	GTEX-13N2G	Amygdala	Male
GTEX.13N2G.0011.R7a.SM.5MR42	GTEX-13N2G	Putamen	Male
GTEX.13NYB.1726.SM.5N9G2	GTEX-13NYB	Cortex	Male
GTEX.13NYB.3026.SM.5IJD7	GTEX-13NYB	Cortex	Male
GTEX.13NYB.0011.R2a.SM.5MR3C	GTEX-13NYB	Substantia ni	Male
GTEX.13NYB.0011.R7a.SM.5MR5D	GTEX-13NYB	Putamen	Male
GTEX.13NYC.2826.SM.5K7WR	GTEX-13NYC	Cortex	Male
GTEX.13NYS.0011.R4b.SM.5MR3O	GTEX-13NYS	Amygdala	Male
GTEX.13NYS.3126.SM.5KLYV	GTEX-13NYS	Cortex	Male
GTEX.13NYS.0011.R2b.SM.5MR4G	GTEX-13NYS	Substantia ni	Male
GTEX.13O1R.2526.SM.5N9FW	GTEX-13O1R	Cortex	Male
GTEX.13O3O.0011.R4b.SM.5KM3F	GTEX-13O3O	Amygdala	Female
GTEX.13O3O.3126.SM.5KM3H	GTEX-13O3O	Cortex	Female
GTEX.13O3O.0011.R7b.SM.5P9GZ	GTEX-13O3O	Putamen	Female
GTEX.13O3O.0011.R2b.SM.5P9H1	GTEX-13O3O	Substantia ni	Female
GTEX.13O3Q.0011.R4a.SM.5P9H2	GTEX-13O3Q	Amygdala	Male
GTEX.13O3Q.2926.SM.5KM45	GTEX-13O3Q	Cortex	Male
GTEX.13O3Q.0011.R7a.SM.5KM38	GTEX-13O3Q	Putamen	Male
GTEX.13O3Q.0011.R2b.SM.5KM3K	GTEX-13O3Q	Substantia ni	Male
GTEX.13OVH.0011.R4a.SM.5KM3W	GTEX-13OVH	Amygdala	Male
GTEX.13OVH.3026.SM.5MR4N	GTEX-13OVH	Cortex	Male
GTEX.13OVI.1126.SM.5KLZF	GTEX-13OVI	Cortex	Female
GTEX.13OVJ.0011.R2b.SM.5L3GP	GTEX-13OVJ	Substantia ni	Female
GTEX.13OVJ.0011.R7a.SM.5L3G1	GTEX-13OVJ	Putamen	Female
GTEX.13OVJ.0011.R4b.SM.5P9H5	GTEX-13OVJ	Amygdala	Female
GTEX.13OVJ.2826.SM.5L3GW	GTEX-13OVJ	Cortex	Female
GTEX.13OVL.0011.R7a.SM.5P9GW	GTEX-13OVL	Putamen	Male
GTEX.13OVL.1826.SM.5KLZR	GTEX-13OVL	Cortex	Male
GTEX.13OVL.0011.R4b.SM.5L3HV	GTEX-13OVL	Amygdala	Male

GTEX.13OW5.0011.R7a.SM.5O9C9	GTEX-13OW5	Putamen	Male
GTEX.13OW5.0011.R4b.SM.5L3HS	GTEX-13OW5	Amygdala	Male
GTEX.13OW6.0011.R2a.SM.5L3HG	GTEX-13OW6	Substantia ni	Male
GTEX.13OW6.0011.R7b.SM.5L3H4	GTEX-13OW6	Putamen	Male
GTEX.13OW6.1826.SM.5N9F9	GTEX-13OW6	Cortex	Male
GTEX.13OW6.0011.R4a.SM.5O9DX	GTEX-13OW6	Amygdala	Male
GTEX.13OW6.3026.SM.5J2MI	GTEX-13OW6	Cortex	Male
GTEX.13OW7.0011.R4b.SM.5O9CX	GTEX-13OW7	Amygdala	Male
GTEX.13OW7.3026.SM.5L3GY	GTEX-13OW7	Cortex	Male
GTEX.13OW8.2826.SM.5L3GC	GTEX-13OW8	Cortex	Male
GTEX.13OW8.0011.R7a.SM.5L3FP	GTEX-13OW8	Putamen	Male
GTEX.13OW8.0011.R2a.SM.5L3G2	GTEX-13OW8	Substantia ni	Male
GTEX.13PL6.3126.SM.5LUAR	GTEX-13PL6	Cortex	Female
GTEX.13PVQ.3026.SM.5SI93	GTEX-13PVQ	Cortex	Male
GTEX.13PVQ.0011.R4a.SM.5O9CT	GTEX-13PVQ	Amygdala	Male
GTEX.13PVQ.0011.R7a.SM.5L3G3	GTEX-13PVQ	Putamen	Male
GTEX.13QIC.2926.SM.5J2NF	GTEX-13QIC	Cortex	Female
GTEX.13QIC.0011.R4a.SM.5PNX8	GTEX-13QIC	Amygdala	Female
GTEX.13QJC.0011.R7b.SM.5PNUM	GTEX-13QJC	Putamen	Female
GTEX.13QJC.0011.R2b.SM.5PNUN	GTEX-13QJC	Substantia ni	Female
GTEX.13RTJ.2226.SM.5S2Q1	GTEX-13RTJ	Cortex	Male
GTEX.13RTJ.0011.R7b.SM.5P9JS	GTEX-13RTJ	Putamen	Male
GTEX.13RTJ.0011.R4b.SM.5PNX1	GTEX-13RTJ	Amygdala	Male
GTEX.13RTJ.0011.R2a.SM.5PNW9	GTEX-13RTJ	Substantia ni	Male
GTEX.13S7M.0011.R7a.SM.5O9DK	GTEX-13S7M	Putamen	Female
GTEX.13S7M.3126.SM.5RQJQ	GTEX-13S7M	Cortex	Female
GTEX.13SLW.0011.R2b.SM.5O9C6	GTEX-13SLW	Substantia ni	Male
GTEX.13SLW.0011.R4b.SM.5S2W2	GTEX-13SLW	Amygdala	Male
GTEX.13SLX.0011.R4a.SM.5O9BJ	GTEX-13SLX	Amygdala	Female
GTEX.13SLX.0011.R2b.SM.5S2VP	GTEX-13SLX	Substantia ni	Female
GTEX.13SLX.3126.SM.5S2Q5	GTEX-13SLX	Cortex	Female
GTEX.13VXU.0011.R4b.SM.5O9CI	GTEX-13VXU	Amygdala	Male
GTEX.13VXU.0011.R7b.SM.5SI72	GTEX-13VXU	Putamen	Male
GTEX.13VXU.2926.SM.5LU5C	GTEX-13VXU	Cortex	Male
GTEX.13X6I.0011.R4b.SM.5PNU9	GTEX-13X6I	Amygdala	Male
GTEX.13X6I.0011.R2b.SM.5PNWQ	GTEX-13X6I	Substantia ni	Male
GTEX.13X6I.0011.R7b.SM.5PNWP	GTEX-13X6I	Putamen	Male
GTEX.13X6J.0011.R7b.SM.5PNUC	GTEX-13X6J	Putamen	Male
GTEX.13X6J.3026.SM.5Q5CU	GTEX-13X6J	Cortex	Male
GTEX.13X6J.0011.R4b.SM.5P9K4	GTEX-13X6J	Amygdala	Male
GTEX.13X6K.0011.R4b.SM.5P9HO	GTEX-13X6K	Amygdala	Female
GTEX.13X6K.0011.R2b.SM.5P9K3	GTEX-13X6K	Substantia ni	Female
GTEX.13X6K.0011.R7b.SM.5P9K7	GTEX-13X6K	Putamen	Female

GTEX.13X6K.2926.SM.5Q5D5	GTEX-13X6K	Cortex	Female
GTEX.145MF.2726.SM.5O995	GTEX-145MF	Cortex	Male
GTEX.145MG.3026.SM.5RQJA	GTEX-145MG	Cortex	Male
GTEX.145MG.0011.R7b.SM.5P9JY	GTEX-145MG	Putamen	Male
GTEX.145MH.3026.SM.5Q5DZ	GTEX-145MH	Cortex	Male
GTEX.145MH.0011.R7a.SM.5PNWH	GTEX-145MH	Putamen	Male
GTEX.145MI.0011.R4b.SM.5PNZH	GTEX-145MI	Amygdala	Female
GTEX.145MN.0326.SM.5QGQI	GTEX-145MN	Cortex	Male
GTEX.147F4.2626.SM.5Q5CS	GTEX-147F4	Cortex	Male
GTEX.147GR.0011.R4a.SM.5S2V2	GTEX-147GR	Amygdala	Male
GTEX.147GR.0011.R7a.SM.5S2RP	GTEX-147GR	Putamen	Male
GTEX.147GR.3026.SM.5S2ML	GTEX-147GR	Cortex	Male
GTEX.147GR.0011.R2b.SM.5S2RQ	GTEX-147GR	Substantia ni	Male
GTEX.1497J.0826.SM.5NQAJ	GTEX-1497J	Cortex	Male
GTEX.14A5I.2926.SM.5Q5CQ	GTEX-14A5I	Cortex	Female
GTEX.14ABY.0011.R4b.SM.5S2VN	GTEX-14ABY	Amygdala	Male
GTEX.14ABY.0011.R7b.SM.5PNWS	GTEX-14ABY	Putamen	Male
GTEX.14ASI.0011.R2b.SM.5YY9H	GTEX-14ASI	Substantia ni	Male
GTEX.14ASI.0011.R4a.SM.69LQ4	GTEX-14ASI	Amygdala	Male
GTEX.14ASI.3026.SM.5S2PN	GTEX-14ASI	Cortex	Male
GTEX.14ASI.0011.R7b.SM.5YY9G	GTEX-14ASI	Putamen	Male
GTEX.14BIL.3026.SM.7EWD4	GTEX-14BIL	Cortex	Male
GTEX.14BIM.0011.R2b.SM.69LQ6	GTEX-14BIM	Substantia ni	Female
GTEX.14BIM.0011.R4b.SM.5S2RK	GTEX-14BIM	Amygdala	Female
GTEX.14BIM.3026.SM.7EWCY	GTEX-14BIM	Cortex	Female
GTEX.14BIN.0011.R2a.SM.5S2RJ	GTEX-14BIN	Substantia ni	Female
GTEX.14BIN.0011.R7b.SM.5S2RI	GTEX-14BIN	Putamen	Female
GTEX.14BIN.3226.SM.62LDR	GTEX-14BIN	Cortex	Female
GTEX.14BMV.0011.R7b.SM.5YY9J	GTEX-14BMV	Putamen	Male
GTEX.14BMV.3026.SM.5S2PQ	GTEX-14BMV	Cortex	Male
GTEX.14C39.3126.SM.5ZZW6	GTEX-14C39	Cortex	Male
GTEX.14C39.2126.SM.664OH	GTEX-14C39	Cortex	Male
GTEX.14C5O.0011.R7b.SM.5YYAH	GTEX-14C5O	Putamen	Male
GTEX.14C5O.0011.R2a.SM.5ZZV2	GTEX-14C5O	Substantia ni	Male
GTEX.14C5O.3026.SM.5YYB2	GTEX-14C5O	Cortex	Male
GTEX.14C5O.2026.SM.5YYB1	GTEX-14C5O	Cortex	Male
GTEX.14DAQ.0011.R4a.SM.5YYAI	GTEX-14DAQ	Amygdala	Female
GTEX.14DAQ.3126.SM.62LDS	GTEX-14DAQ	Cortex	Female
GTEX.14E6D.2526.SM.5YYA9	GTEX-14E6D	Cortex	Male
GTEX.14E7W.0011.R4b.SM.62LEA	GTEX-14E7W	Amygdala	Male
GTEX.14E7W.0011.R7b.SM.5ZZV4	GTEX-14E7W	Putamen	Male
GTEX.14JG1.0011.R4b.SM.62LEB	GTEX-14JG1	Amygdala	Male
GTEX.14JG1.0011.R2a.SM.5ZZVE	GTEX-14JG1	Substantia ni	Male

GTEX.14JG1.3026.SM.5YYAA	GTEX-14JG1	Cortex	Male
GTEX.14JG1.0011.R7b.SM.5YYAV	GTEX-14JG1	Putamen	Male
GTEX.14JIY.0011.R4b.SM.5ZZV7	GTEX-14JIY	Amygdala	Male
GTEX.14JIY.2926.SM.69LPT	GTEX-14JIY	Cortex	Male
GTEX.14LZ3.3026.SM.5ZZVU	GTEX-14LZ3	Cortex	Female
GTEX.14LZ3.0011.R7a.SM.69LQN	GTEX-14LZ3	Putamen	Female
GTEX.14PJM.0011.R7a.SM.6AJBX	GTEX-14PJM	Putamen	Female
GTEX.14PJM.3126.SM.6EU2R	GTEX-14PJM	Cortex	Female
GTEX.14PJM.0011.R4a.SM.5YYAS	GTEX-14PJM	Amygdala	Female
GTEX.14PJO.0011.R2a.SM.5ZZVF	GTEX-14PJO	Substantia ni	Male
GTEX.14PJO.0011.R7a.SM.664NR	GTEX-14PJO	Putamen	Male
GTEX.14PN4.0011.R4a.SM.686ZT	GTEX-14PN4	Amygdala	Male
GTEX.14PN4.0011.R7b.SM.69LQQ	GTEX-14PN4	Putamen	Male
GTEX.14PN4.0011.R2a.SM.6EU2Y	GTEX-14PN4	Substantia ni	Male
GTEX.14PQA.0011.R4b.SM.6AJBU	GTEX-14PQA	Amygdala	Female
GTEX.15CHQ.0011.R2b.SM.6AJAD	GTEX-15CHQ	Substantia ni	Male
GTEX.15CHQ.0011.R4a.SM.686ZX	GTEX-15CHQ	Amygdala	Male
GTEX.15CHQ.2126.SM.6871M	GTEX-15CHQ	Cortex	Male
GTEX.15DCD.0011.R2b.SM.6LPIE	GTEX-15DCD	Substantia ni	Female
GTEX.15DCD.0011.R4a.SM.69LPB	GTEX-15DCD	Amygdala	Female
GTEX.15DCD.0011.R7b.SM.6LPID	GTEX-15DCD	Putamen	Female
GTEX.15DDE.0011.R4b.SM.7KULI	GTEX-15DDE	Amygdala	Female
GTEX.15DYW.0011.R7a.SM.7KULG	GTEX-15DYW	Putamen	Female
GTEX.15DYW.0011.R4a.SM.6M46V	GTEX-15DYW	Amygdala	Female
GTEX.15DYW.0011.R2b.SM.7KUKY	GTEX-15DYW	Substantia ni	Female
GTEX.15DYW.2026.SM.6AJBD	GTEX-15DYW	Cortex	Female
GTEX.15EO6.0011.R7a.SM.6M46Q	GTEX-15EO6	Putamen	Male
GTEX.15EO6.0011.R2a.SM.6M46R	GTEX-15EO6	Substantia ni	Male
GTEX.15EO6.3026.SM.7KUMA	GTEX-15EO6	Cortex	Male
GTEX.15ER7.0011.R7a.SM.6M46N	GTEX-15ER7	Putamen	Female
GTEX.15ER7.3126.SM.7KUGH	GTEX-15ER7	Cortex	Female
GTEX.15ER7.0011.R4a.SM.6M46S	GTEX-15ER7	Amygdala	Female
GTEX.15ER7.0011.R2a.SM.6M46O	GTEX-15ER7	Substantia ni	Female
GTEX.15G19.0011.R7b.SM.6M479	GTEX-15G19	Putamen	Male
GTEX.15G19.2926.SM.7KUFL	GTEX-15G19	Cortex	Male
GTEX.15RJE.3026.SM.7KUM1	GTEX-15RJE	Cortex	Male
GTEX.15RJE.0011.R2b.SM.79OMP	GTEX-15RJE	Substantia ni	Male
GTEX.15RJE.0011.R7a.SM.7DHL9	GTEX-15RJE	Putamen	Male
GTEX.15SHU.0011.R4b.SM.7IGLC	GTEX-15SHU	Amygdala	Female
GTEX.15SHU.3126.SM.6M46H	GTEX-15SHU	Cortex	Female
GTEX.15UF6.3026.SM.7KUKL	GTEX-15UF6	Cortex	Female
GTEX.16GPK.3026.SM.79ON9	GTEX-16GPK	Cortex	Male
GTEX.16MT8.1926.SM.7EPGL	GTEX-16MT8	Cortex	Female

GTEX.16XZZ.3026.SM.79OMJ	GTEX-16XZZ	Cortex	Male
GTEX.16YQH.3026.SM.79OMM	GTEX-16YQH	Cortex	Male
GTEX.16YQH.0011.R4a.SM.7EPGI	GTEX-16YQH	Amygdala	Male
GTEX.16Z82.0011.R2b.SM.79OK9	GTEX-16Z82	Substantia ni	Male
GTEX.16Z82.3026.SM.79ON3	GTEX-16Z82	Cortex	Male
GTEX.16Z82.0011.R7a.SM.7MKGJ	GTEX-16Z82	Putamen	Male
GTEX.17EVP.0011.R4a.SM.79OML	GTEX-17EVP	Amygdala	Male
GTEX.17EVP.0011.R7a.SM.7LTAN	GTEX-17EVP	Putamen	Male
GTEX.17EVP.0011.R2a.SM.7LG4W	GTEX-17EVP	Substantia ni	Male
GTEX.17EVP.3026.SM.7IGQL	GTEX-17EVP	Cortex	Male
GTEX.17EVQ.2826.SM.7IGQK	GTEX-17EVQ	Cortex	Male
GTEX.17EVQ.0011.R7a.SM.7EWDA	GTEX-17EVQ	Putamen	Male
GTEX.17EVQ.0011.R4b.SM.7LT94	GTEX-17EVQ	Amygdala	Male
GTEX.17F97.0011.R4b.SM.7KFS2	GTEX-17F97	Amygdala	Male
GTEX.17F97.3026.SM.79OKC	GTEX-17F97	Cortex	Male
GTEX.17F97.1926.SM.7IGM4	GTEX-17F97	Cortex	Male
GTEX.17HHY.0011.R7a.SM.718A1	GTEX-17HHY	Putamen	Male
GTEX.17HHY.3026.SM.79OKP	GTEX-17HHY	Cortex	Male
GTEX.17HHY.2326.SM.7KFSR	GTEX-17HHY	Cortex	Male
GTEX.17HII.0011.R7a.SM.79OMS	GTEX-17HII	Putamen	Male
GTEX.17HII.3026.SM.7EWFM	GTEX-17HII	Cortex	Male
GTEX.17JCI.3126.SM.7LTA2	GTEX-17JCI	Cortex	Female
GTEX.17MF6.0011.R7b.SM.7LG6J	GTEX-17MF6	Putamen	Male
GTEX.17MF6.2926.SM.7LTAW	GTEX-17MF6	Cortex	Male
GTEX.183WM.2826.SM.731C2	GTEX-183WM	Cortex	Female
GTEX.183WM.0011.R4a.SM.7LG6S	GTEX-183WM	Amygdala	Female
GTEX.183WM.0011.R7b.SM.731AX	GTEX-183WM	Putamen	Female
GTEX.1A3MW.2226.SM.73KUX	GTEX-1A3MW	Cortex	Female
GTEX.1A3MX.3026.SM.731BF	GTEX-1A3MX	Cortex	Male
GTEX.1A3MX.0011.R2b.SM.7EWFL	GTEX-1A3MX	Substantia ni	Male
GTEX.1A3MX.0011.R7a.SM.7DUEM	GTEX-1A3MX	Putamen	Male
GTEX.1A3MX.0011.R4b.SM.7EWFK	GTEX-1A3MX	Amygdala	Male
GTEX.1A8G6.2926.SM.731CK	GTEX-1A8G6	Cortex	Male
GTEX.1A8G7.3126.SM.7IGP7	GTEX-1A8G7	Cortex	Male
GTEX.1A8G7.0011.R2a.SM.7IGQI	GTEX-1A8G7	Substantia ni	Male
GTEX.1AX8Z.2326.SM.731CR	GTEX-1AX8Z	Cortex	Male
GTEX.1AX9I.0011.R4b.SM.7EPIB	GTEX-1AX9I	Amygdala	Male
GTEX.1AX9I.3026.SM.73KUH	GTEX-1AX9I	Cortex	Male
GTEX.1B8L1.0011.R4a.SM.7EWFJ	GTEX-1B8L1	Amygdala	Male
GTEX.1B8L1.3026.SM.7EPHK	GTEX-1B8L1	Cortex	Male
GTEX.1B8SF.3126.SM.73KU1	GTEX-1B8SF	Cortex	Male
GTEX.1B8SG.1726.SM.73KXK	GTEX-1B8SG	Cortex	Male
GTEX.1B8SG.0011.R2b.SM.79OOT	GTEX-1B8SG	Substantia ni	Male

GTEX.1B933.0011.R7a.SM.7DUGX	GTEX-1B933	Putamen	Female
GTEX.1B933.0011.R2b.SM.7IGNM	GTEX-1B933	Substantia ni	Female
GTEX.1B996.0011.R7b.SM.7IGNT	GTEX-1B996	Putamen	Male
GTEX.1B996.0011.R2a.SM.7IGON	GTEX-1B996	Substantia ni	Male
GTEX.1BAJH.1826.SM.731DA	GTEX-1BAJH	Cortex	Male
GTEX.1C64N.0011.R7a.SM.7IGOI	GTEX-1C64N	Putamen	Male
GTEX.1CB4H.3126.SM.7IGN4	GTEX-1CB4H	Cortex	Female
GTEX.N7MS.0011.R7a.SM.2HMKN	GTEX-N7MS	Putamen	Male
GTEX.N7MS.0011.R2a.SM.2HML6	GTEX-N7MS	Substantia ni	Male
GTEX.N7MS.0011.R4a.SM.2HMKW	GTEX-N7MS	Amygdala	Male
GTEX.N7MT.0011.R4a.SM.2I3G9	GTEX-N7MT	Amygdala	Female
GTEX.N7MT.0011.R7a.SM.2I3FZ	GTEX-N7MT	Putamen	Female
GTEX.N7MT.0011.R2a.SM.2I3GI	GTEX-N7MT	Substantia ni	Female
GTEX.NL3H.0011.R4a.SM.2I3GK	GTEX-NL3H	Amygdala	Male
GTEX.NL3H.0011.R7a.SM.2I3G5	GTEX-NL3H	Putamen	Male
GTEX.NL4W.0011.R4a.SM.2I5GZ	GTEX-NL4W	Amygdala	Male
GTEX.NL4W.0011.R2a.SM.2I5GV	GTEX-NL4W	Substantia ni	Male
GTEX.NPJ7.0011.R4a.SM.2I3GJ	GTEX-NPJ7	Amygdala	Female
GTEX.NPJ7.0011.R2a.SM.2I3GF	GTEX-NPJ7	Substantia ni	Female
GTEX.NPJ7.2726.SM.2I3FT	GTEX-NPJ7	Cortex	Female
GTEX.NPJ8.0011.R4a.SM.2HML3	GTEX-NPJ8	Amygdala	Male
GTEX.NPJ8.1526.SM.2D7VU	GTEX-NPJ8	Cortex	Male
GTEX.NPJ8.0011.R2a.SM.2TC6M	GTEX-NPJ8	Substantia ni	Male
GTEX.NPJ8.0011.R7a.SM.2HMJV	GTEX-NPJ8	Putamen	Male
GTEX.NPJ8.2226.SM.3TW8D	GTEX-NPJ8	Cortex	Male
GTEX.OHPN.0011.R7A.SM.2I5FI	GTEX-OHPN	Putamen	Female
GTEX.OHPN.0011.R2A.SM.2I5FB	GTEX-OHPN	Substantia ni	Female
GTEX.OHPN.0011.R4A.SM.2I5FD	GTEX-OHPN	Amygdala	Female
GTEX.OXRN.2426.SM.2I5EQ	GTEX-OXRN	Cortex	Male
GTEX.OXRO.0011.R2A.SM.3NB1W	GTEX-OXRO	Substantia ni	Female
GTEX.P44H.0011.R4A.SM.2XCEW	GTEX-P44H	Amygdala	Male
GTEX.P4QS.1126.SM.3NMD5	GTEX-P4QS	Cortex	Male
GTEX.PVOW.2526.SM.2XCF7	GTEX-PVOW	Cortex	Male
GTEX.PWO3.0926.SM.2I5EY	GTEX-PWO3	Cortex	Female
GTEX.PWO3.0011.R2A.SM.2S1OX	GTEX-PWO3	Substantia ni	Female
GTEX.Q2AG.0011.R4A.SM.2HMKA	GTEX-Q2AG	Amygdala	Female
GTEX.Q2AG.2926.SM.2HMJ3	GTEX-Q2AG	Cortex	Female
GTEX.Q2AG.0011.R7A.SM.2HMJP	GTEX-Q2AG	Putamen	Female
GTEX.Q2AG.0011.R2A.SM.2HMIT	GTEX-Q2AG	Substantia ni	Female
GTEX.QDT8.0011.R4A.SM.32PKM	GTEX-QDT8	Amygdala	Female
GTEX.QDT8.2926.SM.32PKC	GTEX-QDT8	Cortex	Female
GTEX.QDT8.0011.R2A.SM.32PKQ	GTEX-QDT8	Substantia ni	Female
GTEX.QDT8.0011.R7A.SM.32PKF	GTEX-QDT8	Putamen	Female

GTEX.QDVN.1626.SM.48TZC	GTEX-QDVN	Cortex	Male
GTEX.QMR6.0011.R4A.SM.32PKU	GTEX-QMR6	Amygdala	Male
GTEX.QMR6.1426.SM.32PLA	GTEX-QMR6	Cortex	Male
GTEX.QMR6.0011.R2A.SM.32PKV	GTEX-QMR6	Substantia ni	Male
GTEX.QMR6.0011.R7A.SM.32PKL	GTEX-QMR6	Putamen	Male
GTEX.QVJO.1426.SM.2S1QY	GTEX-QVJO	Cortex	Female
GTEX.QVJO.0011.R4A.SM.2S1QL	GTEX-QVJO	Amygdala	Female
GTEX.QVUS.2826.SM.3GADB	GTEX-QVUS	Cortex	Female
GTEX.QVUS.0011.R4A.SM.3GAE7	GTEX-QVUS	Amygdala	Female
GTEX.R55E.0011.R7A.SM.2TC5Z	GTEX-R55E	Putamen	Male
GTEX.R55E.0011.R4A.SM.2TC5H	GTEX-R55E	Amygdala	Male
GTEX.RN64.1626.SM.48FD7	GTEX-RN64	Cortex	Male
GTEX.RNOR.2326.SM.2TF4I	GTEX-RNOR	Cortex	Female
GTEX.RNOR.0011.R4A.SM.3GAD3	GTEX-RNOR	Amygdala	Female
GTEX.RNOR.0011.R7A.SM.2TF4V	GTEX-RNOR	Putamen	Female
GTEX.RU72.0011.R2A.SM.2TF6O	GTEX-RU72	Substantia ni	Female
GTEX.RU72.0011.R7A.SM.2TF5U	GTEX-RU72	Putamen	Female
GTEX.S7SE.0011.R7A.SM.2XCDI	GTEX-S7SE	Putamen	Male
GTEX.S7SE.0011.R4A.SM.2XCDB	GTEX-S7SE	Amygdala	Male
GTEX.S7SE.0011.R2A.SM.2XCDC	GTEX-S7SE	Substantia ni	Male
GTEX.T2IS.0011.R2A.SM.32QPF	GTEX-T2IS	Substantia ni	Female
GTEX.T2IS.3026.SM.32QPM	GTEX-T2IS	Cortex	Female
GTEX.T5JC.2426.SM.3NMDB	GTEX-T5JC	Cortex	Male
GTEX.T5JC.1526.SM.4DM68	GTEX-T5JC	Cortex	Male
GTEX.T5JC.0011.R7A.SM.32PME	GTEX-T5JC	Putamen	Male
GTEX.T5JC.0011.R2A.SM.32PLZ	GTEX-T5JC	Substantia ni	Male
GTEX.T5JC.0011.R4A.SM.32PLT	GTEX-T5JC	Amygdala	Male
GTEX.T6MN.0011.R4A.SM.32QPG	GTEX-T6MN	Amygdala	Male
GTEX.T6MN.2626.SM.32PMQ	GTEX-T6MN	Cortex	Male
GTEX.T6MN.0011.R7A.SM.32QP5	GTEX-T6MN	Putamen	Male
GTEX.T6MN.0011.R2A.SM.32QOW	GTEX-T6MN	Substantia ni	Male
GTEX.TSE9.0011.R7A.SM.3DB7P	GTEX-TSE9	Putamen	Female
GTEX.TSE9.0011.R4A.SM.3DB7H	GTEX-TSE9	Amygdala	Female
GTEX.TSE9.3026.SM.3DB76	GTEX-TSE9	Cortex	Female
GTEX.UTHO.0011.R4A.SM.3GIJP	GTEX-UTHO	Amygdala	Male
GTEX.UTHO.0011.R2A.SM.3GIKC	GTEX-UTHO	Substantia ni	Male
GTEX.UTHO.3026.SM.3GAFB	GTEX-UTHO	Cortex	Male
GTEX.WHSE.3026.SM.3P5ZH	GTEX-WHSE	Cortex	Male
GTEX.WHSE.0011.R4A.SM.3P5ZN	GTEX-WHSE	Amygdala	Male
GTEX.WHSE.0011.R2A.SM.3P5ZL	GTEX-WHSE	Substantia ni	Male
GTEX.WHSE.0011.R7A.SM.3P5YZ	GTEX-WHSE	Putamen	Male
GTEX.WL46.0011.R7A.SM.3LK7X	GTEX-WL46	Putamen	Male
GTEX.WL46.2926.SM.3LK82	GTEX-WL46	Cortex	Male

GTEX.WVLH.3026.SM.3MJG9	GTEX-WVLH	Cortex	Male
GTEX.WVLH.0011.R4A.SM.3MJFS	GTEX-WVLH	Amygdala	Male
GTEX.WVLH.0011.R7A.SM.3MJFB	GTEX-WVLH	Putamen	Male
GTEX.WWYW.3126.SM.3NB39	GTEX-WWYW	Cortex	Female
GTEX.WWYW.0011.R7A.SM.3NB3H	GTEX-WWYW	Putamen	Female
GTEX.WZTO.0011.R4A.SM.3NMC7	GTEX-WZTO	Amygdala	Male
GTEX.WZTO.2926.SM.3NM9I	GTEX-WZTO	Cortex	Male
GTEX.WZTO.0011.R7B.SM.4E3IS	GTEX-WZTO	Putamen	Male
GTEX.X261.0011.R7A.SM.4E3JJ	GTEX-X261	Putamen	Male
GTEX.X4XX.0011.R2A.SM.3P623	GTEX-X4XX	Substantia ni	Male
GTEX.X4XX.3026.SM.3NMB2	GTEX-X4XX	Cortex	Male
GTEX.X4XX.0011.R4B.SM.46MWL	GTEX-X4XX	Amygdala	Male
GTEX.X585.0011.R4B.SM.46MVH	GTEX-X585	Amygdala	Male
GTEX.X585.3026.SM.46MWF	GTEX-X585	Cortex	Male
GTEX.X585.0011.R2B.SM.46MVF	GTEX-X585	Substantia ni	Male
GTEX.XMD1.0011.R2B.SM.4AT5N	GTEX-XMD1	Substantia ni	Male
GTEX.XPVG.0526.SM.4B65N	GTEX-XPVG	Cortex	Male
GTEX.Y111.2726.SM.4TT3N	GTEX-Y111	Cortex	Male
GTEX.Y5V6.2026.SM.5IFHO	GTEX-Y5V6	Cortex	Male
GTEX.Y8DK.0011.R7A.SM.4SOJZ	GTEX-Y8DK	Putamen	Male
GTEX.Y8DK.0826.SM.4TT3T	GTEX-Y8DK	Cortex	Male
GTEX.YFC4.0011.R4a.SM.4RGLQ	GTEX-YFC4	Amygdala	Female
GTEX.YFC4.0011.R2a.SM.4V6DZ	GTEX-YFC4	Substantia ni	Female
GTEX.YFC4.0011.R7b.SM.4V6E2	GTEX-YFC4	Putamen	Female
GTEX.YFC4.3126.SM.5PNV6	GTEX-YFC4	Cortex	Female
GTEX.YJ89.0011.R7a.SM.4V6GO	GTEX-YJ89	Putamen	Male
GTEX.YJ89.3026.SM.5IFJI	GTEX-YJ89	Cortex	Male
GTEX.YJ89.0011.R2b.SM.4RGLT	GTEX-YJ89	Substantia ni	Male
GTEX.Z93S.0011.R2a.SM.4RGNG	GTEX-Z93S	Substantia ni	Male
GTEX.Z93S.0011.R7b.SM.4RGNK	GTEX-Z93S	Putamen	Male
GTEX.Z93S.2926.SM.57WB9	GTEX-Z93S	Cortex	Male
GTEX.ZAB4.0011.R2a.SM.4RGNP	GTEX-ZAB4	Substantia ni	Male
GTEX.ZAB4.0011.R4a.SM.4SOKB	GTEX-ZAB4	Amygdala	Male
GTEX.ZAB4.0011.R7a.SM.4SOKE	GTEX-ZAB4	Putamen	Male
GTEX.ZAJG.3126.SM.5HL9J	GTEX-ZAJG	Cortex	Female
GTEX.ZC5H.1726.SM.5HL7X	GTEX-ZC5H	Cortex	Female
GTEX.ZDXO.0011.R7a.SM.4WWCU	GTEX-ZDXO	Putamen	Male
GTEX.ZDXO.2126.SM.4WKFI	GTEX-ZDXO	Cortex	Male
GTEX.ZDXO.0226.SM.4WKH7	GTEX-ZDXO	Cortex	Male
GTEX.ZDXO.0011.R2b.SM.4WKFG	GTEX-ZDXO	Substantia ni	Male
GTEX.ZE7O.3126.SM.5HL5X	GTEX-ZE7O	Cortex	Female
GTEX.ZE7O.0011.R7a.SM.57WCU	GTEX-ZE7O	Putamen	Female
GTEX.ZF28.3026.SM.4WKHP	GTEX-ZF28	Cortex	Male

GTEX.ZF28.0011.R7a.SM.4WKF5	GTEX-ZF28	Putamen	Male
GTEX.ZLFU.0926.SM.5P9F8	GTEX-ZLFU	Cortex	Male
GTEX.ZUA1.3026.SM.59HJC	GTEX-ZUA1	Cortex	Male
GTEX.ZUA1.0011.R7b.SM.4YCDP	GTEX-ZUA1	Putamen	Male
GTEX.ZV68.0011.R2a.SM.4YCDK	GTEX-ZV68	Substantia ni	Female
GTEX.ZV68.0011.R7a.SM.51MT4	GTEX-ZV68	Putamen	Female
GTEX.ZVT3.3026.SM.5E43N	GTEX-ZVT3	Cortex	Female
GTEX.ZVT3.0011.R7a.SM.57WCO	GTEX-ZVT3	Putamen	Female
GTEX.ZVZP.0926.SM.5GIDB	GTEX-ZVZP	Cortex	Male
GTEX.ZVZQ.0011.R4b.SM.57WCZ	GTEX-ZVZQ	Amygdala	Female
GTEX.ZVZQ.0011.R7b.SM.57WBB	GTEX-ZVZQ	Putamen	Female
GTEX.ZXG5.0011.R7b.SM.57WCC	GTEX-ZXG5	Putamen	Male
GTEX.ZYFD.3026.SM.5E44C	GTEX-ZYFD	Cortex	Male
GTEX.ZYFG.1626.SM.5GZYY	GTEX-ZYFG	Cortex	Female
GTEX.ZYT6.2226.SM.5GIC9	GTEX-ZYT6	Cortex	Male
GTEX.ZYY3.3126.SM.5SI9L	GTEX-ZYY3	Cortex	Female
GTEX.ZZPT.3026.SM.5GZXH	GTEX-ZZPT	Cortex	Male

Age	AgeGroup	RIN
60-69	Aged	6.2
60-69	Aged	6.1
60-69	Aged	6.3
60-69	Aged	6.4
50-59	Young	7.1
60-69	Aged	7.7
60-69	Aged	6.4
60-69	Aged	6
60-69	Aged	6.1
60-69	Aged	6.2
60-69	Aged	6.8
60-69	Aged	6.6
60-69	Aged	7.8
60-69	Aged	6.1
50-59	Young	7.1
60-69	Aged	7.5
60-69	Aged	5.8
50-59	Young	5.9
50-59	Young	6
60-69	Aged	6.2
60-69	Aged	6.1
40-49	Young	8.1
60-69	Aged	7
50-59	Young	6.6
60-69	Aged	7.6
30-39	Young	6.3
50-59	Young	7
20-29	Young	6.9
60-69	Aged	5.8
60-69	Aged	6.5
60-69	Aged	6
60-69	Aged	5.8
60-69	Aged	7.2
60-69	Aged	5.8
60-69	Aged	5.8
60-69	Aged	7.9
20-29	Young	5.9
70-79	Aged	7.1
70-79	Aged	7.1
70-79	Aged	5.9
70-79	Aged	7.3
50-59	Young	7.3

50-59	Young	6
50-59	Young	6
50-59	Young	6.4
50-59	Young	7.1
60-69	Aged	7.7
50-59	Young	7
50-59	Young	7
50-59	Young	6.2
40-49	Young	7.9
40-49	Young	8.4
40-49	Young	6.8
60-69	Aged	7.8
60-69	Aged	6.7
60-69	Aged	8.1
60-69	Aged	6.1
60-69	Aged	6.2
50-59	Young	8.7
50-59	Young	7.1
50-59	Young	6.2
50-59	Young	6.9
50-59	Young	8.9
50-59	Young	6.9
50-59	Young	7
50-59	Young	7.7
50-59	Young	7.1
50-59	Young	6.2
60-69	Aged	7.1
60-69	Aged	6.6
60-69	Aged	7.8
60-69	Aged	5.7
60-69	Aged	7.9
60-69	Aged	6.9
60-69	Aged	7.1
60-69	Aged	7
60-69	Aged	6.1
60-69	Aged	6.8
60-69	Aged	6.9
60-69	Aged	6.1
60-69	Aged	6.8
50-59	Young	6.7
50-59	Young	6.1
50-59	Young	7.9
50-59	Young	5.9

50-59	Young	6.8
50-59	Young	7.5
50-59	Young	6.5
50-59	Young	8.1
50-59	Young	7
60-69	Aged	6.1
60-69	Aged	7.9
70-79	Aged	6.1
40-49	Young	5.8
40-49	Young	5.8
60-69	Aged	6
60-69	Aged	6
60-69	Aged	5.9
60-69	Aged	5.8
60-69	Aged	6.3
60-69	Aged	6.8
40-49	Young	5.7
40-49	Young	6.1
40-49	Young	7.7
40-49	Young	7.1
60-69	Aged	7.1
60-69	Aged	6.3
60-69	Aged	6.9
60-69	Aged	6.7
60-69	Aged	5.7
60-69	Aged	7
60-69	Aged	6.3
60-69	Aged	7.6
60-69	Aged	6.7
50-59	Young	6.7
50-59	Young	6.1
50-59	Young	8.5
50-59	Young	6.9
70-79	Aged	6.3
70-79	Aged	6.3
60-69	Aged	8.8
50-59	Young	6.6
50-59	Young	7.6
50-59	Young	6.7
50-59	Young	6.4
50-59	Young	6
50-59	Young	6
50-59	Young	6.1

60-69	Aged	7.3
60-69	Aged	6.9
50-59	Young	6.5
50-59	Young	8
50-59	Young	5.7
50-59	Young	5.9
50-59	Young	6.5
50-59	Young	5.8
50-59	Young	6
60-69	Aged	5.9
60-69	Aged	7
60-69	Aged	6.7
40-49	Young	5.7
50-59	Young	5.8
50-59	Young	6.3
50-59	Young	6.7
60-69	Aged	6.2
60-69	Aged	5.9
60-69	Aged	7.1
60-69	Aged	6.7
60-69	Aged	5.9
60-69	Aged	9.1
60-69	Aged	7.2
60-69	Aged	7
60-69	Aged	7
60-69	Aged	8
70-79	Aged	6.2
70-79	Aged	6.8
50-59	Young	6.1
50-59	Young	6.5
50-59	Young	5.7
50-59	Young	7.3
50-59	Young	8.3
50-59	Young	6
60-69	Aged	7.8
60-69	Aged	8.4
60-69	Aged	6.5
50-59	Young	8.7
50-59	Young	6.2
50-59	Young	6.5
60-69	Aged	6.7
60-69	Aged	6.7
60-69	Aged	8.2

60-69	Aged	6.5
50-59	Young	6.5
50-59	Young	7.1
50-59	Young	6.8
50-59	Young	6.8
50-59	Young	8.4
60-69	Aged	5.7
30-39	Young	7.5
50-59	Young	6.8
60-69	Aged	6.2
60-69	Aged	7.2
60-69	Aged	6.1
60-69	Aged	6.9
60-69	Aged	6.3
50-59	Young	6.6
50-59	Young	5.8
50-59	Young	5.7
60-69	Aged	5.8
60-69	Aged	6.5
60-69	Aged	6.7
60-69	Aged	7.1
50-59	Young	7.2
60-69	Aged	5.8
60-69	Aged	6.8
60-69	Aged	6.3
50-59	Young	6.4
50-59	Young	8
50-59	Young	7.4
50-59	Young	6.2
50-59	Young	6.5
40-49	Young	7.2
40-49	Young	6.3
60-69	Aged	9.1
60-69	Aged	7.9
60-69	Aged	7.5
60-69	Aged	5.9
60-69	Aged	7.1
60-69	Aged	7.3
50-59	Young	7.5
70-79	Aged	6.6
70-79	Aged	6
40-49	Young	6.6
40-49	Young	7.2

40-49	Young	6.4
40-49	Young	8.5
60-69	Aged	7.3
60-69	Aged	6.8
60-69	Aged	6.1
60-69	Aged	7.4
50-59	Young	7.8
50-59	Young	5.8
50-59	Young	6.8
60-69	Aged	6.7
60-69	Aged	6.4
50-59	Young	6.9
50-59	Young	7.5
50-59	Young	7.1
50-59	Young	7.1
60-69	Aged	7.7
60-69	Aged	7.7
60-69	Aged	6.1
70-79	Aged	6.8
70-79	Aged	7.5
70-79	Aged	7.8
50-59	Young	6.8
60-69	Aged	7.4
60-69	Aged	8
60-69	Aged	7.3
60-69	Aged	5.8
50-59	Young	7.9
50-59	Young	7.5
50-59	Young	7.4
20-29	Young	9.2
20-29	Young	6.9
20-29	Young	6.7
20-29	Young	8
60-69	Aged	7.2
60-69	Aged	6.5
40-49	Young	6.4
40-49	Young	6.7
40-49	Young	8.4
40-49	Young	6.2
40-49	Young	7.3
70-79	Aged	5.9
60-69	Aged	6.5
60-69	Aged	6.8

60-69	Aged	6.5
30-39	Young	6.7
30-39	Young	6.8
60-69	Aged	7.1
60-69	Aged	6.7
60-69	Aged	6.9
40-49	Young	6.6
40-49	Young	8.2
40-49	Young	6.7
40-49	Young	5.9
70-79	Aged	5.8
70-79	Aged	6.4
70-79	Aged	6.3
50-59	Young	7.3
50-59	Young	7.1
50-59	Young	6.1
50-59	Young	7
50-59	Young	7.1
50-59	Young	6.5
50-59	Young	6.5
50-59	Young	6.3
60-69	Aged	6.9
40-49	Young	6.2
40-49	Young	6.2
70-79	Aged	6.6
70-79	Aged	5.7
70-79	Aged	6.7
60-69	Aged	6.6
60-69	Aged	6.8
60-69	Aged	6.4
60-69	Aged	7.4
60-69	Aged	6.3
60-69	Aged	5.9
60-69	Aged	6
60-69	Aged	5.7
60-69	Aged	7.9
60-69	Aged	6.9
60-69	Aged	6.9
60-69	Aged	6.3
60-69	Aged	6.9
60-69	Aged	6.6
50-59	Young	6.1
50-59	Young	6.6

50-59	Young	7.2
50-59	Young	6.3
60-69	Aged	6.1
60-69	Aged	5.9
70-79	Aged	5.7
50-59	Young	5.9
60-69	Aged	6.2
60-69	Aged	6.4
60-69	Aged	7
60-69	Aged	6.2
60-69	Aged	6.5
60-69	Aged	7.3
60-69	Aged	6.6
60-69	Aged	7
60-69	Aged	7
50-59	Young	5.9
50-59	Young	5.1
60-69	Aged	6
60-69	Aged	7
60-69	Aged	8.9
40-49	Young	6.5
40-49	Young	9.4
40-49	Young	5.9
40-49	Young	6.1
40-49	Young	7.2
60-69	Aged	6.4
60-69	Aged	6
60-69	Aged	5
50-59	Young	7.2
60-69	Aged	6.3
40-49	Young	6.5
60-69	Aged	6.4
40-49	Young	7.1
60-69	Aged	5.4
60-69	Aged	6.7
40-49	Young	7.7
40-49	Young	6
40-49	Young	8.8
40-49	Young	8.1
30-39	Young	7.3
30-39	Young	6.5
30-39	Young	7.1
30-39	Young	8.9

50-59	Young	7.3
50-59	Young	6.6
50-59	Young	6.9
50-59	Young	7.3
50-59	Young	6.4
60-69	Aged	6.9
60-69	Aged	7.7
60-69	Aged	6.1
60-69	Aged	6.8
20-29	Young	7.3
20-29	Young	6.9
50-59	Young	6.6
50-59	Young	6.2
50-59	Young	6.2
50-59	Young	7.7
50-59	Young	6.1
50-59	Young	6.4
50-59	Young	6.1
50-59	Young	7.7
50-59	Young	6.2
20-29	Young	6
20-29	Young	7.2
20-29	Young	7
20-29	Young	5.9
20-29	Young	9.1
20-29	Young	8
20-29	Young	8.6
50-59	Young	7.4
50-59	Young	7
50-59	Young	8
50-59	Young	6.1
60-69	Aged	7.3
60-69	Aged	6.9
60-69	Aged	7.2
60-69	Aged	6.8
60-69	Aged	7.2
60-69	Aged	8.7
20-29	Young	6.7
20-29	Young	7.1
20-29	Young	7.4
20-29	Young	7.9
50-59	Young	7.5
50-59	Young	7

50-59	Young	6.8
50-59	Young	7.3
50-59	Young	7
50-59	Young	6.8
50-59	Young	7.3
40-49	Young	7
40-49	Young	7.9
40-49	Young	7.9
50-59	Young	6.3
60-69	Aged	7.6
60-69	Aged	7
60-69	Aged	5.9
50-59	Young	7.7
50-59	Young	6.5
50-59	Young	7.6
60-69	Aged	6
50-59	Young	7.9
50-59	Young	5.9
60-69	Aged	6.5
60-69	Aged	8.2
60-69	Aged	7.1
40-49	Young	6.9
40-49	Young	7.2
40-49	Young	7.9
40-49	Young	6.3
60-69	Aged	7.3
60-69	Aged	6.3
60-69	Aged	6.2
50-59	Young	8.2
50-59	Young	7.7
50-59	Young	6.6
40-49	Young	8.2
40-49	Young	6.2
40-49	Young	8.2
50-59	Young	6.3
40-49	Young	6.1
60-69	Aged	6.5
60-69	Aged	6
60-69	Aged	6.2
60-69	Aged	5.7
50-59	Young	5.8
50-59	Young	6
60-69	Aged	6.1

60-69	Aged	6.3
40-49	Young	6.4
40-49	Young	7.5
40-49	Young	8.9
50-59	Young	6.1
50-59	Young	6.3
60-69	Aged	6.9
60-69	Aged	6.9
50-59	Young	7.8
60-69	Aged	7.9
60-69	Aged	7.1
60-69	Aged	6.7
50-59	Young	6.7
60-69	Aged	8
30-39	Young	6.8
60-69	Aged	6.8
50-59	Young	6.2

Supplementary Table 3: A full list of the anatomical regions segmented in the modified Allen brain atlas

Structure	right label	left label
Tuberomammillary nucleus, ventral part	43690	1
internal capsule	43692	3
Principal sensory nucleus of the trigeminal	43693	4
Parataenial nucleus	43697	8
Anterior amygdalar area	43701	12
Superior colliculus	43702	13
Intergeniculate leaflet of the lateral geniculate complex	43703	14
Periventricular hypothalamic nucleus	43705	16
Oculomotor nucleus	43707	18
Gustatory areas	43708	19
Paraventricular hypothalamic nucleus	43709	20
Precommissural nucleus	43712	23
medial forebrain bundle	43714	25
Nucleus accumbens	43715	26
Medial terminal nucleus of the accessory optic tract	43716	27
medial longitudinal fascicle	43718	29
Anterodorsal nucleus	43720	31
Lateral terminal nucleus of the accessory optic tract	43721	32
Interstitial nucleus of Cajal	43722	33
Frontal pole	43723	34
Anterodorsal preoptic nucleus	43724	35
Dorsal terminal nucleus of the accessory optic tract	43726	37
middle cerebellar peduncle	43727	38
lateral ventricle	43728	39
Inferior olivary complex	43729	40
Anterior hypothalamic nucleus	43731	42
Interposed nucleus	43732	43
motor root of the trigeminal nerve	43733	44
Dorsal cochlear nucleus	43734	45
Temporal association areas	43735	46
subependymal zone	43736	47
Ventral cochlear nucleus	43738	49
nigrostriatal tract	43739	50
Superior olivary complex, medial part	43740	51

Inferior salivatory nucleus	43741	52
choroid plexus	43742	53
Superior olivary complex, lateral part	43744	55
Trochlear nucleus	43745	56
Agranular insular area, posterior part	43748	59
Superior olivary complex, periolivary region	43750	61
Koelliker-Fuse subnucleus	43751	62
optic tract	43752	63
Midbrain reticular nucleus	43754	65
Lateral amygdalar nucleus	43756	67
Intermediate reticular nucleus	43759	70
Nucleus ambiguus, ventral division	43762	73
Pontine reticular nucleus	43764	75
Locus ceruleus	43765	76
Paraventricular nucleus of the thalamus	43767	78
lateral recess	43768	79
Lateral dorsal nucleus of thalamus	43769	80
Anterior olfactory nucleus	43772	83
Laterodorsal tegmental nucleus	43773	84
Nucleus prepositus	43775	87
Prelimbic area	43776	88
Retrochiasmatic area	43777	89
Nucleus of Roller	43778	90
Ventral part of the lateral geniculate complex	43779	91
Nucleus of reuniens	43781	93
Lateral habenula	43782	94
Accessory olfactory bulb	43784	96
pyramid	43786	98
Lateral hypothalamic area	43787	99
Medial vestibular nucleus	43792	104
Linear nucleus of the medulla	43793	105
Area postrema	43796	108
Lateral vestibular nucleus	43797	109
Lateral mammillary nucleus	43798	110
Red nucleus	43800	112
Anterior pretectal nucleus	43801	113
Superior vestibular nucleus	43802	114
Lateral posterior nucleus of the thalamus	43803	115
Arcuate hypothalamic nucleus	43805	117
Spinal vestibular nucleus	43806	118

Lateral preoptic area	43807	119
sensory root of the trigeminal nerve	43808	120
Anterior tegmental nucleus	43810	122
solitary tract	43813	125
Midbrain reticular nucleus, retrorubral area	43816	128
Lateral septal nucleus, caudal (caudodorsal) part	43818	130
Anteroventral nucleus of thalamus	43821	133
Lateral septal nucleus, rostral (rostroventral) part	43823	135
Reticular nucleus of the thalamus	43825	137
Anteroventral preoptic nucleus	43826	138
Lateral septal nucleus, ventral part	43827	139
Nucleus sagulum	43830	142
Anteroventral periventricular nucleus	43831	143
Barrington's nucleus	43834	146
Anteromedial visual area	43835	147
Suprachiasmatic nucleus	43836	148
Bed nucleus of the anterior commissure	43837	149
Bed nucleus of the accessory olfactory tract	43840	152
Magnocellular nucleus	43842	154
stria terminalis	43843	155
Basolateral amygdalar nucleus, anterior part	43844	156
Magnocellular reticular nucleus	43847	159
Basolateral amygdalar nucleus, posterior part	43849	161
Supragenual nucleus	43852	164
Primary motor area	43853	165
Subgeniculate nucleus	43854	166
Suprageniculate nucleus	43855	167
superior cerebelar peduncles	43856	168
Basomedial amygdalar nucleus, anterior part	43857	169
Accessory supraoptic group	43860	172
Septohippocampal nucleus	43861	173
Basomedial amygdalar nucleus, posterior part	43862	174
Substantia innominata	43866	178
Subparaventricular zone	43868	180
supraoptic commissures	43869	181
Subceruleus nucleus	43870	182
Bed nuclei of the stria terminalis	43871	183
Preparasubthalamic nucleus	43874	186
Sublaterodorsal nucleus	43875	187
Mediodorsal nucleus of thalamus	43876	188

Parasubthalamic nucleus	43878	190
Submedial nucleus of the thalamus	43879	191
Infracerebellar nucleus	43881	193
Substantia nigra, compact part	43882	194
cuneate fascicle	43884	196
Substantia nigra, reticular part	43885	197
Field CA1	43886	198
Supraoptic nucleus	43887	199
Medial amygdalar nucleus	43891	203
vestibular nerve	43893	205
Subparafascicular nucleus	43894	206
Lateral visual area	43895	207
Field CA2	43897	209
medial corticohypothalamic tract	43899	211
Spinal nucleus of the trigeminal, caudal part	43900	212
columns of the fornix	43904	216
Spinal nucleus of the trigeminal, interpolar part	43905	217
Retrosplenial area, dorsal part	43908	220
Spinal nucleus of the trigeminal, oral part	43910	222
Orbital area, lateral part	43911	223
Primary somatosensory area, upper limb	43913	225
Basolateral amygdalar nucleus, ventral part	43914	226
Midbrain trigeminal nucleus	43917	229
Field CA3	43919	231
alveus	43920	232
Subthalamic nucleus	43922	234
brachium of the inferior colliculus	43925	237
Medial habenula	43926	238
Orbital area, medial part	43927	239
Subiculum	43930	242
dorsal acoustic stria	43931	243
Main olfactory bulb	43932	244
Medial preoptic nucleus	43935	247
Medial preoptic area	43937	249
Supramammillary nucleus	43938	250
Entorhinal area, medial part, dorsal zone	43939	251
Dorsal auditory area	43940	252
Medial pretectal area	43942	254
Supratrigeminal nucleus	43943	255
dorsal limb	43944	256

Perirhinal area	43945	257
Retrosplenial area, ventral part	43946	258
Central amygdalar nucleus	43948	260
dorsal spinocerebellar tract	43951	263
Primary somatosensory area, nose	43953	265
Postpiriform transition area	43957	269
Tegmental reticular nucleus	43959	271
Central lateral nucleus of the thalamus	43960	272
Accessory facial motor nucleus	43961	273
external capsule	43963	275
Nucleus of the brachium of the inferior colliculus	43964	276
Claustrum	43967	279
Nucleus of Darkschewitsch	43968	280
Anterior cingulate area, ventral part	43969	281
Primary visual area	43972	284
fasciculus retroflexus	43973	285
Diagonal band nucleus	43974	286
Taenia tecta, dorsal part	43975	287
Central medial nucleus of the thalamus	43977	289
fimbria	43980	292
Nucleus incertus	43981	293
Taenia tecta, ventral part	43982	294
Subparafascicular area	43984	296
Nucleus of the lateral lemniscus	43987	299
Tuberal nucleus	43989	301
Cuneiform nucleus	43990	302
Motor nucleus of trigeminal	43992	304
Nucleus of the optic tract	43995	307
Ventral anterior-lateral complex of the thalamus	43996	308
Dentate gyrus	43998	310
Nucleus of the posterior commissure	44000	312
Cortical amygdalar area, anterior part	44002	314
Nucleus of the trapezoid body	44003	315
Nucleus of the solitary tract	44007	319
Abducens nucleus	44008	320
Cortical amygdalar area, posterior part, lateral zone	44010	322
Secondary motor area	44011	323
lateral lemniscus	44013	325
Facial motor nucleus	44014	326
Cortical amygdalar area, posterior part, medial zone	44016	328

lateral olfactory tract, body	44018	330
Retrosplenial area, lateral agranular part	44021	333
Caudoputamen	44022	334
mammillary peduncle	44023	335
Superior central nucleus raphe	44026	338
mammillotegmental tract	44028	340
Ventral medial nucleus of the thalamus	44029	341
Ventrolateral preoptic nucleus	44031	343
mammillothalamic tract	44032	344
Ventromedial hypothalamic nucleus	44034	346
Posterior auditory area	44036	348
medial lemniscus	44037	349
Cortical subplate	44041	353
Agranular insular area, ventral part	44042	354
Olivary pretectal nucleus	44043	355
Infralimbic area	44044	356
Cuneate nucleus	44045	357
Ventral posterolateral nucleus of the thalamus	44046	358
Ventral posteromedial nucleus of the thalamus	44053	365
Primary auditory area	44054	366
Ventral tegmental area	44058	370
Posterolateral visual area	44059	371
principal mammillary tract	44060	372
Olfactory tubercle	44061	373
Ventral tegmental nucleus	44063	375
Nucleus x	44066	378
Posterior amygdalar nucleus	44073	385
Nucleus y	44074	386
corticospinal tract	44076	388
Piriform-amygdalar area	44078	390
spinal tract of the trigeminal nerve	44080	392
Zona incerta	44082	394
facial nerve	44083	395
stria medullaris	44085	397
Fields of Forel	44087	399
posteromedial visual area	44088	400
Inferior colliculus	44091	403
Dorsal peduncular area	44093	405
Dorsomedial nucleus of the hypothalamus	44100	412
oculomotor nerve	44102	414

Ectorhinal area	44104	416
Dorsal motor nucleus of the vagus nerve	44106	418
trapezoid body	44107	419
Parasubiculum	44109	421
Dentate nucleus	44111	423
optic nerve	44113	425
uncinate fascicle	44115	427
Parvicellular reticular nucleus	44117	429
Parasolitary nucleus	44120	432
rubrospinal tract	44122	434
ventral spinocerebellar tract	44123	435
Parabrachial nucleus	44124	436
Dorsal nucleus raphe	44126	438
Supplemental somatosensory area	44127	439
Parabigeminal nucleus	44128	440
Primary somatosensory area, mouth	44129	441
Dorsal tegmental nucleus	44130	442
amygdalar capsule	44132	444
Visceral area	44136	448
Pontine central gray	44137	449
External cuneate nucleus	44140	452
Paracentral nucleus	44143	455
trochlear nerve	44146	458
Lingula (I)	44147	459
Posterodorsal preoptic nucleus	44148	460
brachium of the superior colliculus	44149	461
cerebal peduncle	44151	463
Parafascicular nucleus	44154	466
Pontine gray	44155	467
Anterior cingulate area	44156	468
Nucleus ambiguus, dorsal division	44158	470
cingulum bundle	44159	471
Posterior hypothalamic nucleus	44163	475
vomeronasal nerve	44164	476
Endopiriform nucleus, dorsal part	44167	479
Lateral reticular nucleus, magnocellular part	44169	481
Ventral auditory area	44172	484
Piriform area	44173	485
Lateral reticular nucleus, parvicellular part	44175	487
Endopiriform nucleus, ventral part	44178	490

Orbital area, ventrolateral part	44180	492
Paragigantocellular reticular nucleus, dorsal part	44181	493
Paragigantocellular reticular nucleus, lateral part	44188	500
Dorsal premammillary nucleus	44189	501
Primary somatosensory area, barrel field	44190	502
Fasciola cinerea	44191	503
Fastigial nucleus	44195	507
Agranular insular area, dorsal part	44197	509
Fundus of striatum	44199	511
Ventral premammillary nucleus	44200	512
Primary somatosensory area, trunk	44202	514
Simple lobule	44203	515
olfactory nerve layer of main olfactory bulb	44207	519
Posterior complex of the thalamus	44208	520
Globus pallidus, external segment	44210	522
Paramedian lobule	44212	524
Posterior limiting nucleus of the thalamus	44214	526
Primary somatosensory area, lower limb	44215	527
Globus pallidus, internal segment	44216	528
Copula pyramidis	44217	529
Postsubiculum	44219	531
Gracile nucleus	44221	533
Paraflocculus	44222	534
crossed tectospinal pathway	44223	535
Peripeduncular nucleus	44224	536
Gigantocellular reticular nucleus	44228	540
Flocculus	44229	541
Pedunculopontine nucleus	44231	542
Crus 1	44233	544
Posterior pretectal nucleus	44236	547
Crus 2	44238	549
Parapyramidal nucleus	44240	551
Medial geniculate complex, dorsal part	44242	553
Anterolateral visual area	44243	554
Perireunensis nucleus	44244	555
Medial geniculate complex, ventral part	44245	556
Presubiculum	44247	558
Medial geniculate complex, medial part	44250	561
Hippocampal formation	44251	562
external medullary lamina of the thalamus	44254	565

Pontine reticular nucleus, caudal part	44255	566
Anteromedial nucleus, dorsal part	44257	568
Medullary reticular nucleus, dorsal part	44259	570
Anteromedial nucleus, ventral part	44262	573
Intercalated amygdalar nucleus	44263	574
Medullary reticular nucleus, ventral part	44265	576
Parastrial nucleus	44267	578
Interanterodorsal nucleus of the thalamus	44269	580
Entorhinal area, lateral part	44273	584
inferior cerebellar peduncle	44274	585
Tuberomammillary nucleus, dorsal part	44276	587
Nucleus of the lateral olfactory tract	44279	590
Primary somatosensory area, unassigned	44283	594
Anterior area	44289	600
Laterointermediate area	44295	606
Rostrolateral area	44301	612
Postrhinal area	44307	618
Prosubiculum	44313	624
Area prostriata	44314	625
supra-callosal cerebral white matter	44315	626
optic radiation	44317	628
auditory radiation	44318	629
commissural branch of stria terminalis	44319	630
Dorsal part of the lateral geniculate complex	44321	632
Retroparafascicular nucleus	44327	638
Medial accesory oculomotor nucleus	44328	639
Peritrigeminal zone	44329	640
Accessory trigeminal nucleus	44330	641
Parvicellular motor 5 nucleus	44331	642
Intertrigeminal nucleus	44332	643
Ethmoid nucleus of the thalamus	44333	644
Xiphoid thalamic nucleus	44334	645
Posterior intralaminar thalamic nucleus	44335	646
Posterior triangular thalamic nucleus	44336	647
Intermediate geniculate nucleus	44337	648
Ventromedial preoptic nucleus	44338	649
Perifornical nucleus	44339	650
Hippocampo-amygdalar transition area	44340	651
Paratrigeminal nucleus	44341	652
Vestibulocerebellar nucleus	44342	653

Posterodorsal tegmental nucleus	44344	655
Medial mammillary nucleus	44348	659
Paratrochlear nucleus	44349	660
Paranigral nucleus	44350	661
Supraoculomotor periaqueductal gray	44359	670
Interfascicular nucleus raphe	21846	21846
Induseum griseum	21848	21848
Intermediodorsal nucleus of the thalamus	21853	21853
Interpeduncular nucleus	21856	21856
optic chiasm	21858	21858
third ventricle	21861	21861
cerebral aqueduct	21863	21863
fourth ventricle	21864	21864
posterior commissure	21866	21866
central canal, spinal cord/medulla	21868	21868
Rhomboid nucleus	21873	21873
Rostral linear nucleus raphe	21874	21874
pyramidal decussation	21875	21875
Nucleus raphe magnus	21876	21876
Nucleus raphe obscurus	21878	21878
Nucleus raphe pallidus	21880	21880
Nucleus raphe pontis	21881	21881
Septofimbrial nucleus	21884	21884
superior colliculus commissure	21886	21886
Subfornical organ	21887	21887
dorsal hippocampal commissure	21891	21891
ventral hippocampal commissure	21892	21892
Median preoptic nucleus	21893	21893
dorsal fornix	21898	21898
Medial septal nucleus	21901	21901
Triangular nucleus of septum	21902	21902
Central linear nucleus raphe	21904	21904
habenular commissure	21911	21911
inferior colliculus commissure	21912	21912
arbor vitae	21918	21918
cerebellar commissure	21920	21920
Vascular organ of the lamina terminalis	21921	21921
Hypoglossal nucleus	21923	21923
Periaqueductal gray	21924	21924
superior cerebellar peduncle decussation	21925	21925

anterior commissure	21933	21933
Declive (VI)	21937	21937
Folium-tuber vermis (VII)	21938	21938
Pyramus (VIII)	21940	21940
Uvula (IX)	21941	21941
Nodulus (X)	21942	21942
Edinger-Westphal nucleus	21943	21943
Lobule II	21944	21944
Lobule III	21945	21945
root	21947	21947
fiber tracts	21948	21948
doral tegmental decussation	21951	21951
Lobules IV-V	21952	21952
Interanteromedial nucleus of the thalamus	21955	21955
Median eminence	21956	21956
corpus callosum	21957	21957
Subcommissural organ	21959	21959
ventral tegmental decussation	21965	21965

Supplementary Table 4

Tissue	Gene	Sex	Age	r	p	FDR
Cortex	AHSA1	Female	Aged	0.801678	5.95E-08	5.44E-07
Cortex	AHSA1	Male	Aged	0.622588	1.07E-06	7.03E-06
Cortex	AHSA1	Female	Young	0.583773	0.00434	0.010787
Cortex	AHSA1	Male	Young	0.67189	3.55E-10	5.35E-09
Substantia nigra	AHSA1	Female	Aged	0.1444	0.637883	0.745653
Substantia nigra	AHSA1	Male	Aged	0.475834	0.012116	0.026286
Substantia nigra	AHSA1	Female	Young	0.744607	0.002251	0.005881
Substantia nigra	AHSA1	Male	Young	0.3932	0.063435	0.116829
Putamen	AHSA1	Female	Aged	0.856728	2.26E-05	9.79E-05
Putamen	AHSA1	Male	Aged	0.418129	0.011159	0.024851
Putamen	AHSA1	Female	Young	0.518991	0.039399	0.075269
Putamen	AHSA1	Male	Young	0.789463	5.20E-10	7.39E-09
Amygdala	AHSA1	Female	Aged	0.535101	0.022122	0.045305
Amygdala	AHSA1	Male	Aged	0.617745	0.001001	0.002815
Amygdala	AHSA1	Female	Young	0.14955	0.625807	0.734893
Amygdala	AHSA1	Male	Young	0.341152	0.060355	0.111964
Cortex	BAG3	Female	Aged	-0.24707	0.180247	0.284835
Cortex	BAG3	Male	Aged	-0.13841	0.332737	0.481246
Cortex	BAG3	Female	Young	-0.20688	0.355609	0.498672
Cortex	BAG3	Male	Young	-0.08296	0.501201	0.622852
Substantia nigra	BAG3	Female	Aged	-0.27245	0.367823	0.503544
Substantia nigra	BAG3	Male	Aged	0.006989	0.972402	0.987837
Substantia nigra	BAG3	Female	Young	-0.24599	0.396594	0.528792
Substantia nigra	BAG3	Male	Young	-0.12222	0.57851	0.695298
Putamen	BAG3	Female	Aged	0.022054	0.935388	0.969471
Putamen	BAG3	Male	Aged	-0.01428	0.934117	0.969471
Putamen	BAG3	Female	Young	0.012822	0.962411	0.981583
Putamen	BAG3	Male	Young	-0.12312	0.437274	0.562524
Amygdala	BAG3	Female	Aged	-0.13715	0.587363	0.70264
Amygdala	BAG3	Male	Aged	-0.17946	0.390704	0.526639
Amygdala	BAG3	Female	Young	-0.38394	0.195263	0.306131
Amygdala	BAG3	Male	Young	-0.22111	0.231956	0.353457
Cortex	CDC37	Female	Aged	0.167579	0.367537	0.503544
Cortex	CDC37	Male	Aged	0.682148	3.54E-08	3.49E-07
Cortex	CDC37	Female	Young	0.691122	0.000369	0.001194
Cortex	CDC37	Male	Young	0.406951	0.000573	0.00179
Substantia nigra	CDC37	Female	Aged	0.784006	0.001514	0.004167
Substantia nigra	CDC37	Male	Aged	0.721295	2.18E-05	9.64E-05
Substantia nigra	CDC37	Female	Young	0.746797	0.002149	0.005731
Substantia nigra	CDC37	Male	Young	0.740952	5.25E-05	0.000207
Putamen	CDC37	Female	Aged	0.864958	1.53E-05	7.23E-05
Putamen	CDC37	Male	Aged	0.522386	0.001082	0.003012
Putamen	CDC37	Female	Young	0.5071	0.044979	0.085293
Putamen	CDC37	Male	Young	0.82529	1.78E-11	3.50E-10
Amygdala	CDC37	Female	Aged	0.818055	3.35E-05	0.000136

Amygdala	CDC37	Male	Aged	0.76383	8.87E-06	4.45E-05
Amygdala	CDC37	Female	Young	0.559866	0.046618	0.087752
Amygdala	CDC37	Male	Young	0.681018	2.48E-05	0.000106
Cortex	DNAJB1	Female	Aged	0.00234	0.990034	0.996023
Cortex	DNAJB1	Male	Aged	-0.08427	0.556596	0.675302
Cortex	DNAJB1	Female	Young	-0.1143	0.612508	0.725935
Cortex	DNAJB1	Male	Young	0.001274	0.991774	0.996023
Substantia nigra	DNAJB1	Female	Aged	-0.24826	0.413447	0.542782
Substantia nigra	DNAJB1	Male	Aged	0.124678	0.535509	0.655935
Substantia nigra	DNAJB1	Female	Young	-0.34769	0.223179	0.342118
Substantia nigra	DNAJB1	Male	Young	0.099675	0.65091	0.753995
Putamen	DNAJB1	Female	Aged	0.076997	0.776851	0.868445
Putamen	DNAJB1	Male	Aged	0.047957	0.781205	0.869515
Putamen	DNAJB1	Female	Young	-0.01913	0.943938	0.974388
Putamen	DNAJB1	Male	Young	0.031046	0.845254	0.916351
Amygdala	DNAJB1	Female	Aged	0.042656	0.866537	0.924306
Amygdala	DNAJB1	Male	Aged	-0.20657	0.321817	0.473477
Amygdala	DNAJB1	Female	Young	-0.39809	0.17791	0.284656
Amygdala	DNAJB1	Male	Young	-0.18219	0.32661	0.475069
Cortex	FKBP5	Female	Aged	-0.42375	0.017524	0.037385
Cortex	FKBP5	Male	Aged	-0.35433	0.01074	0.024332
Cortex	FKBP5	Female	Young	-0.3367	0.125465	0.224609
Cortex	FKBP5	Male	Young	-0.48692	2.55E-05	0.000107
Substantia nigra	FKBP5	Female	Aged	0.173131	0.571643	0.690286
Substantia nigra	FKBP5	Male	Aged	0.105223	0.601436	0.716129
Substantia nigra	FKBP5	Female	Young	-0.3523	0.216694	0.334179
Substantia nigra	FKBP5	Male	Young	-0.18733	0.392047	0.526639
Putamen	FKBP5	Female	Aged	-0.05732	0.833002	0.916351
Putamen	FKBP5	Male	Aged	0.472582	0.003609	0.00924
Putamen	FKBP5	Female	Young	0.189345	0.48247	0.602499
Putamen	FKBP5	Male	Young	0.132593	0.402552	0.533956
Amygdala	FKBP5	Female	Aged	0.339954	0.1675	0.27312
Amygdala	FKBP5	Male	Aged	-0.01295	0.95102	0.977756
Amygdala	FKBP5	Female	Young	0.060556	0.844207	0.916351
Amygdala	FKBP5	Male	Young	-0.07146	0.702465	0.806418
Cortex	HSP90AA1	Female	Aged	0.449602	0.011163	0.024851
Cortex	HSP90AA1	Male	Aged	0.45673	0.000755	0.002246
Cortex	HSP90AA1	Female	Young	-0.04105	0.856054	0.916945
Cortex	HSP90AA1	Male	Young	0.588624	1.30E-07	1.07E-06
Substantia nigra	HSP90AA1	Female	Aged	0.001734	0.995513	0.996023
Substantia nigra	HSP90AA1	Male	Aged	0.608146	0.000765	0.00225
Substantia nigra	HSP90AA1	Female	Young	0.600215	0.023246	0.04723
Substantia nigra	HSP90AA1	Male	Young	0.477449	0.021227	0.04418
Putamen	HSP90AA1	Female	Aged	0.647494	0.006693	0.015719
Putamen	HSP90AA1	Male	Aged	0.319878	0.057191	0.106869
Putamen	HSP90AA1	Female	Young	0.389702	0.135674	0.235177
Putamen	HSP90AA1	Male	Young	0.680584	7.09E-07	5.06E-06

Amygdala	HSP90AA1	Female	Aged	0.3176	0.199041	0.308816
Amygdala	HSP90AA1	Male	Aged	0.418662	0.037258	0.071715
Amygdala	HSP90AA1	Female	Young	-0.23686	0.435896	0.562524
Amygdala	HSP90AA1	Male	Young	0.152942	0.411419	0.542782
Cortex	HSP90AB1	Female	Aged	0.415542	0.020082	0.04214
Cortex	HSP90AB1	Male	Aged	0.779474	1.60E-11	3.42E-10
Cortex	HSP90AB1	Female	Young	0.662024	0.00079	0.002299
Cortex	HSP90AB1	Male	Young	0.767174	2.36E-14	1.51E-12
Substantia nigra	HSP90AB1	Female	Aged	0.286926	0.341867	0.49029
Substantia nigra	HSP90AB1	Male	Aged	0.75166	6.20E-06	3.24E-05
Substantia nigra	HSP90AB1	Female	Young	0.871965	4.80E-05	0.000192
Substantia nigra	HSP90AB1	Male	Young	0.835208	7.12E-07	5.06E-06
Putamen	HSP90AB1	Female	Aged	0.915836	6.41E-07	4.82E-06
Putamen	HSP90AB1	Male	Aged	0.416941	0.011415	0.025191
Putamen	HSP90AB1	Female	Young	0.528942	0.035144	0.068678
Putamen	HSP90AB1	Male	Young	0.803949	1.44E-10	2.46E-09
Amygdala	HSP90AB1	Female	Aged	0.616423	0.006442	0.01527
Amygdala	HSP90AB1	Male	Aged	0.716116	5.68E-05	0.00022
Amygdala	HSP90AB1	Female	Young	0.428359	0.144192	0.244559
Amygdala	HSP90AB1	Male	Young	0.651709	7.14E-05	0.000269
Cortex	HSPA1A	Female	Aged	-0.20342	0.272397	0.403085
Cortex	HSPA1A	Male	Aged	-0.18403	0.196115	0.306131
Cortex	HSPA1A	Female	Young	-0.17149	0.445411	0.564481
Cortex	HSPA1A	Male	Young	-0.09311	0.450141	0.564883
Substantia nigra	HSPA1A	Female	Aged	-0.28421	0.346651	0.49029
Substantia nigra	HSPA1A	Male	Aged	0.120791	0.548404	0.66853
Substantia nigra	HSPA1A	Female	Young	-0.4056	0.150192	0.252955
Substantia nigra	HSPA1A	Male	Young	-0.1012	0.645894	0.751585
Putamen	HSPA1A	Female	Aged	0.08314	0.759519	0.852793
Putamen	HSPA1A	Male	Aged	-0.00862	0.960186	0.981583
Putamen	HSPA1A	Female	Young	-0.02965	0.913194	0.962048
Putamen	HSPA1A	Male	Young	-0.0555	0.726999	0.830856
Amygdala	HSPA1A	Female	Aged	-0.19681	0.433765	0.562524
Amygdala	HSPA1A	Male	Aged	-0.23866	0.250603	0.377378
Amygdala	HSPA1A	Female	Young	-0.43414	0.138267	0.237559
Amygdala	HSPA1A	Male	Young	-0.25616	0.164233	0.269511
Cortex	HSPA1B	Female	Aged	-0.17143	0.356472	0.498672
Cortex	HSPA1B	Male	Aged	-0.12221	0.392922	0.526639
Cortex	HSPA1B	Female	Young	-0.21968	0.325953	0.475069
Cortex	HSPA1B	Male	Young	-0.03231	0.793675	0.87957
Substantia nigra	HSPA1B	Female	Aged	-0.26612	0.379485	0.516745
Substantia nigra	HSPA1B	Male	Aged	0.160964	0.422514	0.551855
Substantia nigra	HSPA1B	Female	Young	-0.31675	0.269859	0.401651
Substantia nigra	HSPA1B	Male	Young	0.041205	0.85192	0.916351
Putamen	HSPA1B	Female	Aged	0.084162	0.756645	0.852793
Putamen	HSPA1B	Male	Aged	0.023703	0.890856	0.946304
Putamen	HSPA1B	Female	Young	-0.05434	0.841582	0.916351

Putamen	HSPA1B	Male	Young	0.000793	0.996023	0.996023
Amygdala	HSPA1B	Female	Aged	-0.12353	0.625302	0.734893
Amygdala	HSPA1B	Male	Aged	-0.24033	0.247186	0.374436
Amygdala	HSPA1B	Female	Young	-0.4283	0.144252	0.244559
Amygdala	HSPA1B	Male	Young	-0.17621	0.343025	0.49029
Cortex	HSPB1	Female	Aged	-0.41024	0.021893	0.045198
Cortex	HSPB1	Male	Aged	-0.25279	0.073499	0.134398
Cortex	HSPB1	Female	Young	-0.55851	0.0069	0.016059
Cortex	HSPB1	Male	Young	-0.41829	0.000386	0.001235
Substantia nigra	HSPB1	Female	Aged	-0.23238	0.444886	0.564481
Substantia nigra	HSPB1	Male	Aged	0.038356	0.849353	0.916351
Substantia nigra	HSPB1	Female	Young	-0.38858	0.169737	0.275017
Substantia nigra	HSPB1	Male	Young	-0.13797	0.530149	0.652491
Putamen	HSPB1	Female	Aged	0.022195	0.934974	0.969471
Putamen	HSPB1	Male	Aged	0.053618	0.756121	0.852793
Putamen	HSPB1	Female	Young	-0.02646	0.922524	0.967894
Putamen	HSPB1	Male	Young	-0.14947	0.344771	0.49029
Amygdala	HSPB1	Female	Aged	-0.10955	0.665229	0.767111
Amygdala	HSPB1	Male	Aged	-0.15885	0.448196	0.564883
Amygdala	HSPB1	Female	Young	-0.39855	0.177368	0.284656
Amygdala	HSPB1	Male	Young	-0.20534	0.267784	0.400893
Cortex	PPIA	Female	Aged	0.534862	0.001935	0.00527
Cortex	PPIA	Male	Aged	0.781435	1.32E-11	3.07E-10
Cortex	PPIA	Female	Young	0.832488	1.54E-06	8.97E-06
Cortex	PPIA	Male	Young	0.553109	1.00E-06	6.75E-06
Substantia nigra	PPIA	Female	Aged	0.879768	7.33E-05	0.000272
Substantia nigra	PPIA	Male	Aged	0.783333	1.36E-06	8.28E-06
Substantia nigra	PPIA	Female	Young	0.935757	8.83E-07	6.11E-06
Substantia nigra	PPIA	Male	Young	0.790577	7.15E-06	3.66E-05
Putamen	PPIA	Female	Aged	0.818879	0.000105	0.000367
Putamen	PPIA	Male	Aged	0.883218	1.01E-12	2.86E-11
Putamen	PPIA	Female	Young	0.824831	8.42E-05	0.000304
Putamen	PPIA	Male	Young	0.880593	1.50E-14	1.28E-12
Amygdala	PPIA	Female	Aged	0.952047	1.21E-09	1.55E-08
Amygdala	PPIA	Male	Aged	0.951477	2.91E-13	1.24E-11
Amygdala	PPIA	Female	Young	0.907837	1.80E-05	8.07E-05
Amygdala	PPIA	Male	Young	0.839258	3.66E-09	4.33E-08
Cortex	PPP5C	Female	Aged	0.624051	0.000176	0.000608
Cortex	PPP5C	Male	Aged	0.809278	6.62E-13	2.12E-11
Cortex	PPP5C	Female	Young	0.934116	2.12E-10	3.38E-09
Cortex	PPP5C	Male	Young	0.775397	8.32E-15	1.07E-12
Substantia nigra	PPP5C	Female	Aged	0.718664	0.005645	0.013597
Substantia nigra	PPP5C	Male	Aged	0.784701	1.26E-06	8.09E-06
Substantia nigra	PPP5C	Female	Young	0.958712	6.54E-08	5.77E-07
Substantia nigra	PPP5C	Male	Young	0.875666	4.46E-08	4.23E-07
Putamen	PPP5C	Female	Aged	0.864015	1.60E-05	7.43E-05
Putamen	PPP5C	Male	Aged	0.795529	6.72E-09	7.48E-08

Putamen	PPP5C	Female	Young	0.757507	0.000677	0.002038
Putamen	PPP5C	Male	Young	0.856007	5.02E-13	1.83E-11
Amygdala	PPP5C	Female	Aged	0.846837	9.31E-06	4.58E-05
Amygdala	PPP5C	Male	Aged	0.78742	3.00E-06	1.71E-05
Amygdala	PPP5C	Female	Young	0.718279	0.005683	0.013597
Amygdala	PPP5C	Male	Young	0.784015	1.81E-07	1.42E-06
Cortex	PTGES3	Female	Aged	-0.06213	0.739849	0.841784
Cortex	PTGES3	Male	Aged	0.448874	0.000954	0.002713
Cortex	PTGES3	Female	Young	0.311356	0.158395	0.264954
Cortex	PTGES3	Male	Young	0.259907	0.032319	0.063643
Substantia nigra	PTGES3	Female	Aged	0.233774	0.442071	0.564481
Substantia nigra	PTGES3	Male	Aged	0.496361	0.008453	0.019496
Substantia nigra	PTGES3	Female	Young	0.397422	0.159386	0.264954
Substantia nigra	PTGES3	Male	Young	0.339464	0.113035	0.203782
Putamen	PTGES3	Female	Aged	0.863032	1.68E-05	7.66E-05
Putamen	PTGES3	Male	Aged	0.388243	0.019291	0.040813
Putamen	PTGES3	Female	Young	0.527516	0.035731	0.069297
Putamen	PTGES3	Male	Young	0.749942	1.09E-08	1.12E-07
Amygdala	PTGES3	Female	Aged	0.587467	0.01036	0.023679
Amygdala	PTGES3	Male	Aged	0.621652	0.000909	0.002615
Amygdala	PTGES3	Female	Young	0.273182	0.366485	0.503544
Amygdala	PTGES3	Male	Young	0.485934	0.005581	0.013597
Cortex	SGTA	Female	Aged	0.580962	0.00061	0.00186
Cortex	SGTA	Male	Aged	0.715153	3.72E-09	4.33E-08
Cortex	SGTA	Female	Young	0.592355	0.003676	0.009317
Cortex	SGTA	Male	Young	0.582961	1.83E-07	1.42E-06
Substantia nigra	SGTA	Female	Aged	0.646908	0.01686	0.03627
Substantia nigra	SGTA	Male	Aged	0.563	0.002233	0.005881
Substantia nigra	SGTA	Female	Young	0.194634	0.504914	0.624434
Substantia nigra	SGTA	Male	Young	0.289726	0.179933	0.284835
Putamen	SGTA	Female	Aged	0.802981	0.00018	0.000615
Putamen	SGTA	Male	Aged	0.544475	0.000597	0.001841
Putamen	SGTA	Female	Young	0.389439	0.135962	0.235177
Putamen	SGTA	Male	Young	0.711419	1.29E-07	1.07E-06
Amygdala	SGTA	Female	Aged	0.757964	0.000268	0.000891
Amygdala	SGTA	Male	Aged	0.675519	0.000211	0.000711
Amygdala	SGTA	Female	Young	0.618898	0.024116	0.048612
Amygdala	SGTA	Male	Young	0.674386	3.18E-05	0.000131
Cortex	STIP1	Female	Aged	0.591881	0.000453	0.001431
Cortex	STIP1	Male	Aged	0.480954	0.000354	0.00116
Cortex	STIP1	Female	Young	0.330854	0.132585	0.232478
Cortex	STIP1	Male	Young	0.459237	8.18E-05	0.000299
Substantia nigra	STIP1	Female	Aged	0.034783	0.910183	0.962048
Substantia nigra	STIP1	Male	Aged	0.520221	0.005409	0.013315
Substantia nigra	STIP1	Female	Young	0.64924	0.011988	0.026229
Substantia nigra	STIP1	Male	Young	0.449911	0.031234	0.061984
Putamen	STIP1	Female	Aged	0.819104	0.000104	0.000367

Putamen	STIP1	Male	Aged	0.258438	0.128036	0.227334
Putamen	STIP1	Female	Young	0.36536	0.164055	0.269511
Putamen	STIP1	Male	Young	0.636096	5.98E-06	3.19E-05
Amygdala	STIP1	Female	Aged	0.228259	0.362291	0.503544
Amygdala	STIP1	Male	Aged	0.361055	0.076192	0.138335
Amygdala	STIP1	Female	Young	-0.06434	0.834596	0.916351
Amygdala	STIP1	Male	Young	0.278835	0.128764	0.227334
Cortex	STUB1	Female	Aged	0.856309	8.08E-10	1.09E-08
Cortex	STUB1	Male	Aged	0.833872	3.04E-14	1.56E-12
Cortex	STUB1	Female	Young	0.815564	3.75E-06	2.09E-05
Cortex	STUB1	Male	Young	0.794963	5.77E-16	1.48E-13
Substantia nigra	STUB1	Female	Aged	0.763179	0.002405	0.006219
Substantia nigra	STUB1	Male	Aged	0.731734	1.44E-05	6.97E-05
Substantia nigra	STUB1	Female	Young	0.748109	0.00209	0.005631
Substantia nigra	STUB1	Male	Young	0.794878	5.87E-06	3.19E-05
Putamen	STUB1	Female	Aged	0.95364	1.09E-08	1.12E-07
Putamen	STUB1	Male	Aged	0.849736	5.58E-11	1.02E-09
Putamen	STUB1	Female	Young	0.559185	0.024325	0.048649
Putamen	STUB1	Male	Young	0.849129	1.19E-12	3.05E-11
Amygdala	STUB1	Female	Aged	0.800311	6.65E-05	0.000254
Amygdala	STUB1	Male	Aged	0.800603	1.54E-06	8.97E-06
Amygdala	STUB1	Female	Young	0.733472	0.004327	0.010787
Amygdala	STUB1	Male	Young	0.748305	1.30E-06	8.10E-06

Supplementary Table 5: Volumetric statistical results for each structure in the modified AllenBrain Atlas in terms of standardised betas, t-, p- and q-values for each of the comparisons performed (M83+/+:STI1WT versus WT mice, and M83+/+:ΔHET versus M83+/+:STI1WT).

	M83+/+:STI1WT versus WT mice			
**region**	**beta- M83+/+**	**tvalue- M83+/+**	**pvalue-tvalue M83+/+**	**qvalue-tvalue M83+/+**
anterior commissure	-0.15863	-6.38202	0.00000	0.00133
arbor vitae	-0.97405	-5.40741	0.00000	0.00184
central canal, spinal cord/medulla	NA	NA	NA	NA
Central linear nucleus raphe	-0.00795	-3.12174	0.00000	0.03055
cerebellar commissure	-0.00524	-2.12684	0.00000	0.12332
cerebral aqueduct	-0.04047	-1.55645	0.00000	0.26042
corpus callosum	-0.50716	-3.36455	0.00000	0.02158
Declive (VI)	-0.14888	-1.16761	0.00000	0.40275
doral tegmental decussation	-0.00033	-0.76106	0.00000	0.59903
dorsal fornix	0.00126	1.13445	0.00000	0.41606
dorsal hippocampal commissure	-0.12347	-3.82128	0.00000	0.01069
Edinger-Westphal nucleus	-0.00234	-1.28292	0.00000	0.35071
fiber tracts	-0.07455	-2.69079	0.00000	0.05524
Folium-tuber vermis (VII)	-0.14495	-2.38983	0.00000	0.08684
fourth ventricle	-0.00083	-0.06839	0.00009	0.96250
habenular commissure	-0.00092	-0.60376	0.00000	0.67459
Hypoglossal nucleus	-0.02500	-2.76105	0.00000	0.05022
Induseum griseum	-0.00060	-0.13746	0.00001	0.92556
inferior colliculus commissure	0.00041	0.36617	0.00000	0.80077
Interanteromedial nucleus of the thalamus	-0.00551	-3.42544	0.00000	0.01937
Interfascicular nucleus raphe	-0.00696	-2.56334	0.00000	0.06771
Intermediodorsal nucleus of the thalamus	0.00238	0.54391	0.00000	0.70069
Interpeduncular nucleus	-0.02318	-1.95888	0.00000	0.15428
left Abducens nucleus	0.00045	0.96152	0.00000	0.50136
left Accessory facial motor nucleus	-0.00009	-0.34549	0.00000	0.81186
left Accessory olfactory bulb	-0.01824	-2.01386	0.00000	0.14393
left Accessory supraoptic group	NA	NA	NA	NA
left Accessory trigeminal nucleus	-0.00093	-2.19667	0.00000	0.11109
left Agranular insular area, dorsal part	-0.11798	-3.11351	0.00000	0.03055
left Agranular insular area, posterior part	-0.02346	-1.52424	0.00000	0.27032
left Agranular insular area, ventral part	-0.09114	-4.50446	0.00000	0.00469
left alveus	-0.05529	-4.24292	0.00000	0.00579
left amygdalar capsule	-0.00154	-0.73649	0.00000	0.61181

left Anterior amygdalar area	-0.00901	-1.47499	0.00000	0.29113
left Anterior area	-0.04401	-2.28994	0.00000	0.09885
left Anterior cingulate area	0.00604	0.19212	0.00000	0.89744
left Anterior cingulate area, ventral part	0.01409	0.60490	0.00000	0.67459
left Anterior hypothalamic nucleus	-0.00316	-0.40682	0.00000	0.78141
left Anterior olfactory nucleus	-0.22903	-6.16068	0.00000	0.00136
left Anterior pretectal nucleus	-0.06162	-5.46552	0.00000	0.00184
left Anterior tegmental nucleus	-0.00062	-0.95282	0.00000	0.50500
left Anterodorsal nucleus	-0.00277	-1.02872	0.00000	0.46789
left Anterodorsal preoptic nucleus	-0.00297	-2.96108	0.00000	0.03774
left Anterolateral visual area	-0.02115	-1.87053	0.00000	0.17434
left Anteromedial nucleus, dorsal part	-0.00959	-2.05379	0.00000	0.13522
left Anteromedial nucleus, ventral part	-0.00472	-1.61730	0.00000	0.24533
left Anteromedial visual area	-0.01480	-1.45737	0.00000	0.29561
left Anteroventral nucleus of thalamus	-0.00732	-1.10536	0.00000	0.43010
left Anteroventral periventricular nucleus	-0.00174	-0.86269	0.00000	0.55276
left Anteroventral preoptic nucleus	0.00093	0.70673	0.00000	0.62438
left Arcuate hypothalamic nucleus	0.00349	0.86052	0.00000	0.55338
left Area postrema	0.00039	0.20874	0.00000	0.88876
left Area prostriata	-0.01278	-3.34619	0.00000	0.02212
left auditory radiation	-0.00654	-1.19983	0.00000	0.38412
left Barrington's nucleus	0.00031	0.90252	0.00000	0.52988
left Basolateral amygdalar nucleus, anterior part	-0.02315	-3.11786	0.00000	0.03055
left Basolateral amygdalar nucleus, posterior part	-0.02657	-3.36997	0.00000	0.02150
left Basolateral amygdalar nucleus, ventral part	-0.00790	-2.23503	0.00000	0.10665
left Basomedial amygdalar nucleus, anterior part	-0.01435	-1.75270	0.00000	0.20650
left Basomedial amygdalar nucleus, posterior part	-0.02275	-4.27866	0.00000	0.00571
left Bed nuclei of the stria terminalis	-0.04893	-4.02728	0.00000	0.00821
left Bed nucleus of the accessory olfactory tract	-0.00069	-1.31631	0.00000	0.34117
left Bed nucleus of the anterior commissure	-0.00059	-1.98206	0.00000	0.14896
left brachium of the inferior colliculus	-0.01529	-4.29259	0.00000	0.00571
left brachium of the superior colliculus	-0.01490	-5.22194	0.00000	0.00211
left Caudoputamen	-0.59126	-2.58621	0.00000	0.06512
left Central amygdalar nucleus	-0.04639	-4.26803	0.00000	0.00571
left Central lateral nucleus of the thalamus	-0.01532	-2.67348	0.00000	0.05676
left Central medial nucleus of the thalamus	-0.00404	-1.41530	0.00000	0.30927
left cerebal peduncle	-0.02030	-1.86079	0.00000	0.17624
left choroid plexus	-0.01715	-1.08622	0.00000	0.43820
left cingulum bundle	-0.03650	-3.65632	0.00000	0.01432
left Claustrum	-0.01441	-3.68316	0.00000	0.01377

left columns of the fornix	-0.01343	-3.93402	0.00000	0.00912
left commissural branch of stria terminalis	-0.00022	-0.50795	0.00000	0.72185
left Copula pyramidis	-0.17806	-3.87339	0.00000	0.00996
left Cortical amygdalar area, anterior part	-0.00813	-0.89764	0.00000	0.53013
left Cortical amygdalar area, posterior part, lateral zone	-0.03125	-2.41180	0.00000	0.08368
left Cortical amygdalar area, posterior part, medial zone	-0.04011	-3.55075	0.00000	0.01652
left Cortical subplate	-0.00822	-3.54028	0.00000	0.01652
left corticospinal tract	-0.00577	-3.51535	0.00000	0.01682
left crossed tectospinal pathway	-0.01601	-2.81941	0.00000	0.04671
left Crus 1	-0.22588	-2.41838	0.00000	0.08324
left Crus 2	-0.13511	-1.92628	0.00000	0.16119
left cuneate fascicle	-0.00032	-0.52314	0.00000	0.71295
left Cuneate nucleus	0.00102	0.12872	0.00001	0.92797
left Cuneiform nucleus	-0.01881	-2.54121	0.00000	0.07060
left Dentate gyrus	-0.28401	-3.98325	0.00000	0.00870
left Dentate nucleus	-0.01449	-3.64018	0.00000	0.01462
left Diagonal band nucleus	-0.00591	-0.73615	0.00000	0.61181
left dorsal acoustic stria	0.00020	0.36754	0.00000	0.80077
left Dorsal auditory area	-0.03425	-2.34515	0.00000	0.09253
left Dorsal cochlear nucleus	0.00294	0.27547	0.00000	0.85607
left dorsal limb	-0.00648	-1.20558	0.00000	0.38228
left Dorsal motor nucleus of the vagus nerve	-0.00403	-1.35465	0.00000	0.33094
left Dorsal nucleus raphe	-0.00164	-0.85424	0.00000	0.55493
left Dorsal part of the lateral geniculate complex	-0.04061	-4.51871	0.00000	0.00466
left Dorsal peduncular area	-0.02958	-3.74094	0.00000	0.01240
left Dorsal premammillary nucleus	-0.00174	-0.84583	0.00000	0.55930
left dorsal spinocerebellar tract	-0.00069	-0.56586	0.00000	0.69497
left Dorsal tegmental nucleus	0.00087	0.56277	0.00000	0.69497
left Dorsal terminal nucleus of the accessory optic tract	-0.00094	-3.10746	0.00000	0.03056
left Dorsomedial nucleus of the hypothalamus	-0.01462	-3.58503	0.00000	0.01594
left Ectorhinal area	-0.04619	-2.78662	0.00000	0.04923
left Endopiriform nucleus, dorsal part	-0.05981	-4.26802	0.00000	0.00571
left Endopiriform nucleus, ventral part	-0.02239	-3.03041	0.00000	0.03394
left Entorhinal area, lateral part	-0.22773	-4.74324	0.00000	0.00331
left Entorhinal area, medial part, dorsal zone	-0.11865	-2.18693	0.00000	0.11227
left Ethmoid nucleus of the thalamus	-0.00816	-3.04220	0.00000	0.03328
left external capsule	-0.01683	-3.08771	0.00000	0.03129
left External cuneate nucleus	0.00309	0.88791	0.00000	0.53622
left external medullary lamina of the thalamus	-0.00134	-1.05370	0.00000	0.45775
left Facial motor nucleus	0.00370	0.31928	0.00000	0.82862

left facial nerve	-0.00117	-0.93791	0.00000	0.51226
left fasciculus retroflexus	-0.00503	-2.88379	0.00000	0.04271
left Fasciola cinerea	-0.00214	-1.41588	0.00000	0.30927
left Fastigial nucleus	-0.04378	-5.89021	0.00000	0.00155
left Field CA1	-0.28432	-2.83683	0.00000	0.04589
left Field CA2	-0.02221	-4.29722	0.00000	0.00571
left Field CA3	-0.26682	-4.24437	0.00000	0.00579
left Fields of Forel	-0.00579	-2.11645	0.00000	0.12513
left fimbria	-0.01075	-0.65751	0.00000	0.65256
left Flocculus	-0.02899	-1.55321	0.00000	0.26096
left Frontal pole	0.00932	1.12924	0.00000	0.41749
left Fundus of striatum	-0.00310	-0.47755	0.00000	0.73962
left Gigantocellular reticular nucleus	-0.03766	-1.31381	0.00000	0.34117
left Globus pallidus, external segment	-0.04384	-2.71436	0.00000	0.05458
left Globus pallidus, internal segment	-0.00923	-1.76786	0.00000	0.20255
left Gracile nucleus	-0.00135	-0.81305	0.00000	0.57081
left Gustatory areas	-0.02719	-1.95911	0.00000	0.15428
left Hippocampal formation	-0.01282	-3.23963	0.00000	0.02553
left Hippocampo-amygdalar transition area	-0.01160	-2.44769	0.00000	0.07998
left inferior cerebellar peduncle	-0.02660	-2.96718	0.00000	0.03756
left Inferior colliculus	-0.15233	-2.86048	0.00000	0.04441
left Inferior olivary complex	-0.00819	-0.83369	0.00000	0.56428
left Inferior salivatory nucleus	-0.00012	-0.28302	0.00000	0.85123
left Infracerebellar nucleus	-0.00605	-4.27055	0.00000	0.00571
left Infralimbic area	-0.02650	-2.76847	0.00000	0.05017
left Interanterodorsal nucleus of the thalamus	-0.00375	-2.41256	0.00000	0.08368
left Intercalated amygdalar nucleus	-0.00493	-2.19654	0.00000	0.11109
left Intergeniculate leaflet of the lateral geniculate complex	-0.00139	-1.14398	0.00000	0.41325
left Intermediate geniculate nucleus	-0.00099	-2.14542	0.00000	0.11982
left Intermediate reticular nucleus	-0.02403	-0.68594	0.00000	0.63638
left internal capsule	-0.06930	-2.58338	0.00000	0.06522
left Interposed nucleus	-0.05654	-5.33142	0.00000	0.00184
left Interstitial nucleus of Cajal	-0.00181	-2.05335	0.00000	0.13522
left Intertrigeminal nucleus	-0.00038	-0.41738	0.00000	0.77852
left Koelliker-Fuse subnucleus	-0.00831	-3.32786	0.00000	0.02232
left Lateral amygdalar nucleus	-0.00737	-0.51557	0.00000	0.71792
left Lateral dorsal nucleus of thalamus	-0.02667	-1.89585	0.00000	0.16872
left Lateral habenula	-0.00775	-1.31031	0.00000	0.34161
left Lateral hypothalamic area	-0.06340	-2.61865	0.00000	0.06171
left lateral lemniscus	-0.01808	-2.22904	0.00000	0.10718

left Lateral mammillary nucleus	0.00172	0.62959	0.00000	0.66375
left lateral olfactory tract, body	-0.01369	-1.13418	0.00000	0.41606
left Lateral posterior nucleus of the thalamus	-0.07660	-4.90274	0.00000	0.00303
left Lateral preoptic area	-0.01183	-1.76437	0.00000	0.20328
left lateral recess	-0.01259	-2.03503	0.00000	0.13922
left Lateral reticular nucleus, magnocellular part	-0.00599	-0.98680	0.00000	0.49056
left Lateral reticular nucleus, parvicellular part	-0.00111	-1.67104	0.00000	0.23092
left Lateral septal nucleus, caudal (caudodorsal) part	-0.00916	-1.26604	0.00000	0.35657
left Lateral septal nucleus, rostral (rostroventral) part	-0.00570	-0.21542	0.00000	0.88876
left Lateral septal nucleus, ventral part	-0.00635	-0.73912	0.00000	0.61177
left Lateral terminal nucleus of the accessory optic tract	-0.00054	-1.50474	0.00000	0.27813
left lateral ventricle	-0.00228	-0.08408	0.00004	0.95504
left Lateral vestibular nucleus	-0.00317	-0.98119	0.00000	0.49353
left Lateral visual area	-0.01518	-0.80121	0.00000	0.57670
left Laterodorsal tegmental nucleus	-0.00105	-0.50503	0.00000	0.72310
left Laterointermediate area	-0.00289	-0.38582	0.00000	0.79398
left Linear nucleus of the medulla	-0.00093	-0.87035	0.00000	0.54809
left Lingula (I)	0.00024	0.09572	0.00003	0.94696
left Locus ceruleus	0.00000	-0.00001	0.01613	0.99999
left Magnocellular nucleus	-0.00695	-1.08971	0.00000	0.43760
left Magnocellular reticular nucleus	-0.01701	-2.29796	0.00000	0.09799
left Main olfactory bulb	-1.31020	-6.72477	0.00000	0.00133
left mammillary peduncle	-0.00056	-0.73410	0.00000	0.61181
left mammillotegmental tract	-0.00326	-2.21428	0.00000	0.10857
left mammillothalamic tract	-0.00436	-2.52572	0.00000	0.07227
left Medial accesory oculomotor nucleus	-0.00041	-0.39381	0.00000	0.78938
left Medial amygdalar nucleus	-0.06169	-4.23150	0.00000	0.00585
left medial corticohypothalamic tract	-0.00006	-0.19088	0.00000	0.89744
left medial forebrain bundle	-0.00130	-1.54552	0.00000	0.26267
left Medial geniculate complex, dorsal part	-0.00601	-3.33633	0.00000	0.02225
left Medial geniculate complex, medial part	-0.00325	-1.03889	0.00000	0.46588
left Medial geniculate complex, ventral part	-0.00702	-1.98960	0.00000	0.14773
left Medial habenula	-0.00930	-1.43055	0.00000	0.30489
left medial lemniscus	-0.02755	-4.45469	0.00000	0.00471
left medial longitudinal fascicle	-0.00355	-1.81122	0.00000	0.18969
left Medial mammillary nucleus	-0.00625	-0.60186	0.00000	0.67459
left Medial preoptic area	-0.00471	-0.81993	0.00000	0.56825
left Medial preoptic nucleus	-0.00515	-1.64648	0.00000	0.23555
left Medial pretectal area	-0.00044	-0.68849	0.00000	0.63544
left Medial terminal nucleus of the accessory optic tract	-0.00141	-1.88487	0.00000	0.17071

left Medial vestibular nucleus	0.01405	0.69822	0.00000	0.62890
left Mediodorsal nucleus of thalamus	-0.02477	-1.85851	0.00000	0.17624
left Medullary reticular nucleus, dorsal part	-0.02235	-1.33934	0.00000	0.33426
left Medullary reticular nucleus, ventral part	-0.03892	-2.32691	0.00000	0.09418
left Midbrain reticular nucleus	-0.18431	-3.66275	0.00000	0.01426
left Midbrain reticular nucleus, retrorubral area	-0.00357	-2.06127	0.00000	0.13445
left Midbrain trigeminal nucleus	-0.00002	-0.05898	0.00012	0.96691
left middle cerebellar peduncle	-0.02090	-1.92060	0.00000	0.16245
left Motor nucleus of trigeminal	-0.00821	-1.49117	0.00000	0.28394
left motor root of the trigeminal nerve	0.00033	0.35036	0.00000	0.81021
left nigrostriatal tract	-0.00436	-3.42445	0.00000	0.01937
left Nucleus accumbens	-0.18700	-4.26731	0.00000	0.00571
left Nucleus ambiguus, dorsal division	-0.00014	-0.20412	0.00000	0.89034
left Nucleus ambiguus, ventral division	-0.00087	-1.01310	0.00000	0.47613
left Nucleus incertus	0.00015	0.12426	0.00001	0.93031
left Nucleus of Darkschewitsch	-0.00275	-1.58720	0.00000	0.25158
left Nucleus of reuniens	-0.02260	-3.82196	0.00000	0.01069
left Nucleus of Roller	-0.00151	-2.16829	0.00000	0.11536
left Nucleus of the brachium of the inferior colliculus	-0.00226	-1.66899	0.00000	0.23115
left Nucleus of the lateral lemniscus	-0.01435	-1.66312	0.00000	0.23234
left Nucleus of the lateral olfactory tract	-0.00365	-0.93038	0.00000	0.51523
left Nucleus of the optic tract	-0.01198	-4.72997	0.00000	0.00331
left Nucleus of the posterior commissure	-0.00683	-2.33562	0.00000	0.09361
left Nucleus of the solitary tract	-0.00034	-0.03715	0.00056	0.97969
left Nucleus of the trapezoid body	-0.00343	-2.02556	0.00000	0.14134
left Nucleus prepositus	0.00111	0.43909	0.00000	0.76649
left Nucleus sagulum	-0.00322	-4.10131	0.00000	0.00716
left Nucleus x	-0.00106	-1.59511	0.00000	0.25040
left Nucleus y	-0.00051	-1.21890	0.00000	0.37525
left oculomotor nerve	-0.00020	-1.04795	0.00000	0.46070
left Oculomotor nucleus	-0.00074	-1.35440	0.00000	0.33094
left olfactory nerve layer of main olfactory bulb	-0.10137	-2.76397	0.00000	0.05017
left Olfactory tubercle	-0.06811	-1.32403	0.00000	0.33927
left Olivary pretectal nucleus	-0.00317	-3.76110	0.00000	0.01209
left optic nerve	-0.00210	-1.38936	0.00000	0.31969
left optic radiation	-0.07086	-3.00118	0.00000	0.03573
left optic tract	-0.01509	-1.38596	0.00000	0.32006
left Orbital area, lateral part	-0.06434	-2.74810	0.00000	0.05137
left Orbital area, medial part	-0.06314	-4.89182	0.00000	0.00303
left Orbital area, ventrolateral part	-0.06399	-3.62709	0.00000	0.01482

left Parabigeminal nucleus	-0.00077	-0.93548	0.00000	0.51255
left Parabrachial nucleus	-0.03842	-3.24801	0.00000	0.02539
left Paracentral nucleus	-0.00756	-1.87066	0.00000	0.17434
left Parafascicular nucleus	-0.01156	-1.47326	0.00000	0.29129
left Paraflocculus	-0.15250	-2.53654	0.00000	0.07097
left Paragigantocellular reticular nucleus, dorsal part	0.00243	0.65021	0.00000	0.65493
left Paragigantocellular reticular nucleus, lateral part	0.00057	0.05806	0.00015	0.96691
left Paramedian lobule	-0.27669	-3.53541	0.00000	0.01652
left Paranigral nucleus	-0.00141	-1.99093	0.00000	0.14773
left Parapyramidal nucleus	-0.00132	-0.85480	0.00000	0.55493
left Parasolitary nucleus	0.00021	0.24933	0.00000	0.87045
left Parastrial nucleus	-0.00065	-0.67867	0.00000	0.64102
left Parasubiculum	-0.04198	-3.94014	0.00000	0.00911
left Parasubthalamic nucleus	-0.00315	-1.45855	0.00000	0.29561
left Parataenial nucleus	-0.00561	-1.89423	0.00000	0.16872
left Paratrigeminal nucleus	0.00075	0.37336	0.00000	0.79816
left Paratrochlear nucleus	-0.00038	-0.78747	0.00000	0.58338
left Paraventricular hypothalamic nucleus	-0.02314	-4.45664	0.00000	0.00471
left Paraventricular nucleus of the thalamus	-0.00666	-0.97530	0.00000	0.49671
left Parvicellular motor 5 nucleus	0.00030	0.18605	0.00000	0.89890
left Parvicellular reticular nucleus	-0.02873	-1.22966	0.00000	0.37130
left Pedunculopontine nucleus	-0.03600	-3.90213	0.00000	0.00953
left Perifornical nucleus	-0.00755	-2.91366	0.00000	0.04096
left Peripeduncular nucleus	-0.00241	-4.19801	0.00000	0.00594
left Perireunensis nucleus	-0.00909	-4.78608	0.00000	0.00324
left Perirhinal area	-0.02679	-3.55410	0.00000	0.01652
left Peritrigeminal zone	-0.01040	-2.71252	0.00000	0.05458
left Periventricular hypothalamic nucleus	-0.02004	-2.69974	0.00000	0.05514
left Piriform area	-0.32564	-2.96713	0.00000	0.03756
left Piriform-amygdalar area	-0.01223	-0.76667	0.00000	0.59678
left Pontine central gray	0.00117	0.21018	0.00000	0.88876
left Pontine gray	-0.04674	-3.31126	0.00000	0.02297
left Pontine reticular nucleus	-0.05546	-2.23391	0.00000	0.10665
left Pontine reticular nucleus, caudal part	-0.04103	-1.70298	0.00000	0.21984
left Posterior amygdalar nucleus	-0.03609	-4.41013	0.00000	0.00500
left Posterior auditory area	-0.00950	-1.16073	0.00000	0.40532
left Posterior complex of the thalamus	-0.01143	-0.61947	0.00000	0.67071
left Posterior hypothalamic nucleus	-0.03543	-3.22899	0.00000	0.02584
left Posterior intralaminar thalamic nucleus	-0.00533	-2.16803	0.00000	0.11536
left Posterior limiting nucleus of the thalamus	-0.00635	-3.51522	0.00000	0.01682

left Posterior pretectal nucleus	-0.00719	-5.33161	0.00000	0.00184
left Posterior triangular thalamic nucleus	-0.00697	-2.63446	0.00000	0.06020
left Posterodorsal preoptic nucleus	-0.00006	-0.21653	0.00000	0.88876
left Posterodorsal tegmental nucleus	-0.00033	-0.32502	0.00000	0.82509
left Posterolateral visual area	0.01295	1.33303	0.00000	0.33585
left posteromedial visual area	-0.01763	-1.03026	0.00000	0.46789
left Postpiriform transition area	-0.02443	-1.55585	0.00000	0.26042
left Postrhinal area	0.01742	0.95814	0.00000	0.50219
left Postsubiculum	-0.05051	-4.48869	0.00000	0.00471
left Precommissural nucleus	0.00517	1.58917	0.00000	0.25135
left Prelimbic area	-0.01344	-0.65573	0.00000	0.65256
left Preparasubthalamic nucleus	-0.00018	-0.35823	0.00000	0.80610
left Presubiculum	-0.03609	-3.07256	0.00000	0.03193
left Primary auditory area	-0.03426	-1.52811	0.00000	0.26926
left Primary motor area	0.03392	0.39285	0.00000	0.78938
left Primary somatosensory area, barrel field	-0.12927	-1.86236	0.00000	0.17624
left Primary somatosensory area, lower limb	-0.01088	-0.61664	0.00000	0.67188
left Primary somatosensory area, mouth	-0.05557	-1.10402	0.00000	0.43010
left Primary somatosensory area, nose	-0.04718	-1.56050	0.00000	0.26022
left Primary somatosensory area, trunk	-0.02642	-1.75639	0.00000	0.20568
left Primary somatosensory area, unassigned	-0.01336	-1.03382	0.00000	0.46789
left Primary somatosensory area, upper limb	-0.01584	-0.47666	0.00000	0.73962
left Primary visual area	-0.13701	-1.31505	0.00000	0.34117
left principal mammillary tract	-0.00117	-2.36904	0.00000	0.08953
left Principal sensory nucleus of the trigeminal	-0.01459	-0.94757	0.00000	0.50694
left Prosubiculum	-0.01399	-0.81782	0.00000	0.56825
left pyramid	-0.00066	-0.13635	0.00001	0.92556
left Red nucleus	-0.02511	-2.73630	0.00000	0.05241
left Reticular nucleus of the thalamus	-0.02916	-1.38450	0.00000	0.32006
left Retrochiasmatic area	0.00324	1.34685	0.00000	0.33318
left Retroparafascicular nucleus	-0.00214	-2.29301	0.00000	0.09860
left Retrosplenial area, dorsal part	-0.02090	-0.58483	0.00000	0.68426
left Retrosplenial area, lateral agranular part	-0.02991	-1.24721	0.00000	0.36349
left Retrosplenial area, ventral part	0.03619	0.79675	0.00000	0.57805
left Rostrolateral area	-0.04052	-3.16692	0.00000	0.02841
left rubrospinal tract	-0.01085	-2.76394	0.00000	0.05017
left Secondary motor area	0.09563	0.96065	0.00000	0.50136
left sensory root of the trigeminal nerve	-0.00130	-0.20956	0.00000	0.88876
left Septohippocampal nucleus	-0.00077	-0.89842	0.00000	0.53013
left Simple lobule	-0.38405	-4.48241	0.00000	0.00471

left solitary tract	0.00005	0.16016	0.00000	0.91127
left Spinal nucleus of the trigeminal, caudal part	-0.01056	-0.50971	0.00000	0.72153
left Spinal nucleus of the trigeminal, interpolar part	0.00823	0.37234	0.00000	0.79816
left Spinal nucleus of the trigeminal, oral part	-0.01718	-1.52795	0.00000	0.26926
left spinal tract of the trigeminal nerve	-0.01964	-1.25467	0.00000	0.36001
left Spinal vestibular nucleus	0.00716	0.73236	0.00000	0.61181
left stria medullaris	-0.00839	-2.21630	0.00000	0.10851
left stria terminalis	-0.00198	-0.42548	0.00000	0.77419
left Subceruleus nucleus	-0.00031	-0.57847	0.00000	0.68610
left subependymal zone	0.00003	0.01212	0.00749	0.99304
left Subgeniculate nucleus	-0.00060	-0.96803	0.00000	0.49900
left Subiculum	-0.05684	-1.99709	0.00000	0.14773
left Sublaterodorsal nucleus	0.00073	0.78701	0.00000	0.58338
left Submedial nucleus of the thalamus	-0.01100	-2.08699	0.00000	0.12998
left Subparafascicular area	-0.00418	-1.63613	0.00000	0.23866
left Subparafascicular nucleus	-0.00861	-3.18475	0.00000	0.02760
left Subparaventricular zone	-0.00092	-0.46954	0.00000	0.74397
left Substantia innominata	-0.07261	-1.96584	0.00000	0.15318
left Substantia nigra, compact part	-0.00048	-0.16759	0.00000	0.90773
left Substantia nigra, reticular part	-0.01266	-0.72708	0.00000	0.61382
left Subthalamic nucleus	-0.00360	-1.73554	0.00000	0.21128
left Superior central nucleus raphe	-0.01552	-1.89577	0.00000	0.16872
left superior cerebelar peduncles	-0.02925	-4.04164	0.00000	0.00806
left Superior colliculus	-0.18710	-2.36925	0.00000	0.08953
left Superior olivary complex, lateral part	-0.00689	-1.69171	0.00000	0.22374
left Superior olivary complex, medial part	-0.00198	-1.12217	0.00000	0.42110
left Superior olivary complex, periolivary region	-0.00441	-0.92808	0.00000	0.51593
left Superior vestibular nucleus	-0.00795	-1.58413	0.00000	0.25167
left Supplemental somatosensory area	-0.17502	-2.06250	0.00000	0.13445
left supra-callosal cerebral white matter	-0.02787	-2.61433	0.00000	0.06195
left Suprachiasmatic nucleus	0.00043	0.47630	0.00000	0.73962
left Suprageniculate nucleus	-0.01038	-5.33423	0.00000	0.00184
left Supragenual nucleus	0.00012	0.33466	0.00000	0.81894
left Supramammillary nucleus	-0.00532	-1.99294	0.00000	0.14773
left Supraoculomotor periaqueductal gray	-0.00131	-2.05343	0.00000	0.13522
left supraoptic commissures	-0.00044	-0.76443	0.00000	0.59748
left Supraoptic nucleus	-0.00075	-1.26181	0.00000	0.35821
left Supratrigeminal nucleus	-0.00595	-1.59750	0.00000	0.25024
left Taenia tecta, dorsal part	-0.05625	-4.85898	0.00000	0.00303
left Taenia tecta, ventral part	-0.02158	-2.19572	0.00000	0.11109

left Tegmental reticular nucleus	-0.02946	-3.40251	0.00000	0.02017
left Temporal association areas	-0.04324	-1.25767	0.00000	0.35933
left trapezoid body	-0.00446	-1.42713	0.00000	0.30592
left trochlear nerve	-0.00090	-3.92679	0.00000	0.00916
left Trochlear nucleus	0.00017	0.65780	0.00000	0.65256
left Tuberal nucleus	0.00863	0.97099	0.00000	0.49880
left Tuberomammillary nucleus, dorsal part	-0.00014	-0.21418	0.00000	0.88876
left Tuberomammillary nucleus, ventral part	0.00032	0.13190	0.00001	0.92790
left uncinate fascicle	-0.00340	-2.40443	0.00000	0.08462
left Ventral anterior-lateral complex of the thalamus	-0.01262	-1.02973	0.00000	0.46789
left Ventral auditory area	-0.03686	-2.30957	0.00000	0.09607
left Ventral cochlear nucleus	-0.01845	-1.58261	0.00000	0.25170
left Ventral medial nucleus of the thalamus	-0.00611	-0.33396	0.00000	0.81894
left Ventral part of the lateral geniculate complex	-0.01107	-1.85998	0.00000	0.17624
left Ventral posterolateral nucleus of the thalamus	-0.00115	-0.07251	0.00006	0.96176
left Ventral posteromedial nucleus of the thalamus	0.02635	0.80400	0.00000	0.57560
left Ventral premammillary nucleus	-0.00352	-1.27318	0.00000	0.35331
left ventral spinocerebellar tract	-0.00313	-1.30866	0.00000	0.34178
left Ventral tegmental area	-0.01578	-2.84321	0.00000	0.04558
left Ventral tegmental nucleus	-0.00116	-2.14789	0.00000	0.11964
left Ventrolateral preoptic nucleus	0.00021	0.17300	0.00000	0.90458
left Ventromedial hypothalamic nucleus	-0.00001	-0.00143	0.03994	0.99999
left Ventromedial preoptic nucleus	-0.00009	-0.06891	0.00007	0.96250
left vestibular nerve	-0.00060	-0.14204	0.00001	0.92345
left Vestibulocerebellar nucleus	-0.00406	-2.49058	0.00000	0.07629
left Visceral area	-0.03529	-1.60234	0.00000	0.24876
left vomeronasal nerve	-0.00008	-0.20776	0.00000	0.88876
left Xiphoid thalamic nucleus	-0.00460	-1.99288	0.00000	0.14773
left Zona incerta	-0.03102	-1.98496	0.00000	0.14859
Lobule II	-0.02375	-0.35551	0.00000	0.80716
Lobule III	-0.40193	-3.11646	0.00000	0.03055
Lobules IV-V	-1.06788	-4.55289	0.00000	0.00451
Medial septal nucleus	-0.00229	-0.26558	0.00000	0.85832
Median eminence	-0.00201	-1.33800	0.00000	0.33426
Median preoptic nucleus	-0.00270	-1.64661	0.00000	0.23555
Nodulus (X)	-0.10453	-1.38234	0.00000	0.32047
Nucleus raphe magnus	-0.00552	-0.70336	0.00000	0.62595
Nucleus raphe obscurus	-0.00749	-2.59706	0.00000	0.06395
Nucleus raphe pallidus	-0.00299	-1.43939	0.00000	0.30113
Nucleus raphe pontis	0.00108	0.59798	0.00000	0.67597

optic chiasm	-0.01023	-1.13808	0.00000	0.41527
Periaqueductal gray	-0.25829	-3.24548	0.00000	0.02539
posterior commissure	-0.00099	-0.67053	0.00000	0.64636
pyramidal decussation	-0.00145	-0.53479	0.00000	0.70689
Pyramus (VIII)	-0.25319	-4.26321	0.00000	0.00571
Rhomboid nucleus	-0.00479	-2.28668	0.00000	0.09913
right Abducens nucleus	-0.00025	-0.72134	0.00000	0.61618
right Accessory facial motor nucleus	0.00019	0.83247	0.00000	0.56428
right Accessory olfactory bulb	-0.00585	-0.55570	0.00000	0.69669
right Accessory supraoptic group	-0.00009	-0.55165	0.00000	0.69776
right Accessory trigeminal nucleus	0.00020	0.37220	0.00000	0.79816
right Agranular insular area, dorsal part	-0.12401	-3.36037	0.00000	0.02161
right Agranular insular area, posterior part	-0.00441	-0.23482	0.00000	0.87993
right Agranular insular area, ventral part	-0.08377	-4.21290	0.00000	0.00591
right alveus	-0.03188	-3.73852	0.00000	0.01240
right amygdalar capsule	0.00023	0.10684	0.00002	0.94059
right Anterior amygdalar area	-0.01429	-2.32726	0.00000	0.09418
right Anterior area	-0.02146	-1.09478	0.00000	0.43518
right Anterior cingulate area	0.02128	0.60215	0.00000	0.67459
right Anterior cingulate area, ventral part	0.01329	0.55293	0.00000	0.69776
right Anterior hypothalamic nucleus	0.00015	0.02313	0.00388	0.98565
right Anterior olfactory nucleus	-0.18309	-5.08183	0.00000	0.00249
right Anterior pretectal nucleus	-0.05504	-4.78565	0.00000	0.00324
right Anterior tegmental nucleus	-0.00105	-2.08702	0.00000	0.12998
right Anterodorsal nucleus	-0.00180	-0.75374	0.00000	0.60258
right Anterodorsal preoptic nucleus	-0.00254	-2.47999	0.00000	0.07708
right Anterolateral visual area	-0.01546	-1.44963	0.00000	0.29740
right Anteromedial nucleus, dorsal part	-0.00871	-2.69797	0.00000	0.05514
right Anteromedial nucleus, ventral part	-0.00358	-1.40913	0.00000	0.31157
right Anteromedial visual area	-0.01432	-1.07390	0.00000	0.44529
right Anteroventral nucleus of thalamus	-0.00538	-0.83509	0.00000	0.56428
right Anteroventral periventricular nucleus	-0.00155	-0.64192	0.00000	0.65934
right Anteroventral preoptic nucleus	0.00153	1.28400	0.00000	0.35071
right Arcuate hypothalamic nucleus	0.00110	0.27052	0.00000	0.85752
right Area postrema	-0.00184	-0.91283	0.00000	0.52590
right Area prostriata	-0.01838	-3.54167	0.00000	0.01652
right auditory radiation	-0.00682	-1.33731	0.00000	0.33426
right Barrington's nucleus	-0.00087	-2.46703	0.00000	0.07800
right Basolateral amygdalar nucleus, anterior part	-0.02731	-3.06541	0.00000	0.03214
right Basolateral amygdalar nucleus, posterior part	-0.02417	-3.09884	0.00000	0.03074

right Basolateral amygdalar nucleus, ventral part	-0.00977	-2.61832	0.00000	0.06171
right Basomedial amygdalar nucleus, anterior part	-0.02236	-2.90772	0.00000	0.04102
right Basomedial amygdalar nucleus, posterior part	-0.03062	-5.63242	0.00000	0.00169
right Bed nuclei of the stria terminalis	-0.04294	-3.33240	0.00000	0.02227
right Bed nucleus of the accessory olfactory tract	-0.00093	-2.07440	0.00000	0.13234
right Bed nucleus of the anterior commissure	-0.00146	-3.47164	0.00000	0.01805
right brachium of the inferior colliculus	-0.00951	-4.01118	0.00000	0.00840
right brachium of the superior colliculus	-0.01281	-4.80388	0.00000	0.00324
right Caudoputamen	-0.61192	-3.12693	0.00000	0.03048
right Central amygdalar nucleus	-0.06193	-4.77470	0.00000	0.00324
right Central lateral nucleus of the thalamus	-0.00863	-1.71473	0.00000	0.21582
right Central medial nucleus of the thalamus	-0.00545	-1.92803	0.00000	0.16114
right cerebal peduncle	-0.02164	-1.72169	0.00000	0.21429
right choroid plexus	-0.00682	-0.38016	0.00000	0.79631
right cingulum bundle	-0.02669	-2.28076	0.00000	0.09972
right Claustrum	-0.01630	-3.25760	0.00000	0.02509
right columns of the fornix	-0.01128	-4.62189	0.00000	0.00407
right commissural branch of stria terminalis	-0.00057	-1.00273	0.00000	0.48232
right Copula pyramidis	-0.17200	-3.97798	0.00000	0.00870
right Cortical amygdalar area, anterior part	-0.02407	-2.35657	0.00000	0.09147
right Cortical amygdalar area, posterior part, lateral zone	-0.03794	-3.07702	0.00000	0.03183
right Cortical amygdalar area, posterior part, medial zone	-0.05196	-4.51766	0.00000	0.00466
right Cortical subplate	-0.00622	-2.32465	0.00000	0.09425
right corticospinal tract	-0.00599	-6.11969	0.00000	0.00136
right crossed tectospinal pathway	-0.01187	-1.68130	0.00000	0.22734
right Crus 1	-0.25054	-2.43778	0.00000	0.08030
right Crus 2	-0.15789	-1.59157	0.00000	0.25093
right cuneate fascicle	0.00098	1.71711	0.00000	0.21548
right Cuneate nucleus	0.00583	0.89783	0.00000	0.53013
right Cuneiform nucleus	-0.01808	-2.51726	0.00000	0.07323
right Dentate gyrus	-0.29659	-5.18733	0.00000	0.00215
right Dentate nucleus	-0.02165	-5.97748	0.00000	0.00151
right Diagonal band nucleus	-0.00464	-0.58708	0.00000	0.68356
right dorsal acoustic stria	-0.00033	-0.96389	0.00000	0.50096
right Dorsal auditory area	-0.02712	-1.93026	0.00000	0.16095
right Dorsal cochlear nucleus	-0.00604	-0.52402	0.00000	0.71295
right dorsal limb	-0.00750	-1.79486	0.00000	0.19396
right Dorsal motor nucleus of the vagus nerve	-0.00257	-0.85347	0.00000	0.55493
right Dorsal nucleus raphe	0.00071	0.27420	0.00000	0.85607
right Dorsal part of the lateral geniculate complex	-0.04044	-5.29694	0.00000	0.00189

right Dorsal peduncular area	-0.03313	-4.72810	0.00000	0.00331
right Dorsal premammillary nucleus	-0.00250	-1.36637	0.00000	0.32674
right dorsal spinocerebellar tract	-0.00022	-0.21864	0.00000	0.88876
right Dorsal tegmental nucleus	0.00074	0.72388	0.00000	0.61525
right Dorsal terminal nucleus of the accessory optic tract	-0.00111	-3.94966	0.00000	0.00904
right Dorsomedial nucleus of the hypothalamus	-0.01006	-2.38076	0.00000	0.08811
right Ectorhinal area	-0.04476	-2.33890	0.00000	0.09335
right Endopiriform nucleus, dorsal part	-0.05915	-3.33786	0.00000	0.02225
right Endopiriform nucleus, ventral part	-0.02679	-3.20596	0.00000	0.02671
right Entorhinal area, lateral part	-0.27491	-4.30980	0.00000	0.00571
right Entorhinal area, medial part, dorsal zone	-0.18937	-3.04914	0.00000	0.03298
right Ethmoid nucleus of the thalamus	-0.00910	-3.43652	0.00000	0.01935
right external capsule	-0.01845	-3.10102	0.00000	0.03074
right External cuneate nucleus	-0.00246	-0.57848	0.00000	0.68610
right external medullary lamina of the thalamus	-0.00433	-2.47449	0.00000	0.07763
right Facial motor nucleus	-0.00659	-0.55628	0.00000	0.69669
right facial nerve	-0.00161	-1.23691	0.00000	0.36864
right fasciculus retroflexus	-0.00406	-2.11486	0.00000	0.12513
right Fasciola cinerea	-0.00268	-1.65942	0.00000	0.23264
right Fastigial nucleus	-0.04685	-6.42795	0.00000	0.00133
right Field CA1	-0.30035	-2.82427	0.00000	0.04671
right Field CA2	-0.01990	-3.83388	0.00000	0.01063
right Field CA3	-0.28152	-4.26711	0.00000	0.00571
right Fields of Forel	-0.00822	-2.77327	0.00000	0.04994
right fimbria	-0.01018	-0.49833	0.00000	0.72628
right Flocculus	-0.05502	-2.18813	0.00000	0.11227
right Frontal pole	0.00930	0.90584	0.00000	0.52902
right Fundus of striatum	0.00027	0.03479	0.00084	0.98028
right Gigantocellular reticular nucleus	-0.04131	-1.39467	0.00000	0.31763
right Globus pallidus, external segment	-0.04437	-3.53685	0.00000	0.01652
right Globus pallidus, internal segment	-0.01695	-3.01184	0.00000	0.03512
right Gracile nucleus	0.00021	0.14752	0.00001	0.92026
right Gustatory areas	-0.03675	-2.80426	0.00000	0.04772
right Hippocampal formation	-0.01327	-2.91682	0.00000	0.04092
right Hippocampo-amygdalar transition area	-0.01206	-2.90953	0.00000	0.04102
right inferior cerebellar peduncle	-0.02203	-2.33001	0.00000	0.09418
right Inferior colliculus	-0.16767	-2.83500	0.00000	0.04589
right Inferior olivary complex	-0.01072	-1.32057	0.00000	0.34042
right Inferior salivatory nucleus	-0.00044	-1.32934	0.00000	0.33711
right Infracerebellar nucleus	-0.00605	-4.20333	0.00000	0.00594

right Infralimbic area	-0.02691	-2.85097	0.00000	0.04508
right Interanterodorsal nucleus of the thalamus	-0.00422	-2.69419	0.00000	0.05514
right Intercalated amygdalar nucleus	-0.00415	-1.79453	0.00000	0.19396
right Intergeniculate leaflet of the lateral geniculate complex	-0.00374	-3.43035	0.00000	0.01937
right Intermediate geniculate nucleus	-0.00041	-1.21894	0.00000	0.37525
right Intermediate reticular nucleus	-0.04120	-1.28130	0.00000	0.35071
right internal capsule	-0.07863	-2.77890	0.00000	0.04960
right Interposed nucleus	-0.06742	-5.66861	0.00000	0.00169
right Interstitial nucleus of Cajal	-0.00489	-4.21363	0.00000	0.00591
right Intertrigeminal nucleus	-0.00019	-0.17578	0.00000	0.90458
right Koelliker-Fuse subnucleus	-0.00820	-3.22760	0.00000	0.02584
right Lateral amygdalar nucleus	-0.00713	-0.41121	0.00000	0.78012
right Lateral dorsal nucleus of thalamus	-0.03179	-2.45007	0.00000	0.07998
right Lateral habenula	-0.00348	-0.66094	0.00000	0.65256
right Lateral hypothalamic area	-0.07339	-3.49395	0.00000	0.01748
right lateral lemniscus	-0.02112	-2.44489	0.00000	0.07998
right Lateral mammillary nucleus	0.00113	0.42051	0.00000	0.77711
right lateral olfactory tract, body	-0.02031	-1.74166	0.00000	0.20952
right Lateral posterior nucleus of the thalamus	-0.06754	-5.00696	0.00000	0.00281
right Lateral preoptic area	-0.00905	-1.63717	0.00000	0.23866
right lateral recess	-0.00508	-0.94741	0.00000	0.50694
right Lateral reticular nucleus, magnocellular part	-0.00906	-1.31140	0.00000	0.34161
right Lateral reticular nucleus, parvicellular part	-0.00091	-1.66557	0.00000	0.23195
right Lateral septal nucleus, caudal (caudodorsal) part	-0.01045	-1.57963	0.00000	0.25238
right Lateral septal nucleus, rostral (rostroventral) part	-0.02005	-0.84424	0.00000	0.55950
right Lateral septal nucleus, ventral part	-0.01413	-1.95532	0.00000	0.15486
right Lateral terminal nucleus of the accessory optic tract	-0.00066	-1.34665	0.00000	0.33318
right lateral ventricle	-0.00839	-0.21891	0.00000	0.88876
right Lateral vestibular nucleus	-0.00390	-1.12001	0.00000	0.42163
right Lateral visual area	-0.00284	-0.12948	0.00001	0.92797
right Laterodorsal tegmental nucleus	-0.00054	-0.23934	0.00000	0.87744
right Laterointermediate area	-0.00045	-0.05148	0.00028	0.97087
right Linear nucleus of the medulla	-0.00008	-0.10680	0.00002	0.94059
right Lingula (I)	0.00170	0.56057	0.00000	0.69497
right Locus ceruleus	0.00012	0.31539	0.00000	0.83062
right Magnocellular nucleus	-0.01378	-2.25973	0.00000	0.10237
right Magnocellular reticular nucleus	-0.01453	-1.84033	0.00000	0.18075
right Main olfactory bulb	-1.20993	-5.16981	0.00000	0.00215
right mammillary peduncle	-0.00111	-1.16436	0.00000	0.40392
right mammillotegmental tract	-0.00497	-2.28003	0.00000	0.09972

right mammillothalamic tract	-0.00346	-1.82356	0.00000	0.18594
right Medial accesory oculomotor nucleus	-0.00256	-2.48910	0.00000	0.07629
right Medial amygdalar nucleus	-0.09316	-5.47603	0.00000	0.00184
right medial corticohypothalamic tract	-0.00043	-1.22811	0.00000	0.37143
right medial forebrain bundle	-0.00130	-1.93920	0.00000	0.15872
right Medial geniculate complex, dorsal part	-0.00683	-4.11368	0.00000	0.00706
right Medial geniculate complex, medial part	-0.00611	-2.48616	0.00000	0.07643
right Medial geniculate complex, ventral part	-0.00777	-2.10822	0.00000	0.12635
right Medial habenula	-0.00609	-0.91109	0.00000	0.52619
right medial lemniscus	-0.03074	-4.45818	0.00000	0.00471
right medial longitudinal fascicle	-0.00191	-0.75430	0.00000	0.60258
right Medial mammillary nucleus	-0.00593	-0.63709	0.00000	0.66209
right Medial preoptic area	-0.00475	-0.83674	0.00000	0.56408
right Medial preoptic nucleus	-0.00699	-1.65400	0.00000	0.23366
right Medial pretectal area	0.00003	0.03918	0.00039	0.97937
right Medial terminal nucleus of the accessory optic tract	-0.00131	-1.28049	0.00000	0.35071
right Medial vestibular nucleus	-0.00343	-0.19579	0.00000	0.89590
right Mediodorsal nucleus of thalamus	-0.01828	-1.37259	0.00000	0.32416
right Medullary reticular nucleus, dorsal part	-0.01249	-0.78523	0.00000	0.58371
right Medullary reticular nucleus, ventral part	0.00134	0.07539	0.00005	0.96073
right Midbrain reticular nucleus	-0.18304	-3.89816	0.00000	0.00953
right Midbrain reticular nucleus, retrorubral area	-0.00311	-1.54964	0.00000	0.26194
right Midbrain trigeminal nucleus	-0.00118	-3.16517	0.00000	0.02841
right middle cerebellar peduncle	-0.03423	-2.69643	0.00000	0.05514
right Motor nucleus of trigeminal	-0.00466	-0.99567	0.00000	0.48534
right motor root of the trigeminal nerve	0.00099	1.15398	0.00000	0.40787
right nigrostriatal tract	-0.00344	-2.68336	0.00000	0.05585
right Nucleus accumbens	-0.16317	-3.21002	0.00000	0.02666
right Nucleus ambiguus, dorsal division	0.00059	0.81037	0.00000	0.57183
right Nucleus ambiguus, ventral division	-0.00012	-0.18301	0.00000	0.90015
right Nucleus incertus	0.00057	0.39992	0.00000	0.78590
right Nucleus of Darkschewitsch	-0.00508	-2.63698	0.00000	0.06020
right Nucleus of reuniens	-0.03211	-5.81581	0.00000	0.00158
right Nucleus of Roller	-0.00089	-1.38545	0.00000	0.32006
right Nucleus of the brachium of the inferior colliculus	-0.00342	-3.73483	0.00000	0.01240
right Nucleus of the lateral lemniscus	-0.01630	-1.94256	0.00000	0.15818
right Nucleus of the lateral olfactory tract	-0.00963	-2.49918	0.00000	0.07570
right Nucleus of the optic tract	-0.01127	-4.87751	0.00000	0.00303
right Nucleus of the posterior commissure	-0.00328	-1.20160	0.00000	0.38386
right Nucleus of the solitary tract	-0.00791	-0.71290	0.00000	0.62063

right Nucleus of the trapezoid body	-0.00352	-2.22782	0.00000	0.10718
right Nucleus prepositus	0.00076	0.26574	0.00000	0.85832
right Nucleus sagulum	-0.00270	-2.47022	0.00000	0.07798
right Nucleus x	-0.00069	-0.54443	0.00000	0.70069
right Nucleus y	-0.00093	-2.26806	0.00000	0.10141
right oculomotor nerve	-0.00008	-0.18832	0.00000	0.89829
right Oculomotor nucleus	-0.00144	-1.72376	0.00000	0.21407
right olfactory nerve layer of main olfactory bulb	-0.09901	-2.44975	0.00000	0.07998
right Olfactory tubercle	-0.10479	-2.09472	0.00000	0.12930
right Olivary pretectal nucleus	-0.00159	-2.06554	0.00000	0.13421
right optic nerve	-0.00057	-0.37875	0.00000	0.79631
right optic radiation	-0.06211	-2.81250	0.00000	0.04714
right optic tract	-0.01527	-1.45603	0.00000	0.29561
right Orbital area, lateral part	-0.05678	-2.18499	0.00000	0.11231
right Orbital area, medial part	-0.05566	-3.99643	0.00000	0.00857
right Orbital area, ventrolateral part	-0.04336	-2.22321	0.00000	0.10779
right Parabigeminal nucleus	-0.00280	-3.52336	0.00000	0.01682
right Parabrachial nucleus	-0.03760	-3.25924	0.00000	0.02509
right Paracentral nucleus	-0.00584	-1.23148	0.00000	0.37101
right Parafascicular nucleus	-0.00580	-0.65566	0.00000	0.65256
right Paraflocculus	-0.15942	-2.69737	0.00000	0.05514
right Paragigantocellular reticular nucleus, dorsal part	0.00243	0.71895	0.00000	0.61700
right Paragigantocellular reticular nucleus, lateral part	-0.00714	-0.63239	0.00000	0.66333
right Paramedian lobule	-0.32642	-3.48665	0.00000	0.01761
right Paranigral nucleus	-0.00099	-1.30123	0.00000	0.34513
right Parapyramidal nucleus	-0.00103	-0.60364	0.00000	0.67459
right Parasolitary nucleus	0.00043	0.58098	0.00000	0.68610
right Parastrial nucleus	-0.00110	-1.31551	0.00000	0.34117
right Parasubiculum	-0.05967	-4.97177	0.00000	0.00281
right Parasubthalamic nucleus	-0.00479	-2.81922	0.00000	0.04671
right Parataenial nucleus	-0.01037	-3.54913	0.00000	0.01652
right Paratrigeminal nucleus	-0.00322	-1.55915	0.00000	0.26022
right Paratrochlear nucleus	-0.00075	-2.34672	0.00000	0.09253
right Paraventricular hypothalamic nucleus	-0.02639	-4.60405	0.00000	0.00413
right Paraventricular nucleus of the thalamus	-0.00276	-0.37871	0.00000	0.79631
right Parvicellular motor 5 nucleus	0.00068	0.56155	0.00000	0.69497
right Parvicellular reticular nucleus	-0.04394	-1.61316	0.00000	0.24533
right Pedunculopontine nucleus	-0.03302	-3.58125	0.00000	0.01594
right Perifornical nucleus	-0.01003	-3.63773	0.00000	0.01462
right Peripeduncular nucleus	-0.00254	-2.96884	0.00000	0.03756

right Perireunensis nucleus	-0.00713	-4.37498	0.00000	0.00530
right Perirhinal area	-0.03331	-3.83683	0.00000	0.01063
right Peritrigeminal zone	-0.00634	-1.33923	0.00000	0.33426
right Periventricular hypothalamic nucleus	-0.01447	-1.46912	0.00000	0.29193
right Piriform area	-0.32990	-2.49312	0.00000	0.07629
right Piriform-amygdalar area	-0.02952	-1.80622	0.00000	0.19091
right Pontine central gray	0.00331	0.48919	0.00000	0.73254
right Pontine gray	-0.04914	-3.20074	0.00000	0.02683
right Pontine reticular nucleus	-0.05473	-2.23392	0.00000	0.10665
right Pontine reticular nucleus, caudal part	-0.03981	-1.91050	0.00000	0.16509
right Posterior amygdalar nucleus	-0.04414	-4.86466	0.00000	0.00303
right Posterior auditory area	-0.01014	-1.34403	0.00000	0.33358
right Posterior complex of the thalamus	-0.01447	-0.81946	0.00000	0.56825
right Posterior hypothalamic nucleus	-0.02916	-2.69387	0.00000	0.05514
right Posterior intralaminar thalamic nucleus	-0.00381	-1.54517	0.00000	0.26267
right Posterior limiting nucleus of the thalamus	-0.00926	-3.94906	0.00000	0.00904
right Posterior pretectal nucleus	-0.00644	-2.95955	0.00000	0.03774
right Posterior triangular thalamic nucleus	-0.00943	-3.14237	0.00000	0.02966
right Posterodorsal preoptic nucleus	0.00003	0.16356	0.00000	0.90976
right Posterodorsal tegmental nucleus	0.00072	0.81794	0.00000	0.56825
right Posterolateral visual area	-0.00171	-0.11441	0.00002	0.93698
right posteromedial visual area	-0.01287	-0.72732	0.00000	0.61382
right Postpiriform transition area	-0.02846	-2.13228	0.00000	0.12255
right Postrhinal area	-0.00121	-0.06045	0.00020	0.96691
right Postsubiculum	-0.05611	-4.98917	0.00000	0.00281
right Precommissural nucleus	0.00474	1.44496	0.00000	0.29903
right Prelimbic area	0.00067	0.02956	0.00132	0.98313
right Preparasubthalamic nucleus	-0.00027	-0.73278	0.00000	0.61181
right Presubiculum	-0.04720	-5.40206	0.00000	0.00184
right Primary auditory area	-0.03798	-1.63177	0.00000	0.23989
right Primary motor area	-0.02919	-0.40770	0.00000	0.78141
right Primary somatosensory area, barrel field	-0.08305	-1.02405	0.00000	0.46960
right Primary somatosensory area, lower limb	-0.01208	-0.61417	0.00000	0.67276
right Primary somatosensory area, mouth	-0.08491	-2.32161	0.00000	0.09443
right Primary somatosensory area, nose	-0.05444	-2.03784	0.00000	0.13891
right Primary somatosensory area, trunk	-0.00993	-0.56232	0.00000	0.69497
right Primary somatosensory area, unassigned	-0.01368	-1.15943	0.00000	0.40532
right Primary somatosensory area, upper limb	-0.02014	-0.61116	0.00000	0.67408
right Primary visual area	-0.08950	-0.79798	0.00000	0.57805
right principal mammillary tract	-0.00017	-0.20755	0.00000	0.88876

right Principal sensory nucleus of the trigeminal	-0.02000	-1.34613	0.00000	0.33318
right Prosubiculum	-0.02290	-1.46667	0.00000	0.29244
right pyramid	-0.00115	-0.21489	0.00000	0.88876
right Red nucleus	-0.02305	-2.44173	0.00000	0.07998
right Reticular nucleus of the thalamus	-0.03714	-1.45408	0.00000	0.29588
right Retrochiasmatic area	0.00141	0.53189	0.00000	0.70813
right Retroparafascicular nucleus	-0.00155	-1.66124	0.00000	0.23250
right Retrosplenial area, dorsal part	0.00462	0.10405	0.00003	0.94154
right Retrosplenial area, lateral agranular part	-0.01845	-0.60107	0.00000	0.67459
right Retrosplenial area, ventral part	0.06354	1.27722	0.00000	0.35179
right Rostrolateral area	-0.02228	-1.35797	0.00000	0.33050
right rubrospinal tract	-0.00948	-2.12566	0.00000	0.12332
right Secondary motor area	0.05431	0.54921	0.00000	0.69863
right sensory root of the trigeminal nerve	-0.00182	-0.26949	0.00000	0.85752
right Septohippocampal nucleus	-0.00056	-0.63147	0.00000	0.66333
right Simple lobule	-0.44093	-5.47004	0.00000	0.00184
right solitary tract	0.00089	2.26160	0.00000	0.10236
right Spinal nucleus of the trigeminal, caudal part	-0.02208	-1.08614	0.00000	0.43820
right Spinal nucleus of the trigeminal, interpolar part	-0.03801	-1.37497	0.00000	0.32363
right Spinal nucleus of the trigeminal, oral part	-0.02217	-1.89235	0.00000	0.16882
right spinal tract of the trigeminal nerve	-0.01587	-0.81921	0.00000	0.56825
right Spinal vestibular nucleus	0.00331	0.41335	0.00000	0.77967
right stria medullaris	-0.00683	-2.07793	0.00000	0.13186
right stria terminalis	-0.00570	-1.02792	0.00000	0.46789
right Subceruleus nucleus	0.00051	0.85307	0.00000	0.55493
right subependymal zone	-0.00099	-0.43114	0.00000	0.77186
right Subgeniculate nucleus	-0.00053	-1.03103	0.00000	0.46789
right Subiculum	-0.05599	-1.99062	0.00000	0.14773
right Sublaterodorsal nucleus	-0.00055	-0.82327	0.00000	0.56825
right Submedial nucleus of the thalamus	-0.00908	-1.85642	0.00000	0.17642
right Subparafascicular area	-0.00352	-1.28498	0.00000	0.35071
right Subparafascicular nucleus	-0.00744	-2.64614	0.00000	0.05957
right Subparaventricular zone	-0.00499	-2.27051	0.00000	0.10128
right Substantia innominata	-0.05836	-1.65660	0.00000	0.23319
right Substantia nigra, compact part	-0.00334	-1.25718	0.00000	0.35933
right Substantia nigra, reticular part	-0.01112	-0.64568	0.00000	0.65744
right Subthalamic nucleus	-0.00254	-1.28860	0.00000	0.34990
right Superior central nucleus raphe	-0.01827	-2.89383	0.00000	0.04203
right superior cerebelar peduncles	-0.02617	-3.59157	0.00000	0.01588
right Superior colliculus	-0.22216	-2.78487	0.00000	0.04923

right Superior olivary complex, lateral part	-0.00356	-1.29164	0.00000	0.34896
right Superior olivary complex, medial part	-0.00207	-2.21671	0.00000	0.10851
right Superior olivary complex, periolivary region	-0.00481	-1.41498	0.00000	0.30927
right Superior vestibular nucleus	-0.00735	-1.41934	0.00000	0.30918
right Supplemental somatosensory area	-0.19303	-2.31995	0.00000	0.09443
right supra-callosal cerebral white matter	-0.02616	-2.63632	0.00000	0.06020
right Suprachiasmatic nucleus	-0.00141	-1.46954	0.00000	0.29193
right Suprageniculate nucleus	-0.00936	-5.63620	0.00000	0.00169
right Supragenual nucleus	0.00029	0.81958	0.00000	0.56825
right Supramammillary nucleus	-0.00513	-1.58412	0.00000	0.25167
right Supraoculomotor periaqueductal gray	-0.00062	-0.93724	0.00000	0.51226
right supraoptic commissures	0.00013	0.31323	0.00000	0.83121
right Supraoptic nucleus	-0.00078	-1.13135	0.00000	0.41700
right Supratrigeminal nucleus	-0.00610	-1.73206	0.00000	0.21203
right Taenia tecta, dorsal part	-0.05351	-4.43287	0.00000	0.00484
right Taenia tecta, ventral part	-0.01559	-1.59422	0.00000	0.25040
right Tegmental reticular nucleus	-0.03168	-3.30186	0.00000	0.02310
right Temporal association areas	-0.04983	-1.60899	0.00000	0.24651
right trapezoid body	-0.00272	-0.96952	0.00000	0.49888
right trochlear nerve	-0.00005	-0.29625	0.00000	0.84276
right Trochlear nucleus	-0.00020	-0.45477	0.00000	0.75483
right Tuberal nucleus	0.01008	1.13941	0.00000	0.41527
right Tuberomammillary nucleus, dorsal part	-0.00208	-2.20438	0.00000	0.11034
right Tuberomammillary nucleus, ventral part	0.00164	0.63244	0.00000	0.66333
right uncinate fascicle	-0.00363	-2.01289	0.00000	0.14393
right Ventral anterior-lateral complex of the thalamus	-0.00937	-0.90198	0.00000	0.52988
right Ventral auditory area	-0.04317	-2.34595	0.00000	0.09253
right Ventral cochlear nucleus	-0.02058	-1.72913	0.00000	0.21258
right Ventral medial nucleus of the thalamus	0.00523	0.28895	0.00000	0.84756
right Ventral part of the lateral geniculate complex	-0.01158	-2.08865	0.00000	0.12998
right Ventral posterolateral nucleus of the thalamus	-0.00611	-0.34762	0.00000	0.81128
right Ventral posteromedial nucleus of the thalamus	0.01393	0.42567	0.00000	0.77419
right Ventral premammillary nucleus	-0.00132	-0.41316	0.00000	0.77967
right ventral spinocerebellar tract	-0.00441	-1.78554	0.00000	0.19664
right Ventral tegmental area	-0.01690	-2.92223	0.00000	0.04067
right Ventral tegmental nucleus	-0.00015	-0.30055	0.00000	0.84043
right Ventrolateral preoptic nucleus	-0.00076	-0.65456	0.00000	0.65256
right Ventromedial hypothalamic nucleus	0.00430	0.60677	0.00000	0.67459
right Ventromedial preoptic nucleus	0.00042	0.49960	0.00000	0.72628
right vestibular nerve	-0.00304	-0.75181	0.00000	0.60303

right Vestibulocerebellar nucleus	-0.00557	-3.30779	0.00000	0.02297
right Visceral area	-0.04140	-1.85188	0.00000	0.17742
right vomeronasal nerve	-0.00163	-3.10785	0.00000	0.03056
right Xiphoid thalamic nucleus	-0.00312	-1.40508	0.00000	0.31294
right Zona incerta	-0.02825	-1.61565	0.00000	0.24533
root	-0.09740	-1.50714	0.00000	0.27767
Rostral linear nucleus raphe	-0.00641	-3.06390	0.00000	0.03214
Septofimbrial nucleus	-0.01650	-1.74233	0.00000	0.20952
Subcommissural organ	0.00002	0.02339	0.00219	0.98565
Subfornical organ	-0.00141	-1.29409	0.00000	0.34834
superior cerebellar peduncle decussation	-0.00424	-2.66437	0.00000	0.05759
superior colliculus commissure	-0.00177	-2.44262	0.00000	0.07998
third ventricle	-0.05562	-2.86879	0.00000	0.04386
Triangular nucleus of septum	-0.01423	-1.61436	0.00000	0.24533
Uvula (IX)	-0.35531	-2.98705	0.00000	0.03662
Vascular organ of the lamina terminalis	-0.00009	-0.17363	0.00000	0.90458
ventral hippocampal commissure	-0.00195	-0.99890	0.00000	0.48404
ventral tegmental decussation	-0.00321	-2.46599	0.00000	0.07800

	**M83+/+:ΔHET versus M83+/+:ΔSTI1**			
**region**	**beta-M83+/+:ΔHET**	**tvalue-M83+/+:ΔHET**	**pvalue-tvalue-M83+/+:ΔHET**	**qvalue-tvalue-M83+/+:ΔHET**
anterior commissure	0.083254897	2.680272029	2.21E-08	0.053494483
arbor vitae	0.471294329	2.414228748	3.78E-08	0.068891441
central canal, spinal cord/medulla	NA	NA	NA	NA
Central linear nucleus raphe	0.008292098	3.057055699	1.36E-08	0.037004667
cerebellar commissure	0.004896383	1.640753956	2.25E-07	0.178152778
cerebral aqueduct	0.058181693	2.098915413	7.62E-08	0.099239787
corpus callosum	0.943965715	5.754034721	4.98E-09	0.00893279
Declive (VI)	0.057984467	0.496327174	3.19E-05	0.6631472
doral tegmental decussation	0.000926105	2.58939781	2.61E-08	0.058189844
dorsal fornix	0.002040882	1.541465559	2.84E-07	0.203183437
dorsal hippocampal commissure	0.166175858	4.175379689	6.37E-09	0.016557529
Edinger-Westphal nucleus	0.006285493	3.847847442	7.37E-09	0.019935335
fiber tracts	0.13230396	4.300206191	6.24E-09	0.015563882

Folium-tuber vermis (VII)	-0.031393295	-0.540756866	2.77E-05	0.633168275
fourth ventricle	0.029343889	2.119287215	6.99E-08	0.097332787
habenular commissure	0.003790147	2.457434301	3.37E-08	0.066406347
Hypoglossal nucleus	0.036195283	4.575398816	5.81E-09	0.014437957
Induseum griseum	0.006431335	1.35534483	5.19E-07	0.255594522
inferior colliculus commissure	0.000274399	0.227422436	1.25E-03	0.835112546
Interanteromedial nucleus of the thalamus	0.004699114	2.768464643	1.95E-08	0.048696787
Interfascicular nucleus raphe	0.00374732	1.271319161	6.80E-07	0.283616058
Intermediodorsal nucleus of the thalamus	0.00138922	0.297614363	4.43E-04	0.786871673
Interpeduncular nucleus	0.026994186	2.420865304	3.69E-08	0.068622566
left Abducens nucleus	-0.000325848	-0.711729569	8.41E-06	0.535081763
left Accessory facial motor nucleus	0.000428751	1.738363254	1.72E-07	0.157463483
left Accessory olfactory bulb	0.001389101	0.13841441	9.27E-03	0.897074418
left Accessory supraoptic group	2.57E-05	0.183161639	2.13E-03	0.865888209
left Accessory trigeminal nucleus	0.000454481	1.033122933	2.14E-06	0.371367478
left Agranular insular area, dorsal part	0.089635212	2.017606688	9.04E-08	0.109292202
left Agranular insular area, posterior part	0.088288691	5.594550609	5.02E-09	0.009572946
left Agranular insular area, ventral part	0.058301686	2.591376811	2.59E-08	0.058159568
left alveus	0.06879745	6.245589288	4.89E-09	0.00745455
left amygdalar capsule	0.003344253	1.439065214	3.91E-07	0.230707003
left Anterior amygdalar area	0.014371697	2.050804557	8.50E-08	0.104962126
left Anterior area	0.066302418	2.806546257	1.81E-08	0.04763306
left Anterior cingulate area	0.074260381	2.220349125	5.51E-08	0.086554904
left Anterior cingulate area, ventral part	0.052496716	2.046705794	8.59E-08	0.105469342
left Anterior hypothalamic nucleus	0.02014271	2.524435497	2.89E-08	0.062511
left Anterior olfactory nucleus	0.06065975	1.417533716	4.33E-07	0.23616578
left Anterior pretectal nucleus	0.050601281	4.509781606	5.58E-09	0.014437957
left Anterior tegmental nucleus	0.002032287	3.050768996	1.37E-08	0.037258334
left Anterodorsal nucleus	0.010058518	4.477650691	5.49E-09	0.014437957
left Anterodorsal preoptic nucleus	0.002752579	2.934559158	1.51E-08	0.042556436
left Anterolateral visual area	0.023427131	1.750272253	1.62E-07	0.155548595
left Anteromedial nucleus, dorsal part	0.016961404	4.146245938	6.48E-09	0.016701326
left Anteromedial nucleus, ventral part	0.01131903	5.134842816	5.12E-09	0.012947224
left Anteromedial visual area	0.031179003	2.478066485	3.11E-08	0.065256185

left Anteroventral nucleus of thalamus	0.026188134	4.750333243	5.44E-09	0.014422307
left Anteroventral periventricular nucleus	0.007657487	3.478848974	9.62E-09	0.024827193
left Anteroventral preoptic nucleus	0.003447152	2.351306339	4.32E-08	0.073851424
left Arcuate hypothalamic nucleus	0.003026977	0.729343603	7.33E-06	0.525384014
left Area postrema	0.001457769	0.72116331	7.66E-06	0.529897882
left Area prostriata	0.008746568	2.019165704	9.14E-08	0.109292202
left auditory radiation	0.019919795	3.485678369	9.53E-09	0.024816546
left Barrington's nucleus	-0.000351575	-1.036884156	2.08E-06	0.370113031
left Basolateral amygdalar nucleus, anterior part	0.019808317	2.563814714	2.68E-08	0.060181163
left Basolateral amygdalar nucleus, posterior part	0.018633516	2.003850948	9.53E-08	0.11121241
left Basolateral amygdalar nucleus, ventral part	0.005282211	1.53331906	2.97E-07	0.204764514
left Basomedial amygdalar nucleus, anterior part	0.015555059	1.889454085	1.24E-07	0.128525639
left Basomedial amygdalar nucleus, posterior part	0.014474655	2.337910194	4.43E-08	0.074935727
left Bed nuclei of the stria terminalis	0.055703416	3.981981123	6.89E-09	0.018438655
left Bed nucleus of the accessory olfactory tract	0.000480202	1.138291636	1.24E-06	0.329710502
left Bed nucleus of the anterior commissure	0.000677426	2.208190098	5.61E-08	0.088005496
left brachium of the inferior colliculus	0.011087531	2.6286699	2.47E-08	0.055588919
left brachium of the superior colliculus	0.006928635	2.670280948	2.25E-08	0.053932371
left Caudoputamen	0.8151537	3.234036321	1.19E-08	0.030463735
left Central amygdalar nucleus	0.039916767	3.293485677	1.09E-08	0.029802285
left Central lateral nucleus of the thalamus	0.016001025	2.544886047	2.77E-08	0.061334326
left Central medial nucleus of the thalamus	0.005925394	1.720070534	1.84E-07	0.160794084
left cerebal peduncle	0.021754773	1.855539628	1.33E-07	0.134586411
left choroid plexus	0.030355658	1.781623259	1.52E-07	0.149056978
left cingulum bundle	0.063815498	5.98099854	4.95E-09	0.008786948
left Claustrum	0.017021492	3.637320144	8.69E-09	0.0216394
left columns of the fornix	0.013728629	4.408857232	5.65E-09	0.014437957
left commissural branch of stria terminalis	0.000823206	2.423286346	3.66E-08	0.068508705
left Copula pyramidis	0.032756897	0.706317183	9.24E-06	0.537003555
left Cortical amygdalar area, anterior part	0.010358577	1.069328605	1.75E-06	0.356429486
left Cortical amygdalar area, posterior part, lateral zone	0.028760556	2.16505266	6.25E-08	0.092320043
left Cortical amygdalar area, posterior part, medial zone	0.037575742	3.653146007	8.49E-09	0.021637953
left Cortical subplate	0.006422726	2.672082578	2.24E-08	0.053932371
left corticospinal tract	0.004527625	2.439648032	3.42E-08	0.067403702

left crossed tectospinal pathway	0.022998294	3.753036743	7.74E-09	0.02076658
left Crus 1	0.109127044	1.105040858	1.53E-06	0.342031984
left Crus 2	-0.000333901	-0.004668228	1.39E-01	0.996358892
left cuneate fascicle	0.000531651	0.850968363	4.83E-06	0.457488619
left Cuneate nucleus	0.008326411	1.086913322	1.61E-06	0.349841353
left Cuneiform nucleus	0.020991755	2.493978013	3.04E-08	0.064688366
left Dentate gyrus	0.236550385	3.242609146	1.14E-08	0.03032609
left Dentate nucleus	0.00398744	0.819823474	5.44E-06	0.474049718
left Diagonal band nucleus	0.026145233	2.844032229	1.71E-08	0.046045082
left dorsal acoustic stria	-2.57E-05	-0.047142736	1.54E-02	0.967012063
left Dorsal auditory area	0.040294248	2.356160584	4.22E-08	0.073470476
left Dorsal cochlear nucleus	0.004047437	0.33423693	1.59E-04	0.765991453
left dorsal limb	0.006011096	1.303068221	6.16E-07	0.272632202
left Dorsal motor nucleus of the vagus nerve	0.010375798	3.91421238	7.11E-09	0.019764937
left Dorsal nucleus raphe	0.002795467	1.367203811	5.00E-07	0.251832352
left Dorsal part of the lateral geniculate complex	0.021917864	2.203059638	5.66E-08	0.088393232
left Dorsal peduncular area	0.013034013	1.439442313	3.84E-07	0.230707003
left Dorsal premammillary nucleus	0.00521361	2.631828612	2.45E-08	0.055447069
left dorsal spinocerebellar tract	0.001843623	1.652934952	2.13E-07	0.175904926
left Dorsal tegmental nucleus	0.00462193	3.271904988	1.11E-08	0.030151432
left Dorsal terminal nucleus of the accessory optic tract	0.000900374	2.549673787	2.74E-08	0.061153979
left Dorsomedial nucleus of the hypothalamus	0.009303932	2.311850384	4.61E-08	0.077497743
left Ectorhinal area	0.072682213	3.983753215	6.92E-09	0.018438655
left Endopiriform nucleus, dorsal part	0.049649533	3.06997316	1.33E-08	0.036804518
left Endopiriform nucleus, ventral part	0.019431011	2.480933218	3.15E-08	0.065256185
left Entorhinal area, lateral part	0.185709942	3.728894521	7.78E-09	0.02076658
left Entorhinal area, medial part, dorsal zone	0.148150732	2.660223538	2.30E-08	0.054212512
left Ethmoid nucleus of the thalamus	0.010521581	4.127059424	6.60E-09	0.016711482
left external capsule	0.034325878	7.932888373	4.83E-09	0.00272439
left External cuneate nucleus	0.001200515	0.496140938	2.97E-05	0.6631472
left external medullary lamina of the thalamus	0.003652957	3.175379903	1.24E-08	0.032389702
left Facial motor nucleus	0.023058338	2.453017503	3.39E-08	0.066747867
left facial nerve	0.001860786	1.617533373	2.31E-07	0.18432499
left fasciculus retroflexus	0.006611364	3.608107035	8.86E-09	0.022251122

left Fasciola cinerea	0.001320578	0.781861841	6.42E-06	0.494464533
left Fastigial nucleus	0.019293897	2.485012681	3.18E-08	0.065256185
left Field CA1	0.474851026	7.533972541	4.85E-09	0.002952701
left Field CA2	0.021308968	4.066109219	6.68E-09	0.017198719
left Field CA3	0.272351647	5.055375844	5.06E-09	0.012947224
left Fields of Forel	0.00926107	3.64214098	8.53E-09	0.021637953
left fimbria	0.061688716	3.452671245	9.67E-09	0.025838207
left Flocculus	0.008583797	0.443146212	4.34E-05	0.697975067
left Frontal pole	0.017390221	2.095629997	7.47E-08	0.099239787
left Fundus of striatum	0.010941672	1.450458032	3.78E-07	0.227411353
left Gigantocellular reticular nucleus	0.083409494	2.665545052	2.28E-08	0.054042913
left Globus pallidus, external segment	0.050481339	2.854343556	1.68E-08	0.045717165
left Globus pallidus, internal segment	0.014714758	2.619000796	2.50E-08	0.056210341
left Gracile nucleus	0.003455744	1.916101152	1.18E-07	0.124062713
left Gustatory areas	0.047643162	2.826151419	1.75E-08	0.046778579
left Hippocampal formation	0.018779333	4.632365861	5.76E-09	0.014437957
left Hippocampo-amygdalar transition area	0.013917248	3.310780478	1.06E-08	0.029540377
left inferior cerebellar peduncle	0.022783922	2.725113512	2.07E-08	0.050986133
left Inferior colliculus	0.143838392	2.197388202	5.81E-08	0.088859216
left Inferior olivary complex	0.012733872	1.281288766	6.40E-07	0.280775875
left Inferior salivatory nucleus	0.0008918	2.480010113	3.09E-08	0.065256185
left Infracerebellar nucleus	0.003858784	2.283527634	4.81E-08	0.080320733
left Infralimbic area	0.015478076	1.32232323	5.59E-07	0.267189837
left Interanterodorsal nucleus of the thalamus	0.006962921	5.087651688	5.14E-09	0.012947224
left Intercalated amygdalar nucleus	0.003258533	1.232719738	8.21E-07	0.296050889
left Intergeniculate leaflet of the lateral geniculate complex	0.000746032	0.653712794	1.32E-05	0.565661336
left Intermediate geniculate nucleus	0.000480207	1.185567189	9.37E-07	0.314232788
left Intermediate reticular nucleus	0.105061426	2.802166132	1.82E-08	0.047643948
left internal capsule	0.125847029	4.394667359	5.46E-09	0.014437957
left Interposed nucleus	0.03433444	2.77975205	1.91E-08	0.04822422
left Interstitial nucleus of Cajal	0.00416747	4.37816005	5.51E-09	0.014437957
left Intertrigeminal nucleus	0.001492063	1.408770315	4.41E-07	0.238991456
left Koelliker-Fuse subnucleus	0.007580344	2.569890727	2.64E-08	0.059888677
left Lateral amygdalar nucleus	0.024644589	1.531681156	3.02E-07	0.204943199

left Lateral dorsal nucleus of thalamus	0.057881495	3.769226168	7.60E-09	0.02076658
left Lateral habenula	0.010667294	1.541627161	2.80E-07	0.203183437
left Lateral hypothalamic area	0.078581589	3.303498854	1.07E-08	0.029597394
left lateral lemniscus	0.014997879	1.49796207	3.48E-07	0.212870413
left Lateral mammillary nucleus	0.0018779	0.628268952	1.57E-05	0.579531355
left lateral olfactory tract, body	0.012399448	0.943098552	3.59E-06	0.409491468
left Lateral posterior nucleus of the thalamus	0.047402804	2.769151776	1.93E-08	0.048696787
left Lateral preoptic area	0.021729054	2.790880453	1.87E-08	0.047944415
left lateral recess	0.006285536	1.219473843	8.57E-07	0.300454124
left Lateral reticular nucleus, magnocellular part	0.010067168	1.609361662	2.38E-07	0.186110545
left Lateral reticular nucleus, parvicellular part	0.001372004	1.734512774	1.74E-07	0.158174243
left Lateral septal nucleus, caudal (caudodorsal) part	0.024078709	3.493892448	9.30E-09	0.024715094
left Lateral septal nucleus, rostral (rostroventral) part	0.097455259	3.399783206	1.01E-08	0.026865674
left Lateral septal nucleus, ventral part	0.031427526	3.445848135	9.72E-09	0.02587069
left Lateral terminal nucleus of the accessory optic tract	0.000754607	2.234776668	5.32E-08	0.08518895
left lateral ventricle	0.098750022	3.557870387	9.08E-09	0.022894006
left Lateral vestibular nucleus	0.008146325	2.303767043	4.65E-08	0.078416532
left Lateral visual area	0.042189409	1.988320315	1.00E-07	0.11304932
left Laterodorsal tegmental nucleus	0.006448431	3.311725293	1.06E-08	0.029540377
left Laterointermediate area	0.017767601	2.145740504	6.42E-08	0.094782105
left Linear nucleus of the medulla	0.0026497	2.095295013	7.55E-08	0.099239787
left Lingula (I)	0.004261807	1.503735747	3.37E-07	0.21165983
left Locus ceruleus	0.000343002	0.705515372	8.81E-06	0.537003555
left Magnocellular nucleus	0.008712166	1.201971778	8.96E-07	0.307772714
left Magnocellular reticular nucleus	0.022758166	3.586610192	8.99E-09	0.022514101
left Main olfactory bulb	0.275282886	1.236753427	8.04E-07	0.294760033
left mammillary peduncle	0.001114755	1.516195869	3.21E-07	0.208598021
left mammillotegmental tract	0.001972251	1.320743206	5.49E-07	0.267189837
left mammillothalamic tract	0.007700389	3.670203549	8.45E-09	0.021353414
left Medial accesory oculomotor nucleus	0.00118336	1.258663644	6.94E-07	0.288545797
left Medial amygdalar nucleus	0.052084685	3.772289072	7.64E-09	0.02076658
left medial corticohypothalamic tract	0.000145774	0.432530622	5.13E-05	0.703879775
left medial forebrain bundle	0.00181791	2.084395273	7.85E-08	0.100528865
left Medial geniculate complex, dorsal part	0.003052734	1.930620046	1.13E-07	0.121827234

left Medial geniculate complex, medial part	0.005993974	2.192466074	5.92E-08	0.089216861
left Medial geniculate complex, ventral part	0.008180626	2.368901701	4.18E-08	0.072197732
left Medial habenula	0.012896801	1.758283232	1.60E-07	0.154074846
left medial lemniscus	0.026822792	4.44048447	5.69E-09	0.014437957
left medial longitudinal fascicle	0.003850198	2.989828355	1.46E-08	0.039632355
left Medial mammillary nucleus	0.020957284	1.938838866	1.11E-07	0.120797167
left Medial preoptic area	0.02018559	3.009250534	1.42E-08	0.038766796
left Medial preoptic nucleus	0.016601262	5.624793696	5.04E-09	0.009572946
left Medial pretectal area	0.00198083	3.686118471	8.30E-09	0.021285691
left Medial terminal nucleus of the accessory optic tract	0.001191944	1.422239144	4.19E-07	0.235233971
left Medial vestibular nucleus	0.045936688	1.971370351	1.06E-07	0.115201866
left Mediodorsal nucleus of thalamus	0.042009121	3.207365375	1.22E-08	0.031040175
left Medullary reticular nucleus, dorsal part	0.054803172	3.367649915	1.02E-08	0.028089985
left Medullary reticular nucleus, ventral part	0.049769638	2.917306493	1.56E-08	0.042662245
left Midbrain reticular nucleus	0.201805366	4.124582286	6.54E-09	0.016711482
left Midbrain reticular nucleus, retrorubral area	0.006697109	4.245246972	6.19E-09	0.015563882
left Midbrain trigeminal nucleus	0.000360149	1.239184405	7.87E-07	0.294169272
left middle cerebellar peduncle	0.017853297	1.673072491	2.02E-07	0.171473409
left Motor nucleus of trigeminal	0.01472337	2.249784356	5.28E-08	0.083173148
left motor root of the trigeminal nerve	0.001946526	1.840783379	1.41E-07	0.136834053
left nigrostriatal tract	0.005170754	4.500589778	5.53E-09	0.014437957
left Nucleus accumbens	0.095543044	2.458541324	3.34E-08	0.066406347
left Nucleus ambiguus, dorsal division	0.000394455	0.572218444	2.26E-05	0.613752332
left Nucleus ambiguus, ventral division	0.000823216	1.04897339	2.02E-06	0.364502567
left Nucleus incertus	0.002118029	1.47316531	3.66E-07	0.220215182
left Nucleus of Darkschewitsch	0.005239334	4.022509512	6.83E-09	0.017974086
left Nucleus of reuniens	0.024576019	4.390972067	5.55E-09	0.014437957
left Nucleus of Roller	0.001552086	2.53227271	2.83E-08	0.062001701
left Nucleus of the brachium of the inferior colliculus	0.001877939	1.150780502	1.16E-06	0.325852718
left Nucleus of the lateral lemniscus	0.0114821	1.123936664	1.41E-06	0.334491533
left Nucleus of the lateral olfactory tract	0.008026202	2.186141395	6.03E-08	0.089593356
left Nucleus of the optic tract	0.008506433	3.323329867	1.05E-08	0.029377148
left Nucleus of the posterior commissure	0.008497842	3.01134687	1.43E-08	0.038766796
left Nucleus of the solitary tract	0.035586447	3.848722061	7.27E-09	0.019935335

left Nucleus of the trapezoid body	0.003773025	2.221411836	5.46E-08	0.086554904
left Nucleus prepositus	0.004587655	1.549336678	2.76E-07	0.201351572
left Nucleus sagulum	0.00162926	1.900137154	1.23E-07	0.126719281
left Nucleus x	0.002092314	3.890933288	7.08E-09	0.019764937
left Nucleus y	0.000814629	1.728806767	1.77E-07	0.159375338
left oculomotor nerve	0.000240103	1.138308573	1.28E-06	0.329710502
left Oculomotor nucleus	0.00155208	3.144889179	1.25E-08	0.033909936
left olfactory nerve layer of main olfactory bulb	0.039848273	1.072469822	1.70E-06	0.355935273
left Olfactory tubercle	0.034196856	0.579052178	2.12E-05	0.609861289
left Olivary pretectal nucleus	0.002658269	3.01590573	1.41E-08	0.038645663
left optic nerve	0.001286256	0.737309079	7.01E-06	0.521027153
left optic radiation	0.135305225	5.084285062	5.21E-09	0.012947224
left optic tract	0.027097007	2.657406667	2.33E-08	0.054310458
left Orbital area, lateral part	0.054563282	2.200363649	5.76E-08	0.08860188
left Orbital area, medial part	0.023478592	1.836189944	1.43E-07	0.137629255
left Orbital area, ventrolateral part	0.029901458	1.575111197	2.56E-07	0.194922641
left Parabigeminal nucleus	0.00072888	1.143453273	1.21E-06	0.328286977
left Parabrachial nucleus	0.039788229	2.861199123	1.66E-08	0.045446122
left Paracentral nucleus	0.015066384	3.233003835	1.20E-08	0.030463735
left Parafascicular nucleus	0.027508792	2.921259345	1.55E-08	0.042662245
left Paraflocculus	0.060145999	0.948805701	3.47E-06	0.407560128
left Paragigantocellular reticular nucleus, dorsal part	0.005333677	1.24304889	7.38E-07	0.293120038
left Paragigantocellular reticular nucleus, lateral part	0.02370148	3.028552311	1.38E-08	0.038597388
left Paramedian lobule	0.032860097	0.462847512	4.01E-05	0.684498052
left Paranigral nucleus	0.000986131	1.649031015	2.19E-07	0.176403981
left Parapyramidal nucleus	0.002306705	1.536880923	2.93E-07	0.20396117
left Parasolitary nucleus	0.000222956	0.261754708	7.89E-04	0.810985892
left Parastrial nucleus	0.003524331	3.650692413	8.57E-09	0.021637953
left Parasubiculum	0.023804327	2.044680721	8.68E-08	0.105469342
left Parasubthalamic nucleus	0.005805307	2.969146111	1.46E-08	0.040783876
left Parataenial nucleus	0.007151564	2.383133879	4.08E-08	0.070940536
left Paratrigeminal nucleus	0.001303426	0.739866943	6.71E-06	0.520133846
left Paratrochlear nucleus	0.000591681	1.166046092	1.05E-06	0.320740509
left Paraventricular hypothalamic nucleus	0.01743303	3.620360199	8.74E-09	0.022103493

left Paraventricular nucleus of the thalamus	0.008849333	1.160365048	1.08E-06	0.322880505
left Parvicellular motor 5 nucleus	0.001843653	0.992895841	3.02E-06	0.386241787
left Parvicellular reticular nucleus	0.080819893	3.500740154	9.34E-09	0.024715094
left Pedunculopontine nucleus	0.041614663	4.281776651	6.21E-09	0.015563882
left Perifornical nucleus	0.007597488	3.493072124	9.43E-09	0.024715094
left Peripeduncular nucleus	0.00339571	5.902767564	4.96E-09	0.008786948
left Perireunensis nucleus	0.008060537	4.965970037	5.10E-09	0.012947224
left Perirhinal area	0.029017984	3.439623494	9.76E-09	0.02587069
left Peritrigeminal zone	0.01134478	2.483466809	3.20E-08	0.065256185
left Periventricular hypothalamic nucleus	0.023941516	4.252678176	6.16E-09	0.015563882
left Piriform area	0.277745047	2.440571184	3.45E-08	0.067403702
left Piriform-amygdalar area	0.015229173	1.105905354	1.49E-06	0.342031984
left Pontine central gray	0.01345423	2.085028563	7.93E-08	0.100528865
left Pontine gray	0.01582946	1.006349325	2.65E-06	0.381262894
left Pontine reticular nucleus	0.08696825	2.917958328	1.53E-08	0.042662245
left Pontine reticular nucleus, caudal part	0.066199608	2.276161308	4.89E-08	0.080934447
left Posterior amygdalar nucleus	0.03210492	4.416234257	5.72E-09	0.014437957
left Posterior auditory area	0.01897666	1.928798933	1.12E-07	0.121827234
left Posterior complex of the thalamus	0.064853166	2.944168772	1.49E-08	0.042295696
left Posterior hypothalamic nucleus	0.034145931	2.713194933	2.11E-08	0.051367794
left Posterior intralaminar thalamic nucleus	0.006405576	2.503281773	3.00E-08	0.063987985
left Posterior limiting nucleus of the thalamus	0.006782848	3.676350428	8.33E-09	0.021285691
left Posterior pretectal nucleus	0.004570494	3.065048899	1.33E-08	0.036804518
left Posterior triangular thalamic nucleus	0.007914774	3.071870057	1.34E-08	0.036804518
left Posterodorsal preoptic nucleus	0.000188652	0.623917907	1.76E-05	0.581662193
left Posterodorsal tegmental nucleus	0.001054723	0.99743772	2.83E-06	0.384986327
left Posterolateral visual area	0.019508349	1.670249219	2.04E-07	0.17195344
left posteromedial visual area	0.042240914	1.994328206	9.73E-08	0.112349479
left Postpiriform transition area	0.02837468	1.597497431	2.45E-07	0.189033405
left Postrhinal area	0.034977847	1.743277331	1.68E-07	0.156529958
left Postsubiculum	0.045447776	3.43804976	9.81E-09	0.02587069
left Precommissural nucleus	0.00353289	1.137123441	1.31E-06	0.329756764
left Prelimbic area	0.055369328	2.675699421	2.22E-08	0.053758659
left Preparasubthalamic nucleus	0.00018007	0.34565348	1.40E-04	0.758788166

left Presubiculum	0.02563075	2.062211997	8.25E-08	0.103571032
left Primary auditory area	0.070101136	2.916795706	1.54E-08	0.042662245
left Primary motor area	0.217155739	2.121242051	6.92E-08	0.097216494
left Primary somatosensory area, barrel field	0.199884942	2.386525177	4.05E-08	0.070695869
left Primary somatosensory area, lower limb	0.07416575	3.492165671	9.39E-09	0.024715094
left Primary somatosensory area, mouth	0.127940224	2.115098824	7.06E-08	0.097427205
left Primary somatosensory area, nose	0.08036565	2.132309933	6.73E-08	0.095999744
left Primary somatosensory area, trunk	0.059133624	3.256831051	1.15E-08	0.03032609
left Primary somatosensory area, unassigned	0.036769872	2.396684004	3.99E-08	0.069792108
left Primary somatosensory area, upper limb	0.097987277	2.528589261	2.87E-08	0.062229558
left Primary visual area	0.251095545	1.956556582	1.08E-07	0.117677041
left principal mammillary tract	0.001209081	2.16075684	6.31E-08	0.092804169
left Principal sensory nucleus of the trigeminal	0.028906635	1.550087632	2.72E-07	0.201351572
left Prosubiculum	0.062186158	5.138441098	5.17E-09	0.012947224
left pyramid	0.005419395	1.019138778	2.42E-06	0.376477268
left Red nucleus	0.028880813	3.224943962	1.18E-08	0.030463735
left Reticular nucleus of the thalamus	0.084429635	3.560690683	9.12E-09	0.022894006
left Retrochiasmatic area	0.003575788	1.334669929	5.28E-07	0.262897452
left Retroparafascicular nucleus	0.002744018	2.723744088	2.08E-08	0.050986133
left Retrosplenial area, dorsal part	0.105533486	2.466159969	3.27E-08	0.066102521
left Retrosplenial area, lateral agranular part	0.091693183	3.088139027	1.29E-08	0.036160924
left Retrosplenial area, ventral part	0.08068312	1.654642765	2.10E-07	0.175748266
left Rostrolateral area	0.041546118	2.859788802	1.67E-08	0.045446122
left rubrospinal tract	0.0129912	3.560020592	9.16E-09	0.022894006
left Secondary motor area	0.18503374	1.662637964	2.07E-07	0.17378736
left sensory root of the trigeminal nerve	0.01711583	3.870803265	7.23E-09	0.019935335
left Septohippocampal nucleus	0.00227239	2.478207776	3.13E-08	0.065256185
left Simple lobule	0.014638925	0.166773542	4.08E-03	0.877411362
left solitary tract	-0.000351576	-1.365361549	5.09E-07	0.252128176
left Spinal nucleus of the trigeminal, caudal part	0.04858636	2.644070718	2.39E-08	0.054589968
left Spinal nucleus of the trigeminal, interpolar part	0.047977631	2.331929059	4.50E-08	0.075357084
left Spinal nucleus of the trigeminal, oral part	0.035920907	3.223358056	1.18E-08	0.030463735
left spinal tract of the trigeminal nerve	0.053937091	4.356053747	5.74E-09	0.014437957
left Spinal vestibular nucleus	0.015735351	1.482862155	3.54E-07	0.217623814

left stria medullaris	0.015632271	3.728480134	7.71E-09	0.02076658
left stria terminalis	0.015186342	2.796900695	1.85E-08	0.047720246
left Subceruleus nucleus	0.00052308	1.03086667	2.27E-06	0.371541591
left subependymal zone	0.007991947	3.601189656	8.82E-09	0.022251122
left Subgeniculate nucleus	0.001114755	1.976575409	1.04E-07	0.114643484
left Subiculum	0.117726635	4.87599413	5.38E-09	0.013010395
left Sublaterodorsal nucleus	-0.000385877	-0.378572909	1.11E-04	0.73610943
left Submedial nucleus of the thalamus	0.02364998	4.423253959	5.60E-09	0.014437957
left Subparafascicular area	0.006971506	2.755454472	1.98E-08	0.049162557
left Subparafascicular nucleus	0.012510997	4.709691384	5.62E-09	0.014437957
left Subparaventricular zone	0.003833033	1.941965114	1.10E-07	0.120396732
left Substantia innominata	0.080030473	2.04263274	8.86E-08	0.105562555
left Substantia nigra, compact part	0.002581129	0.798216942	6.16E-06	0.485107678
left Substantia nigra, reticular part	0.025819693	1.245130526	7.23E-07	0.293120038
left Subthalamic nucleus	0.007631776	3.893455874	7.04E-09	0.019764937
left Superior central nucleus raphe	0.023752937	2.489770095	3.06E-08	0.064990771
left superior cerebelar peduncles	0.028820721	3.305606188	1.08E-08	0.029597394
left Superior colliculus	0.188857316	1.982562547	1.02E-07	0.113936592
left Superior olivary complex, lateral part	0.010573035	2.348986246	4.28E-08	0.073851424
left Superior olivary complex, medial part	0.004467608	2.376152735	4.15E-08	0.071458628
left Superior olivary complex, periolivary region	0.005650969	1.057278023	1.90E-06	0.361393495
left Superior vestibular nucleus	0.009792738	1.72641922	1.79E-07	0.159708478
left Supplemental somatosensory area	0.248934834	2.418480766	3.72E-08	0.068732612
left supra-callosal cerebral white matter	0.05445151	5.053207208	5.19E-09	0.012947224
left Suprachiasmatic nucleus	0.001157632	1.153201185	1.13E-06	0.325221861
left Suprageniculate nucleus	0.006182621	2.817589534	1.79E-08	0.047042216
left Supragenual nucleus	0.000265825	0.686966306	1.13E-05	0.546040543
left Supramammillary nucleus	0.009175268	3.273420706	1.11E-08	0.030151432
left Supraoculomotor periaqueductal gray	0.001526351	3.003343783	1.44E-08	0.039012888
left supraoptic commissures	0.000994702	2.270778201	5.01E-08	0.081313203
left Supraoptic nucleus	0.000403025	0.702500838	9.71E-06	0.538202995
left Supratrigeminal nucleus	0.010538708	2.403797236	3.80E-08	0.069587455
left Taenia tecta, dorsal part	0.01564937	1.279235412	6.53E-07	0.281186081
left Taenia tecta, ventral part	0.000274378	0.024714568	2.80E-02	0.983279256

left Tegmental reticular nucleus	0.027748923	2.642863989	2.40E-08	0.054589968
left Temporal association areas	0.109821018	2.835425763	1.73E-08	0.04641185
left trapezoid body	0.004544733	1.27178094	6.67E-07	0.283616058
left trochlear nerve	0.000300127	1.418720338	4.26E-07	0.236125601
left Trochlear nucleus	-0.000171504	-0.57049003	2.42E-05	0.614106174
left Tuberal nucleus	0.006851408	0.710812376	8.02E-06	0.535081763
left Tuberomammillary nucleus, dorsal part	-0.00020579	-0.369564884	1.25E-04	0.741845103
left Tuberomammillary nucleus, ventral part	0.001569222	0.638285655	1.48E-05	0.574394113
left uncinate fascicle	0.003532924	2.253956392	5.10E-08	0.083049896
left Ventral anterior-lateral complex of the thalamus	0.048637628	3.747118605	7.67E-09	0.02076658
left Ventral auditory area	0.069792406	4.115214091	6.57E-09	0.016711482
left Ventral cochlear nucleus	0.012699746	1.013703365	2.49E-06	0.378443378
left Ventral medial nucleus of the thalamus	0.078392973	4.416822993	5.67E-09	0.014437957
left Ventral part of the lateral geniculate complex	0.011756387	2.182947121	6.14E-08	0.089890139
left Ventral posterolateral nucleus of the thalamus	0.047968781	2.53946031	2.79E-08	0.061596292
left Ventral posteromedial nucleus of the thalamus	0.11012083	2.838030994	1.72E-08	0.046368419
left Ventral premammillary nucleus	0.005702379	2.194683578	5.87E-08	0.089075533
left ventral spinocerebellar tract	0.002529638	1.060808672	1.85E-06	0.360158027
left Ventral tegmental area	0.014491875	2.511106603	2.96E-08	0.063283236
left Ventral tegmental nucleus	0.001672133	2.911792962	1.59E-08	0.042876245
left Ventrolateral preoptic nucleus	0.000780327	0.655175705	1.25E-05	0.5654756
left Ventromedial hypothalamic nucleus	0.006096839	1.097344588	1.57E-06	0.345253245
left Ventromedial preoptic nucleus	0.001114754	0.934317907	4.15E-06	0.412520075
left vestibular nerve	0.009175305	2.39922511	3.96E-08	0.069657177
left Vestibulocerebellar nucleus	0.002598259	1.376722996	4.91E-07	0.248596453
left Visceral area	0.063369968	2.416313088	3.75E-08	0.068816837
left vomeronasal nerve	7.72E-05	0.183144716	2.90E-03	0.865888209
left Xiphoid thalamic nucleus	0.003267081	2.137518741	6.61E-08	0.095423361
left Zona incerta	0.07123279	5.119823445	5.08E-09	0.012947224
Lobule II	0.122408947	1.688213713	1.96E-07	0.167887995
Lobule III	0.103776149	0.666278007	1.19E-05	0.558882954
Lobules IV-V	0.381531606	1.456472202	3.72E-07	0.225666074
Medial septal nucleus	0.035346317	3.842731539	7.30E-09	0.019935335
Median eminence	0.002778313	1.857992371	1.31E-07	0.134478647

Median preoptic nucleus	0.003558643	2.252676244	5.14E-08	0.083049896
Nodulus (X)	0.046734646	0.562618546	2.58E-05	0.618727111
Nucleus raphe magnus	0.014397534	2.104754504	7.40E-08	0.098519369
Nucleus raphe obscurus	0.003455727	1.157169176	1.10E-06	0.323869424
Nucleus raphe pallidus	0.002220904	0.952464536	3.35E-06	0.406157597
Nucleus raphe pontis	0.004150327	2.017751865	9.23E-08	0.109292202
optic chiasm	0.010272875	0.984505005	3.13E-06	0.390103299
Periaqueductal gray	0.368606975	4.181566883	6.40E-09	0.016557529
posterior commissure	0.003267078	2.186949494	6.08E-08	0.089593356
pyramidal decussation	0.001363442	0.425990321	6.12E-05	0.706785853
Pyramus (VIII)	0.025827963	0.395118908	9.00E-05	0.725758058
Rhomboid nucleus	0.006465592	2.895204298	1.60E-08	0.043994394
right Abducens nucleus	7.72E-05	0.316780025	2.06E-04	0.776959928
right Accessory facial motor nucleus	-6.00E-05	-0.285959431	5.31E-04	0.79467117
right Accessory olfactory bulb	0.005676614	0.470445253	3.71E-05	0.67989745
right Accessory supraoptic group	-0.000214374	-1.847121853	1.38E-07	0.135905854
right Accessory trigeminal nucleus	0.000188649	0.399041391	8.14E-05	0.723821124
right Agranular insular area, dorsal part	0.097472879	2.274497126	4.97E-08	0.080977169
right Agranular insular area, posterior part	0.077981523	3.524696824	9.25E-09	0.024108062
right Agranular insular area, ventral part	0.058816201	2.651877969	2.37E-08	0.054336389
right alveus	0.0552918	8.819176162	4.81E-09	0.001967727
right amygdalar capsule	0.00208371	0.94398458	3.72E-06	0.409491468
right Anterior amygdalar area	0.016901357	2.701132067	2.17E-08	0.052044429
right Anterior area	0.050618679	2.143489875	6.54E-08	0.094782105
right Anterior cingulate area	0.079902628	2.029786204	8.95E-08	0.107696862
right Anterior cingulate area, ventral part	0.042489717	1.560106326	2.68E-07	0.19861222
right Anterior hypothalamic nucleus	0.015709408	2.013737632	9.33E-08	0.109793526
right Anterior olfactory nucleus	0.068274479	1.697316164	1.91E-07	0.165818233
right Anterior pretectal nucleus	0.043381164	4.121015748	6.63E-09	0.016711482
right Anterior tegmental nucleus	0.000617404	1.244625983	7.54E-07	0.293120038
right Anterodorsal nucleus	0.010032774	3.71734501	7.81E-09	0.02076658
right Anterodorsal preoptic nucleus	0.003009832	2.51774756	2.91E-08	0.063077846
right Anterolateral visual area	0.022449621	1.743085727	1.70E-07	0.156529958
right Anteromedial nucleus, dorsal part	0.017544502	4.998008929	5.33E-09	0.012947224

right Anteromedial nucleus, ventral part	0.011130388	4.26364881	6.35E-09	0.015563882
right Anteromedial visual area	0.031041757	1.855217005	1.35E-07	0.134586411
right Anteroventral nucleus of thalamus	0.024713203	3.76523203	7.85E-09	0.02076658
right Anteroventral periventricular nucleus	0.008369228	4.476962498	5.96E-09	0.014437957
right Anteroventral preoptic nucleus	0.002289535	1.696893258	1.94E-07	0.165818233
right Arcuate hypothalamic nucleus	6.00E-05	0.01399536	5.77E-02	0.99037049
right Area postrema	0.004193201	2.476098241	3.22E-08	0.065304269
right Area prostriata	0.019285304	3.247489268	1.17E-08	0.03032609
right auditory radiation	0.021909186	3.713804196	8.26E-09	0.02076658
right Barrington's nucleus	0.000574527	1.631559675	2.28E-07	0.180507648
right Basolateral amygdalar nucleus, anterior part	0.031213098	3.181490945	1.23E-08	0.032224492
right Basolateral amygdalar nucleus, posterior part	0.019928314	2.137052223	6.67E-08	0.095423361
right Basolateral amygdalar nucleus, ventral part	0.010710196	2.718327772	2.10E-08	0.05131053
right Basomedial amygdalar nucleus, anterior part	0.0296096	4.373850953	5.98E-09	0.014437957
right Basomedial amygdalar nucleus, posterior part	0.020717251	3.375804005	1.02E-08	0.027865625
right Bed nuclei of the stria terminalis	0.056612366	3.768742043	7.92E-09	0.02076658
right Bed nucleus of the accessory olfactory tract	0.001149055	2.85190399	1.69E-08	0.045743854
right Bed nucleus of the anterior commissure	0.001114754	3.087520059	1.30E-08	0.036160924
right brachium of the inferior colliculus	0.005522351	1.973266081	1.05E-07	0.115061007
right brachium of the superior colliculus	0.004750582	2.128773361	6.79E-08	0.09637781
right Caudoputamen	0.865135418	4.291639692	6.29E-09	0.015563882
right Central amygdalar nucleus	0.060428159	4.057120548	6.77E-09	0.017198719
right Central lateral nucleus of the thalamus	0.012656758	2.114946684	7.12E-08	0.097427205
right Central medial nucleus of the thalamus	0.007760429	2.712949489	2.14E-08	0.051367794
right cerebal peduncle	0.029489436	2.094328282	7.70E-08	0.099239787
right choroid plexus	0.031341555	1.524446382	3.12E-07	0.206241248
right cingulum bundle	0.062151966	4.827202102	5.42E-09	0.013530584
right Claustrum	0.015315052	2.744931662	2.03E-08	0.049756675
right columns of the fornix	0.015820931	6.19360118	4.91E-09	0.00745455
right commissural branch of stria terminalis	0.000660281	1.403495045	4.49E-07	0.240546511
right Copula pyramidis	0.047231635	1.074557528	1.66E-06	0.355431192
right Cortical amygdalar area, anterior part	0.026385329	2.940814273	1.50E-08	0.042375807
right Cortical amygdalar area, posterior part, lateral zone	0.033871254	2.438032898	3.55E-08	0.067403702
right Cortical amygdalar area, posterior part, medial zone	0.044109899	3.409904455	1.01E-08	0.026546464

right Cortical subplate	0.010075637	3.144319134	1.26E-08	0.033909936
right corticospinal tract	0.003378567	2.992314226	1.45E-08	0.039622424
right crossed tectospinal pathway	0.017441682	2.226541525	5.42E-08	0.086022212
right Crus 1	0.204361396	1.826814774	1.45E-07	0.139542662
right Crus 2	0.083676134	0.80138031	5.90E-06	0.483867226
right cuneate fascicle	-7.72E-05	-0.153125848	5.99E-03	0.886830519
right Cuneate nucleus	0.012176607	2.27624203	4.93E-08	0.080934447
right Cuneiform nucleus	0.021163275	2.604511528	2.55E-08	0.05715762
right Dentate gyrus	0.218208405	3.980986376	6.95E-09	0.018438655
right Dentate nucleus	0.011747821	2.869523799	1.64E-08	0.045176827
right Diagonal band nucleus	0.024361665	2.612901633	2.54E-08	0.056479817
right dorsal acoustic stria	0.000325853	1.170375762	1.03E-06	0.319234538
right Dorsal auditory area	0.036684227	2.113600332	7.26E-08	0.097442583
right Dorsal cochlear nucleus	0.021686271	1.583129294	2.52E-07	0.192770044
right dorsal limb	0.003558647	0.817920651	5.67E-06	0.474517847
right Dorsal motor nucleus of the vagus nerve	0.010547307	3.564274524	9.21E-09	0.022894006
right Dorsal nucleus raphe	0.001389192	0.47446843	3.44E-05	0.677900206
right Dorsal part of the lateral geniculate complex	0.029077982	3.229411417	1.21E-08	0.030463735
right Dorsal peduncular area	0.01139621	1.429397008	4.04E-07	0.23341593
right Dorsal premammillary nucleus	0.003875913	2.125193335	6.86E-08	0.096764941
right dorsal spinocerebellar tract	0.00292408	2.93239447	1.52E-08	0.042556436
right Dorsal tegmental nucleus	0.004193192	4.242202758	6.32E-09	0.015563882
right Dorsal terminal nucleus of the accessory optic tract	0.000514502	2.056912254	8.33E-08	0.104301363
right Dorsomedial nucleus of the hypothalamus	0.006156911	1.675587251	1.99E-07	0.171081149
right Ectorhinal area	0.083049422	3.750754469	8.14E-09	0.02076658
right Endopiriform nucleus, dorsal part	0.052762244	2.559007794	2.70E-08	0.060519067
right Endopiriform nucleus, ventral part	0.024730385	2.732747205	2.04E-08	0.050509999
right Entorhinal area, lateral part	0.21814084	2.969802137	1.47E-08	0.040783876
right Entorhinal area, medial part, dorsal zone	0.203348431	2.814423794	1.80E-08	0.047137338
right Ethmoid nucleus of the thalamus	0.007768998	2.926937309	1.58E-08	0.042662245
right external capsule	0.039110737	6.266433599	4.93E-09	0.00745455
right External cuneate nucleus	0.008506456	2.514333894	2.93E-08	0.063280644
right external medullary lamina of the thalamus	0.004673379	3.099941193	1.28E-08	0.036057403
right Facial motor nucleus	0.030167025	3.708174845	8.07E-09	0.02076658

right facial nerve	0.003112743	2.667658984	2.27E-08	0.054011838
right fasciculus retroflexus	0.007571743	5.815211406	5.00E-09	0.00893279
right Fasciola cinerea	0.002169509	1.129340634	1.38E-06	0.332428969
right Fastigial nucleus	0.021429081	2.786762025	1.90E-08	0.047958755
right Field CA1	0.497103232	6.679410281	4.87E-09	0.006681417
right Field CA2	0.018616374	3.64395434	8.61E-09	0.021637953
right Field CA3	0.286706042	4.921201375	5.29E-09	0.012947224
right Fields of Forel	0.011867868	4.549935858	6.11E-09	0.014437957
right fimbria	0.062871881	2.771716591	1.96E-08	0.048696787
right Flocculus	0.04185493	1.505496995	3.32E-07	0.211432988
right Frontal pole	0.020974533	1.88960191	1.25E-07	0.128525639
right Fundus of striatum	0.012630946	1.428081882	4.11E-07	0.233501378
right Gigantocellular reticular nucleus	0.109563355	3.252450581	1.17E-08	0.03032609
right Globus pallidus, external segment	0.062160383	4.006020112	6.86E-09	0.018274991
right Globus pallidus, internal segment	0.02256944	3.739929818	8.18E-09	0.02076658
right Gracile nucleus	0.004244663	2.653903914	2.36E-08	0.054310458
right Gustatory areas	0.052187872	3.676429454	8.37E-09	0.021285691
right Hippocampal formation	0.021334669	3.909375393	7.20E-09	0.019764937
right Hippocampo-amygdalar transition area	0.013299854	3.284152678	1.10E-08	0.030137227
right inferior cerebellar peduncle	0.033562758	3.595586808	8.95E-09	0.022315594
right Inferior colliculus	0.189491843	2.614984021	2.52E-08	0.056441965
right Inferior olivary complex	0.012648138	1.76173515	1.58E-07	0.153483918
right Inferior salivatory nucleus	0.000437331	1.069818498	1.80E-06	0.356429486
right Infracerebellar nucleus	0.004930655	2.875408381	1.62E-08	0.045050275
right Infralimbic area	0.011602163	1.032282314	2.20E-06	0.371367478
right Interanterodorsal nucleus of the thalamus	0.004990649	3.426335179	9.91E-09	0.026252172
right Intercalated amygdalar nucleus	0.005736713	2.555211815	2.72E-08	0.060749856
right Intergeniculate leaflet of the lateral geniculate complex	0.003224219	3.409884368	1.00E-08	0.026546464
right Intermediate geniculate nucleus	0.000711729	1.86994087	1.28E-07	0.132312314
right Intermediate reticular nucleus	0.119193101	4.100288337	6.66E-09	0.01692587
right internal capsule	0.134884912	4.055231012	6.74E-09	0.017198719
right Interposed nucleus	0.044350143	3.208683397	1.22E-08	0.031040175
right Interstitial nucleus of Cajal	0.00548802	4.608411792	5.84E-09	0.014437957
right Intertrigeminal nucleus	0.001474911	1.25269385	7.08E-07	0.29064881

right Koelliker-Fuse subnucleus	0.005968242	2.378827085	4.12E-08	0.071301719
right Lateral amygdalar nucleus	0.031264443	1.52472619	3.16E-07	0.206241248
right Lateral dorsal nucleus of thalamus	0.056097843	4.597682727	5.88E-09	0.014437957
right Lateral habenula	0.00532505	0.938369782	4.00E-06	0.410891785
right Lateral hypothalamic area	0.091358315	4.436356009	5.93E-09	0.014437957
right lateral lemniscus	0.022235195	2.253429615	5.19E-08	0.083049896
right Lateral mammillary nucleus	0.001912202	0.62852088	1.66E-05	0.579531355
right lateral olfactory tract, body	0.026102378	1.872830223	1.27E-07	0.131926018
right Lateral posterior nucleus of the thalamus	0.045027518	3.313561534	1.07E-08	0.029540377
right Lateral preoptic area	0.016978541	2.712136377	2.12E-08	0.051367794
right lateral recess	0.00563384	1.053335678	1.96E-06	0.362839741
right Lateral reticular nucleus, magnocellular part	0.015786706	3.065961869	1.35E-08	0.036804518
right Lateral reticular nucleus, parvicellular part	0.001423453	2.40588053	3.93E-08	0.069587455
right Lateral septal nucleus, caudal (caudodorsal) part	0.024558813	3.352178311	1.04E-08	0.028230329
right Lateral septal nucleus, rostral (rostroventral) part	0.104683729	3.951147746	7.01E-09	0.018984073
right Lateral septal nucleus, ventral part	0.039942465	5.276317724	5.23E-09	0.012947224
right Lateral terminal nucleus of the accessory optic tract	0.001560653	3.251007501	1.16E-08	0.03032609
right lateral ventricle	0.093535849	2.065215828	8.17E-08	0.103254021
right Lateral vestibular nucleus	0.012991215	3.482651538	9.57E-09	0.024816546
right Lateral visual area	0.041297754	1.568457513	2.60E-07	0.196319964
right Laterodorsal tegmental nucleus	0.007100118	3.822004117	7.57E-09	0.020015083
right Laterointermediate area	0.014217578	1.310954072	5.92E-07	0.270287636
right Linear nucleus of the medulla	0.001534941	1.929453782	1.15E-07	0.121827234
right Lingula (I)	0.003490075	0.941784327	3.85E-06	0.409616068
right Locus ceruleus	0.000394455	1.023860361	2.34E-06	0.374593253
right Magnocellular nucleus	0.011764909	1.782240311	1.54E-07	0.149056978
right Magnocellular reticular nucleus	0.022252312	2.661877239	2.31E-08	0.054212512
right Main olfactory bulb	0.261185451	0.982005617	3.23E-06	0.390856831
right mammillary peduncle	0.000686001	0.700360804	1.02E-05	0.538835433
right mammillotegmental tract	0.00445904	1.978014778	1.03E-07	0.114593352
right mammillothalamic tract	0.008600762	4.935279022	5.31E-09	0.012947224
right Medial accesory oculomotor nucleus	0.001226235	1.219934241	8.77E-07	0.300454124
right Medial amygdalar nucleus	0.094916968	5.342103285	5.25E-09	0.012947224
right medial corticohypothalamic tract	0.000471624	1.335532523	5.38E-07	0.262897452

right medial forebrain bundle	0.00177503	2.654064593	2.34E-08	0.054310458
right Medial geniculate complex, dorsal part	0.006268361	3.604331408	8.90E-09	0.022251122
right Medial geniculate complex, medial part	0.009020947	3.099408767	1.28E-08	0.036057403
right Medial geniculate complex, ventral part	0.010598772	2.462936784	3.30E-08	0.066300114
right Medial habenula	0.010204227	1.384929316	4.65E-07	0.246704007
right medial lemniscus	0.03087019	3.840459908	7.40E-09	0.019935335
right medial longitudinal fascicle	0.008257742	3.242659522	1.15E-08	0.03032609
right Medial mammillary nucleus	0.018144678	1.859353714	1.30E-07	0.134431422
right Medial preoptic area	0.019671093	2.872951966	1.63E-08	0.045073974
right Medial preoptic nucleus	0.020022699	4.35728244	6.03E-09	0.014437957
right Medial pretectal area	0.000737456	1.005626003	2.74E-06	0.381262894
right Medial terminal nucleus of the accessory optic tract	0.002040861	1.779657264	1.56E-07	0.149263146
right Medial vestibular nucleus	0.056080848	2.756198829	1.99E-08	0.049162557
right Mediodorsal nucleus of thalamus	0.036632621	2.425516591	3.63E-08	0.068418597
right Medullary reticular nucleus, dorsal part	0.062400725	4.149274943	6.51E-09	0.016701326
right Medullary reticular nucleus, ventral part	0.049083651	2.822775112	1.77E-08	0.046778579
right Midbrain reticular nucleus	0.201042261	3.904051329	7.14E-09	0.019764937
right Midbrain reticular nucleus, retrorubral area	0.005771013	2.440502646	3.52E-08	0.067403702
right Midbrain trigeminal nucleus	0.000668849	2.402336691	3.86E-08	0.069587455
right middle cerebellar peduncle	0.037876042	2.539216125	2.81E-08	0.061596292
right Motor nucleus of trigeminal	0.014225986	2.864399144	1.65E-08	0.045418219
right motor root of the trigeminal nerve	0.001414883	1.437012431	3.98E-07	0.231056333
right nigrostriatal tract	0.006139712	4.605937942	5.86E-09	0.014437957
right Nucleus accumbens	0.118532605	2.598616037	2.57E-08	0.057587183
right Nucleus ambiguus, dorsal division	0.000523081	0.643530479	1.40E-05	0.57167325
right Nucleus ambiguus, ventral division	-0.000265827	-0.416690665	6.71E-05	0.71268703
right Nucleus incertus	0.001749312	1.378675991	4.82E-07	0.248261924
right Nucleus of Darkschewitsch	0.006105401	3.263807161	1.13E-08	0.03026965
right Nucleus of reuniens	0.027868812	4.449111897	5.91E-09	0.014437957
right Nucleus of Roller	0.001706435	2.436934941	3.50E-08	0.067403702
right Nucleus of the brachium of the inferior colliculus	0.002606817	3.180931183	1.24E-08	0.032224492
right Nucleus of the lateral lemniscus	0.013342884	1.321689116	5.70E-07	0.267189837
right Nucleus of the lateral olfactory tract	0.012845375	3.711074121	8.22E-09	0.02076658
right Nucleus of the optic tract	0.010410103	4.469319549	6.06E-09	0.014437957

right Nucleus of the posterior commissure	0.005419408	2.321996505	4.57E-08	0.076311811
right Nucleus of the solitary tract	0.053611128	4.80977266	5.40E-09	0.013530584
right Nucleus of the trapezoid body	0.004759144	2.824164214	1.76E-08	0.046778579
right Nucleus prepositus	0.006697131	2.067576494	8.09E-08	0.103052604
right Nucleus sagulum	0.002058023	1.703255337	1.89E-07	0.16469743
right Nucleus x	0.001629277	1.19869283	9.16E-07	0.308765185
right Nucleus y	0.000763182	1.928000936	1.17E-07	0.121827234
right oculomotor nerve	-0.000137197	-0.313213844	2.73E-04	0.778167869
right Oculomotor nucleus	0.001440605	1.651878205	2.16E-07	0.175904926
right olfactory nerve layer of main olfactory bulb	0.032593715	0.825141562	5.22E-06	0.471526664
right Olfactory tubercle	0.083134781	1.508898297	3.27E-07	0.210659704
right Olivary pretectal nucleus	0.002709712	3.867641771	7.43E-09	0.019935335
right optic nerve	0.000531654	0.309599826	3.18E-04	0.77986907
right optic radiation	0.127879425	4.901082687	5.35E-09	0.012947224
right optic tract	0.032164887	2.880802181	1.61E-08	0.044791278
right Orbital area, lateral part	0.059476681	2.070818171	8.01E-08	0.102695638
right Orbital area, medial part	0.028134665	1.931711704	1.16E-07	0.121827234
right Orbital area, ventrolateral part	0.033888659	1.501336156	3.43E-07	0.212101011
right Parabigeminal nucleus	0.00190367	2.216073809	5.56E-08	0.087002248
right Parabrachial nucleus	0.043303996	3.498496856	9.48E-09	0.024715094
right Paracentral nucleus	0.013471449	2.34404615	4.39E-08	0.074313065
right Parafascicular nucleus	0.022518175	2.115819339	7.19E-08	0.097427205
right Paraflocculus	0.099865109	1.784309003	1.50E-07	0.148955366
right Paragigantocellular reticular nucleus, dorsal part	0.008420693	2.228832685	5.37E-08	0.085878575
right Paragigantocellular reticular nucleus, lateral part	0.024336043	2.626436153	2.49E-08	0.055639731
right Paramedian lobule	0.110498642	1.171888372	1.00E-06	0.319032627
right Paranigral nucleus	0.001629252	2.097960155	7.77E-08	0.099239787
right Parapyramidal nucleus	0.003430008	2.68924526	2.19E-08	0.052815276
right Parasolitary nucleus	0.000728881	1.013194137	2.57E-06	0.378443378
right Parastrial nucleus	0.002426728	2.461038227	3.32E-08	0.066342613
right Parasubiculum	0.040028269	2.795486707	1.86E-08	0.047720246
right Parasubthalamic nucleus	0.006937206	4.388014537	6.01E-09	0.014437957
right Parataenial nucleus	0.010238543	3.087842726	1.30E-08	0.036160924
right Paratrigeminal nucleus	0.003781595	2.28323026	4.85E-08	0.080320733

right Paratrochlear nucleus	0.000883229	3.01576435	1.42E-08	0.038645663
right Paraventricular hypothalamic nucleus	0.021334647	3.580130479	9.03E-09	0.022616376
right Paraventricular nucleus of the thalamus	0.012665215	1.538014706	2.88E-07	0.203947567
right Parvicellular motor 5 nucleus	0.000308712	0.224249155	1.61E-03	0.836450969
right Parvicellular reticular nucleus	0.081197305	3.08970268	1.31E-08	0.036160924
right Pedunculopontine nucleus	0.038982125	3.82854827	7.53E-09	0.019973111
right Perifornical nucleus	0.009689804	3.280933201	1.10E-08	0.030147901
right Peripeduncular nucleus	0.003181345	3.833875939	7.50E-09	0.019973111
right Perireunensis nucleus	0.006439865	4.073438459	6.80E-09	0.017198719
right Perirhinal area	0.035457855	3.7586888	8.03E-09	0.02076658
right Peritrigeminal zone	0.012596738	2.300205905	4.73E-08	0.078526363
right Periventricular hypothalamic nucleus	0.014166055	1.849230313	1.36E-07	0.135686096
right Piriform area	0.367542842	2.441832895	3.58E-08	0.067403702
right Piriform-amygdalar area	0.037455636	2.756200073	2.01E-08	0.049162557
right Pontine central gray	0.012468115	1.721735865	1.81E-07	0.160649346
right Pontine gray	0.024704527	1.382145916	4.73E-07	0.247351469
right Pontine reticular nucleus	0.087268363	3.020860868	1.39E-08	0.038625783
right Pontine reticular nucleus, caudal part	0.080751325	3.226370063	1.20E-08	0.030463735
right Posterior amygdalar nucleus	0.041528834	4.406799949	6.08E-09	0.014437957
right Posterior auditory area	0.021206185	2.251973684	5.23E-08	0.083049896
right Posterior complex of the thalamus	0.064715973	3.07912769	1.32E-08	0.036540586
right Posterior hypothalamic nucleus	0.029712635	2.474513061	3.25E-08	0.06530843
right Posterior intralaminar thalamic nucleus	0.00667998	2.53339379	2.85E-08	0.062001701
right Posterior limiting nucleus of the thalamus	0.007160153	2.734123291	2.06E-08	0.050509999
right Posterior pretectal nucleus	0.004810621	2.155379181	6.37E-08	0.093466842
right Posterior triangular thalamic nucleus	0.010204305	3.260521099	1.14E-08	0.030289131
right Posterodorsal preoptic nucleus	0.000565954	2.349053275	4.35E-08	0.073851424
right Posterodorsal tegmental nucleus	0.000265829	0.250848081	9.83E-04	0.818244723
right Posterolateral visual area	0.040422884	2.26270316	5.06E-08	0.082277609
right posteromedial visual area	0.041211806	1.906674445	1.21E-07	0.12557058
right Postpiriform transition area	0.025287663	1.643914171	2.22E-07	0.177558531
right Postrhinal area	0.053688581	2.203030863	5.71E-08	0.088393232
right Postsubiculum	0.052805226	3.897697426	7.17E-09	0.019764937
right Precommissural nucleus	0.004776272	1.528257305	3.07E-07	0.205705264

right Prelimbic area	0.055497808	2.40436606	3.83E-08	0.069587455
right Preparasubthalamic nucleus	0.000523075	1.307020306	6.04E-07	0.271454151
right Presubiculum	0.036349569	3.684935358	8.41E-09	0.021285691
right Primary auditory area	0.081283071	2.931603139	1.52E-08	0.042556436
right Primary motor area	0.264746622	3.337813855	1.05E-08	0.028794324
right Primary somatosensory area, barrel field	0.173945696	1.797011784	1.46E-07	0.146391269
right Primary somatosensory area, lower limb	0.068720554	2.919508316	1.57E-08	0.042662245
right Primary somatosensory area, mouth	0.150552289	3.438328775	9.86E-09	0.02587069
right Primary somatosensory area, nose	0.08055418	2.567834343	2.66E-08	0.059929596
right Primary somatosensory area, trunk	0.046202539	2.108217537	7.33E-08	0.098142521
right Primary somatosensory area, unassigned	0.030227117	2.143771655	6.48E-08	0.094782105
right Primary somatosensory area, upper limb	0.098124492	2.646577835	2.44E-08	0.054589968
right Primary visual area	0.239391262	1.708746553	1.86E-07	0.163517816
right principal mammillary tract	0.001114766	1.395841629	4.57E-07	0.242998782
right Principal sensory nucleus of the trigeminal	0.027929073	1.587798679	2.49E-07	0.191667464
right Prosubiculum	0.062091822	3.859431429	7.47E-09	0.019935335
right pyramid	0.010195633	1.914962742	1.20E-07	0.124062713
right Red nucleus	0.030810234	3.112175844	1.27E-08	0.035574776
right Reticular nucleus of the thalamus	0.084129404	2.888378526	1.61E-08	0.044360911
right Retrochiasmatic area	0.002529621	0.847320143	5.02E-06	0.458982126
right Retroparafascicular nucleus	0.002581094	2.431888186	3.61E-08	0.067829432
right Retrosplenial area, dorsal part	0.102352095	1.845361323	1.39E-07	0.136046793
right Retrosplenial area, lateral agranular part	0.077270022	2.000307277	9.63E-08	0.111660554
right Retrosplenial area, ventral part	0.070187376	1.227791479	8.39E-07	0.297737982
right Rostrolateral area	0.029095352	1.478069596	3.60E-07	0.21889705
right rubrospinal tract	0.011156191	2.300358744	4.69E-08	0.078526363
right Secondary motor area	0.25353036	2.333978383	4.46E-08	0.075270723
right sensory root of the trigeminal nerve	0.01905383	2.642289321	2.42E-08	0.054589968
right Septohippocampal nucleus	0.001757888	1.748159041	1.66E-07	0.155801917
right Simple lobule	0.161769025	2.044357368	8.77E-08	0.105469342
right solitary tract	-0.000377301	-1.184306234	9.59E-07	0.314317324
right Spinal nucleus of the trigeminal, caudal part	0.05500909	3.354162296	1.04E-08	0.028230329
right Spinal nucleus of the trigeminal, interpolar part	0.051982228	1.99025138	9.94E-08	0.112905854
right Spinal nucleus of the trigeminal, oral part	0.034085913	2.761783449	1.97E-08	0.04911216

right spinal tract of the trigeminal nerve	0.057718708	3.138530193	1.26E-08	0.034100839
right Spinal vestibular nucleus	0.02687428	2.965973505	1.48E-08	0.040846393
right stria medullaris	0.01570087	4.167346166	6.46E-09	0.016557529
right stria terminalis	0.018393386	2.750664662	2.02E-08	0.049416763
right Subceruleus nucleus	0.000908951	1.318596949	5.81E-07	0.267622454
right subependymal zone	0.008017636	3.021251156	1.39E-08	0.038625783
right Subgeniculate nucleus	0.000231527	0.439937351	4.71E-05	0.699383233
right Subiculum	0.100662442	3.617018691	8.78E-09	0.022103493
right Sublaterodorsal nucleus	0.000720305	0.892942243	4.30E-06	0.435077659
right Submedial nucleus of the thalamus	0.018727914	3.743598447	7.96E-09	0.02076658
right Subparafascicular area	0.0072545	2.800374636	1.84E-08	0.047643948
right Subparafascicular nucleus	0.013274187	4.947515564	5.27E-09	0.012947224
right Subparaventricular zone	0.005933937	2.512321907	2.98E-08	0.063283236
right Substantia innominata	0.081042536	2.296661867	4.77E-08	0.078826124
right Substantia nigra, compact part	0.00561668	1.750475114	1.64E-07	0.155548595
right Substantia nigra, reticular part	0.039462546	1.960767923	1.07E-07	0.117069699
right Subthalamic nucleus	0.007254479	3.781711442	7.99E-09	0.02076658
right Superior central nucleus raphe	0.02744875	3.717661624	8.10E-09	0.02076658
right superior cerebelar peduncles	0.028443416	3.364702745	1.03E-08	0.028089985
right Superior colliculus	0.258092534	2.780373271	1.92E-08	0.04822422
right Superior olivary complex, lateral part	0.004716283	1.614884753	2.35E-07	0.184786067
right Superior olivary complex, medial part	0.003395718	3.264476406	1.12E-08	0.03026965
right Superior olivary complex, periolivary region	0.00768323	2.01001749	9.43E-08	0.110269135
right Superior vestibular nucleus	0.015623745	2.832195957	1.74E-08	0.046507899
right Supplemental somatosensory area	0.247442829	2.40124194	3.89E-08	0.069587455
right supra-callosal cerebral white matter	0.051072952	4.704703343	6.14E-09	0.014437957
right Suprachiasmatic nucleus	0.002735436	2.3891874	4.02E-08	0.070543326
right Suprageniculate nucleus	0.006268368	3.76816084	7.88E-09	0.02076658
right Supragenual nucleus	0.000180075	0.622519521	1.87E-05	0.581800964
right Supramammillary nucleus	0.009158123	2.495857219	3.02E-08	0.064654827
right Supraoculomotor periaqueductal gray	0.000531652	0.88301554	4.47E-06	0.440128543
right supraoptic commissures	8.58E-05	0.300032331	3.74E-04	0.786080484
right Supraoptic nucleus	0.001294827	1.605668465	2.42E-07	0.186890782
right Supratrigeminal nucleus	0.009569727	2.327549787	4.54E-08	0.07575441

right Taenia tecta, dorsal part	0.015434976	1.181718046	9.81E-07	0.315009028
right Taenia tecta, ventral part	0.009372526	0.869556411	4.64E-06	0.447263948
right Tegmental reticular nucleus	0.033356969	2.803745222	1.83E-08	0.047643948
right Temporal association areas	0.116629502	3.062033853	1.35E-08	0.036839461
right trapezoid body	0.003318539	1.110792386	1.45E-06	0.340286244
right trochlear nerve	-0.000120049	-0.690338886	1.07E-05	0.544601125
right Trochlear nucleus	0.000197232	0.429479208	5.59E-05	0.705171856
right Tuberal nucleus	0.002615361	0.276768074	6.43E-04	0.800606464
right Tuberomammillary nucleus, dorsal part	0.001003286	1.291256854	6.28E-07	0.277063096
right Tuberomammillary nucleus, ventral part	0.001020408	0.38883421	1.00E-04	0.729450674
right uncinate fascicle	0.002126636	0.996279856	2.93E-06	0.385028739
right Ventral anterior-lateral complex of the thalamus	0.045945051	3.964369281	6.98E-09	0.018762516
right Ventral auditory area	0.072364945	3.420985774	9.96E-09	0.026342661
right Ventral cochlear nucleus	0.032928258	2.442955136	3.47E-08	0.067403702
right Ventral medial nucleus of the thalamus	0.067528453	3.641489486	8.65E-09	0.021637953
right Ventral part of the lateral geniculate complex	0.018179089	3.020856122	1.40E-08	0.038625783
right Ventral posterolateral nucleus of the thalamus	0.052453369	2.548184822	2.75E-08	0.061153979
right Ventral posteromedial nucleus of the thalamus	0.130992291	3.272190041	1.12E-08	0.030151432
right Ventral premammillary nucleus	0.001234782	0.404289216	7.37E-05	0.720900371
right ventral spinocerebellar tract	0.005822476	2.055152413	8.42E-08	0.104397993
right Ventral tegmental area	0.018144858	2.703292424	2.15E-08	0.052014442
right Ventral tegmental nucleus	0.001226231	1.995126683	9.83E-08	0.112349479
right Ventrolateral preoptic nucleus	0.001877936	1.569052081	2.64E-07	0.196319964
right Ventromedial hypothalamic nucleus	0.002435301	0.33340024	1.80E-04	0.765991453
right Ventromedial preoptic nucleus	0.001037579	1.13341725	1.34E-06	0.331003262
right vestibular nerve	0.016155388	4.237515031	6.27E-09	0.015563882
right Vestibulocerebellar nucleus	0.005265093	2.694205728	2.18E-08	0.05252034
right Visceral area	0.077887483	2.788892267	1.89E-08	0.047945195
right vomeronasal nerve	0.000557374	1.242675196	7.70E-07	0.293120038
right Xiphoid thalamic nucleus	0.00072031	0.316175033	2.36E-04	0.776959928
right Zona incerta	0.076737946	4.189306368	6.43E-09	0.016557529
root	0.170677171	2.579449335	2.62E-08	0.059053541
Rostral linear nucleus raphe	0.006448451	3.361294534	1.03E-08	0.028097568
Septofimbrial nucleus	0.041537424	4.058492143	6.71E-09	0.017198719

Subcommissural organ	0.001054718	1.791806071	1.48E-07	0.147386595
Subfornical organ	0.00330139	3.300297815	1.08E-08	0.029604202
superior cerebellar peduncle decussation	0.004073145	2.846828242	1.70E-08	0.045987579
superior colliculus commissure	0.001423453	2.179594158	6.19E-08	0.090213194
third ventricle	0.098835748	4.580543771	5.79E-09	0.014437957
Triangular nucleus of septum	0.036229519	3.860996172	7.33E-09	0.019935335
Uvula (IX)	0.160482148	1.145915017	1.18E-06	0.327633191
Vascular organ of the lamina terminalis	-0.000248671	-0.596032071	1.99E-05	0.599001937
ventral hippocampal commissure	0.004561938	2.189534812	5.97E-08	0.089470194
ventral tegmental decussation	0.002864065	2.357205366	4.25E-08	0.073470476
